# Analyzing determinants of social practices in infectious diseases among Indigenous and Afro-Colombian communities: A participatory diagnosis of malaria, tuberculosis, and leishmaniasis in Colombia

**DOI:** 10.1371/journal.pgph.0004918

**Published:** 2025-07-08

**Authors:** Martha Milena Bautista Gomez, Laura Sofia Zuluaga, Marcos Medina Tabares

**Affiliations:** 1 Centro Internacional de Entrenamiento e Investigaciones Médicas (CIDEIM), Cali, Colombia; 2 Universidad Icesi, Cali, Colombia; Universidad de Las Americas, ECUADOR

## Abstract

Infectious diseases pose major public health challenges worldwide, particularly in developing countries, where their impact is more severe. This article presents a participatory social diagnosis to analyze the determinants of health-related social practices associated with infectious diseases (malaria, tuberculosis, and leishmaniasis) among Indigenous and Afro-Colombian communities in Pueblo Rico, Colombia. We explore how social determinants, attitudes, and knowledge influence health-related practices. Our findings show that each disease is shaped by a different dominant factor: malaria by structural conditions, leishmaniasis by attitudes, and tuberculosis by limited knowledge, while all are affected by distrust in the health system and low awareness of self-care. We argue that health practices are complex, historically structured social practices, so their change requires a long-term holistic health approach. Through this research, we seek to understand health practices related to infectious diseases and to inform the design of more effective and culturally grounded interventions.

## Introduction

Infectious diseases have historically been a leading cause of death worldwide. As such, they represent a major public health challenge for the world, particularly in developing countries where their impact is more severe [1–4]. Colombia reports high rates of tuberculosis (TB), malaria, and cutaneous leishmaniasis (CL) [5,6]. Controlling these diseases is especially difficult due to their prevalence in rural populations with high levels of poverty across low- and middle-income countries. These areas are often affected by complex social factors, such as political instability, armed conflict, and challenging geographical conditions, posing barriers to the control of infectious diseases mainly due to their negative impact on people’s access to healthcare [7]. These factors easily intertwine with demographic characteristics such as ethnicity, gender and race, and make the situation more complex.

The municipality of Pueblo Rico (Risaralda, Colombia), where the study was conducted, is endemic to various infectious diseases. According to national statistics [8], the minimum of cases of CL was recorded in 2008 with 33 reported cases. Since then, there was a wave that peaked in 2016 with 417 cases, reaching 186 cases in 2024 [9]. In the case of malaria, after a period with fewer than 100 cases during 2017 and 2018, the cases increased dramatically, reaching 8 588 cases in 2024 [9]. For TB, 2022 registered the highest number of cases since 2007, with 22 cases and an incidence rate of 113.5 per 100 000 inhabitants. Our previous studies have shown that the control and prevention of these infectious diseases involves geographical barriers, environmental conditions, limited health education and cultural barriers. Moreover, there is a link between these diseases and both ethnicity and rurality, as the population most affected are indigenous and afro-Colombian communities who inhabit remote rural areas and face higher levels of poverty, social exclusion, and violence in the municipality [10].

While there is a growing recognition of the importance of the social approach in shaping infectious disease outcomes, comprehensive analysis of social practices remains limited. From behavioral psychology, various theoretical models suggest that behavioral change can be achieved by modifying specific components. For instance, the COM-B model identifies these as capacity, opportunity, and motivation [11], while the KAP model focuses on knowledge, attitudes, and practices, using a standardized survey. From a psychosocial perspective, the experiences and meanings attributed to diseases, the social determinants of health, and the value of participatory approaches have been analyzed through qualitative methods [12]. Meanwhile, social studies have mainly addressed the connection between infectious diseases and various perspectives of vulnerability, including structural violence, social inequality, and social capital [13].

This study analyzes the social practices in infectious diseases through the lens of social theory, drawing on Bourdieu’s notion of habitus, as structured patterns of action shaped by past experiences and social conditions [13]. Rather than isolated, conscious decisions, these practices are historically embedded ways of acting, thinking, and being, reinforced through daily repetition, and therefore tend to persist over time, remaining relatively stable unless disrupted by significant external events. Within this framework, the analysis of social practices in infectious diseases was operationalized following Shove, Pantzar, and Watson [14] in three dimensions: material conditions, understood as constraining factors to action related to social determinants of health; competences, in terms of knowledge, skills, and abilities; and meanings, which encompass shared social attitudes, experiences, and perceptions.

The objective of this article is to analyze the determinants of social practices associated with infectious diseases (malaria, TB, and CL) among ethnic populations (Indigenous Embera and Afro-Colombian communities) in Pueblo Rico, Colombia. Through this research, we seek to understand the health-related social practices associated with infectious diseases and provide information to support the development of more effective interventions that consider these practices within their socio-health context, shaped by socially shared meanings and conditioned by the competences people need to make autonomous decisions about their health and to adapt to changing circumstances.

## Methods

### Ethics statement

To carry out this study, informed consent was obtained from all participants involved. The research was approved by the CIDEIM Ethics Committee with approval act number: FOR08039–02. Informed consent was provided in written form, with each participant’s signature and/or fingerprint. Consent from three Indigenous Cabildos and two Afro-Colombian Community Councils was obtained prior to the research.

### Methodology

A qualitative case study was conducted, using participatory diagnosis in infectious diseases as a research strategy in Pueblo Rico (Colombia). Participants were selected through purposive sampling to ensure representation of key stakeholders: healthcare workers, Afro-Colombian, and indigenous communities, of rural areas affected by infectious diseases.

According to the inclusion criteria (see Table 1), separate focus groups were conducted within the community for each population, incorporating insights from ancestral knowledge holders and community leaders. Recruitment was coordinated through three Indigenous Cabildos and two Afro-Colombian Community Councils, ensuring both participatory engagement and territorial coverage. For healthcare workers, those with direct community involvement were included, drawn from four main healthcare local institutions, as they possess the most comprehensive understanding of local health practices.

**Table 1:** Inclusion criteria according to participant type.

Participant type	Inclusion criteria
Community Leaders	• Traditional healers or midwives or herbalist or individuals with health training • People who self-identify as indigenous or Afro-Colombian • People who have lived in rural areas in Pueblo Rico, for more than 5 years • People of legal age
Health workers	• Workers from local health institutions in charge of the rural area • People of legal age • People who have lived or worked in Pueblo Rico for at least 5 years.

Prior to data collection, participants were informed about the study’s objectives, as well as the research team’s background and interests. In total, 31 people attended the focus group discussions (FGDs). Recruitment was successful in terms of participants from both communities and health institutions, as well as in ethnic and gender diversity. No participants declined to participate or dropped out after recruitment (See Table 2).

**Table 2:** Participants.

Focus Group	Ethnicity	Occupation	Gender
Health Institutions Focus Group Discussion	Mestizo (n = 6) Indigenous (n = 3) Afro-Colombian (n = 1)	Health worker (n = 10)	Men (n = 3) Women (n = 7)
Indigenous Focus Group Discussion	Indigenous (n = 9)	Health community leaders (n = 7) Traditional health worker (n = 1) Young person with health training (n = 1)	Men (n = 5) Women (n = 4)
Afro-Colombian Focus Group Discussion	Afro-Colombian (n = 12)	Health community leaders (n = 5) Traditional health worker (n = 1) Young person with health training (n = 6)	Men (n = 3) Women (n = 9)

Data collection was conducted through FGDs using didactic pedagogical tools that enable participants to collectively express their infectious disease practices, first visually and then in a more in-depth discussion. Each FGDs lasted approximately two hours, with a total of nine sessions —one for each disease (CL, TB, and malaria) and each group (Embera Indigenous community, Afro-Colombian community, and local health workers). The three sessions with health institutions were held in the local hospital in the urban area of Pueblo Rico, while the community sessions took place at the facilities of the Afro-Colombian Council in the rural area of the municipality. All sessions occurred between May 1st and May 5th, 2023, and were attended exclusively by the researchers and participants (see S1 Text).

**Fig 1:**
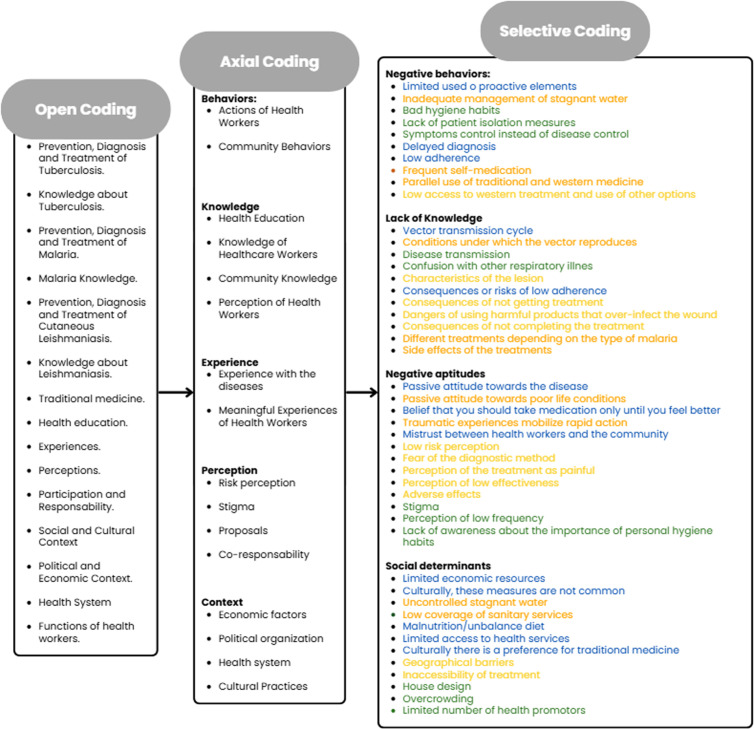
Grounded theory coding process.

FGDs were audio-recorded, transcribed, and then coded following grounded theory procedures [15] (Fig 1). Open coding involved assigning initial themes by assigning initial labels to segments of data. Axial coding grouped related codes into analytical categories based on thematic connections. Finally, selective coding refined the analysis by identifying negative influencers and comparing these factors across diseases. Atlas.ti software was used to structure and systematize the analysis (See S2 Text).

Dimensions (bold font), categories (standard font), category related to malaria in orange, related to CL in yellow, and related to TB in green, and category related to more than one disease in blue.

### Study trustworthiness

To ensure the trustworthiness of the study four criteria were included [16]. Credibility was supported through triangulation of participants’ perspectives, incorporating cultural and territory diversity approach from community and healthcare workers, and traditional and western medicine. In the data collection, pedagogical tools adequate for each type of participants were included to facilitate the visual expression and discussion. Moreover, a comparative analysis of the three diseases studied was conducted using a comprehensive theoretical approach to understand the social practices in infectious diseases through multidimensional analysis of competences, meanings and material conditions.

Dependability was ensured through the use of the Consolidated Criteria for Reporting Qualitative Research (COREQ) checklist (See S1 Table). [17]. Confirmability was addressed through dual reviews of the coding and analysis process in which two researchers worked independently, resolving discrepancies with a third researcher. As a result of the process described, the findings of this study may be transferable to similar rural contexts, characterized by cultural diversity and populations affected by infectious diseases in vulnerable settings.

### Inclusivity in global research

Additional information regarding ethical, cultural, and scientific considerations specific to inclusivity in global research is included in the Supporting Information (See S3 Text).

## Results

### Social practices in malaria

In terms of prevention, an unfavorable practice is the limited use of protective elements. Knowledge can be considered a minor factor in this practice, since participants showed awareness of the protective measures against the vector, although they lacked understanding of the vector’s transmission cycle (See Table 3). This limited use is related to a passive attitude, as most participants reported doing nothing to protect themselves from insects, despite the significant impact of the disease.

**Table 3:** Determinants of unhealthy social practices in malaria.

	Unhealthy practices	Knowledge gaps	Attitudinal barriers	Social determinants
Prevention	• Limited use of protective elements	• Vector transmission cycle	• Passive attitude towards the disease	• Limited economic resources • Culturally, insect repellent is not used
	• Inadequate management of stagnant water	• Conditions under which the vector reproduces	• Passive attitude towards poor living conditions	• Uncontrolled stagnant water • Low coverage of sanitary services
Treatment	• Low adherence to treatment	• Consequences of low adherence	• Belief that medication should be taken only until symptoms disappear	• Malnutrition/ unbalance diet
	• Frequent self-medication	• Different treatments depending on the type of malaria	• Traumatic experiences mobilize rapid action	• Limited access to health services
	• Parallel use of traditional and western medicine	• Side effects of the treatments	• Mistrust between health workers and the community	• Culturally there is a preference for traditional medicine

However, social determinants appear to be the main factor. The lack of economic resources limits the acquisition of some of these products, while cultural barriers also play a role. Some protective elements are considered foreign to the local culture. Additionally, the use of bed nets is often avoided due to discomfort caused by high temperatures in the area and the smell of insecticide, which discourages their use, or are repurposes for non-preventive use, as described below:

*“... the children were playing with the repellents…instead of giving them the use they should have,”* [Afro-Colombian woman, health worker. Focus group. May 2023].

Another unfavorable preventive practice is the inadequate management of stagnant water. Contributing factors include a lack of knowledge about the conditions in which the vector reproduces and the appropriate ways to manage breeding sites. For example, the community believes that garbage serves as breeding grounds for the vector. This is reinforced by a passive attitude toward living conditions and limited health awareness.

However, this practice is primarily a response to social determinants, including: i. environmental conditions related to the presence of water sources, such as lakes; ii. living conditions, including reliance on fishing as a main source of income and the need to store water in tanks or containers at home; and iii. a low level of government technical assistance to drain large amounts of unused or dirty water, as described in the following fragment:

*“... the indigenous people don’t... sometimes there is a lake near the house, let’s say, but since they hardly use it, since it is so dirty, they don’t clean that tank, and let’s say, malaria occurs. That’s why I say that that’s where it comes from [the malaria] [...]”* [Indigenous man, health worker. Focus group. May 2023]

Regarding treatment, one of the unfavorable practices is low adherence. People often take the medication without following medical instructions regarding dosage and duration. Knowledge is considered an important factor, as many are unaware of the consequences of low adherence. Attitudes also are an important factor, since people mistakenly believe that medication should be taken only until symptoms improve. Additionally, social determinants contribute to this practice. For instance, high level of malnutrition, particularly in the indigenous community, can lead to adverse effects when taking the medication. This issue is illustrated in the following quote:

*“There are many patients who only buy 3 doses and there are many indigenous people who buy medicine, improve their health and when their health is already improved, they save a small part of the medicine for another time, so they are not cured…”* [Indigenous man, community leader. Focus group. May 2023]

The second unfavorable practice related to treatment is frequent self-medication. When symptoms appear, people often go to the pharmacy first and purchase medications, which, according to the community and health workers, leads to delayed diagnoses, false negatives in tests, delayed treatment, and the risk of taking the wrong medication for the specific type of malaria. Frequent self-medication stems from the community’s lack of knowledge about the existence of different treatments depending on the malaria type. Additionally, a high perception of disease risk, combined with limited access to health services and the occasional unavailability of medication, motivates people to address the situation on their own, as evidenced in the following fragment:

*“… for me it is a good thing, because at that moment the hospital does not have the indicated treatment for malaria, so, if I have malaria and the hospital does not give me that treatment... what should I do? Go to the pharmacy and buy it” [Mestizo woman, health worker. Focus group. May 2023]*

A third unfavorable practice mentioned by participants is the frequent parallel use of traditional and Western medicine, such as taking herbal baths and bitter drinks made from medicinal plants while simultaneously undergoing Western treatments. The community exhibits a general attitude of distrust towards health institutions. Combined with social factors, such as ancestral practices and a preference for traditional medicine, this often leads people to try ancestral remedies first, or to combine it with Western medicine, as expressed below: “*I think that.... we have more faith in herbs*” [Afro-Colombian woman, community leader. Focus group. May 2023]

### Social practices in leishmaniasis

Regarding prevention, there is limited use of protective elements against the vector, similar as in the case with malaria. This is sometimes due to a lack of economic resources to purchase them, and cultural barriers that limit and discourage their use (See Table 4). Participants generally demonstrate a moderate level of knowledge, although there is a lack of understanding about the parasite-reservoir-sandflies-human transmission process. Furthermore, this limited use mainly reflects a passive community’s attitude towards the disease. In contrast to malaria-related practices, this is linked to a low-risk perception regarding the severity and prevalence of the disease, as described below:

**Table 4:** Determinants of unhealthy social practices in leishmaniasis.

	Unhealthy practices	Knowledge gaps	Attitudinal barriers	Social determinants
Prevention	• Limited use of protective elements	• Vector transmission cycle and its breeding sites	• Passive attitude towards the disease • Low risk perception	• Limited economic resources. • Culturally, insect protection barriers are not used
Diagnosis	• Delayed diagnosis	• Characteristics of the lesion	• Low risk perception • Perception of low efficiency of health system • Fear of the diagnostic method	• Limited access to health services
Treatment	• Limited access to western treatment and use of other options	• Consequences of not getting treatment • Risks of using harmful products that worsen the infection	• Mistrust between health workers and the community • Perception of low efficiency of the health system	• Culturally there is a preference for traditional medicine
	• Low adherence to treatment	• Consequences of not completing the treatment.	• Fear of injection-based treatment • Perception of low effectiveness • Fear of adverse effects • Low risk perception	• Geographical barriers • Barriers in the administration of treatment • Malnutrition/ unbalanced diet

“*[C-A01]: It is almost not heard about now [that there are cases of leishmaniasis], before we saw several people...[C-A09]: before yes, but not now... and in the indigenous communities we see more cases of leishmaniasis than in Afro communities* […]” [two Afro-Colombian women, community leaders. Focus group. May 2023].

In terms of diagnosis, delayed diagnosis was frequently mentioned. People often receive a diagnosis only after the lesion has grown, worsened, or failed to heal after considerable amount of time. This delay can primarily be understood as a result of limited knowledge about the characteristics of the lesion caused by the disease. As a result, people do not easily recognize it, mistake it for conditions such as allergies, fungal infections, or other unrelated issues, and associate it with symptoms that do not correspond to CL.

*“There is a mother who comes out with leishmaniasis and gets a pimple, there is a mother who does not know what that is, if it is leishmaniasis, one does not know, until it increases, then she does worry”* [Indigenous man, community leader. Focus group. May 2023]

Furthermore, delayed diagnosis is related to barriers linked to limited access to health services. Sometimes, the diagnostic test cannot be performed at the local health center due to a lack of trained health workers or to a shortage of rapid test supplies. As a result, people have to travel to the nearest hospital, which frequently requires long travel and incurs additional economic burdens.

There are also factors related to motivational barriers due to the low-risk perception regarding the severity and prevalence of the disease, as well as a perception of low efficiency of the health system based on previous negative experiences. As a result, many believe they won’t receive care, that it will take too long, or that they will be sent home un treated, so they don’t even attempt to access healthcare. These factors disincentivize people from seeking diagnosis. Moreover, fear of pain during the test and concerns about the aesthetic consequences of taking a sample from the lesion are additional minor factors contributing to delayed diagnosis.

In terms of treatment, an unfavorable practice is the low access to Western medical treatment and the use of alternative options. This can be explained by a lack of knowledge about the consequences of not receiving treatment and the risks associated with harmful remedies that can worsen the wound, such as sulfur, battery acid, charcoal, pen ink, among others. This practice is also influenced by mistrust towards health workers due to past experiences of mistreatment, and by a perception of inefficiency in the health system, which discourages people from seeking care in the first place. Additionally, a cultural preference for home remedies often leads them to try other solutions first. Some of this is expressed in the following fragment:

*“Sulfur, a spoonful of sulfur and a little piece of charcoal, well burned, and a little legume that kills cattle worms, I prepared that, the 4 little things, you wash the [leishmaniasis] wound well... that medicine destroys everything, applying it only three times...”* [Indigenous man, community leader. Focus group. May 2023]

A second unfavorable practice is low adherence to treatment, primarily due to people’s low perception of CL risk. It is also related to a perception that the treatment has low effectiveness, fears that it may be long and painful, and concerns about possible adverse effects, particularly in communities with widespread malnutrition and poor dietary balance. As mentioned in the following fragment:

*“[I-M06]: There is a lot of desertion. They do not finish treatment, because it hurts a lot and there are many injections. Also, the pills are very strong, they are strong, and they cause gastritis... they once gave them to a lady, and she said [...] that they made her sick”* [Mestizo woman, health worker. Focus group. May 2023].

This practice can also be understood through social determinants. There are barriers to accessing treatment, since the intrahospital administration usually lasts for 20 days, and providing it in a rural context with 100 villages and only two health centers pose a significant logistical challenge. Moreover, the duration of the treatment and the fact that it must be received at the health center means that patients often face travel times of up to eight hours, and high economic costs, or they must find a place to stay closer to the health center. Lastly, to a lesser extent, low adherence is also partially driven by limited understanding about the consequences of not getting treatment or not completing the treatment, such as failing to eliminate the parasite that causes CL or developing more lesions.

### Social practices in tuberculosis

In terms of prevention, one of the unfavorable practices is poor hygiene habits, which include inappropriate use of surgical masks, not covering the mouth and nose when coughing or sneezing and not washing hands afterwards (See Table 5). This practice is primarily due to cultural barriers, especially within the Indigenous community, since these habits are foreign to their culture and are therefore often not adopted. Moreover, there is a lack of awareness about the importance of hygiene habits, which further hinders the adoption of these practices. Lastly, to a lesser extent, poor hygiene habits are partially due to confusion about how TB is transmitted, which in turn leads to confusion about how to prevent the disease. As described below:

**Table 5:** Determinants of unhealthy social practices in tuberculosis.

	Unhealthy practices	Knowledge gaps	Attitudes barriers	Social determinants
Prevention	• Inappropriate hygiene habits	• Disease transmission	• Lack of awareness about the importance of personal hygiene habits	• Culturally, these measures are not common.
	• Lack of patient isolation measures	• Disease transmission		• House design • Overcrowding
Diagnosis	• Symptom control instead of disease control	• Confusion with other respiratory illnesses	• Perception of low frequency • Mistrust between health workers and the community	• Culturally there is a preference for home remedies
	• Delayed diagnosis	• Confusion with other respiratory illnesses	• Stigma	• Limited number of health promotors
Treatment	• Low adherence to treatment	• Risks of low adherence	• Belief that medication should be taken only until symptoms disappear • Long treatment • Adverse effects	• Malnutrition/ unbalanced diet

*“When they sneeze, they are never going to cover themselves. They will never protect themselves with anything and the masks, they sneeze with the masks, then they lend them to the other person, and there it is”* [Afro-Colombian woman, health worker. Focus group. May 2023]

The second unfavorable practice in prevention is the lack of isolation measures for TB patients. This is mainly a consequence of social determinants. Implementing isolation or social distancing is very difficult due to overcrowding, especially within the Indigenous community. Knowledge is also a factor that impacts this unfavorable practice. There is confusion about how TB is transmitted and a lack of awareness about the need to isolate patients, as is evidenced below:

*“Well, how it is transmitted, it is transmitted by air, when a person coughs or sneezes, or also when this already infected person manipulates elements or objects, since these particles remain in these objects or elements and a healthy person arrives and uses them and becomes infected”* [Afro-Colombian man, community leader. Focus group. May 2023]

A common unfavorable practice is that people focus on symptoms management rather than disease control. This means they buy medication at the pharmacy or use home remedies, such as homemade drinks and medicinal baths, to treat flu-like symptoms without getting a diagnosis. This practice responds to a cultural preference for home remedies and to the mistrust that exists between the community and health workers, which leads people to try other treatment options first. It is also influenced by the perception that the disease is not very common in the community. Above all, however, this practice is a consequence of difficulty distinguishing TB symptoms from those of common respiratory infections. As mentioned in the following quote:

*“The symptoms are flu-like, so what do people do? They go to the pharmacy and buy things for the flu, they get shots for the flu. Here people feel discomfort and go and get an injection because they don’t know what it is and it makes them feel better and all that, so they go to the pharmacy*” [Afro-Colombian woman, community leader. Focus group. May 2023]

Regarding diagnosis, an unfavorable practice is diagnostic delays, which can be primarily understood as a result of limited knowledge about the symptoms of TB. Furthermore, it is related to social determinants, specifically access barriers, since people must travel to the nearest hospital to get the diagnostic test, incurring expenses and long travel times. It is also related to the perception that TB is not frequent in the community. Additionally, delayed diagnosis responds to the stigma surrounding TB, as it is often associated with sex work and homelessness, especially within the Afro-Colombian community. As evidenced in the following quote:

“*They are going to say the town whore [if they hear that they have tb]. They are going to say that she went to a whorehouse, the cases of tuberculosis that have confirmed have been from people who live on the streets, because they know that that is the stigma, we relate it to being a good for nothing*” [Afro-Colombian man, community leader. Focus group. May 2023]

In terms of treatment, the unfavorable practice is low adherence. Health worker participants mentioned that people often do not finish the treatment, and in some cases, people reportedly sell or share their medication. This can be understood as a result of limited knowledge about the risks associated with not accessing correct and timely treatment. Additionally, a negative perception of the treatment plays a key role, as participants mentioned that the treatment is long, the medication is strong, and it can cause adverse effects, especially for malnourished patients, a condition that is prevalent in Pueblo Rico. As mentioned in the fragment below:

*“There are some people who become drug resistant. Because they get lost a lot, they start [the treatment], they get lost, they start again, they get lost […] because they get better with the first treatment”* [Mestizo woman, health worker. Focus group. May 2023]

## Discussion

### Overview of malaria, leishmaniasis and tuberculosis

The results suggest that the health-related practices for malaria in Pueblo Rico are primarily influenced by social determinants. The prevalence of widespread self-medication is driven by barriers to accessing health services. Consistently with evidence reported in Kenya and Tanzania [18], the long distance to health facilities and the lack of availability of medicines likely contribute to non-formal care-seeking, irrational drug use, and the presence of substandard antimalarial drugs in the market. Furthermore, similar to studies conducted in India and Ethiopia [10,19], poor living conditions in Pueblo Rico increase exposure to risk factors, such as limited access to water and housing design, which complicate vector control [19,20], and malnutrition hinders treatment adherence [21]. By contrast, the population in Pueblo Rico demonstrates a medium-high level of knowledge and high-risk perception due to the frequent occurrence of the disease in the community.

As is documented in other studies [22], the results suggest the unfavorable practices related to CL are primarily shaped by community attitudes. The population’s low risk perception may underlie the limited adoption of preventive measures and delayed diagnosis. Also, the preference for traditional treatments and low treatment adherence are strongly influenced by perceptions of low health system efficiency and low treatment effectiveness based on negative past experiences. This is consistent with research conducted in Bolivia [23], which report how cultural, economic, and geographical barriers discourage the appropriate use of health services and discourage effective health-seeking behaviors. Furthermore, in line with findings from Guyana [24], knowledge emerges as the least influential factor in these practices. Although participants have a medium level knowledge about CL, it seems that this is not sufficient to drive changes in their social practices.

Concerning TB, knowledge emerges as the primary factor influencing the population’s unhealthy practices. Consistent with studies that highlight how limited knowledge results in underutilization of health services [25,26], delays in seeking diagnosis [27], and negatively affects TB patients’ willingness to seek healthcare [13], the data indicates the significant lack of knowledge about TB leads to its confusion with other diseases and drive most of the unhealthy practices identified. However, these unfavorable practices are also linked with meanings and attitudes associated with the disease. For instance, low awareness of the importance of hygienic measures contributes to continued transmission, while stigma and the perception that TB is not frequent contribute to delays in seeking diagnosis and treatment, and the unpleasantness of the challenging nature of the treatment discourages adherence [27,28]. Finally, poverty-related social determinants, such as overcrowding and malnutrition, along with structural limitations of the healthcare system, further worsen TB-related practices.

### Insights to promote healthy practices in infectious diseases

Social practices in health are complex and historically structured processes, which is why the adoption of new practices often requires a long period of time [29]. As social theory explains, these practices result from the interconnections between competences, meanings, and materials or artefacts [27]. These practices are part of the lived experience of everyday life and emerge from the intersection between the social structure and the actors’ agency, shaped by individual’s competences and meanings around diseases.

Meanings of health, well-being, and care practices within groups’ worldview play a crucial role in the adoption of health practices [30,31]. As shown in this study, fear, along with negative perceptions and opinions, underlies some of the unfavorable health practices. Several studies on culturally tailored interventions have reported favorable outcomes in promoting changes in health-related social practices [32–34], whereas a mismatch between health interventions and cultural norms can hinder the adoption of such practices [35]. In the case of Pueblo Rico, our previous study indicated that Indigenous communities perceive well-being as a balance between the spiritual, territorial, and community spheres, which can serve as a driver for adopting community-based care and environmental practices. Meanwhile, for Afro-Colombian communities, well-being tends to be more practical than spiritual and more individual than communal, making their knowledge about diseases and some of their usual prevention and control practices key drivers [11].

Social determinants of health establish a particular context in which the necessary material conditions to promote the adequate use of health services should be provided. As observed in the case of Pueblo Rico, poor living conditions expose the population to multiple risk factors, complicating disease control and diminishing the effectiveness of treatments. Additionally, structural poverty and social exclusion, often linked to limited access to education and the urgency of meeting basic survival needs, frequently lead to passive attitudes and reduce the community’s capacity for agency in improving health conditions [36]. Moreover, negative community experiences stemming from the limitations of the local health system have fostered distrust and discouraged individuals from following health advice or seeking health services [9].

Lastly, competencies provide knowledge and skills necessary to take action [37], thus, their absence inevitably leads to unhealthy practices. However, competencies alone are insufficient to drive change. As other studies noted, if knowledge alone were enough to address challenges related to healthy practices, no one would likely smoke or overeat to the point of obesity [38]. Similarly, our results showed that the community had high and medium levels of knowledge about malaria and CL, respectively, but unfavorable practices persisted. In other words, while competencies and capabilities are essential for promoting changes in health-related social practices, they are not sufficient if they do not resonate at a personal level or if the material conditions required to support them are absent.

### Limitations

This study, designed as a case study, prioritizes analytical depth over representativeness, so that the credibility supported by purposive sampling and triangulation allows transferability of its findings to other contexts with similar settings. However, similar future studies in comparable contexts would enhance the confirmability of the study, while the development of cross-case analyses would enhance its transferability. Furthermore, quantitative and interdisciplinary studies would provide a broader approach to understanding the phenomenon under study.

## Conclusion

The unhealthy practices associated with infectious diseases in Pueblo Rico indicate that malaria is primarily influenced by social determinants, where the shortage of medicines fosters irrational drug use, and poor living conditions expose people to various risk factors. Leishmaniasis is mostly shaped by attitudes, with a low perception of risk leading to delayed diagnosis, and the perception of low treatment efficiency resulting in low adherence and a preference for traditional or non-Western medicine. Tuberculosis is predominantly impacted by a significant lack of knowledge, which, combined with attitudinal barriers such as stigma and the perception of the disease’s low frequency, often leads to underutilization of health services and delays in diagnosis. For all diseases, negative experiences stemming from limitations in healthcare quality, access, and coverage discourage the use of services and create distrust in health systems, while low awareness of the importance of self-care hinders the adoption of preventive practices.

For the prevention and control of infectious diseases, it is crucial to understand social health practices as historically structured and embedded in everyday practices that result from the interaction between social structure and individual agency. Therefore, long-term holistic health interventions are required. These interventions must be feasible within setting limitations and grounded in the cultural drivers of each social group. They should ensure that the material conditions for action are in place, while also fostering the development of competencies and capacities necessary for informed decision-making and addressing the meanings and attitudes surrounding diseases that influence the adoption of healthy practices.

## Supporting information

S1 Text Methodological Guide used to conduct the focus group discussions described in the study. (PDF)

S2 Text Final coding matrix of focus group discussions detailing the thematic categories and subcategories used in the analysis. (PDF)

S3 Text Inclusivity in Global Research Questionnaire. (DOCX)

S1 Table COREQ Checklist, the consolidated criteria for reporting qualitative research (32-item checklist). (XLSX)
