# Supplementary material for: Analyzing determinants of social practices in infectious diseases among Indigenous and Afro-Colombian communities: A participatory diagnosis of malaria, tuberculosis, and leishmaniasis in Colombia
Source: PLOS Glob Public Health. 2025 Jul 8;5(7):e0004918. doi: 10.1371/journal.pgph.0004918 (PMC12237040; doi:10.1371/journal.pgph.0004918)
Supplement: S1 Text — (PDF) [file pgph.0004918.s001.pdf]

SOCIAL DIAGNOSIS OF INFECTIOUS DISEASES: METHODOLOGICAL GUIDE FOR FOCUS GROUPS OF INSTITUTION LEADERS

General objective: To conduct a participatory social diagnosis on the effectiveness of infectious disease prevention and control interventions currently being implemented in Pueblo Rico, in order to identify cultural barriers and facilitators.

Population: Local health workers in charge of rural áreas.

I. INTRODUCTION

- Icebreaker
- Presentation of CIDEIM and Research Team
- Presentation of the research and objective of the diagnosis
- Signing of the informed consent form
- Presentation of the day’s agenda
- Formation of groups

II. EXPERIENCE

- What does your work consist of?
- In which territories and with which populations do you work?

III. KNOWLEDGE

1. Health Education:

Participants are organized in pairs with the person next to them, they are given the questions written on a colored paper and have 10 minutes to answer them, then they must share their answer with the rest of the group and among all discuss them.

- **VISIT PLANNING** How do you organize visits to the community for workshops or talks? Who do you communicate with for the organization? At what times and places?
- **DESIGN OF WORKSHOP CONTENT.** What topics do you teach about infectious diseases prevention and control? How do you teach it? What resources do you use?What are the key points to keep in mind?
- **FULFILLMENT OF OBJECTIVES** How do you evaluate the unedrstanding of the topics? How can you know if in the long term the workshops conducted generate changes in the community?

2. Perceptions

Brainstorming What challenges do you face in teaching the community? In each of the following aspects:

- Use of audiovisual resources and artistic methodologies
- Participatory approach
- Comunication Barriers (Embera)
- Deep adaptations (level of schooling, socioeconomic barriers, incorporating ancestral medicine)

IV. BEHAVIORS AND PERCEPTIONS

1. Route of the institutions

The open questions that seeks to build the institutional route of the diease is asked and printed images that represent the route are taken so that participants can organize them. In addition, paper is provided so that if steps are missing, thay can draw them.

What is the route established by the health institutions to attend and control infectious diseases (Identify in each case the main actions and the actors involved)?

- Prevention
- Diagnosis
- Treatment
- Follow-up

2. Perception

2.1. On a scale of 1 to 5, how serious would you rate the infectious disease situation in Pueblo Rico? Why?

2.2. A horizontal line is drawn on the board, the line is divided into 2, one end represents what Works best and the other what Works least. Based on the institutional route, the actions carried out will be evaluated as follows:

- Which actions or measures of the route to prevent and control infectious diseases are easy or work bet in real life for patients ans which are more difficult or do not work? Put the images on the left the ones that do not work and on the right the one that do work.
- By consensus order from left to right from the actions that work the least to those that work the most.
- Why do you think these actions work or don’t work? (go deeper into the answer and try to investigate the cultural aspect)

SOCIAL DIAGNOSIS OF INFECTIOUS DISEASES: METHODOLOGICAL GUIDE FOR FOCUS GROUPS OF COMMUNITY LEADERS

General objective: To conduct a participatory social diagnosis on the effectiveness of infectious disease prevention and control interventions currently being implemented in Pueblo Rico, in order to identify cultural barriers and facilitators.

Population: 1) Community leaders who work as jaibanas, midwives and healers 2) Young people belonging to the communities with training in health.

I. INTRODUCTION

- Icebreaker
- Presentation of CIDEIM and Research Team
- Presentation of the research and objective of the diagnosis
- Signing of the informed consent form
- Presentation of the day's agenda
- Formation of groups

II. EXPERIENCE

- Have you or anyone close to you had some infectious diseases? How did it go?
- In your community, which infectious disease is common?

III. KNOWLEDGE

1. Knowledge assessment: Each participant will individually answer the questios and the socialize as a group. Answer the following questions using drawings:

- What is and how are Leishmaniasis, Tuberculosos ans Malaria transmitted?
- What signs or symptoms suggest that you or someone in the community may have Leishmaniasis, Tuberculosis and Malaria? (lesión with raised edges, lesión that does not cause pain, number of lesions, lesions on arms and legs)
- How are they prevented?
- How are they treated?

2. Perception:
3. Open-ended questions: Do you remember receiving workshops, trainings or visits from health workers to learn about infectious diseases? Tell me one in particular that you remember

4. Of the workshopsthat health workers conduct, what do you like, what don't like, what do you find difficult, how could they be improved, what would you like to learn.

IV. BEHAVIORS AND PERCEPTIONS

1. Scoial maps of the natural disease pathway. Printed images of all the diseases will be brought and people will have to organize and connect them as they answer the following questions:

- What do you do in your community to prevent infectious diseases?
  Images: use of long-sleeved clothing, use of repellents (chemical or natural), use of awnings or tarpaulins, garbage management – keep common spaces clean, avoid accumulation of wáter, and avoid wáter accumulation.
- What do you do in your community when you have symptoms of infectious diseases? Who do you ask for help?List by steps:
  Images: self-medication, hospital, home remedies, medicinal baths, take medicines with medical formula, jaibanás, yerbatero, teacher, moms, governor, health worker and ICBF, children, women, pergnant women, youth, adults. Blank colored paper in case it is necessary to draw,
  * How is adherence to treatment? What about prgnant women?
  * Why are some options chosen first over others?

2. Perceptions: Explore each of the teps pf the Nature Trail in depth:

- How do you assess the infectious disease situation in Pueblo Rico? Why?
- Which actions or measures work well to prevent and control infectious diseases in your community and which do not? Put on each action a red post-it if you think it doe not work well, a yellow one if it more or less Works, and a Green one of it Works very well.
- What new actions could be taken to improve the prevention and control of infectious diseases in your community?
