# Supplementary material for: Analyzing determinants of social practices in infectious diseases among Indigenous and Afro-Colombian communities: A participatory diagnosis of malaria, tuberculosis, and leishmaniasis in Colombia
Source: PLOS Glob Public Health. 2025 Jul 8;5(7):e0004918. doi: 10.1371/journal.pgph.0004918 (PMC12237040; doi:10.1371/journal.pgph.0004918)
Supplement: S2 Text — (PDF) [file pgph.0004918.s002.pdf]

ANEXO 1. Codificación total V2

Guía de codificación

Leishmaniasis cutánea

- Barreras de aptitud_LC
- Determinantes sociales_LC
- Malos comportamientos_LC
- Vacios de conocimiento_LC

Malaria

- Barreras de aptitud_ML
- Determinantes sociales_ML
- Malos comportamientos_ML
- Vacios de conocimiento_ML

Tuberculosis

- Barreras de aptitud_TB
- Determinantes sociales_TB
- Malos comportamientos_TB
- Vacios de conocimiento_TB

Proyecto (Codificación total v2)

Informe de códigos

Todos los (12) códigos

LEISHMANIASIS

○ Barreras de aptitud_ LC

46 Citas:

8:28 ¶ 19 – 20, in Salud

2:81 ¶ 154, in Indigena TB.docx

[C-I05]: No, pero hay unos también si no, también muy largo la fecha de la cita. La dan muy largo. Yo la pedí el mes de abril y me llegó el diez de junio. ¿Y a eso uno enfermo qué? Hasta la fecha esperando qué [inaudible]. Yo he ido siempre a donde el médico y realmente el paciente lleva enfermo, con esa cita no se cura. ¿Qué se va a curar? Se muere. [inaudible] Que allá uno hace atender, pero, pero, metiendo candela y eso es muy maluco, pero toca así, porque si no se muere la familia de uno, mi hermanito murió así. Igualito. Vino a la consulta. No, usted que está bien, más o menitos, saque cita. ¿Cuándo? Dizque para tres meses. No, no dijo nada, mi hermanito era poca palabra, entonces se fue para la casa. Siguió la fiebre y allá le dio el derrame y se acabó de joder.

8:29 ¶ 17 – 18, in Salud

2:78 ¶ 132, in Indigena TB.docx

[C-I05] Con la práctica que hemos hecho, caso especial, hace dos semanas, una señora venía con un cólico fuerte. Resulta que la llevamos al hospital al centro de salud. El médico me dijo que yo te voy a tomar signo vital y si aparece signo vital, bien, normal, hay que pedir cita. Entonces yo devolví en esta manera: "Doctor, si el signo vital normal, pero en dolor lo lleva ahí el paciente, ¿Qué hacemos? HÁgame el favor, consulten esa señora". Y me insistió otra vez: hay que sacar cita. Y teniendo el dolor ahí. Y yo no retire del hospital. Yo me quedé ahí. Hagamos una cosa. Yo automáticamente le llame al gerente. Es que él es un médico y a él tiene un padre quien manda. En ese hemos tenido error. Llamé al gerente, al grande allá. Me preguntó quién es esa, tal señor. Y el paciente es tal. Ah, entonces un momentico... Me atendieron. Me atendieron. En buena forma ya, porque yo me tocó.. Al ratico, en una observación se vomitó en un momentico, cuatro vómitos. ¿Qué tal en el camino se me vomita? ¿No se me muere? En ese hemos tenido, porque uno no tiene pacientes que no tienen conocimiento, lo que yo estoy hablando, no lo hablan así mismo. En ese hemos tenido error.

8:32 ¶ 25 – 28, in Salud

2:108 ¶ 102 – 104, in Indigena TB.docx

[C-I04]: El año pasado, acá en hospital que trabajé dos meses en la traductora, el niño era grave. Tenía mucha asfixia, venía de la comunidad de Piedra y ahí se pasó. Y la mamá lo llevó a hospital. Y el niño que estaba asfixiado, tosía, todo. Y el médico le mandó para la casa. No, que el niño que estaba bien, estaba bien. No, yo le dije a el doctor "como así que usted va a mandar para la casa, ¿no ve que el niño estaba grave? ¿No ve que el niño está asfixiada?". Y no, no. Ahí también me regañan porque no se puede hacer nada, no era su trabajo. Y por eso me quedé callada. Desde 3 días, lo mismo lo traía ella al hospital. Y en hospital, dice que "no, que el niño no está asfixiado, llévelo para la casa". Y la mamá también vuelve a traer pa' la casa. Y allá... cuatro veces lo llevó al hospital. Y lo llevó a Pereira. Y de Pereira lo llevó a Armenia. Armenia lo transmitió allá y en la mitad murió el niño. El niño que estaba grave. Y por eso ahí hicieron un paro. También allá en el hospital.

[INVESTIGADORA] ¿eso fue hace..?

[C-I04]: El año pasado, trabajé dos meses y ya se acabó mi contrato.

8:36 ¶ 39 – 42, in Salud

3:41 ¶ 410 – 412, in AFRO_TB.docx

[A01]: ah no, y le digo que las enfermeras también lo hacen...

[A07]: y ellas lo hacen porque como ven al médico hacerlo y como la comunidad no le dice nada al médico, ella se va adoptando esas malas costumbres.

[A01]: cuando yo trabaje en el centro de recuperación nutricional, nosotros tenemos un médico, pero contábamos con la doctora, que ella vive en la cabecera municipal, a veces el niño o algo le decíamos a la doctora, vea ella decía vamos a hacer esto y esto y vamos a mirar la evolución, vamos vigilar el niño o hay niños, los niños con desnutrición sabemos que es una enfermedad mortal, que tiene atención... una urgencia prioritaria, en cualquier momento se descompensa y llega uno a la seis y media con un niño y que hay... no tuvo todo el día para traerlo, por qué lo trae a esta hora. Entonces uno tiene que saber a qué horas el niño se va a enfermar para llevarlo... eso es lo que le dice la enfermera... ¡ay! Por qué lo trajo... o uno a veces va a las nueve de la noche: "a esta hora no hay médico, por qué no lo trajeron temprano que están los médicos, ahora qué quieren qué haga a esta hora, yo le tomo los signos y le digo al médico a ver él qué dice".

8:39 ¶ 131 – 132, in Salud

4:249 ¶ 1422 – 1425, in INSTITUCIONES ML & LC.docx

[I-I02]: cuando está enfermo dicen que tienen que pedir cita y dan para un mes, para dos meses así y mientras... se muere uno, el niño [inaudible]

8:54 ¶ 69 – 70, in Salud

4:3 ¶ 273, in INSTITUCIONES ML & LC.docx

[I-M01]: entonces se lo llevan, no... enfermera que el niño sigue enfermo, otra vez, devolvámoslo. Hagámosle el acompañamiento para ver si así nos lo atienden porque no sé si es que ellos no saben hablar, no saben explicar o no se les presta la atención que se debe, entonces se les hace el acompañamiento para que ese niño sea atendido y le manden pues la fórmula, explicarles a ellos muy bien cómo vigilar esa fórmula, porque a veces "ah bueno papito, el niño está ah bueno, papito, el niño tiene una infección gastrointestinal y esta es la fórmula", quizá no se le explica cómo se le debe dar el medicamento y se lo llevan para la casa, no saben cómo darle el medicamento. O se lo explican y no se acuerdan, entonces eso es como desde esa parte. Entonces ellos se van como desmotivando.

8:63 ¶ 102 – 103, in Salud

4:118 ¶ 281, in INSTITUCIONES ML & LC.docx

[I-I07]: el niño que falleció, primero llevaron al puesto de salud y después, al otro día, llegó de Sallada [inaudible] tenía... eso es lo que quiero explicar, ellos mandan para la casa ¿por qué? Porque el niño se pone peor y cuando llega a Pereira y allá se muere. Eso es lo que pasa. Porque ese niño tenía malaria, desnutrición, tratamiento también, pero como le digo, mandan para la casa al otro día.

8:68 ¶ 178 – 179, in Salud

4:379 ¶ 1412, in INSTITUCIONES ML & LC.docx

[I-A03]: porque a mi me pasó que día que una recaída y yo llegué que no era capaz ni de dar el paso y me dice un auxiliar ah es que ya fichos no hay y no la pueden atender y yo que pena con usted pero el problema que... a mí, si yo voy así me deben atender porque mi caso porque mi caso no es cualquier cosa y si aquí no hay nadie... o sea, yo sé que aquí, de pronto los primeros auxilios y váyase, ya... pero es que no, que yo no la puedo antender porque ya no hay ficho y el médico está muy ocupado, entonces yo le dije bueno, listo, me acerqué al médico, me dijeron, le comenté mi caso particular y le dije mire que me pasa esto y esto y dijo suspendan lo que estén haciendo y me la atienden, el mismo y a los 3 días me tuvieron que hospitalizar. Entonces el problema no es que yo no la puedo atender, el caso es que hay que priorizar. Hay que priorizar porque si una persona está en muy mal estado de salud y si, por ejemplo, si usted está más mal y yo estoy más estable, atiendan a usted y depsués me pasan a mi, pero hay casos que no dan espera, no dan espera. Entonces...

9:6 ¶ 21 – 22, in Corresponsabilidad

1:66 ¶ 535, in Instituciones_TB.docx

[I-M08] Yo he pensado siempre que una de las soluciones es no darles todo, como se lo da el gobierno. Yo pienso es como por los resguardos, por comunidades, darle como más agentes comunitarios, en comida darles... no darles el mercado, darles un ingeniero agrónomo... entonces en este resguardo o para tantas comunidades y empezar a hacer truque, que ellos sepan, que hay que cultivar para comer, no que todo es regalado, porque es que ahí donde va. Y a todos nos pasaría, yo creo que si a mí me dicen ven [I-M08], te doy el millón de pesos que te ganas y no haces nada, yo no hago nada, me quedo en la casa. Entonces pasa con ellos pienso yo

9:9 ¶ 27 – 29, in Corresponsabilidad

1:144 ¶ 271 – 272, in Instituciones_TB.docx

[I-M08] Pero yo no entiendo, entonces, por qué vienen después a decir que el hospital es el culpable de todo.

[I-A03] O sea, porque esa es como la percepción que ellos tienen...

9:11 ¶ 37 – 40, in Corresponsabilidad

1:156 ¶ 266 – 268, in Instituciones_TB.docx

[I-M01] Yo creo que de pronto también a veces son despreocupados, una falta de consciencia. Por ejemplo, hoy, vinieron a cobrar lo del incentivo que están, o sea, sí, estuve por ahí cerca y una niña tuvo sangrados nasales, ¿cierto? Y la mamá no la quería traer al hospital, "—no, es que yo tengo que cobrar. — no mamá, primero lleve la niña. — No, que no, que no y que no".

[I-M08] y usted no los puede obligar, qué más hace uno.

[I-M01] Pero se le dieron opciones [inaudible] la pasamos allá adelanta para que la atendieran de los primeras, pa que ella pudiera venirse, pero de no ser así, ella no viene. Entonces como falta también de...

9:16 ¶ 53 – 54, in Corresponsabilidad

1:186 ¶ 484, in Instituciones_TB.docx

[I-I04] ellos decían: si se van para el hospital allá los matan [Risas]. Eso decían, por es ellos... les daba a todos, pero quedaban reservados en la casa.

9:17 ¶ 55 – 56, in Corresponsabilidad

2:1 ¶ 119, in Indigena TB.docx

[C-I02]: A la vez la doctora no es de acá, es de otro departamento. Ella es cauquenia. Entonces no está bien... ...no está muy de mano con el resguardo Chamí Embera Unificado, nos está desconociendo, ella es muy cascarrabia. Cuando ella se está enojando, ella no quiere a los indígenas.

9:18 ¶ 57 – 58, in Corresponsabilidad

2:9 ¶ 143, in Indigena TB.docx

[C-I05]. O sea, otra importancia que hace anitos, hace añitos, médicos que tenían, o sea, confianza a los indígenas, que decían "— Quenverde, ¿dónde duele? — acaí, acaí". Entonces decía "a dónde", entonces el indígena tocas, donde duele ahí, toca el indígena ahí. Entonces, ellos ya el médico ya tenían esa confianza. ¡Poropira! ¡Poropira! ¡Dolor de cabeza! ¡Acaí! acá. El médico ya recetaba... la confianza. La confianza era inmensa, era confianza. Pero ahora no hay confianza.

9:19 ¶ 59 – 60, in Corresponsabilidad

2:19 ¶ 107, in Indigena TB.docx

[C-I02]: Ahí en el centro de salud llegan médicos. Médicos vienen a hacer rurales. Tiene para práctica. No conoce todo lo que es médico abarca de trabajo. Como es nuevo, está practicando, es rural. Pero el médico hay un fallo. Y hay un médico de buena gente. Lo quieren a los indígenas. Hay un médico que no quieren a los indígenas, lo rechazan. Hay dos cosas a jefe. Ahí nosotros vemos la debilidad del Centro de salud. Hay un médico que quieren mucho al Embera. Hay unos, no todos. Y hay unos... Tratan muy poco. No socializan. Más bien dice que esto está bien y esos niños han muerto en la casa. Una vez murió una... No quisiera contarle, pero recordemos. Mi nietica así murió en Santa Cecilia. Venía con asfixia, de la

casita venía con asfixia esa nochecita. Vino al médico y no lo atendieron. Dijeron que espere, que espere y en la puertica del Centro de salud murió la nietecita. Y no los demande [inaudible].

9:20 ¶ 61 – 62, in Corresponsabilidad

2:43 ¶ 111, in Indigena TB.docx

[C-I03]: Yo creo que el [C-I05] sabe, [C-I02] sabe... ...lo demás enfermeros saben. Un ejemplo, en buzón de sugerencias, Si uno ya escribe, un ejemplo, si yo escribe y pasa el buzón de sugerencia. Y eso como fue... ...y quién va a mirar o el... el gerente. Porque ya lleva cuatro veces que yo siempre... una cartica partida... haciendo... y hay dos, una negra de anterior... ...y hay una persona que contratado... ...ya es nuevo. Ellos siempre meten grosero. Uno cuando va ahí siempre meten grosero... ...por eso yo escribí para que...

9:21 ¶ 63 – 64, in Corresponsabilidad

2:58 ¶ 133, in Indigena TB.docx

[C-I03]: El médico maltrataba por la palabra. Entonces si mujer avispadita o sabe hablar, enfrentar a uno, uno cuando ya habla, ellos pueden entender normalmente. Pero si uno queda así... Un ejemplo, usted me habla muchas cosas y no entiende, Me queda así. Así como nosotros, usted cuando yo hablo con mi lengua, usted no va a entender, se queda así. Y nosotros también así mismo. Así mismo. Así cuando ya una mujer no entiende palabra en español, uno hablando, mal tratando por la palabra, uno que está hablando, uno piensa eso. Así me paso de en Santa Cecilia. Una mujer que vine y este... la enfermera, una negrita que trabaja allá, ella había hablado mal de mí a la señora y al paciente. Y yo escuchando ahí, al lado de ella, escuchando, al último me tocó hablar, porque hay que respetar a la señora. Ellos no entienden tu palabra, pero tienen que respetar, ella es paciente, usted es un enfermero, pero usted también tiene su jefe que manda. Entonces quedó callado y se fue y consultó a la médica. Y eso pasa.

9:22 ¶ 65 – 66, in Corresponsabilidad

2:60 ¶ 105, in Indigena TB.docx

[C-I01] uno cree que hoy en día de las mujeres, por qué razón no lo quieren traer al hospital. Yo creo que varios años atrás, siempre pasaba así. Este año ha pasado así. Porque hay muchas mujeres, porque [inaudible] no traen cuando un niño está en enfermo, porque no les gusta eso, porque hay veces los médicos solamente lo miran, que el niño está bien y lo mandan a la casa. Y por eso la mujer casi no le gusta, que no, que el médico... y lo mandan, ¿para qué lo van a llevar? Para eso tratan con Jaibaná y lo pasa con mejor trabajo [inaudible] familia. A los último, los enoja uno. Es que el médico no se mide y por qué van a ir más allá. Siempre hay una mujer que lo tratan así. Porque eso... es un mundo porque.... No hay buena atención.

9:25 ¶ 74 – 75, in Corresponsabilidad

2:149 ¶ 110, in Indigena TB.docx

[C-I02]: Es que en el hospital hay unos médicos muy groseros. Sí, muy grosero. Yo estaba la semana pasada, había estado allá, al hospital, que una señora llegó del Chocó... que una niña que tiene mucho brote en la cabeza. Y habló que no, que mantiene muy cochino, que no... Yo no quedo... Que yo le dije al señor ¿Y porque no tratan así a usted? Y como dicea ellas ¿Y usted por qué no [inaudible]? [inaudible] cómo así que van a decir que hay un [inaudible]. No puede tratar así, yo les dije a usted le faltan al respeto. Y la mamá, como ella no sabe hablar... de Choco hay una mujer, allá casi no se habla. Y también que no... que del Chocó son muy brutos que no saben hablar. Y no entienden nada. Ahí me cogió la raya, yo iba a hablar, pero yo les pego mi carpeta y ellos meten el buzón de sugerencias. Todo la parte... uno entiende nada o si no hace caso y a veces no quieren atender. Eso pasa ahí en el hospital.

9:28 ¶ 87 – 88, in Corresponsabilidad

3:64 ¶ 254, in AFRO_TB.docx

[A06]: yo que día llevé a mi mamá, en la noche y lo primero que me dijeron fue "y usted por qué no vino más temprano". ¿Y es que a ti una urgencia te dice voy? La sola urgencia, ah, no es que el médico ya se fue. Y que usted tiene la presión alta y qué por qué la tiene. Oiga niña, la tiene alta por el dolor, el [inaudible] y los signos vitales que se alteren. Es que “tome este” reporte y le dije "yo para qué me lo llevo, si yo no le voy a dar ningún medicamento, si la tiene alta, si la tiene en tanto por tanto, yo no le voy a dar ningún medicamento [inaudible]”. Hay otros días que usted va y ya le digo, usted amaneció como con dios o yo no sé, pero vienen y lo atienden ya sea aquí en Pueblo Rico, Pereira, en cualquier departamento. Esa [inaudible].

9:31 ¶ 102 – 106, in Corresponsabilidad

3:82 ¶ 406 – 409, in AFRO_TB.docx

[INVESTIGADORA] y tiene, porque ayer hablábamos de que un problema en el hospital era que venía gente afuera y había una relación tensa y con los tratos a veces, pero... o sea, qué porcentaje de la gente del hospital es de afuera...

[A01]: más que todos son los médicos...

[A07]: pero los médicos vienen pensando que somos una comunidad ignorante, una comunidad no educada y así nos tratan. No es el respeto con el que te trata un médico de la Clínica los Rosales o el Pinares Médico... no. "Pase a ver... usted no tiene nada, váyase para su casa".

[A02]: tiene que escoger el día y la hora en que se va a enfermar, porque si usted se enferma un domingo, llega con un dolor de cabeza, un malestar, "ah, no... eso es un guayabo"

9:32 ¶ 107 – 119, in Corresponsabilidad

3:133 ¶ 413 – 424, in AFRO_TB.docx

[A07]: Por esa actitud, yo perdí a un hijo. Por esa actitud, el 15 de octubre en el 2021 perdí a mi hijo. Porque desde el 8 consultamos porque ella tenía dolores de parto y ya estaba en los días y llegamos a la... bueno el médico dijo "se va a que es por cesaria" pero llegó el de Pueblo Rico y la devolvió "no, usted no tiene nada, para su casa" y el 12 volvimos y eran las 7 y media y me acuerdo de lo que dijo la enfermera, una persona que trabajó conmigo, una persona que a mí me [inaudible] y dice "siga está bien" ¿y qué hizo? Abrió la puerta malagana a las 7 y media de la noche: "yo no sé a qué vienen aquí si a esta hora no hay médico a trasnocharla a uno".

[A01]: lo primero que le dicen a uno: "¿por qué no vinieron temprano cuando estaba en el médico?", es el saludo que le dan, por qué no vinieron temprano cuando estaba en el médico.

[A07]: llamó al médico de malagana y le dijo la información como no era. Que ella tenía cólico y que tenía estreñimiento. El médico dijo "devuélvala para su casa". No dijo que eran espasmos abdominales que eso también es síntoma de parto, no, ni que estaba dilatando, no, ella no dijo eso. Yo no estaba aquí, yo estaba en una capacitación y cuando yo llego me cuenta todo eso y yo me enojé.

[A02]: y ella cómo sabía que era estreñimiento si le hizo una palpación...? Solamente la miró y dijo: ¿es esto?

[A01]: es que se supone que si una mujer ya llega en los días de trabajo de parto, hay que hacer, hay que reportarle al médico y hay que hacer el tacto. Hay que hacerle el tacto, reportarlo y remitir.

[A07]: lo primero que dicen es "¿A qué EPS es?" "— Medimás — ah, no, a Medimás no le gustan esos casos" y la devuelven. "no, no, no, no se puede". Sí, así es. Pero cuando llega un docente que dice ahí COSMITEC, inmediatamente el CHIP cambia. [Inaudible] Estos son los que facturan y dan la plata. O otro más perverso, accidente de tránsito, no saben dónde ponerlo... Pero cuando es subsidiado, Medimás que está en la quiebra, que le debe miles de millones... ¿cuál fue el resultado? en la mañana del 15 ya dijo "yo no aguanto más", llegamos con 6 de dilatación, la subieron a la ambulancia, no medicada, sabiendo que ya tenía 6 [inaudible] era de este color el líquido amniótico. Cuarto grado de meconio, mi hijo murió ahogado. Comió meconio y lo respiró. Entonces mire la secuencia: si hay un programa de salud, si hay unas rutas que atender, en lo que sea en lo que nos estemos imaginando, en lo que estamos haciendo hoy y desde la institucionalidad no se le presta la debida atención ni se sigue protocolo diseñado para eso, nunca va a haber un resultado positivo. Nunca. ¿A qué conlleva eso? A que cuando le hicimos ese... para cambiar a la gerente del hospital no había una sola prueba de que ella estuviera haciendo mal su trabajo ¿por qué? porque ni los afros ni los indígenas demandamos. No demandamos.

[A01]: sí, eso dijeron que no había demanda, ni nada...

[A07]: no hubo una prueba, no tuvimos una prueba para sacarla

[A01]: qué pasa a las cosas y nadie demanda.

[X] se hace búlla y ya. Ahí muere.

[A01]: un día de huelga y ya

[A07]: entonces uno como líder... hoy el hospital ya tiene cinco demandas incluyendo la mía y como a mí no me interesa los 1000, 2000 o 50 pesos que lleguen por eso. Es el antecedente. Es el antecedente, con ese antecedente, el nuevo gerente que llegue, porque este ya se va en marzo del próximo año ah, él sí se va a poner la atención y va a decir que no quiero que me sancionen, yo no quiero que mi trabajo aquí se entorpezca porque todos los recursos que llegan son para pagar demandas, ahora sí voy a mirar por eso.

9:33 ¶ 120 – 122, in Corresponsabilidad

3:149 ¶ 268 – 269, in AFRO_TB.docx

[A06]: allá hacen casa y cocina, más si llegan urgencias eso terminan de excusarlos, y pueden haber dos o tres médicos y se demoran para darle una acetaminofén. Por eso es que uno se automedica y da hasta pereza ir allá.

[A02]: a mí sí me da mucha pereza ir la verdad.

9:34 ¶ 123 – 124, in Corresponsabilidad

4:10 ¶ 1452, in INSTITUCIONES ML & LC.docx

[I-I07]: pues la población indígena digamos que no interesa digamos los íntomas, pero como te dije ahorita, recurso, distancia, [inaudible] y a veces no apoya los padres de familia, a veces como los papás,cierto? A veces pasa eso [inaudible] sí pesa mucho que... y también las demás [inaudible] es por eso... como... explicar... no dan ganas de ir al hospital por esos motivos, a veces y también da pereza de caminar a pie sin plata, sin pasajes, eso pasa. Pero cuando a veces, A VECES, uno compra un medicamento, algnos son juiciosos, llevan tratamiento. Pero cuando yo veo que no me importa nada salud o compra medicamento, si no le dan al niño, pero eso cuando compran los medicamentos sí le dan sus medicamentos o hacen tratamiento con medicina así, ¿sí me entiende? Es así de sencillo

9:35 ¶ 125 – 127, in Corresponsabilidad

4:18 ¶ 1493 – 1501, in INSTITUCIONES ML & LC.docx

"tiene que llevar al niño enfermo" y no lleva de pornto es que no les creen. ¿Qué creen ustedes? ¿eso puede pasar o no?

[I-M01]: si, se ha visto muchos casos, claro.

9:36 ¶ 131 – 132, in Corresponsabilidad

4:20 ¶ 233, in INSTITUCIONES ML & LC.docx

[I-I07]: Bueno, ese niño de Dokabú era del programa, y él pertenecía a esa comunidad, pero en esa parte, pues el médico y el demás equipo de trabajo, siempre dan una recomendación a los padres del hijo: que lleve al hospital después de tratar con el jaibaná. Si jaibaná no puede, si con el tratamiento ya no mejora la salud del niño, inmediatamente hay que llevarlo médico, al puesto de salud, para que el médico valore cómo está el niño y qué medicamento le den, pero a veces lo indignan, no hacen caso. Simplemente dicen no hubo plata, no tengo pasaje, eso es lo que pasa. Y ahí el niño va empeorando la salud, eso pasa acá, en Dokabú, siempre damos la recomendación con [inaudible]: lleve al puesto de salud y decía que no, que falta el tratamiento con Jaibaná y después mira el encuentro con la nutricionista y la psicosocial y observa que ese niño [inaudible] era muy tarde y nos toca hacer un compromiso con el papá y como el psicosocial hizo como algo...

9:38 ¶ 135 – 136, in Corresponsabilidad

4:59 ¶ 1450, in INSTITUCIONES ML & LC.docx

[I-I04]: por la distancia también, como ahorita...

9:39 ¶ 137 – 138, in Corresponsabilidad

4:80 ¶ 1458, in INSTITUCIONES ML & LC.docx

[I-M01]: ellos digo yo que no les gusta como mucho desplazarse a muchos lugares, entonces qué dicen ellos: el niño está mal, lo traen al hospital y obviamente lo tienen que remitir, entonces muchas veces las mamás, como es la mamá la que tiene que ir con el niño, ella puede que le preocupen los demás hijos o hasta el mismo esposo, dejarlo solo en la casa, entonces ellos como que priorizan la demás familia y no piensan como en las consecuencias de si yo dejo el niño en la comunidad se me puede hasta morir, ellos no...

9:40 ¶ 139 – 140, in Corresponsabilidad

4:81 ¶ 1410, in INSTITUCIONES ML & LC.docx

[I-I02]: el niño tiene malaria y el médico no quiso atender el niño, entonces yo lo iba a traer y él no quiso atender y yo hablé por urgencias al otro médico que era más formalito y él sí atendió, ese médico no quiso mandar a ese niño con malaria. Entonces él mismo atendió y mando otro medicamento para la casa y dijo que pidiera cita, el otro y a veces uno [inaudible] entonces eso pasa algunos.

9:41 ¶ 141 – 142, in Corresponsabilidad

4:94 ¶ 1461, in INSTITUCIONES ML & LC.docx

[I-I07]: a veces es miedo de ellos y ya ellos... dicen que no hay recurso para comprar pañales, difícilmente, por ese motivo, eso que ellos... esa parte y uno como...

9:42 ¶ 143 – 144, in Corresponsabilidad

4:131 ¶ 237, in INSTITUCIONES ML & LC.docx

Lo otro es que las autoridades deben asumir el rol de autoridad. No es simplemente yo soy cabildo, no, ellos tienen su junta directiva, tienen justicia, tienen gobernadores locales, tiene gobernadores mayores. Pero yo he evidenciado de que ellos ponen de cabildo a cualquiera. Me explico. Un ejemplo, yo soy el que más habla en las asambleas, entonces yo soy cabildo, simplemente porque yo soy el que más hablo, yo no tengo ni idea de pa' qué sirve ser cabildo o cuales son las obligaciones de ser cabildo. Porque ser cabildo no es ser cabildo, eso tiene una responsabilidad legal, porque él es el representante legal de ese territorio. Entonces mientras ellos no asuman ese rol, de que yo, por ejemplo, [I-I04] es cabildo, yo le digo "Cabildo vea, ese niño está enfermo y la mamá no lo quiere sacar" "ah, es que la mamá no quiere". No, yo soy autoridad. "Justicia, haga favor, me retira al niño y me lleva", pero no ejercen esa autoridad. Entonces, si no ejercen esa autoridad, el papá está libre de hacer para lo que a él le convenga y hay muchas excusas. Muchas excusas. No hay recurso, pero, resulta que muchas veces usted le dicen "bájelo, yo le doy los pasajes", "no pero hoy no puedo, mañana". Entonces ya lo llevan mañana cuando el niño ya está complicado y no pasa nada. Y se murió el niño y no hay una investigación a no ser que sea una entidad que pida un informe, no hay... o sea... nada, a no ser que sea miembro de algún programa que esté vinculado al informe, pero en últimas, no hay una responsabilidad sobre nadie. Aun conociendo de que hay una sentencia, pero no hay responsabilidad, si usted llama las autoridades que vayan a las mesas, no van, están ocupados, no delegan. Entonces hay responsabilidad de las instituciones, hay responsabilidad de los cabildos

9:43 ¶ 145 – 147, in Corresponsabilidad

4:140 ¶ 1462 – 1463, in INSTITUCIONES ML & LC.docx

¿Qué... por qué lado puede irse más la cosa?

[I-M01]: ellos tal vez creen que no les va a pasar.

9:44 ¶ 148 – 149, in Corresponsabilidad

4:141 ¶ 237, in INSTITUCIONES ML & LC.docx

No sé cuál es la percepción que tienen dentro de los resguardos con respeto a la muerte de los niños, con respecto a la muerte de las personas en el territorio, porque ellos conocen las consecuencias, porque no es que ellos no las conozcan, ellos conocen las consecuencias de que si el niño está malito, tienen que sacarlo o si no se les va a morir, y ellos saben que si el niño está enfermo se les va a morir. Pero entonces, es más fácil culpar... Es la percepción personal, ¿cierto? Entonces duró 7 días en jaibaná, lo trajeron, el niño está muy grave, tiene que llevarlo a Pereira, pero la percepción de que se lo lleven apara Pereira es que allá los dejan morir. Pero entonces, yo como familia, yo como padre de familia, ¿por qué dejé llegar al niño a ese estado? No, la percepción es: lo llevo a Pereira y allá se muere o muchas veces no lo llevan, porque ellos no tienen la percepción de la enfermedad del niño, sino de ellos, como padres, y a mi allá quién los pasajes... y a mi allá... Y a mí allá... y a mí allá... y a mí allá... exacto no visualizan la enfermedad o el estado

de salud del niño. Entonces eso ha sido una barrera grandísima, por más que uno les dice que en Pereira hay hogar de paso, que usted les dice las cosas. Entonces, muchas veces, estando en el puesto de salud, porque cuando yo era coordinador, les decía los vamos a llevar a Pueblo Rico y ¡Pum! Se volaban... y si los vamos a llevar a Pueblo Rico es porque el niño está maluco, no lo van a llevar a pasear, pero ellos se vuelan. Entonces es como que priorizan primero sus necesidades de padres, a el estado de salud del niño. Y eso pasa con todo. Si usted le dice, el niño tiene malaria,hasta que el niño no está titilando de fiebre y del IRA y que está que... pero ellos ya conocen los signos de malaria. Entonces eso es algo que... algo que tenemos que ir... sacando de la cabeza a ellos de que sepan de que... de que tiene que haber una consecuencia. De que si yo, papá, no lo saco debo tener una responsabilidad. No estoy diciendo que lo metan preso y la justicia ordinaria... aunque estamos violentando los derechos del niño, que son derechos fundamentales y están por encima de autoridades y están por encima de todo. Pero no sé si el estado es... le es mucho más fácil hacerse el... ¿cierto? Exacto y decir "no, ellos tienen justicia propia, son autoridades, ellos allá manejan..." pero entonces dónde están los derechos fundamentales de los niños que están por encima de todo. Y mientras ello no ocurra y mientras ellos sepan de que el cabildo no es simplemente mandar, y que del cabildo no es simplemente decir vea trabaje usted por puestos... mientras no haya una responsabilidad en ellos, no va a pasar nada y eso no va a cambiar. A parte de que hay un brote de diarrea impresionante y hay mucha malnutrición por la diarrea.

9:46 ¶ 152 – 154, in Corresponsabilidad

4:148 ¶ 271 – 272, in INSTITUCIONES ML & LC.docx

Los niños se enferman, los papás llevan los niños al hospital, llegan al hospital... Ah, en el momento que el niño llega, primero prueba de malaria, porque ahorita es eso, primero prueba de malaria, lo primero así el niño lleve diarrea, bueno, otros síntomas, lo primero es prueba de malaria porque se han visto casos, ¿cierto? El papá, ahí está motivado en llevar ese niño... Porque ellos no son de que se enfermó el niño y corramos al hospital, pues no. Ellos no. Si ellos llevan al niño al hospital es porque nosotros, como operador, le decimos, cuando el niño presente estos síntomas, llévelo al hospital. Entonces, para ellos es desgastante quizás que ellos, bueno, motivados por nosotros, lleven el niño al hospital y que en el hospital, cierto, se le atiende al niño una urgencia siempre y cuando los signos de los niños estén alterados. Si de los signos está bien, para la casa. Pida una cita y para la casa, ¿cierto?. Entonces ellos se desmotivan. Si yo llevo a mi niño al hospital es porque está enfermo, pero sí me lo regresan para la casa, entonces, se van a sentir aprerezados de llevar los hijos al hospital, entonces casos se han visto. Entonces le dicen al papá "papito hay que pedirle una cita", ¿para cuántos días? Las citas están super demoradas.

[I-I02]: un mes, dos.

9:47 ¶ 155 – 162, in Corresponsabilidad

4:228 ¶ 1398 – 1404, in INSTITUCIONES ML & LC.docx

tiene que llevar el niño al médico, el niño está mal, usted está mal y no lo hacían, como si no quisieran llevarlo, ¿por qué creenq ue pasa eso?

[I-A03]: falta de voluntad pienso yo

[INVESTIGADORA] falta de voluntad, ¿qué más?

[I-I02]: falta de recurso

[I-I07]: y a veces los papás dicen que si llevo al hospital, no atienden mi hijo urgente, tengo que esperar una hora, dos horas, eso es lo que pasa casi la mayoría. Dos cosas: recursos y la demora y no atienden correctamente.

[I-I04]: ellos dicen si van a al hospital los medicamentos, primero que van a dar es acetaminofén, ellos dicen.

[I-I07]: y la acetaminofén la compra en la farmacia, es lo que dicen también a veces.

9:54 ¶ 177 – 178, in Corresponsabilidad

4:350 ¶ 1457, in INSTITUCIONES ML & LC.docx

[I-I07]: falta de interés, cuando uno va, se puede ver la falta de control y las vacunas, ¿no? Siempre me dicen no tengo plata con qué, entonces yo les digo, yo los llevo en la moto, yo colaboro y ya... tiene la posibilidad, entonces también por el interés [inaudible] uno colabora, se colabora, ahí llevo en la moto conmigo, pero también [inaudible] llecarlo solo a ellos o sola... y tampoco no caminan, algunos sí caminan, pero alguno sno, todos no, les da pereza, no sé y a veces dicen ah que mi marido está cuidando a mi hijo que yo por qué...[inaudible] que mi esposo no está haciendo nada, que no está apoyando al niño que está enfermo, que le está dando plata y las mamitas quedan ahí, paradas, así es como lo que yo... en el programa, eso comentaban

9:59 ¶ 192 – 194, in Corresponsabilidad

6:32 ¶ 689 – 690, in Anexo 2 Transcripcion grupo focal de malaria y leishmaniasis con comunidad afrodescendiente.docx

ustedes creen que también hayan como problemas tal vez con el conocimiento de la gente como en identificar esos síntomas y saber cuando hay que ir o sí van y siempre es que no los atenden. Bueno, ya hemos dicho que pereza, que a vedes les da pereza.

[C-A01]: la verdad es que le dicen a uno que consulte al otro día. A mí me pasó. Yo tengo una niña de 7 años, desafortunadamente ella me nació enfermita, ella me nació con un asma, todavía la tiene. Una vez, a ella le dan las crisis de la nada, igual es un asma no especificada, los médicos no saben qué... y una vez le dio una crisis así y ella cuando le dan las crisis ella como que se queda así como dormida y bastante agitadita, cuando eso estaba el doctor pediatra y yo la lleve al hospital así tapadita en la tarde, y le dije doc, ya era como las 5, y yo le dije "yo entiendo — por eso le digo que hay mucha falta de persona también, que ya un médico

cansado, haber atendido 40, 50, personas en el día también es mucho cansancio, pero si llega alguien suplicándole a uno, quizá uno también debe ponerse la mano en el corazón, yo le dije— Mire, la niña está muy mal" y me dijo "no, tráigala mañana, que ya no estoy haciendo más consulta y todo" y pero mire y no me escuchó, me tocó pedirle el favor a la hermana, a el hermano de la señor que me llevara a Pueblo Rico, la niña de una vez para Pereira y en la UCI 19 días, entonces mire, si b yo no hago la gestión la niña se me mure y fui al hospital...

9:60 ¶ 195 – 206, in Corresponsabilidad

6:108 ¶ 648 – 658, in Anexo 2 Transcripcion grupo focal de malaria y leishmaniasis con comunidad afrodescendiente.docx

[C-A02]: yo sentía que me hacía así, así, así, fue tanto que yo empecé a hiperventilar, cuando me dijeron acuéstela ahí en la camilla, dijeron, acuéstela ahí en la camilla, fueron y le tocaron a la doctora, ah no, yo estoy ocupada, cuando por ahí llegó, atendió a un paciente que llegó por una consulta, para que le leyera unos exámenes, eh, lo atendió y ahí sí salió "qué es lo que tiene", entonces yo no podía hablar, entonces mi hermana, mi mamá y mi prima que estaban ahí, dijeron que no, es que tiene un dolor de cabeza muy fuerte, no es capaz de hablar, vea que está hiperventilando y "ah, ya va" y se fue. Ya por allá llegó una enfermera a aplicarme una inyección, ni siquiera fueron capaz de decir "ah, no, ella tiene esto, voy a aplicar esto para que le baje el dolor", nada, nada, entonces "doctor, qué es lo que le van a aplicar", "ah, usted quiere que se mejore o quiere seguir así?". Entonces mi prima le dijo, yo quiero saber qué le van a aplicar [inaudible] me dijo disque, usted está con dolor de cabeza o se está haciendo.

[INVESTIGADORA] o sea, la actitud del personal de salud

[C-A06]: no y la fama que [inaudible] con un dolor de cabeza horrible... eh.. le dieron la cita y el médico le mando la farmacia y la muchacha le dio el que no era y se lo tomó y la noche le estaba dando...

[C-A01]: se iba a infartar y era un medicamento muy básico, pero a ella le caía mal, la señora se complicó

[C-A06]: y lo que le dijo ella, no, me equivoqué...

[inaudible]

[C-A01]: pero fue la semana pasada, mi hermana fue antier al hospital y dijo que le habían despachado unos medicamentos...

[C-A06]. y le dijo que ay no que se equivocó, que... tan metidas.

[C-A01]: no, cualquiera se equivoca, sí, pero son cosas de mucho cuidado, porque uno puede...

[INVESTIGADORA] ¿es común ese tipo de equivocaciones?

[C-A02]: a mi una vez, le dije que yo era alérgica al ibuprofeno y me formuló ibuprofeno, yo cuando la muchacha fue yo le dije por qué y no es que aquí en la

fórmula, cuando yo le dije al médico que era alérgica, cuando regresé donde él le dije, Doc, me está formulando ibuprofeno y yo soy alérgica al ibuprofeno, y qué te da el ibuprofeno, me da taquicardia, ah eso no es una alergia... entonces imagínese pues... eso es negligencia, tanto enfermeras... todo el personal

9:62 ¶ 213 – 215, in Corresponsabilidad

6:212 ¶ 931 – 932, in Anexo 2 Transcripcion grupo focal de malaria y leishmaniasis con comunidad afrodescendiente.docx

[INVESTIGADORA] porque nosotros escuchamos, pues nos han contado que varios niños se han muerto [inaudible] [C-A01]: ¿señora?

[INVESTIGADORA] que últimamente nos han llegado historias de niños indígenas que han muerto [C-A09]: sí, pero cuando los sacan, ellos ya vienen de una manera ya deshidratados, de todo. Entonces a las mamás indígenas hay que educarlas mucho sobre eso, que si los mismos enfermeros ¡Sáquenlos! No dejarlos tanto tiempo.

9:63 ¶ 216 – 222, in Corresponsabilidad

6:213 ¶ 642 – 647, in Anexo 2 Transcripcion grupo focal de malaria y leishmaniasis con comunidad afrodescendiente.docx

[C-A02]: creería que una de los factores que la gente no va y no va al hospital es la negligencia, la negligencia... aquí se ve mucha negligencia con el trato, al servicio en el hospital eso es pero re malo y eso es lo que más está afectando.

[INVESTIGADORA] ¿pero negligencia quieres decir que no los atienden?

[C-A08]: no, en algunos casos sí los atienden, la cosa es que sí los atienden, pero, cómo le dijera yo, a usted lo atienden y usted va por una cosa lo mira el médico, le manda medicamento y nunca le manda pues un examen que es lo que gente anda buscando y lo mandan para la casa, la persona se toma el medicamento y han habido casos que la persona la mandan para la casa, pasan dos tres días y fallecen

[C-A01]: se complican

[C-A08]: y en otros casos les dan la cita, si la persona va por una cita hoy, se la dan para dos, tres, cuatro meses, la persona, ¿qué va a hacer en todo ese tiempo, entonces es más que todo por eso?

[C-A07]: por eso uno busca particular, es más ligero

10:19 ¶ 70 – 75, in Estigma

4:85 ¶ 561 – 566, in INSTITUCIONES ML & LC.docx

[I-I02]: se burlan, le da pena cuando tiene cicatriz, no ese está tiene cicatriz, hay uno indígena, a veces sienten mal, pero uno qué va a hacer si le dio ese coso.

[INVESTIGADORA] sobretodo en la cara

[I-I02]: pues está bien que les de ahí en el cuerpo, así no... no es nada.

[INVESTIGADORA] ¿y tú qué decías de eso? ¿Que te daba pena..? [I-M01]: de pronto sí, en el rostro, pero de pronto no... ellos no es que los agobia tanto en esa parte. De pronto al indígena no tanto.

[I-M06]: de pronto a los retornados sí.

10:22 ¶ 80 – 83, in Estigma

6:172 ¶ 445 – 447, in Anexo 2 Transcripcion grupo focal de malaria y leishmaniasis con comunidad afrodescendiente.docx

[INVESTIGADORA] pero el tener las úlceras, ¿hay gente que le da pena salir a la calle con...? [C-A01]: hay gente que sí, que eso le genera como un poquito de inconveniente

[inaudible]

[C-A02]: yo creo que... somos muy vanidosos.

10:23 ¶ 84 – 86, in Estigma

6:208 ¶ 928 – 929, in Anexo 2 Transcripcion grupo focal de malaria y leishmaniasis con comunidad afrodescendiente.docx

C-A01]: yo también , 100%. Con la misma gente de uno es muy pesado, ¿sí o no [C-A08]?

[C-A08]: porque lo meten en chisme a uno.

13:38 ¶ 130 – 135, in Percepción de riesgo

4:29 ¶ 854 – 858, in INSTITUCIONES ML & LC.docx

[INVESTIGADORA]: también en Bajo San Juan hay harta leishmanisis

[I-A03]: no...

[I-M06]: sí hay, pero no...

[I-A03]: no es tan relevante

[I-M06]: lo que pasa es que como ahorita a está la vía, pues... exacto, pero no...

13:54 ¶ 201 – 202, in Percepción de riesgo

4:282 ¶ 1473, in INSTITUCIONES ML & LC.docx

[I-M01]: ellos no piensan en eso y tal vez confían de que el niño se va a aliviar o... por ejemplo el caso que yo tenía, porque gracias a dios ya se solucionó y me preocupaba un montón, ella... veníamos luchando mucho con la niña y con ellos, pero ellos no, porque no tenían con quién dejarlo, un niño más pequeño, porque la niña tenía que estar en el centro de recuperación de santa cecilia y entonces ella decía que no que ella no se podía ir con la niña, que porque ella tenía una niña más

pequeña, quién la iba a cuidar, que la niña no podía estar sola, entonces por eso ella se negaba a desplazarse con la niña hasta que ya la niña se complicó tanto que ellos vieron la necesidad de que decidieron buscar ayuda.

14:24 ¶ 72 – 73, in Propuestas

5:95 ¶ 561, in Anexo 1 Transcripcion grupo focal de malaria y leishmaniasis con comunidad indígena.docx

[C-I03]: en caso de la comunidad de nosotros, las mujeres... como le digo... cuando encuentra un taller como hay veces, como los hombres, no más que discriminan nosotras, un ejemplo, si yo estoy recibiendo aquí talleres, si yo voy a la comunidad y por la tarde voy llamando para yo avisarle qué entendí y le estoy enseñando y eso como casi no quiere arrimar, uno desanima y no quiere entender "que ah, que esa mujer qué va a hacer, solamente sale eso". Entonces uno ahí cómo queda, uno quiero llevar una información aquí a la comunidad y ahí queda desanimado, uno queda pensativo, eso no más hace falta.

○ Determinantes sociales_LC

37 Citas:

5:1 ¶ 7 – 8, in Factores económicos

1:2 ¶ 257, in Instituciones_TB.docx

[I-I02] unos dicen, si el niño está enfermo, llévenlo al hospital y dicen no... no hay platica con qué llevarlo al niño al hospital, puedo conseguirlo mañana o pasado mañana [inaudible] las mamás

5:2 ¶ 17 – 18, in Factores económicos

5:125 ¶ 725, in Anexo 1 Transcripcion grupo focal de malaria y leishmaniasis con comunidad indígena.docx

[C-I04]: algunas mamás, algunas mamitas para llevar al hospital, les falta el recurso, porque... un ejemplo, si un enfermara mi hija y no hay recurso, ¿yo con qué voy a llevarla al hospital? No hay recurso. Queda difícil

5:5 ¶ 11 – 12, in Factores económicos

1:108 ¶ 256, in Instituciones_TB.docx

[I-M01] yo pienso que a veces no tienen la facilidad económicamente, o a veces les falta es como la voluntad, porque a veces uno le pone hasta los medios ahí, para que bajen el niño y no, no lo hacen.

7:2 ¶ 9 – 10, in Prácticas culturales

1:30 ¶ 546, in Instituciones_TB.docx

[I-M08] pero si el papá no da la orden, no lo llevan.

7:3 ¶ 14 – 15, in Prácticas culturales

1:46 ¶ 548, in Instituciones_TB.docx

[I-M08] el que manda es el hombre, si él no está ahí, ahí no sé cómo hace, pero la mamá no... que porque no puede disponer, que porque el esposo no está y él es el que manda.

7:5 ¶ 16 – 17, in Prácticas culturales

2:16 ¶ 461, in Indigena TB.docx

[C-I05]: el médico pensará que nosotros no nos alimentamos bien por lo que él tiene alimentación occidental y es diferente a la alimentación de nosotros, por eso debe... pero nosotros comemos alimentación que nos da alimento, por ejemplo las enfermedades que previene, por ejemplo, el que come bastante yuyo, nunca va a tener dificultad de piel la sangre, algo así, no tiene contaminación, en cambio en la occidental, comemos, pero nos contaminamos.

7:6 ¶ 18 – 20, in Prácticas culturales

2:30 ¶ 373 – 374, in Indigena TB.docx

[C-I02] el primitivito

[INVESTIGADORA] ¿el primitivito y qué más? [C-I02] bananito, yuquita... mucho alimento, la yuca y el maíz...

7:8 ¶ 23 – 24, in Prácticas culturales

2:45 ¶ 468, in Indigena TB.docx

[C-I03]: y no comemos tanta carne, para qué voy a decir mentiras si yo no como carne y arroz [inaudible] porque somos culturas diferentes, yo cuando tengo carne, a veces arroz, a veces con primitivo, porque el primitivo nos hace falta. Siempre lo tenemos de la finca, hacemos jugo, fritamos y ya, con eso...

7:9 ¶ 25 – 26, in Prácticas culturales

2:48 ¶ 467, in Indigena TB.docx

[INVESTIGADORA] eso es por la mañana. [C-I04]: por la mañana tomamos esa harina, así como usted toma la aguapanela, siempre tomamos eso, harina con bequé.

7:11 ¶ 30 – 31, in Prácticas culturales

2:93 ¶ 466, in Indigena TB.docx

[INVESTIGADORA] primitivo, yuyo, ¿cómo cocinan el yuyo? [C-I03]: a través del monte es como una... vino, se llama, vino. Otro es el pringamoso. Y eso los

cocinamos, ya, eso no echamos nada, eso lo cocinamos y esa es sal, y con harina y ya.

7:12 ¶ 32 – 33, in Prácticas culturales

2:104 ¶ 463, in Indigena TB.docx

[C-I03]: pues yo he sentido cuando fui a hacer un control del niño, me preguntaba el médico si el niño alimentaba en la mañana huevo con arepa, que si toma chocolate, y yo todo escuché, escuché y a lo último yo contesté al médico, médico sabe qué, usted no sabe que indígena alimentamos diferente, culturalmente tenemos diferente comida típica. Un ejemplo, nosotros como embera, en la mañana desyunamos harina con maíz culturalmente que nosotros hicimos, con una hierba que se llama Bequé, a nosotras, ese es como nuestro chocolate con leche, harina con Bequé, así mismo alimentamos. No alimentamos leche por la mañana, no, tenemos diferente, pero alguno médicos piensan que nosostros desnutrimos los niños por no alimento de occidental. No, es que nosostros tenemos otro alimento que es natural.

7:13 ¶ 34 – 36, in Prácticas culturales

2:119 ¶ 464 – 465, in Indigena TB.docx

[INVESTIGADORA] bueno, entonces en el día, ese es el desayuno, la harina de maíz con bequé, ¿qué más?

[C-I03] y yuyo. Sí, ese es nuestro desayuno y a la nevera también primitivo.

7:14 ¶ 37 – 38, in Prácticas culturales

2:122 ¶ 475, in Indigena TB.docx

[C-I05]: lo que pasa la modernización nos ha cambiado, anteriormente la abuelita nos daba pringamosa con salecita y con primitivito y de encima de sobremesa nos daba harina, y quedaba normal, esa era como que la comida y conserbávamos mucho porque algunos muchachos, por decir algo, me confunden con [inaudible], yo tengo 80 años y yo en mi cabeza [inaudible] por encima, eso trae es de comida, en cambio ahora, yo tengo un sobrinito, sobrinito mío, hombre, por dios se llenaron de... es un niñito que crecieron, eso se traía... de resistencia y y lernarnos igualito, de pronto occidental cree que como yo como pollo me quedé bien, pero comiendo comida típica, queda igual lleno la resistencia la sangra, eso ha pasado.

7:18 ¶ 50 – 55, in Prácticas culturales

2:145 ¶ 368 – 372, in Indigena TB.docx

[INVESTIGADORA] ¿qué más? La buena alimentación... ¿el indígena se alimenta bien, regular o mal?

[C-I05]: el indígena se alimenta bien. Ahora ya está alimentando bien.

[C-I03]: estamos alimentando pura natural.

[C-I01] comida natural

[C-I06] y típica.

7:25 ¶ 81 – 82, in Prácticas culturales

4:113 ¶ 254, in INSTITUCIONES ML & LC.docx

Y que en todo lo que tiene que ver con el hogar, el papá no participa para nada. Para nada. Para nada es para nada; ni en la crianza de los hijos, ni en ir a buscar leña porque eso es trabajo de mujer, ellos lo simplemente llegan, se levanta, trabaja en el campo, llevan los alimentos y ya. Las tardes son para jugar futbol, ¿sí o no? Para reunirse entre compañeros hombre, para reunirse entre hombres y hablar, y hablar y hablar y socializar, esa es la vida del hombre en la humanidad y de ir a proteger el hogar, esa es la razón de ser del hombre, la protección del hogar trabajar la tierra y llevar los alimentos hasta ahí llega el hombre, lo demás, porque ni siquiera la cultura, si ustedes miran las que se pintan quiénes son? La mujer, las que danzan a quiénes son? Las mujeres. ¿las que llevan la crianza de los hijos? Las mujeres. ¿La que llevan la tradición, la tradición oral, toda la tradición? La mujer. Todo, porque todo, todo todo gira al rededor de la mujer. Todo, todo, todo, el hombre no está sino para procrear, proteger el hogar y llevar la alimentación. Pare de contar. No más. Entonces, ella no tiene tiempo de nada más, y antes son muy verracas que tienen tiempo de buscar leña y tienen tiempo de... Y por eso se ve... exacto y por eso es que usted ve que nosotros tratamos de abordar el tema de que, las niñas son niñas y tienen que estar jugando, pero es que ellas no tienen tiempo, y la responsabilidad de la niña que va creciendo, es cuidar al hermanito. Y no hay otra razón, y por eso, muchas veces que eso ha ido cambiando, es que la mujer se vaya vinculando a la educación, pero antes, no ahorita se ve más, y esto es un loco que se ha logrado a través del tiempo, que la mujer vaya a la educación, porque antes no. Y antes había mucho analfabetismo en las comunidades, ahora las mujeres están un poquito más despiertas. Además, de que a los hombres tampoco le gustan mucho que eso suceda porque, o sea, tu cosmovisión va a cambiar, tu pensamiento va a cambiar y es algo que dentro de los territorios no es tan viable, pero sí ha ido cambiando, y nosotros tenemos que buscar metodologías duales. Por ejemplo, lo que decía el compañero, allá llegó el señor de ratón, vamos a ver qué nos dice el señor del ratón... allá llegó [I-A03] con los bichos, vamos a ver cuáles son los bichos y ahí van... Pero ellos hacen un esfuerzo grandísimo, grandísimo, grandísimo. Entonces los encuentros de la modalidad de bienestar familiar... usted nunca ve un hombre. Y son por la tarde. Y nunca ve un hombre... está ocupado, está arreglando la casa, está haciendo otras cosas que.... entonces nosotros para llegar a ellos hay que ser así: puntual.

7:48 ¶ 195 – 200, in Prácticas culturales

6:197 ¶ 869 – 873, in Anexo 2 Transcripcion grupo focal de malaria y leishmaniasis con comunidad afrodescendiente.docx

[INVESTIGADORA] pero volviendo a lo que estábamos hablando ahorita, ¿ustedes creen que hay menos personas dedicadas al campo? O sea, que tal vez eso sea un factor para que haya disminuido la leishmaniasis.

[C-A01]: Ay no, eso sí

[C-A08]: en los últimos años sí.

[C-A09]: la gente, por lo menos mire, nosotros aquí en Santa Cecilia, la gente no compraba revuelto, usted iba a donde el vecino y el vecino le vendía todo. Ahorita no, el revuelto está carísimo, aquí un kilo de plátano vale 3000 pesos. La gente ya casi no...

[C-A01]: pero vea que últimamente y a finales del año pasado, la gente ha tomado consciencia de eso y nuevamente están sembrando, se está apropiando la gente nuevamente del campo porque ya no, la verdad es que la gente no quería cultivar y la generación de nosostros, ahora, nosotros no trabajamos el campo, antes sí. Pero ya último, por la encarecida y todo la gente está cosechando nuevamente.

8:2 ¶ 9 – 10, in Salud

1:153 ¶ 287, in Instituciones_TB.docx

[I-I04] Pues ellos creen los dos, pues. Sí, los dos. El jaibaná y el médico occidental. Ellos acuden primero al médico que trata con jai, y después ellos vienen a los hospitales. Pero también hay unas enfermedades que cura el jai, y el otro que no cura. Ahí, ellos, como confían, pues ellos, se confían de Jaibaná y a veces que no son capaz de curar, ahí sí se queda más... después vienen ya todos malos los niños ya al hospital.

8:18 ¶ 116 – 123, in Salud

4:213 ¶ 1650 – 1657, in INSTITUCIONES ML & LC.docx

[INVESTIGADORA] de hecho en estos días había uno que estaba era como con fiebre y diarrea, porque esa niña estaba en...

[I-M01]: en el centro de recuperación

[I-A03]: de ahí mismo del pueblo, de ahí mismo, era del barrio Cinto y nos quedamos todos aterrados porque, porque...

[I-M01]: ¿y de Piedras también?

[I-A03]: yo supe de dos niños del barrio Cinto que estaban en el CRN, entonces quedé como así... porque yo dije venga... eso es como general aquí que eso pase, pero era... se me hizo raro que en afro pase algo así, se le hace a uno muy raro, porque generalmente nunca pasa.

[INVESTIGADORA]: ¿con desnutrición?

[I-A03]: eso es muy poquito usted ver un afro desnutrido, eso es un chiripazo, entonces yo me quedé como así...

8:23 ¶ 268 – 271, in Salud

6:65 ¶ 941 – 943, in Anexo 2 Transcripcion grupo focal de malaria y leishmaniasis con comunidad afrodescendiente.docx

[C-A01]: mi papá tiene una balsámica para las lombrices.

[C-A02]: acá la mayoría de Pueblo Rico [inaudible]

[C-A01]: y el ajo y la gasolina son buenísimo, la gasolina con el [inaudible] usted coge un poquito de gasolina, le echa tres goticas de limón y se lo toma y se unta [inaudible] buenísimo

8:25 ¶ 248 – 265, in Salud

6:52 ¶ 949 – 965, in Anexo 2 Transcripcion grupo focal de malaria y leishmaniasis con comunidad afrodescendiente.docx

[C-A08]: en la zona indígena, es un día, ayer estaban a punto de echarnos a todos, un niño de Bajo Gitó, cabeza y...

[C-A01]: yo no sé si el centro fue por dos que habían en Sinaí...

[C-A08]: que yo llevé... salió otro [inaudible]

[C-A01]: en Sinaí vi dos y yo les dije a las den centro

[C-A08]: aquí el cierre y por eso bienestar está que arde

[C-A01]: en La Loma también...

[INVESTIGADORA] ya... eso era lo que nos decían ayer... [C-A08]: ¿el centro? El centro tiene cupo para 10 y está ocupado y está pidiendo cupo...

[C-A01]: pero eso se puede pedir los dos cupos adicionales, eso se puede hacer, eso lo hace la coordinadora.

[C-A08]: ahí hay uno...

[C-A01]: pero solicitar el sobrecupo sí se puede para los 12

[C-A08]: pero mira que los han devuelto, ya que no quieran subir, pero los han devuelto.

[C-A01]: esa gestión sí se hace.

[C-A08]: no sabía, pero ahora sé para pelear.

[C-A01]: hacele, que se pueden los 12, eso se manda un correo.

[C-A04]: ese peladito que yo vi... eso parece una menudencia...

[C-A08]: hay un niño del deportivo que también va para esa misma posición, del deportivo.

[C-A01]: yo estuve saliendo de unas actividades, pues yo les dije, yo cumplí, yo ya no trabajo en el Centro, pero estuve saliendo como 3 meses a campo. Me encontré muchos niños con desnutrición, yo les dije ya si los focalizan o no... ya yo no puedo hacer más de ahí. Y a veces uno les colabora a las mamás, les da el pasaje porque uno ve unos niños que uy no...

8:34 ¶ 31 – 32, in Salud

2:136 ¶ 109, in Indigena TB.docx

[C-I03]: Y creo que... Es que yo creo que por el médico... Yo creo que tiene una remedio... Que puede servir mucho la enfermedad de una persona... de ser humano. Pero lo que pasa... Que el médico siempre cuando va a consultar... Él siempre le da a acetaminofén. Acetaminofén. Y no le da una medicamento que sirva. Eso es para un ratico. Pa un rato sirve el aceterminofen. Pa quitar el dolor. Pero no es para curar. Es que a mí me duele cuando va al médico... Que me de un acetaminofén. Y sabiendo que en esa... Médico siempre cana por nosotros. Uno por fijao. Pagamos la plata por resguardo Indígena, por nosotros. Y ellos no siente ellos. Solamente ellos le dan siempre acetaminofén, metrodinazol, que valen los dos 4 mil pesos y no le da para un remedio que sirva, que se calme la enfermedad de uno. Eso quería... ...que siempre pasa eso.

8:35 ¶ 33 – 34, in Salud

3:2 ¶ 252, in AFRO_TB.docx

[A06]: pues eso dependiendo, pues sí, yo lo diría como que dependiendo como que del día de que uno se levante con el pie izquierdo, el derecho, el santo, porque sí atienden, pero a veces va por una urgencia porque usted se aguantó el dolor. Aquí está y usted puede tener un dolorcito y usted puede decir, no yo me lo aguanto y no le doy a este dolorcito aquí y en algunas ocasiones usted va a determinado sitio y le dicen "ay, el médico ya se fue". Así esté ahí, por no querer atender, o le dicen "no, es que esto no es una urgencia". Y si usted va es porque ya usted, su nivel de tolerancia con el dolor, usted no lo soporta. Y ya usted se automedicó alguna bebida, alguna cosita que lo [inaudible] ya se lo hizo, entonces en esa parte, pues sí atienden, pero hay unos días, sea aquí o en cualquier otro sitio de este pueblo, de cualquier parte de Colombia, hay un día de que algo sucede allá.

8:37 ¶ 125 – 128, in Salud

4:218 ¶ 1675 – 1677, in INSTITUCIONES ML & LC.docx

[I-I07]: la diarrea con no lavar bien los alimentos, eso pasa, como dicen por ahí. Llegan a comer el alimento, rapidito lavan, rapidito y listo con su cuchillo. A veces eso pasa, se contamina. Y a veces, lo indígenas, a veces, cuando tienen niños de 3 años a veces mandan solos a ensuciar al baño y no acompañan a veces y como el niño, a veces el no limpia eso, entonces ahí está. A veces mete la mano en la boca y ahí sale la... y a veces también se contaminan jugando en el patio y se meten la comida, porque a veces los padres de familia a veces no están pendientes de sus hijos, eso pasa mucho con el indígena a veces. Y la respiración en [inaudible] por

la cocina como dijimos ahorita, a veces en la misma pieza hay cocina, digamoslo... se contamina con eso también y a veces también llevan a otra comunidad cuando está serenando.

[INVESTIGADORA]: ¿cuando está qué?

[I-I07]: cuando está serenando, a eso también afecta la respiración y también las mamás a veces fuman cigarro, tienen la mano cerca del niño y ellos se afectan la respiración, eso pasa y de [inaudible] y la IRA y también le da malaria también y leishmaniasis también da, no mucho, pero eso tiene su riesgo en este momento, a veces. Eso es lo que pasa en la comunidad

8:41 ¶ 135 – 141, in Salud

4:269 ¶ 266 – 271, in INSTITUCIONES ML & LC.docx

[I-M01]: Bueno, pues si voy a hablar un poquito. Pues, de parte de mi trabajo y como amiga de ellos, porque pues yo tengo mucho tiempo distinguiendo de los indígenas y he aprendido a conocerlos como tal. Bueno, como ya sabemos, ellos tienen sus propias creencias, ¿cierto? Pero también hay unos que también han ido cambiando esas creencias. Entonces, cuando se enferma un niño, si está bien primero jaibaná, hay unos que primero jaibaná, como hay otros que ya han cambiado esa mentalidad, y van al hospital, ¿cierto? Entonces, ellos, ¿qué dicen? Bueno, llevamos el niño al jaibaná, en cuanto se enferma. Otras veces se demoran para llevarlo al jaibaná. ¿Por qué? Porque quizás no tienen el dinero para levarlo, porque un jaibaná cobra, eso no es gratis. EL jaibaná cobra.

[I-I02]: cobran 30 mil.

[INVESTIGADORA] ¿ah sí?

[I-M01]: sí señora el jaibaná. Otras veces unos cobran 30 mil y otros pueden cobrar más.

[I-I02]: a veces cobran 50.

[I-M01]: Pues dependiendo de lo que tenga el niño, igual hasta una parteras, las parteras también cobran. Todos ellos cobran, eso no es gratis. Entonces, digamos así, llevan el niño, pues los dos primeros días al jaibaná, o los llevan cuando el niño lleva varios días enfermo, cuando el niño ya está muy complicado. Entonces, mientras el jaibaná hace el ritual que hacen ellos, digamos dos o tres días, y que él ve que el niño definitivamente no mejora, lo manda pues al hospital. Ya un poco complicado porque imagínese, todo ese tiempo el niño enfermoo. Entonces es ahí donde el niño llega muy complicado al hospital. Puede que en el hospital lo recuperen o sencillamente ya demasiado tarde.

8:48 ¶ 47 – 48, in Salud

3:111 ¶ 444, in AFRO_TB.docx

[A07]: los extramurales no se dan para el pueblo negro. Sólo se da para las comunidades indígenas.

8:50 ¶ 56 – 60, in Salud

3:117 ¶ 425 – 428, in AFRO_TB.docx

[A01]: y yo digo que hace falta más médicos.

[A07]: claro.

[A01]: somos mucha población para dos médicos y eso genera mucha...

[A07]: somos mucha población para no tener un hospital. Si nuestra estructura física... tenemos el puesto de salud más grande de Colombia, al que se le invirtieron cuatro mil ochocientos... el puesto de salud de Santa Cecilia es el más grande de Colombia. ¿Tú sabias eso? El puesto de salud de Santa Cecilia es el más grande.

8:51 ¶ 61 – 64, in Salud

3:119 ¶ 264 – 266, in AFRO_TB.docx

[A02]: sí, yo que día madrugue y cogí una cita. me la dieron para las 9:30 de la mañana. Eran las 3 de la tarde y no me habían atendido.

[A01] y llega usted a las 9:40 y le dicen que ya no la pueden atender porque...

[A02]: me tocó irme para la casa y luego regresar porque las 3 de la tarde y el médico no me había atendido. Esperando ahí desde las 9 de la mañana. [inaudible] no la cita, yo ya tenía la cita agendada, o sea, el ficho, para sacar la cita a uno lo atienden a las 10 de la mañana y a uno lo atienden normal, pero ya era la atención médica como tal.

8:57 ¶ 80 – 82, in Salud

4:43 ¶ 1420 – 1421, in INSTITUCIONES ML & LC.docx

o solo dan es acetaminofén, pero que no atienden también, el tema de las fichas...

[I-M01]: o que si no es una urgencia

8:59 ¶ 87 – 88, in Salud

4:82 ¶ 1678, in INSTITUCIONES ML & LC.docx

[I-M01]: también de los factores que influyen en cuanto a desnutrición, digamoslo así, eh... las madres sin lunas tienden a darle lactancia materna al niño, ¿cierto? Eso sí ellas son muy juiciosas en eso y la lactancia materna exclusiva es hasta los seis meses, ¿cierto? Pero ella el niño cumple los seis meses y le siguen dando leche materna, entonces eso no le aporta los nutrientes que el niño necesita, entonces ya no van a estar como tan preocupadas de ir a hacerle como la alimentación complementaria que la sopita, que la cremita, sino que si le llegó las 8 y no hay nada [inaudible] entonces ya es la leche, entonces no se preocupan tanto como por una alimentación complementaria que le aporte los nutrientes al niño, sino por esa leche. Entonces esa es una de los factores que lleva a los niños a la desnutrición y

la otra es la comida a deshoras, o sea, no son como muy puntuales en el momento de la la alimentación al niño y que tampoco tienen los nutrientes pues... no le aportan los nutrientes suficientes al niño, como... digamos que si tienen primitivo, es primitivo, porque digamos que las condiciones del indígena es muy diferente, ellos no mantienen como todas esas oportunidades que uno tiene, entonces ellos parecen más bien de bajos recursos, digámoslo así, entonces a ellos no les preocupa tanto pues que haya un buen almuerzo o que haya un buen desayuno. Entonces eso lleva como a la desnutrición como tal.

8:62 ¶ 89 – 90, in Salud

4:95 ¶ 237, in INSTITUCIONES ML & LC.docx

y hay responsabilidad de la salud también. Porque la gerente trata de hacer muchas cosas, pero hay muchas cosas que se les salen de las manos, y dentro esas cosas que se le salen de las manos es que de pronto no consigue un médico o apenas hay uno solo y es el que atiende todo y no le va a dar para hacer todo, y ella tiene la voluntad de hacerlo, pero no da. Entonces también, no es un secreto que tal vez cuando llegan al centro de salud no los atendemos de la mejor manera, entonces se junta todo, todo se junta. Entonces... cuando nosotros abordamos los temas, sobre todo esos casos que yo no quiero ni mencionar aquí, pero que estamos alborotados todos... uno trata de ayudar.

8:64 ¶ 112 – 115, in Salud

4:199 ¶ 1407 – 1409, in INSTITUCIONES ML & LC.docx

[I-I07]: el acetaminofén [inaudible]

[I-I02]: y a veces en el hospital, a veces el niño tiene malaria y...

[I-I07]: si tiene infección o algo así: acetaminofen.

8:67 ¶ 110 – 111, in Salud

4:170 ¶ 278, in INSTITUCIONES ML & LC.docx

los de la modalidad tiene que estar pendiente de 350 beneficiarios, ellos tienen que saber quién está enfermo y quién no... entonces llegamos al hospital y encontramos la barrera, porque no nos articulamos. Entonces, es lo que le pasa a uno "llevemos al niño al puesto de salud... pero resulta de que llega al puesto salud y hay un triaje donde se clasifica y dice que el niño no es una urgencia, pero, que lo que la clasificación la clasificación nos da es para una consulta externa, pero, nosotros dentro de la cosmovisión indígena, sabemos de que si se lo llevan, no va a volver. Entonces bueno, entonces, qué vamos a hacer.

8:71 ¶ 213 – 214, in Salud

6:19 ¶ 666 – 671, in Anexo 2 Transcripcion grupo focal de malaria y leishmaniasis con comunidad afrodescendiente.docx

[C-A06]: por ejemplo, Santa Cecilia como tal, es un puesto de salud, no es un hospital, por ejemplo, el problema aquí es el cambio, de que hoy permanece un médico, mañana permanece otro, así sucesivamente, supuestamente hay un médico para estar las 24 horas, cosa que nunca se ha dado

8:72 ¶ 220 – 221, in Salud

6:36 ¶ 664 – 665, in Anexo 2 Transcripcion grupo focal de malaria y leishmaniasis con comunidad afrodescendiente.docx

[C-A01]: yo digo que hace falta más de personal médico, porqe el personal médico no tiene la capacidad para atender tanta población, son dos médicos para tanta gente, eso les genera estrés mental y de todo.

8:81 ¶ 317 – 323, in Salud

6:206 ¶ 683 – 688, in Anexo 2 Transcripcion grupo focal de malaria y leishmaniasis con comunidad afrodescendiente.docx

[INVESTIGADORA] yo les preguntaba ahorita, los médicos que están trabajando aquí, bueno el personal, ¿son de afuera? [C-A08]: de afuera, ni los de aquí quieren trabajar.

[C-A06]: por ejemplo, hay uno que va a trabajar hasta el 8 de este mes, el flaquito, Daniel, que ya se va.

[INVESTIGADORA] ¿y por qué se va? [C-A06]: porque ya terminó el rural y él ya se va. Ahora viene... o sea que acá el médico acá, especializado en sí, no mandan, no vienen, o sea, no vienen que eso acá es un hueco, que porque eso acá es zona roja.

[C-A08]: y por la fama que se ha generado

[C-A06]: y por la fama que se ha generado, entonces les da hasta miedo venir.

[INVESTIGADORA] ¿qué fama se ha generado? [C-A08]: lo que pasa es que acá se ha generado una fama en [inaudible] entonces esa fama la generan y eso se ha creado que Santa Cecilia es la peor cosa que puede exisitir a nivel regional, entonces muchos médicos también evitan venir.

9:4 ¶ 17 – 18, in Corresponsabilidad

1:53 ¶ 471, in Instituciones_TB.docx

[I-M08] Ellos son muy enfermos de otras cosas, entonces [inaudible] la desnutrición es muy alta, entonces no hay sistema de defensa.

9:27 ¶ 80 – 86, in Corresponsabilidad

3:46 ¶ 270 – 275, in AFRO_TB.docx

[A06]: de 7 a 6 de la tarde funciona.

[A02]: de 7 a 7, siete de la mañana a siete de la noche.

[A06]: no, yo ese día que fui con mi mamá lo primero que me dijeron fue "no es que el médico ya se fue hasta el celador"

[A02]: cuando tiene una urgencia vital el médico se va, pero supuestamente es de 7 a 7, si yo fui ese día a la 1 de la tarde y no estaba el médico y era las 5 de la tarde y no había vuelto, se va.

[A06]: y así estén ahí chateando, como uno no sabe quién es quién... ah no, es que el médico ya se fue y puede estar ahí chateando.

[A02]: entonces que ah, llega alguien para una urgencia, que no que es que el médico se fue a almorzar y llega hasta las 3, si quiere espera, si no pues...

○ Malos comportamientos_LC

62 Citas:

4:100 ¶ 329, in Comportamientos

[I-M05]: yo tengo la cicatriz, yo me acuerdo que era jabón rey, no me acuerdo qué más cosas le echaban a uno...

4:105 ¶ 343 – 347, in Comportamientos

4:13 ¶ 1034 – 1041, in INSTITUCIONES ML & LC.docx

[INVESTIGADORA] pero en leishmaniasis, [I-A03] nos dice que el afro y el mestizo va al médico, ¿el indígena va al médico por leishmaniasis, porque también lo trata con..?

[I-I07]: ah... pues a veces pues depende.

[I-I04]: como dice... dependiendo la distancia de la comunidad.

[I-A03]: por ejemplo, yo digo, yo al encanto fui, pero allá... es que eso es muy lejos

4:109 ¶ 364 – 365, in Comportamientos

mientras que la comunidad indígena sí es más receptiva como de ir al hospital, primero, que le tienen pánico a las inyecciones, porque ellos ven una inyección y eso es una muerte lenta que ay no... y no...

[I-M06]: y no terminan el tratamiento...

4:113 ¶ 382 – 386, in Comportamientos

4:46 ¶ 1088 – 1091, in INSTITUCIONES ML & LC.docx

I-A03]: uy respecto a eso, de todas maneras si usted a la comunidad a 8 horas de camino... créame que usted no aguanta, usted irá al primero y el segundo día, pero el tercero usted no lo va, porque el primero... pues a mí nunca me han aplicado,

pero yo sé que eso duele porque todos a los que lo han aplicado yo sé que eso duele mucho y usted cree que a ese cansancio físico, esa subida, que es una subida de papá y mamá...

[I-M05]: es que sería irse llegar, medio dormir y volver a voltear.

[I-A03]: es que mire a qué hora salen del Encanto, se vienen tipo tres de la mañana, del encanto, mire a qué horas están llegando a Itaurí, porque es que eso está desde muy arriba, mientras baja un bus, para ellos desplazarse a santa cecilia, mientras llega, les aplican el medicamento, esperan otro bus para regresarse y pegan la loma, ¿a qué horas están llegando?

[I-M01]: esa es una de las cosas que a ellos les da como pereza ir al hospital y eso: la distancia, porque es que eso es demasiado

4:118 ¶ 402 – 404, in Comportamientos

4:65 ¶ 648 – 649, in INSTITUCIONES ML & LC.docx

[I-M06]: el tratamiento de la leishmaniasis todo el mundo sabe que es en el hospital. Lo único es que hay mucha deserción. No terminan tratamiento, porque igual eso duele mucho y son muchas inyecciones, por eso es que yo preguntaba por la efectividad de las tabletas, porque eso daría a disminuir el abandono del tratamiento.

[I-M05]: también las pastillas son muy fuertes, son fuertes y eso la gastritis es... una señora una vez le mandaron y ella decía, no yo prefería las inyecciones y pues por algo no le mandarían las inyecciones y ella que no que la enfermaban y [inaudible]

4:122 ¶ 425 – 432, in Comportamientos

4:91 ¶ 1052 – 1058, in INSTITUCIONES ML & LC.docx

[I-M01]: ¿pero allá sí han habido casos de leishmaniasis?

[I-A03]: allá sí hay casos de leishmaniasis

[I-M01]: pero entonces no tienen tratamiento, ¿cómo se curan?

[I-A03]: se hacen los tratamientos

[inaudible, algarabía]

[I-M01]: y entonces ahí cómo hacen?

[I-A03]: hospitalizarlos, porque cuando la herida está demasiada... eh... grande o demasiado infectada y ellos ni con el tratamiento casero, como lo llaman ellos, ni con las inyecciones porque no se la aplican, los tienen que hospitalizar y es la única forma de poder ir al tratamiento, hospitalizándolos, de lo contrario, no hay como

4:127 ¶ 466 – 467, in Comportamientos

4:120 ¶ 71, in INSTITUCIONES ML & LC.docx

[I-M06]: Aparte de eso, súmenle los animales que también duermen juntos. El animal que tenga, por lo general al indígena le gusta mucho, mucho, mucho, mucho un perro. Mucho, les gusta demasiado, los perros les encantan, les encantan los perros. Y usted ve que hay viviendas donde hay, a veces, hasta más perros que hijos.

4:128 ¶ 468 – 484, in Comportamientos

4:125 ¶ 1135 – 1154, in INSTITUCIONES ML & LC.docx

[INVESTIGADORA] y para leishmaniasis han dicho antes que sí funciona la medicina tradicional o funciona para... o sea, no digo que funciona o no, pero la gente va.

[I-M05]: la medicina tradicional apra la leishmaniasis. Pues yo solamente conozco una persona que hace el tratamiento y funciona no sé los demás.

[INVESTIGADORA] ¿ustedes conocen gente que sepa tratar la leishmaniasis?

[I-M05]: yo una

[I-I07]: ¿a quién?

[I-M05]: a Orlando, Orlando el de Dokabú

[I-I07]: sí, eso he escuchado, pero Orlando, qué medicamento... no sé, ¿qué planta medicinal? Lo que yo sé es que él compra ese, viene en la bolsita blanco polvo... ese.. se me olvida ese nombre, es una bolsita viene un polvo como en blanco, pero eso parecía... Azufre...

[I-M01]: tomebol?

[I-I07]: exacto

[I-M01]: ácido fusídico

[I-I07]: a veces eso... Orlando ha hecho con eso también tratamiento.

[I-M01]: yo no sé qué tratamiento utiliza

[I-I07]: lo que yo no he visto es mucha planta, no, no estoy seguro, no sé, porque él venía como hacer un [inaudible] leishmaniasis venía trabajando así conmigo, es lo que conocía.

[I-M01]: él le hizo el tratamiento a una niña

[I-I04]: o será que él prepara con eso, con la planta, debe preparar con eso...

[I-I02]: iba echando

4:129 ¶ 485 – 486, in Comportamientos

4:132 ¶ 552, in INSTITUCIONES ML & LC.docx

[I-A03]: y como ellos dicen no es que nosotros vivimos muy lejos y todos los días bajar por la inyección, entonces abandonan el tratamiento, o sea, que si el bichito no muere siempre va a estar ahí, porque él lo que hace es que comió aquí, comió por acá y como están ahí escondidos, entonces por eso es que generalmente los indígena acostumbran a hacerse el tratamiento en su vereda

4:133 ¶ 527 – 530, in Comportamientos

4:144 ¶ 54 – 56, in INSTITUCIONES ML & LC.docx

Otra cosa, se ha hecho mucho énfasis también en el toldillo porque resulta que hemos tenido una experiencia muy maluca, porque entregamos los toldillos y cuando salimos, ya están pescando. Me ha tocado evidenciar eso, me ha tocado llamar al gobernador de la comunidad a decirle "venga, ¿qué pasó aquí? Hacia 15 minutos, nosotros terminamos de entregar y cuando el río se creció, cuando eso parecía una jauría, corrían para el río. Entonces, mire, donde queda... todo se pierde, y luego los toldillos terminan como cerca de los sembrados. No estoy diciendo nada que no sea cierto.

[INVESTIGADORA] ¿Por qué crees que pase eso?

[I-M01]: eso, ahí es dónde voy. Resulta que ese toldillo viene con algo, no sé [un repelente]. A algunos les cae mal ese repelente, no les gusta, no les da alergia, sí, entonces esa parte como que no.

4:137 ¶ 552 – 558, in Comportamientos

4:160 ¶ 1023 – 1028, in INSTITUCIONES ML & LC.docx

[INVESTIGADORA] ¿leishmaniasis?

[I-M05]: pues por ahí...

[I-A03]: es que el que más asiste a tratamiento para malaria es el afro y el mestizo, ve, perdón para la leishmaniasis. Porque el indígena siempre va a tender a abandonar. Si le aplicaron las dos primeras inyecciones...

[I-M01]: es que el tratamiento que ellos utilizan para la leishmaniasis es un chuzón, porque si no van hospital...

[I-A03]: porque ellos siempre, es como ellos dicen como son tantas inyecciones y con el peso de la persona... no es que... es entendible porque es que hay personas, un ejemplo, pongamos un ejemplo, los del encanto, el encanto está es en la mismísima conchcinchina, es que eso está muy lejos. Imagínese uno viajar todos los días del Encanto para que le pongan una inyección... por dios bendigo, yo fui, pero le digo que yo no tengo ganas de volver perdóneme.

[I-M01]: pero entonces el tratamiento les funciona, pero no hablando de medicina... pues sino las plantas de ellos.

4:143 ¶ 584 – 586, in Comportamientos

4:176 ¶ 1070 – 1071, in INSTITUCIONES ML & LC.docx

[INVESTIGADORA] claro, porque es un tratamiento largo. Si ustedes tuvieran que escoger alguna de las dos con cuál se quedan? Con la facilidad del medicamento y que la apliquen bien o por la facilidad de tener el medicamento?

[I-M05]: a mí me pasó una vez, por ejemplo, yo trabajaba en la villa y un niño, un niño de Citrú salió positivo, entonces nosotros hablamos en el hospital porque la mamá aplicaba inyecciones, entonces hablamos en el hospital si la mamá se lo podía aplicar y resulta que tuvieron que irse de que cuando el director, cuando eso era [inaudible], entonces el director "ve, es que en este tratamiento le mandaron muy poquito, miremos" y él dijo sí, es muy poquito, el mismo doctor le cambió la dosis, la mamá se lo estaba aplicando cuando una vez llega al hospital y me dice el que era el sepulturero "cómo se le ocurre que usted intoxicó un niño con glucantin", y sí ahí llegó un niño intoxicado con glucantin y a mí me aparece que usted le había cambiado la dosis, y oiga qué niño, cuando era ese niño, cuando yo le dije, doctor acuérdese que había un niño al que se le cambió la dosis, fue usted y yo no estaba aplicando el medicamento porque acuérdese que ustedes habían autorizado que la mamá se lo aplicara, porque es que el niño que iba a ir todos los días a Villa Claré, eso sí es mentira y resulta que no solo le estaba aplicando con una solo jeringa el tratamiento, sino que el niño se cogió un tarro de esos y se lo tomó y ya según él ya la culpa era mía y no, yo no tengo nada que ver, pero ahora llego al hospital y era todo el mundo regañándome porque yo iba a mataar a ese niño. Entonces cuando dice el médico, no yo fui el que le cambié el tratamiento porque era muy poquito, pero entonces ya... o sea que sí corren peligro.

4:146 ¶ 596 – 603, in Comportamientos

4:183 ¶ 399 – 405, in INSTITUCIONES ML & LC.docx

[INVESTIGADORA] entonces, vamos a ponerle soluciones caseras que es como ácido de batería ¿y qué más?

[I-M01]: eh... con hipoclorito...

[INVESTIGADORA] bueno, esto va para leishmaniasis.

[I-M06]: ácido de batería, las hierbas amargas, [inaudible]

[inaudible]

[I-A03]: he visto mucho que se aplican esmalte. Ellos se pitan se echan esmalte por el... por el... acetona.

[I-M01]: con limón caliente.

4:149 ¶ 610 – 613, in Comportamientos

4:203 ¶ 74 – 77, in INSTITUCIONES ML & LC.docx

[I-I07]: solo que ellos instalan el toldillo en la pared pero, ellos mismos dicen que [inaudible].

[I-M06]: exacto, entonces la armadera, desarmar eso...

[I-I07]: a veces sueltan [inaudible]

4:154 ¶ 639 – 642, in Comportamientos

4:215 ¶ 1189 – 1191, in INSTITUCIONES ML & LC.docx

I-M05]: por ejemplo, en la Villa, a veces con el ejército se conseguía el tratamiento.

[I-M01]: ¿de leishmaniasis?

[I-A03]: ah, es que por eso [inaudible] porque usted sabe que ellos tienen sus médicos, ellos tienen todo ahi a la mano, entonces ellos...

4:156 ¶ 648 – 661, in Comportamientos

4:220 ¶ 1005 – 1017, in INSTITUCIONES ML & LC.docx

[INVESTIGADORA] ¿y los que usan plantas medicinales igual se hacen antes la prueba para saber si sí..?

[I-A03]: pues hay algunos que no se la hacen, hay muchos que no se hacen...

[I-M05]: ya si nos les sana ahí sí van al hospital

[I-I02]: algunos no la hacen

[I-M01]: pues yo pienso que ellos para saber si es leishmaniasis tienen que hacérsela

[I-A03]: no porque mire que...

[I-M01]: ellos le hacen así y ya

[algarabía]

[I-M01]: y puede ser un hongo

[I-A03]: eso parece... eso cuando está iniciando ellos empiezan es a aplicarse, aplicarse, a aplicarse cosas. Entonces cuando ya no tiene solución es cuando ellos acuden, pero ya cuando la leishmaniasis se le convirtió en un área infectada, a veces, no hay ni siquiera leishmaniasis, se convirtió en un área infectada y de tanto echarse cosas quedó... y cuando menos piensan tienen tremenda lesión.

[INVESTIGADORA] entonces le ponemos un amarillo?

[VARIAS VOCES]: sí

[INVESTIGADORA] ¿unos sí y otro no o ninguno

4:157 ¶ 662 – 664, in Comportamientos

4:221 ¶ 897 – 898, in INSTITUCIONES ML & LC.docx

[I-A03]: recolección de inservibles porque generalmente comunidad donde vamos hacemos recolección de inservibles, de hecho...

[INVESTIGADORA] ¿pero la comunidad sigue manteniendo eso? [I-A03]: mientras está uno allá. Es verdad, no nos digamos mentiras... mientras está uno allá está todo limpio, porque claro, hay comunidades muy organizadas, hay comunidades que... persiguen, continúan con la recolección, como hay otras que es únicamente en el momento que uno está. De hecho, estos días toca programar una, que hay que ir a la alcaldía que nos facilite la volqueta porque hay que ir desde la punta hacia... hacia... eso sí lo hemos hecho también.

4:158 ¶ 665 – 668, in Comportamientos

4:229 ¶ 109 – 114, in INSTITUCIONES ML & LC.docx

[I-I02]: a la sobrinita mía le dio uno, leishmaniasis, en los piecitos y mi mamá con una planta lo machucó y lavaba con jabón, bien limpio, lo lavó y con ese ya... se curó.

[INVESTIGADORA] ¿Sí? ¿Y le quedó cicatriz?

[I-I02]: Sí, pero un poquitico cicatriz, pero no comió mucho, pero sí quedó cicatriz.

4:159 ¶ 672 – 676, in Comportamientos

4:231 ¶ 1096 – 1108, in INSTITUCIONES ML & LC.docx

[INVESTIGADORA] y el de leishmaniasis?

[I-I02]: amarillo

[I-I04]: amarillo

[I-A03]: leishmaniasis, sí amarillo, porque... porque leishamaniasis la verdad... de pronto el que vaya a que el tomen a que se haga el tratamiento tiene que ser que viva o en Santa Teresa o que viva en la Punta, o en Dokabú, o que viva en Chifá o que viva en Santa Marta porque son veredas muy cerquina y eso que hasta caminando se van, pero si es como... Minitas, las Tumácaras, Caja de Oro... esa otra que queda por acá arriba.

4:164 ¶ 692 – 698, in Comportamientos

4:262 ¶ 963 – 968, in INSTITUCIONES ML & LC.docx

[INVESTIGADORA] También habíamos escuchado otras cosas que... con los niños, ¿cómo es que los protegen?

[I-M05]: ah que los, según lo que dijo [anónimo] ayer, que los, ellos hacen el fogón dentro de la casa es porque el humo les sirve para ahuyentar los... los zancudos, que por eso es que las hamacas de los niños también las ponen cerca del fogón.

[I-M01]: esa no la sabía

[I-A03]: usted es embera, ustedes deben de saber que si...

[I-M01]: no...

[I-I07]: lo que yo he visto es que cerca del niño nos ponen... como en una olla, ¿no? Ahí mismito hacen como una olla, el humo...

4:166 ¶ 714 – 720, in Comportamientos

4:292 ¶ 932 – 939, in INSTITUCIONES ML & LC.docx

isto, el repelente

[I-I07]: rojo

[I-A03]: rojo porque el repelente lo usan es para jugar. Es que con la experiencia que ya tuvimos...

[I-I04]: no se usa mucho en...

[INVESTIGADORA] la cultura indígena no usa mucho... ¿y el afro? ¿Usa repelente?

[I-A03]: no todos, hay algunos que no.

4:169 ¶ 730 – 732, in Comportamientos

4:300 ¶ 58 – 59, in INSTITUCIONES ML & LC.docx

[INVESTIGADORA] Bueno, esa es una razón. ¿Qué otra razón puede haber para que la gente no los use? ¿Qué creen ustedes?

[I-M01]: al indígena no le gusta. A ellos no les gusta dormir encerrados.

4:170 ¶ 733 – 742, in Comportamientos

4:309 ¶ 1072 – 1080, in INSTITUCIONES ML & LC.docx

[I-A03]: con seguridad fue en el hospital, porque uno se cura en salud con muchas cosas de pronto hizo una reacción adversa entonces el problema sobre quién va a quedar?

[I-M01]: pues la verdad yo soy enfermera, pero a mí nada de esas cosas me gusta hacerlas, yo solamente las hago con mi familia, pero así que el vecino diga "ah, venga", ah, no que pena, pero no.

[I-A03]: es mejor, por seguridad.

[I-M01]: sí me ha tocado decir que el trabajo que tengo no me permite porque no, lo mío es promoción y prevención, a mí no me tienen autorizado de colocar medicamentos, dirán que uno es malagente, pero uno no es mala gente, uno se tiene que cuidar en salud, por cualquier cosa me llega a... y dónde queda [inaudible] no... ni en las comunidades indígenas, yo eso sí no lo hago.

[I-I02]: por eso me mantiene regañando mi marido.

[I-M01]: eso que ella hace, yo no lo hago

[I-A03]: porque es que vea, el problema es que usted no sabe qué reacción adversa pueda tener el medicamento que usted está aplicando

[I-I02]: [inaudbile] echan culpa a usted que usted hizo este y para echar culpa, para que no ponga así, mejor no...

[I-A03]: es que es verdad, además que es muy delicado del caso de... hace tiempo ya, alguien comenzó a trbaajr en el puesto de salud de santa cecilia y ese alguien no tenía experiencia en inyectología, le aplicó una inyección a un niño que por poquito lo deja invalido. Y eso dio vueltas y vueltas y vueltas y eso estuvo muy delicado, porque el niño estuvo mucho tiempo en una cama, casi lo deja inválido.

4:171 ¶ 743 – 752, in Comportamientos

4:314 ¶ 921 – 931, in INSTITUCIONES ML & LC.docx

[INVESTIGADORA] ¿el toldillo? Antes hablamos harto de eso, ¿el toldillo funciona? ¿Funciona, no funciona, más o menos?

[I-M01]: más o menos

[I-A03]: parcialmente

[I-M01]: para no decir que no.

[I-I04]: en algunos ítems.

[I-A03]: para las moscas

[I-I02]: no funciona.

[INVESTIGADORA] ahí le ponemos...

[I-I02]: amarilla... verde

4:172 ¶ 753 – 767, in Comportamientos

4:315 ¶ 902 – 915, in INSTITUCIONES ML & LC.docx

[INVESTIGADORA] ¿qué más en prevención? ¿la gente usa ropa larga cuando sale..?

[VARIAS VOCES] no

[I-I07]: más o menos, algunos salen con su licra por debajo, con guantes a la finca...

[I-A03]: de pronto las mujeres

[I-I07]: las mujeres...

[I-A03]: claro que las mujeres generalmente van de vestido de manga larga.

[I-I07]: a veces llevan licra por debajo.

[I-A03]: me refiero a que ellas, pues su vestido, el vestido es de manga larga, es muy poquita la que utiliza...

[INVESTIGADORA] ¿y las piernas? [I-I07]: sí, a veces usan licra, todos no, pero algunos.

[INVESTIGADORA] pero lo usan cuando van a ir a...

[I-I07]: a la finca

[I-I04]: a trabajar.

[INVESTIGADORA] ¿entonces amarillo? [I-I07]: sí, amarillo

[I-A03]: sí, porque todas no. Algunas sí, otras no. Y muchas se van en... he visto muchas... no sé si... tienen que picarle, he visto muchas de pronto con la falda y como el niño se lo amarraron a la espalda... pues, imagínese ahí, hay más...

4:173 ¶ 768 – 771, in Comportamientos

4:321 ¶ 390 – 392, in INSTITUCIONES ML & LC.docx

[FACILITADORA]: ¿en leishmaniasis también se automedican?

[I-M06]: no, es que ese es con inyecciones...

[I-M05]: pero las cosas caseras... sí se hacen muchas cosas casera. Sí, se queman con ácidos...

4:176 ¶ 779 – 780, in Comportamientos

4:328 ¶ 893, in INSTITUCIONES ML & LC.docx

I-I02]: toldillo no.

4:182 ¶ 798 – 808, in Comportamientos

4:347 ¶ 1042 – 1051, in INSTITUCIONES ML & LC.docx

[I-M01]: pero entonces, [I-A03], qué pasa con los del campo que usted dice, si tienen leishmaniasis, ¿entonces qué hacen? ¿Ahí funciona es la planta, porque cómo se van a curar?

[I-A03]: muchos a veces, en ocasiones, algunos tienen que bajarse de la comunidad para aplicarse el tratamiento, de pronto donde un familiar o algo, porque es que vea, miremos a cuántas horas está el Ecanto de acá, porque uno se mente Itaurí, sigue a Cundumí, de Condumí...

[I-M01]: ¿son unas seis horas?

[I-A03]: son más horas.

[I-M01]: ¿siete horas?

[I-A03]: más, ponga 8 horas porque

[[FACILITADOR]A]: ese es antes de Tumatá o después?

[I-A03]: antes, están jodidos, porque yo me eché ocho horas

[I-I07]: ¿de dónde?

[I-A03]: de acá, salí de acá para ir al Encanto, y sola, porque no iba con nadie, camiandndo, en ese entonces yo caminaba, ahora ya no camino tanto. Sí porque en ese entonces estaba bien sana, no tenía nada, entonces me rendía, salí de mi casa a las 5 de la mañana y regresé a mi casa a las 10 y media de la noche y me toco venir sola y con literna. ünicamente por bajonar, tres perros, no, porque es que hay un accidente, el problema me toca a mí y yo no me puedo exponer, la visita a la escuela únicamente era para las actividades que hbaía que hacer, entonces mire y verá, como hay muchos que dicen "no es que mire", es que mire la distancia tan grande para usted venir a que le apliquen la inyección, se tiene que venir madrugado y cuando tiene que coger camino ya el dolor es demasiado, porque el dolor de la inyección, llevan dos tres días seguidos, el cansancio, el dolor de la inyección...

4:188 ¶ 824 – 825, in Comportamientos

4:358 ¶ 945, in INSTITUCIONES ML & LC.docx

[INVESTIGADORA] entonces digamos que... con los afros funciona un poco mejor que con los indígenas. [I-A03]: porque algunos afros utilizan repelente, bueno no todos, pero unos...

4:191 ¶ 828 – 830, in Comportamientos

4:374 ¶ 66 – 67, in INSTITUCIONES ML & LC.docx

[I-M01] yo soy una que toldillo no uso, a mí me da calor el toldillo, no me gusta como el encierro ahí, de estar ahí metida. [INVESTIGADORA] Pero la mayoría de viviendas son abiertas... Entonces, al ser abiertas...

[I-A03]: No les debería de dar.

4:192 ¶ 831 – 832, in Comportamientos

4:376 ¶ 65, in INSTITUCIONES ML & LC.docx

[INVESTIGADORA] eso es cuando, digamos, uno ya lo entendió, se acomodó y pues lo usa y se acostumbra, pero entonces si uno no lo usa y no le gusta, pues... [I-I07]: da calor. [I-A03]: o sea, da calor pero ya.

4:198 ¶ 855 – 857, in Comportamientos

5:9 ¶ 599 – 600, in Anexo 1 Transcripcion grupo focal de malaria y leishmaniasis con comunidad indígena.docx

¿en las comunidades de ustedes les gusta el toldillo?

[C-I07]: no tienen platica con qué comprar.

4:200 ¶ 862 – 865, in Comportamientos

5:13 ¶ 433 – 435, in Anexo 1 Transcripcion grupo focal de malaria y leishmaniasis con comunidad indígena.docx

[C-I02]: yo curo la leishmaniasis en la forma en que me enseñó un médico jaibaná. Yo preparaba azufre con carbón bien... bien quemadito, raspadito, luego cogía brea.

[INVESTIGADORA] ¿mata de brea? [C-I02]: mata de brea, eso es muy difícil de conseguir ahora y cogía un poquitico de legudón.

[INVESTIGADORA] ¿legudón? [C-I02]: legudón, que mata gusanos para... preparar las tres cositas, lavar bien lavadito, luego le tapaba yo bien tapadito. Bueno... y otro día, se suelta eso tapado, bien tiene mucha agua, como un baba muy pegajoso, eso hierva y se chupa todo, todo. El [inaudible] queda hasta aquí, vuelva usted y tapa, mete con las 3 cositas preparado, cubre varios... yo después de vacunador se me olvidó el preparado que yo usaba, con eso curé mucho paciente curaba hasta morenos, hasta indígenas, eso era todo, pero ya no estoy utilizando.

4:202 ¶ 871 – 873, in Comportamientos

5:18 ¶ 674 – 675, in Anexo 1 Transcripcion grupo focal de malaria y leishmaniasis con comunidad indígena.docx

[INVESTIGADORA] en el terreno... listo, pero entonces, lo del repelente le ponemos rojo porque no se usa, ¿cierto?

[C-I04]: sí.

4:206 ¶ 884 – 888, in Comportamientos

5:25 ¶ 463 – 466, in Anexo 1 Transcripcion grupo focal de malaria y leishmaniasis con comunidad indígena.docx

[C-I01]: hay una mamá que sale con leishmania y le sale el granito, hay una mamá que no sabe qué es, si es leishmaniasis, uno no sabe, a pesar de ir al hospital, hasta que ya aumente eso, ahí sí ya se preocupa, una mamá que no sabe [inaudible]

[INVESTIGADORA] entonces, a veces les sale el grano, pero no saben si es leishmaniasis o no, entonces esperan. Cuando ya la herida empieza a ponerse peor, entonces ¿ahí sí van?

[C-I01]: sí porque el año pasado quedó así y cuando pasó así, la mamá dijo que se quemó con...

[INVESTIGADORA] ¿ácido? [C-I01]: estaba fumando un cigarro y [inaudible] se quemó [inaudible] entonces ese Claudia

4:209 ¶ 898 – 902, in Comportamientos

5:33 ¶ 730 – 733, in Anexo 1 Transcripcion grupo focal de malaria y leishmaniasis con comunidad indígena.docx

[INVESTIGADORA] [C-I01], cuando tú trabajas con las mamás y les das recomendaciones, ¿las mamás hacen caso inmediatamente? [C-I01]: pues ahí normal [inaudible] hay unas que no.

[INVESTIGADORA] ¿y por qué no cumplirán? [C-I01]: pues a veces... por constancia, por lo económico.

[INVESTIGADORA] ¿tú les dices que vayan al médico pero les queda difícil?

[C-I01]: sí

4:212 ¶ 909 – 913, in Comportamientos

5:36 ¶ 624 – 627, in Anexo 1 Transcripcion grupo focal de malaria y leishmaniasis con comunidad indígena.docx

[INVESTIGADORA] ¿y lo usan todos o lo usan más lo niños? Bueno, don [C-I02] dice que de pronto los mayores de la antigua no tanto. ¿Los niños? [C-I07]: los niños más que todo

[C-I08]: más importante los niños

[C-I06]: todos tienen toldillo

[C-I04]: en donde nosotros no hay toldillos, pues así como nosotros, la costumbre, duermen sin toldillo

4:219 ¶ 939 – 940, in Comportamientos

5:65 ¶ 726, in Anexo 1 Transcripcion grupo focal de malaria y leishmaniasis con comunidad indígena.docx

[INVESTIGADORA] ¿y los extramurales, vacunadores, promotores que van son suficientes, van seguido? [C-I02]: no es suficiente porque el resguardo de nosotros es muy grande, es como 35 veredas por todo Pueblo Rico. Yo recuerdo 37, porque hay poquitos trabajadores, en este momento andan 3 vacunadores no más, antes éramos 8, cuando yo trabajaba. Siempre peleábamos ubicarnos en la veredas Chamí, hay unas veredas de 3 horas, hay unas veredas de 6 horas, entonces los trabajadores, son muy poquitos trabajadores, no alcanza. No alcanza con eso, llega a la cola y no alcanza a llegar hasta la cabecera, entonces [inaudible] siempre se queda...

4:224 ¶ 957 – 962, in Comportamientos

5:83 ¶ 555 – 559, in Anexo 1 Transcripcion grupo focal de malaria y leishmaniasis con comunidad indígena.docx

[INVESTIGADORA] bueno, listo, ¿ustedes creen que al indígena le gusta los talleres, las charlas que les enseñan?

[VARIAS VOCES] sí

[INVESTIGADORA] ¿y hay suficientes o faltan?

[VARIAS VOCES] faltan

[C-I02]: faltan muchas cosas. Para ser suficiente, nos falta mucho, jefe, para conocer al acento cómo arrancar... como disminuir esta enfermedad, pues nosotros seguimos mucho el taller porque aprendemos, porque sin orientación uno conoce, pero no conoce uno más allá cómo mejorar, me gustaría mucho más.

4:225 ¶ 963 – 964, in Comportamientos

5:84 ¶ 633, in Anexo 1 Transcripcion grupo focal de malaria y leishmaniasis con comunidad indígena.docx

[INVESTIGADORA] listo... el repelente, entonces el repelente, ¿el indígena usa el repelente? [C-I02]: el repelente el embera no utiliza, no sé porque el cuerito del embera no se cuida, yo no sé.

4:240 ¶ 1033 – 1045, in Comportamientos

5:136 ¶ 643 – 654, in Anexo 1 Transcripcion grupo focal de malaria y leishmaniasis con comunidad indígena.docx

INVESTIGADORA] ¿y alguna protección para los moscos? ¿para que no los piquen? [C-I08]: alguien le dijo que va a prender el fogón que tiene mucho humo, entonces dice que con ese humo le da miedo y se va

[INVESTIGADORA] ya, entonces ponen humo. [C-I04]: sí.

[C-I08]: eso con el humo se va a otro lado, si va a picar a niños, adultos...

[INVESTIGADORA] ah... ¿si ven que si hacen cosas? ya, entonces prenden el humo y eso sí saca los zancudos. ¿y dónde lo prenden? ¿en la cocina?

[C-I01]: por debajo de la casa

[INVESTIGADORA] ¿por debajo de la casa? Ah... ya, por debajo de la casa hacen eso... [C-I08]: pero si es una casa de esterillo o de pala, en material no es capaz, de humo dentro y cuando ese humo se alborota y ya los niños se pueden afectar.

[INVESTIGADORA] ¿eso es importante no? [C-I01]: cambia mucho, a veces va p'allá [inaudible]

[INVESTIGADORA] ¿y todas las noches hacen eso o cada cuánto? [C-I02]: cuando ventea.

[C-I04]: anteriormente no hacen eso...

[C-I02]: cuando echan ese humo hay que sacar todo los que están en la casa.

[inaudible]

[C-I04]: trae enfermedad.

4:254 ¶ 1098 – 1099, in Comportamientos

5:183 ¶ 436, in Anexo 1 Transcripcion grupo focal de malaria y leishmaniasis con comunidad indígena.docx

[C-I02]: [lengua embera] azufre, una cucharada de azufre y un pedacito de carbón bien quemadito, bien quemao' [lengua embera] corazón y un poquitico legudón que mata gusanos de ganado, preparaba eso, las 4 cositas, lavados bien lavadito [lengua embera] azufre, el carbón, el brea y un poquitico de legudón, eso hervía mucha agua, iba espeso... [lengua embera] esos medicamento los tumba todo [inaudible] en tres curacioncitas ya... [inaudible] se me olvidó esa práctica, seguí mucho medicamento del médico.

4:258 ¶ 1114 – 1115, in Comportamientos

5:201 ¶ 641, in Anexo 1 Transcripcion grupo focal de malaria y leishmaniasis con comunidad indígena.docx

[C-I02]: nosotros no tenemos repelente.

4:268 ¶ 1164 – 1181, in Comportamientos

6:26 ¶ 716 – 732, in Anexo 2 Transcripcion grupo focal de malaria y leishmaniasis con comunidad afrodescendiente.docx

[INVESTIGADORA] ¿y lo del toldillo que nos decías ahorita que antes lo usaban y ya no lo usan? ¿Creen que haya forma de volver a promover ese uso? [C-A04]: pueda...

[C-A06]: pueda que sí...

[C-A02]: el problema ahorita es como estamos con ese apogeo de la malaria...

[C-A06]: con estos calores quién lo usa...

[C-A02]: resulta que este año la malaria se ha disparado pero horrible, yo creo que si ente momento llegan a darle charla sobre la necesidad de los toldillos, no venga... con un 60% que lo utilice estamos ganando. En la comunidad con un 60% que lo utilice estamos ganando, entonces yo creería que puede funcionar, o sea...

[C-A04]: es que ahora está muy alborotado.

[C-A06]: funciona, pero que no le echen ese químico...

[C-A02]: es que tiene que tener el químico, eso es lo que repele los mosquitos.

[C-A06]: hay unos toldillos demasiado, demasiado, que no pasa, que el zancudo no pasa, pero la gente aquí dejó de utilizar eso, más que todo por ese problema... porque es que había personas...

[C-A01]: da alergia

[C-A02]: da alergia, amanecíamos con la cara hinchada, entonces... no.

[C-A02]: ¿y sí utilizaban el protocolo correspondiente para usar el toldillo? De sacarlo de la bolsa, ponerlo un rato a airear...

[C-A06]: incluso nosotros lo lavábamos

[C-A02]: no, es que ese es el problema, si se lava va activar más ese...

[C-A06]: es que yo lo lavé, ¿sabe por qué lo lavé?

[C-A02]: es que hay químicos que no son compatibles con el agua...

[C-A06]: ¿pero sabe yo por qué lo lavé? Porque los que los intalaron así, fueron los que estuvieron más enfermo y fueron al hospital, entonces dije, vea lo voy a lavar, incluso lo eché en cloro, en agua a ver si sí, incluso lo eché en agua hervida, ¿quién dijo? Me dieron 3, todos 3 los boté, yo sí lo reconozco, los boté y en mi casa... ¿sabe algo? Mi hija, mi hija por ejemplo tuvo un bebé, que día fui a punto rojo y le dije al señor que me vendiera un toldillo, pero le pregunté pues que no, un toldillo que no traiga nada, un toldillo normal o sino no se lo hubiera puesto tampoco.

4:272 ¶ 1204 – 1217, in Comportamientos

6:37 ¶ 467 – 481, in Anexo 2 Transcripcion grupo focal de malaria y leishmaniasis con comunidad afrodescendiente.docx

pero qué tanto la gente usa el toldillo? Y lo que... [C-A01]: muy poco, la verdad es esa, muy poco, la verdad es esa...

[C-A06]: yo creo que hasta menos

[C-A02]: yo creo que un 40%, de un 100% de la comunidad un 40.

[INVESTIGADORA] entonces vamos a hacer un semáforo, entonces es rojo... rosado, si en serio eso no sirve aquí, nadie lo usa, amarillo si hay una cantidad considerable, un 40% que lo usa y verde si sí funciona y la mayoría de la gente...

[C-A06]: más que todo los reparten en las comunidades, ¿cierto? Porque aquí en Santa se hizo pero la gente después de eso hicieron un recorrido para ver si la gente lo estaba usando y se dieron cuenta que no...

[C-A04]: es que esos toldillos los dejaron de utilizar desde que usted dijo...

[C-A06]: desde eso sí, porque mucha gente...

[C-A01]: eso le picaba a uno la cara, le daba alergia.

[C-A02]: pero eso se utilizaba mucho [inaudible]

[C-A06]: en mi caso, nosotros lo lavamos y pensábamos que lavándolo... porque ellos decían que no podíamos lavar y después de que lo lavamos, al otro día amanecimos con un brote en la cara, hinchada, entonces no...

[INVESTIGADORA] ¿entonces la gente no lo usa?

[C-A02]: en el momento, no

[C-A06]: es muy poco, yo pues no lo utilizo.

4:274 ¶ 1222 – 1224, in Comportamientos

6:44 ¶ 620 – 621, in Anexo 2 Transcripcion grupo focal de malaria y leishmaniasis con comunidad afrodescendiente.docx

[INVESTIGADORA] y esas cosas que influyen, decías que no les daban cita rápido, que les toca esperar todo el día en el hospital, ¿qué otras cosas influyen para que la gente no vaya, no quiera ir al hospital?

[C-A01]: pues por lo general les da pereza por lo que yo les dije, les toca esperar mucho o que no consiguen citas y entonces eso lleva a que la gente se automedique y que vaya a la farmacia, que tengan que ir a la farmacia.

4:276 ¶ 1236 – 1237, in Comportamientos

6:54 ¶ 87, in Anexo 2 Transcripcion grupo focal de malaria y leishmaniasis con comunidad afrodescendiente.docx

Pues en mi caso, en mi casa no se puede usar toldillo, una vez nos repartieron unos toldillos los cual los lavamos, pues nos decían que no se podía lavar, pero más, sin embargo, los lavamos y al otro día amanecimos con la cara hinchada, alborotada, o sea, en mi casa no los utilizamos... en mi casa habemos seis, siete personas ahí pues y hasta ahora únicamente le dio a mi hijo, de resto a nadie de mi casa le ha dado. Porque incluso en el tanque que mantenemos el agua, no me gusta mantenerlo lleno, pero sí conseguimos pastillas de cloro y cuando lo llenamos le echamos esa pastilla, entonces por lo tanto, a veces debajo se mantienen los zancudos, pero entonces nosotros mantenemos pendiente de lavarlo con mucho cloro y después sale esa pastilla.

4:280 ¶ 1250 – 1254, in Comportamientos

6:72 ¶ 321 – 327, in Anexo 2 Transcripcion grupo focal de malaria y leishmaniasis con comunidad afrodescendiente.docx

[C-A06]: cómo es que se llama eso? Que uno le echa alcohol, como un polvito y uno le echa...

[C-A01]: ah, el salicílico con alcohol

[C-A06]: el salicílico con alcohol también es super bueno... para la leishmaniasis, para tratarlo

[FACILITADOR] ¿cómo se escribe? [C-A06]: salicílico con alcohol, ese también es super bueno para [inaudible]

4:282 ¶ 1273 – 1284, in Comportamientos

6:80 ¶ 550 – 560, in Anexo 2 Transcripcion grupo focal de malaria y leishmaniasis con comunidad afrodescendiente.docx

[C-A08]: tenga su amarillo

[INVESTIGADORA] ¿y usar ropa mangalarga y pantalón largo, ¿sí se hace?

[C-A06]: amarillo

[C-A04]: pues la mayoría de aquí los que van para campo, siempre... y aquí con este calor yo he visto mucha gente con su camisa manga larga

[C-A01]: un shortcito sí

[C-A04]: algo deportivo prácticamente

[INVESTIGADORA] más o menos, sí

x: lo único manga larga que consiguen en mi armario es un saco.

[C-A04]: y eso que cuando llueve hace calor

[C-A01]: quema de eucalipto, no muy poco, eso...

[C-A07]: mas bien uno le prende el ventilador y eso también los ahuyenta así...

4:290 ¶ 1308 – 1311, in Comportamientos

6:104 ¶ 482 – 484, in Anexo 2 Transcripcion grupo focal de malaria y leishmaniasis con comunidad afrodescendiente.docx

[INVESTIGADORA] ¿y esta primera de recoger hojas? [C-A02]: ¿eso es como limpiar alrededor de la casa? Sí, yo creo que un 40, un 50% aquí mantiene sus predios limpios, al rededor, pues con respecto...

[C-A06]: hay unos, porque hay otros que nos toca a nosotros que limpiar.

[C-A02]: por eso un 50% mantiene sus casas limpias

4:292 ¶ 1315 – 1318, in Comportamientos

6:114 ¶ 401 – 403, in Anexo 2 Transcripcion grupo focal de malaria y leishmaniasis con comunidad afrodescendiente.docx

¿qué otras cosas se pueden hacer o qué cosas se hacen en las comunidades? Tanto para malaria como para leishmaniasis...

[C-A06]: en mi caso yo quemo las cubetas del huevo

[C-A01]: también quemar basura, cuando hay muchos zancudos

4:299 ¶ 1342 – 1358, in Comportamientos

6:141 ¶ 488 – 503, in Anexo 2 Transcripcion grupo focal de malaria y leishmaniasis con comunidad afrodescendiente.docx

[INVESTIGADORA] bueno, ¿y el repelente? ¿qué tanto se usa?

[C-A06]: yo sí lo utilizo, pero cuando salgo a campo, de resto en mi casa no.

[C-A04]: En mi casa sí a veces lo usamos pero para la niña, como está haciendo tanto calor.

[C-A02]: aquí en la población de aquí, no...

[C-A06]: pero mire que fuera del repelente hay otro medicamento que se llama el Caladryl, mi hija sí lo utiliza, es transparente, ella siempre antes de acostarse... ella antes de acostarse se empapa todo porque ella duerme hasta sin ropa, entonces ella se unta eso...

[C-A07]: ¿ese caladryl cómo es?

[C-A06]: es super...

[inaudible]

[INVESTIGADORA] pero entonces el caladryl... bueno, el repelente en general sí es rojo o es más o menos?

[C-A02]: es rojo

[INVESTIGADORA] bueno... ¿las charlas educativas qué tanto pasa? [C-A06]: ¿qué significa este rojo?

[C-A01]: el repelente, que es muy poco

[INVESTIGADORA] rojo es que no se usa... [C-A03]: pero el repelente sí no debería ser rojo

[C-A06]: porque hay muchas mamás que lo utilizan más que todo en sus hijos

[C-A01]: entonces amarillo

[INVESTIGADORA] ¿qué? [C-A01]: para el repelente

4:301 ¶ 1364 – 1371, in Comportamientos

6:147 ¶ 543 – 549, in Anexo 2 Transcripcion grupo focal de malaria y leishmaniasis con comunidad afrodescendiente.docx

[INVESTIGADORA] ¿en quemar basuras y hojas? ¿cómo lo ven ustedes? ¿se hace mucho? ¿Se hace poco?

[C-A08]: amarillo... ya tiene

[C-A06]: amarillo porque es más o menos.... ya tiene amarillo ese...

[C-A01]: entonces este coloquémoslo con este, porque este es recolectar y este es quemar

[INVESTIGADORA] sí, ese es recoger y ese es quemar

[C-A01]: es que son dos cosas diferentes

[INVESTIGADORA] entonces amarillo

4:302 ¶ 1372 – 1373, in Comportamientos

6:149 ¶ 504, in Anexo 2 Transcripcion grupo focal de malaria y leishmaniasis con comunidad afrodescendiente.docx

[INVESTIGADORA] amarillo para el repelente... ¿y charlas educativas? ¿sí se hace? ¿es común? [C-A02]: sí se hace, no a fondo como debería ser, pero sí se hace, así como mencionaba el compañero ahorita, que muchos vienen acá a dictar charla y también a legalizar cosas, pero sí se hace, sino que si... si uno en el momento que vienen a dar charla, nosotros como los propietarios del problema le exigimos a ellos que nos aclaren, que nos digan las cosas bien... pero nosotros, ellos vienen a legalizar y uno por irse con el afán, ah bueno, lo poquito que dé y se va para su casa, entonces es culpa de parte y parte.

4:303 ¶ 1374 – 1385, in Comportamientos

6:150 ¶ 433 – 443, in Anexo 2 Transcripcion grupo focal de malaria y leishmaniasis con comunidad afrodescendiente.docx

[INVESTIGADORA] y en leishmaniasis, ¿es normal que se hagan el tratamiento o la mayoría de gente...?

[C-A08]: eso es muy duro

[C-A01]: hay gente que no... vea yo conocí el caso de un muchacho de Ágüita que estaba... y ya uno que le daba pena ir al hospital porque, uno, se había dejado avanzar eso y le iban a echar cantaleta y, dos, porque ya sabía lo que le esperaba con las inyecciones, o sea que, se tapaba

[C-A08]: sino que mucha gente evitaba las inyecciones era porque eran muchas, como era del peso suyo, así mismo le contaban, pero igualmente como todo va cambiando, son como dos dosis...

[C-A01]: ya es una dosis más concentrada...

[C-A08]: o también, si el hospital tiene, también se la quema, también lo curan.

[C-A06]: pero también ahora ya no se está inyectando directamente, sino por suero, algunas que es por suero y otra que la inyectan

[INVESTIGADORA] ¿intravenoso? [C-A08]: Sí

[C-A06]: sí

[C-A01]: sino que la gente le tiene mucho miedo a las inyecciones

[C-A06]: en mi caso me mandaron 65 y me pusieron la primera ese mismo día y no más y no más, al hospital no voy... que con el simple hecho de ver que me aplicaron dije yo esto no lo aguanto y 65 inyecciones.... no

4:306 ¶ 1388 – 1391, in Comportamientos

6:161 ¶ 523 – 525, in Anexo 2 Transcripcion grupo focal de malaria y leishmaniasis con comunidad afrodescendiente.docx

[C-A01]: bueno, nos quedamos en las charlas educativas.

[INVESTIGADORA] ¿se hace mucho, poco, cómo lo ven ustedes?

[C-A02]: pongámosle amarillo

4:309 ¶ 1398 – 1406, in Comportamientos

6:166 ¶ 591 – 598, in Anexo 2 Transcripcion grupo focal de malaria y leishmaniasis con comunidad afrodescendiente.docx

[C-A01]: ¿la leishmaniasis?

[INVESTIGADORA] en tratamiento

[C-A01]: no, la gente sí se lo hace

[C-A08]: la gente sí se lo hace...

[C-A06]: o sea, la gente se lo hace, pero hay unos... el problema es que ahora el tratamiento realmente no está funcionando como funcionaba, entonces la gente...

[C-A01]: es que todos los organismos son diferente, entonces a unos les sirve y a otros no

[C-A08]: se va a una fiesta buena y hasta ahí llegó el tratamiento

[C-A06]: o sea, que ahí va el amarillo, así es.

4:312 ¶ 1419 – 1421, in Comportamientos

6:181 ¶ 426 – 427, in Anexo 2 Transcripcion grupo focal de malaria y leishmaniasis con comunidad afrodescendiente.docx

[INVESTIGADORA] y la gente cuando se ve las lesioncitas de la leishmaniasis, ¿se va y se hace la prueba? [C-A07]: algunos porque a veces no...

[C-A01]: algunos empiezan a hacer remedios caseros, inician su tratamiento en casa

4:316 ¶ 1438 – 1442, in Comportamientos

6:195 ¶ 602 – 605, in Anexo 2 Transcripcion grupo focal de malaria y leishmaniasis con comunidad afrodescendiente.docx

[INVESTIGADORA] y todo estos de jabón rey, limón pajarillo…

[C-A08]: amarillo

[C-A06]: no, verde, cómo le vas a poner amarillo, pórtese serio

[C-A08]: verde... son los propios para automedicarse con hierbas raras.

4:318 ¶ 1455 – 1459, in Comportamientos

6:202 ¶ 315 – 318, in Anexo 2 Transcripcion grupo focal de malaria y leishmaniasis con comunidad afrodescendiente.docx

[C-A06]: ¿también sabe qué? El tabaco también, lo mastican y se echan

[C-A06]: ¿sabe qué también están utilizando para eso?

[C-A08]: también usan mucho eucalipto [inaudible]

[C-A06]: el cloro, el cloro también lo usan para le leishmaniasis.

4:320 ¶ 1468 – 1469, in Comportamientos

6:210 ¶ 175, in Anexo 2 Transcripcion grupo focal de malaria y leishmaniasis con comunidad afrodescendiente.docx

[C-A02]: ¿está cambiando ahorita por qué? porque antes, por lo mismo que usted decía mandaban yo no sé cuántas inyecciones y yo no sé, les daba pereza ir a aplicárselas. Ahorita qué hacen, en una sola inyección o dos o tres inyecciones concentran esa fórmula para que apenas sean dos o tres inyecciones

9:55 ¶ 179 – 182, in Corresponsabilidad

4:381 ¶ 43 – 45, in INSTITUCIONES ML & LC.docx

¿El indígena está acostumbrado a usar repelente?

[I-M01]: No. Ellos no lo utilizan.

[I-A03]: porque dicen que no, porque... es que sí, es amargo. Porque es que son muy amargos, que es eso me molesta, pero igual antes de entregarlos, por eso se hizo... porque era que los mismos gobernadores pedían "venga, por qué no nos dan repelente", entonces... De pronto algunos utilizan, otros no lo utilizan...

○ Vacios de conocimiento_LC

31 Citas:

1:62 ¶ 194 – 196, in Conocimiento comunidad

5:5 ¶ 306 – 307, in Anexo 1 Transcripcion grupo focal de malaria y leishmaniasis con comunidad indígena.docx

[INVESTIGADORA] ¿Qué pasa con las mujeres embarazadas cuando tienen leishmaniasis? [C-I07]: hacen tratamiento

[C-I09]: no, no, es como de cuidado. Primero el diagnóstico, sí. Porque esta semana se fue una señora con malaria, la hospitalizaron 3 días y no le mandaron tratamiento, porque es muy delicado porque el medicamento es muy fuerte, entonces como que le administraron un medicamento más diferente que es el medicamento de malaria, el tratamiento de malaria, ella estuvo varios días hospitalizada, entonces como es... eso es como prevención, ¿será? Sí, porque no le dieron medicamento.

1:68 ¶ 216 – 217, in Conocimiento comunidad

5:32 ¶ 130, in Anexo 1 Transcripcion grupo focal de malaria y leishmaniasis con comunidad indígena.docx

[C-I03]: [C-I09] no entendí y puedo preguntar, dónde es el mico que transmite una enfermedad... leishmaniasis, ahí no entiendo y quería que me explique con la lengua de él porque a veces uno entiende, pero igual...

1:77 ¶ 246 – 247, in Conocimiento comunidad

5:57 ¶ 693, in Anexo 1 Transcripcion grupo focal de malaria y leishmaniasis con comunidad indígena.docx

[INVESTIGADORA] saben que es mosquito, pero ¿es el mismo mosco de la leishmaniasis, de la piña, que de la leishmaniasis? [C-I07]: hay varios mosquitos que son de leishmaniasis, que son la malaria, de cuál de los dos cuál es de leishmaniasis o de la malaria.

1:83 ¶ 261 – 263, in Conocimiento comunidad

5:79 ¶ 127 – 128, in Anexo 1 Transcripcion grupo focal de malaria y leishmaniasis con comunidad indígena.docx

[C-I09]: este dibujo es caso en... cómo se transmite leishmaniasis. La leishmaniasis, hay muchas formas de transmitir la leishmaniasis, especialmente yo lo he escuchado y por ahí siempre le han dicho, se transmite por medio de garrapatas y unos piojitos que... el piojito se transmite el mico. Las personas cuando van al monte a trabajar, a realizar su labor diario en la agricultura, o algunos indígenas por acá, como acá hay muchas montañas, salen a trabajar al monte, no van cubiertos de pronto con manga largas o de pronto con camisitas así con manga corta, entonces por allá en el monte, el mico tiene un piojo que especialmente se va a transmitir, el piojito del mico sale y de pronto si el agricultor está en el monte trabajando, le pega, llega allá y le pica a uno, a la persona, entonces el mico tiene un piojo especialmente que puede transmitir la leishmaniasis y se junta bastante, cuando el mico pasa por ahí, deja el piojo por ahí, en los árboles, en las matas, entonces... la persona que va a laborar, su trabajo diario, va y pica a la persona, el piojito de monte o la garrapatita y entonces ahí es donde le causa la leishmaniasis y para poder uno proteger eso, pues tiene que ir con manga larga a trabajar para que de pronto no sean picaduras de mosco, de la garrapata, especialmente he escuchado eso.

[INVESTIGADORA] entonces tu pones el lago, basura... [C-I09]: digamos que ahí en la basura, también hay un mosco también que transmite también la leishmaniasis, no solo la garrapata que también es la basura, en las orillas de los lagos, en las orillas de los ríos, donde está estancada el agua en la orillita, también producen una garrapata que transmite la leishmaniasis, entonces estancados en el agua, también en la orillita está el montecito, ellos pueden criar unas garrapatitas ahí y la persona va a la orilla donde está el estanque y la persona puede picar y puede causar la leishmaniasis, igualmente las basuras, esa misma garrapatica [se] produce en las basuras igual que en donde está el agua estancada, esos son

1:84 ¶ 264 – 265, in Conocimiento comunidad

5:89 ¶ 386, in Anexo 1 Transcripcion grupo focal de malaria y leishmaniasis con comunidad indígena.docx

[C-I09]: sí como mirando la herida porque hay sitios que uno no lo puede ver, qué tal le de a uno en la espalda, entonces es muy difícil... él está es como mirando eso, diagnosticando solito la lesión.

1:91 ¶ 285 – 286, in Conocimiento comunidad

5:113 ¶ 139, in Anexo 1 Transcripcion grupo focal de malaria y leishmaniasis con comunidad indígena.docx

[C-I02]: doctora, agrego un poquitico ahí, cuando él tomo... el mosquito puede picar a un serpiente, como serpiente tiene un veneno y ese veneno muy delicado y el mosquito está chupado la sangre de una culebra, pica a una persona, puede [inaudible] y eso es duro para curar de culebra.

1:92 ¶ 289 – 291, in Conocimiento comunidad

[C-I02]: y en el verano, peor.

[C-I09]: sí.

[C-I02]: estos tiempo está regado en casi todas las veredas.

1:96 ¶ 294 – 299, in Conocimiento comunidad

5:122 ¶ 482 – 486, in Anexo 1 Transcripcion grupo focal de malaria y leishmaniasis con comunidad indígena.docx

[INVESTIGADORA] última pregunta, cómo es el tratamiento de leishmaniasis.

[C-I09]: el tratamiento de leishmaniasis ahora en este momento es hospitalario, tiene que acudir la mamá con el niño o con el paciente cada tres días porque ella es intravenosos.

[INVESTIGADORA] o sea, es una inyección. [C-I09]: cada 3 días.

[INVESTIGADORA] ¿por cuánto tiempo? [C-I09]: eso es como... se le entregan como unas 4 ampollas, pero ahora es más diferente 4 ampollas entonces lo diluyen en solución salina, entonces tiene que venirse 4 veces, como son 4 ampollas, cada 3 días.

[INVESTIGADORA] ya no es todos los días, no tiene que ir todos los días. [C-I09]: ya no es todos los días, eso ha cambiado mucho y también llegó unas pastas que se pueden mandar para la casa para que el paciente esté tratando en la casa.

1:97 ¶ 300 – 302, in Conocimiento comunidad

5:129 ¶ 476 – 477, in Anexo 1 Transcripcion grupo focal de malaria y leishmaniasis con comunidad indígena.docx

¿ustedes lo conocen, el tratamiento? [C-I08]: de esa parte no tengo idea.

[C-I03]: como el tratamiento de la leishmaniasis

1:101 ¶ 322 – 323, in Conocimiento comunidad

5:144 ¶ 366, in Anexo 1 Transcripcion grupo focal de malaria y leishmaniasis con comunidad indígena.docx

[C-I03]: tengo una pregunta también, es que no entiendo, un ejemplo si... yo creo que hay dos animalitos que se terminen la enfermedad, un ejemplo, creo que los mosquitos tienen dos mosquitos creo pues porque yo pienso que solamente hay una mosca que produce la enfermedad, al leishmaniasis o puede ser el paludismo... entonces eso quiero saber cómo previenen la enfermedad si hay dos animalitos o hay tres...

1:107 ¶ 346 – 347, in Conocimiento comunidad

5:163 ¶ 95, in Anexo 1 Transcripcion grupo focal de malaria y leishmaniasis con comunidad indígena.docx

[C-I07]: pues acá les presento el dibujo lo que hice, yo de parte de leishmaniasis no tengo ni idea, nunca me ha dado ni a mis hijos, pero esta es mi casa.

1:112 ¶ 359, in Conocimiento comunidad

[C-I02]: el mosquito palomilla mantiene en el monte en un capote [lengua embera], hay dos cosas: te puede picar en el día o en la noche [inaudible] y también si las casitas, el embera nunca en casita hace ventanas ni puertas, entonces las casitas, sin puerta abierta el mosquito siempre viene a buscar donde hay caliente [lengua embera]. Como no hay puerta, allá donde... [lengua embera] coge la oreja, coge la nariz, ahí quedó contaminado, en cualquier cuerpo donde llegue hasta el más [lengua embera] es delicado, come la carita, come la nariz, eso come donde llegue. ¿Y cómo se transmite? Se va a transmitir [lengua embera

1:118 ¶ 385 – 386, in Conocimiento comunidad

5:192 ¶ 138, in Anexo 1 Transcripcion grupo focal de malaria y leishmaniasis con comunidad indígena.docx

[C-I06]: esto es... la enfermedad se puede transmitir con unos mosquitos, es pues el significado, en estos como casas pues, en estos... términos acá, condiciones, se puede producir la enfermedad porque está lleno de basuras, tiene un pozo con mucha basura, con un lago pues... que hayan dejado mucho tiempo, por ejemplo, ya se pueden producir los mosquitos y tiene pues... ya pueden producir al ser humano con unas picazones que les puede transmitir la leishmaniasis. La leishmaniasis puede transmitir porque los mosquitos no pican solo como al ser humano, pueden picar a varios animales que no conocemos, puede ser a una serpiente, a otro... muchos animales vienen y pican al ser humano ya no puede producir lo que es la leishmaniasis.

1:120 ¶ 384, in Conocimiento comunidad

[INVESTIGADORA] pero estamos hablando de leishmaniasis, ¿tú hiciste de leishmaniasis? [C-I01]: Sí, de leishmaniasis por la basura también, lo que tanto como la botella o las llantas y también...

1:123 ¶ 389 – 393, in Conocimiento comunidad

5:199 ¶ 115 – 118, in Anexo 1 Transcripcion grupo focal de malaria y leishmaniasis con comunidad indígena.docx

[C-I04]: bueno, cómo se produce la leishmaniasis, ese se produce la leishmaniasis, ese tiene un atajo, porque está lleno de mosquitos....

[INVESTIGADORA] ¿eso es qué dijiste? [C-I04]: como se produce la leishmaniasis.

[INVESTIGADORA] ¿cómo se produce la leishmaniasis? [C-I04]: estos son los tanques y esos tanques llenos de mosquitos, llega el mosquito, llega el tanque, se va a esas casas donde están dormidos, ya van casi llegando para picar a esas personitas y también, esos mosquitos son los que están acá, los de leishmaniasis y esos están en cicatrices, donde está el mosquito, el mosquito está lleno de sangre.

[INVESTIGADORA] ah, por eso está rojo, la herida es roja y el mosquito está lleno de sangre. [C-I04]: y acá lo picó acá y... está lleno de sangre.

1:126 ¶ 394 – 398, in Conocimiento comunidad

5:206 ¶ 106 – 109, in Anexo 1 Transcripcion grupo focal de malaria y leishmaniasis con comunidad indígena.docx

y cuando pica al niño, ahí se queda como un granito y de ahí sale todo el grano grande, ya comienza de... llevar todo y ya no...

[INVESTIGADORA] ¿se crea un qué [C-I03]? [C-I03]: un granito, sí, eso se pican y de ahí queda granito rojo y de ahí se va todo... y de ahí se cría esa mosca en el tanque o en... una quebradita pequeña ahí también crían que la mosca y eso ya tiene como gusanito y ya eso va creciendo como... este zancudo.

[INVESTIGADORA] ¿y por qué la dibujaste de blanco? [C-I03]: porque el blanco, hay dos formas de mosca, palomilla, el palomilla y el mosca negra.

[INVESTIGADORA] ah, ya. Muestrales el dibujo a tus compañeros. Ahí [C-I03] dibuja la cicatriz en la cara y en la pierna. Ya... y le... [C-I03]: la quebrada... y el tanque...

1:128 ¶ 402 – 403, in Conocimiento comunidad

5:209 ¶ 472, in Anexo 1 Transcripcion grupo focal de malaria y leishmaniasis con comunidad indígena.docx

[C-I09]: se descubrieron muchos casos de leishmaniasis, casos diferentes, es que hay leishmaniasis... hay muchas clases de leishmaniasis, hay unos que esas

heriditas no se abren, pero se ponen unas burbujitas ahí, duras, y como encarnizado ahí, entonces a eso pues también toman una muestra y algunos no saben cómo tomar muestra y dicen que no es leishmaniasis, pero mentiras que dentro está...

1:131 ¶ 408 – 409, in Conocimiento comunidad

6:24 ¶ 145, in Anexo 2 Transcripcion grupo focal de malaria y leishmaniasis con comunidad afrodescendiente.docx

[INVESTIGADORA] bueno, ya hemos hablado un poquito, pero qué signos y síntomas de la leishmaniasis conocen... [C-A06]: a mi me daba escalofrío, dolor de cabeza una vez que otra.

1:134 ¶ 412 – 417, in Conocimiento comunidad

6:31 ¶ 38 – 42, in Anexo 2 Transcripcion grupo focal de malaria y leishmaniasis con comunidad afrodescendiente.docx

[C-A06]: de eso no hay que dejarle coger ventaja, porque el que es mojado es muy maluco, porque él por ejemplo le da a usted aquí y cuando usted menos piensa le salió aquí. Va brincando... pero el que es seco siempre se queda en el punto.

[INVESTIGADORA] o sea, la diferencia es que el seco no se...

[C-A06]: no se esparce... no le sale en otro lado y así

[INVESTIGADORA] ¿pero las heridas se ven igual en los dos? [C-A06]. sí, hay unas que son más grandes, otras que... y diario está echándole agua...

[C-A03]: el mojao' y el seco es como una moneda y se va creciendo, se va creciendo, pero ahí en la misma parte.

1:142 ¶ 434 – 435, in Conocimiento comunidad

6:67 ¶ 119, in Anexo 2 Transcripcion grupo focal de malaria y leishmaniasis con comunidad afrodescendiente.docx

[C-A06]: o sea, de lo mismo de [C-A02], yo digo... si fuera...aunque yo un día pregunté, yo digo que si eso fuera un huevito, digo pues yo así, no digo que no sea así, sino que un día yo le pregunté a la doctora Leidy y que no, que eso no se trataba de un huevo, sino que los zancudos muchas veces picaban a un animal, o sea, se sentaban en un animal... es lo mismo que la leishmaniasis, por ejemplo, la leishmaniasis se le sienta a un verrugoso, o a una serpiente así y por eso es que ahí de unas y unas, entonces me decía que era lo mismo, que era algo contagioso, y que al picarle a uno, eso se le iba a lo vasos sanguíneos y etc... porque yo digo que si le mandan a uno un medicamento de leishmaniasis para matar todo eso..

1:145 ¶ 441 – 442, in Conocimiento comunidad

6:78 ¶ 146, in Anexo 2 Transcripcion grupo focal de malaria y leishmaniasis con comunidad afrodescendiente.docx

[INVESTIGADORA] ¿pero cómo se da cuenta uno que uno tiene leishmaniasis? [C-A06]: como le digo, o sea, es que hay uno que a usted le puede salir uno aquí, otro aquí, como en forma de una moneda, pero muchas veces como dice ella puede ser un hongo.

1:152 ¶ 455 – 456, in Conocimiento comunidad

6:107 ¶ 144, in Anexo 2 Transcripcion grupo focal de malaria y leishmaniasis con comunidad afrodescendiente.docx

[C-A02]: pues lo que tengo... lo que he escuchado, me han enseñado que la leishmaniasis es una enfermedad endémica o que es producida o transmitida por un insecto, ya sea la palomilla que dice la compañera o hay otra que es una mosca que la puede transmitir... eh... inclusive hasta el mismo gato puede hacer que le de leishmaniasis porque resulta que los gatos por su naturaleza ellos tienen la leishmaniasis, cuando a ellos les brota ese pus [inaudible] ese pus puede ser un zancudito, [inaudible] la verdad, la verdad pues con respecto a que si es palomilla, algunos dicen que sí es palomilla, eso es producida por verrugoso, tampoco he tenido conocimiento, pero sí sé que es una enfermedad endémica y que lo más ideal es al momento de que aparezca ir al hospital que hagan la prueba, porque se pueden confundir con las úlceras adiposas

1:157 ¶ 473 – 474, in Conocimiento comunidad

6:127 ¶ 289 – 290, in Anexo 2 Transcripcion grupo focal de malaria y leishmaniasis con comunidad afrodescendiente.docx

[INVESTIGADORA] bueno y acá, ¿ustedes creen que estas acciones de recoger las basuras, las aguas estancadas, no sirven para la leishmaniasis? [C-A01]: también porque se trata de un mosquito

1:159 ¶ 480 – 481, in Conocimiento comunidad

6:136 ¶ 159 – 160, in Anexo 2 Transcripcion grupo focal de malaria y leishmaniasis con comunidad afrodescendiente.docx

[INVESTIGADORA] ¿y cómo se previene la leishmaniasis? [C-A03]: ahí sí no sé

1:164 ¶ 510 – 511, in Conocimiento comunidad

6:156 ¶ 148, in Anexo 2 Transcripcion grupo focal de malaria y leishmaniasis con comunidad afrodescendiente.docx

[C-A06]: en mi caso no fue así, en mi caso yo tenía dos punticos, por eso mi hija me preguntó que si me había quemado, yo me miré y de una vez supe que tenía leishmaniasis y por lo que... yo sé porque cuando yo me toqué, sentí que algo... como cuando le pica algo a uno, entonces yo no le paré bolas a eso y sí, supe que era leishmaniasis, voy al hospital, me hicieron la muestra, pero yo ya sabía que era leishmaniasis, pero el otro... parece como la moneda, ese tiende a ser más grande, hay que rezarle las benditas a uno. Y el que es mojado, ese es el que lo tiene aquí y

lo puede reventar por otro lado, pero ese no. Pues en mi caso yo nunca sentí fiebre, dolor de cabeza, nada.

1:168 ¶ 523 – 524, in Conocimiento comunidad

6:171 ¶ 133, in Anexo 2 Transcripcion grupo focal de malaria y leishmaniasis con comunidad afrodescendiente.docx

[INVESTIGADORA] entonces, ¿qué es y cómo se transmite la leishmaniasis [C-A03]: yo tengo entendido que hay como una palomilla, una mosquita así en la noche le pica a uno [inaudible] y esa tremenda roncha que deja y me han dicho que de esa palomilla era la leishmaniasis, no sé... me gustaría saber si es cierto o no, pero eso me han dicho. La verdad es una enfermedad... yo he visto personas que ay, qué pesar y más pesar me da saber todas las inyecciones que les tienen que aplicar, por dios, creo que quedan sin nalga para sentarse.

1:172 ¶ 531 – 532, in Conocimiento comunidad

94 ¶ 167, in Anexo 2 Transcripcion grupo focal de malaria y leishmaniasis con comunidad afrodescendiente.docx

[C-A06]: ¿es que sabe qué es lo que pasa? Es que el medicamento que están utilizando los médicos, o sea, estas inyecciones ya no funcionan, las inyecciones buenas, antes habían unas inyecciones que me cuenta mi papá porque a él le dio como 5 veces leishmaniasis, que habían unas inyecciones super buenas que les aplicaban unas solas y del poquitico que les quedaban, se lavaban y se las untaban ahí y era con eso, pero las inyecciones, imagínese que antes mandaba una, ahora si usted pesa 100 kg, le mandan 100 inyecciones.

1:173 ¶ 533 – 534, in Conocimiento comunidad

6:199 ¶ 153 – 158, in Anexo 2 Transcripcion grupo focal de malaria y leishmaniasis con comunidad afrodescendiente.docx

[C-A02]: el principal síntoma es como el salpullido, esa es como rojizo ese salpullido y que sean varios puntos en la misma parte puede ser la leishmaniasis, [inaudible] pero al ver... y pica, da picasón...

1:175 ¶ 540 – 543, in Conocimiento comunidad

6:200 ¶ 95 – 97, in Anexo 2 Transcripcion grupo focal de malaria y leishmaniasis con comunidad afrodescendiente.docx

[C-A06]: en mi caso, la leishmaniasis yo no creo en ese remedio, aunque hay unos que sí, otros que no... porque las inyecciones buenas ya no vienen

[C-A03]: si vienen ya vienen falsificadas.

[C-A06]: no, ya no... esas inyecciones ya no están funcionando.

1:181 ¶ 550 – 555, in Conocimiento comunidad

6:214 ¶ 168 – 172, in Anexo 2 Transcripcion grupo focal de malaria y leishmaniasis con comunidad afrodescendiente.docx

[INVESTIGADORA] ¿solo mandan las inyecciones o también tiene pastas?

[C-A06]: aquí que yo sepa inyecciones no más

[C-A08]: hace aproximadamente 1 año, eso ya cambió, aproximadamente. Aproximadamente un año ya cambió en el territorio.

[C-A04]: ¿el medicamento?

[C-A08]: la forma de tratar la leishmaniasis.

1:182 ¶ 556 – 557, in Conocimiento comunidad

6:221 ¶ 142, in Anexo 2 Transcripcion grupo focal de malaria y leishmaniasis con comunidad afrodescendiente.docx

[INVESTIGADORA] bueno, pero antes de que sigamos, volvemos un poquito a la pregunta inicial que era [inaudible] que ya nos habían dicho que la palomilla [inaudible] [C-A06]: pues hay unos que le decimos chácara y hay otros que le decimos... es parecido a un grajo, pero es muy pequeñito.

MALARIA

○ Barreras de aptitud_ML

43 Citas:

8:28 ¶ 19 – 20, in Salud

2:81 ¶ 154, in Indigena TB.docx

[C-I05]: No, pero hay unos también si no, también muy largo la fecha de la cita. La dan muy largo. Yo la pedí el mes de abril y me llegó el diez de junio. ¿Y a eso uno enfermo qué? Hasta la fecha esperando qué [inaudible]. Yo he ido siempre a donde el médico y realmente el paciente lleva enfermo, con esa cita no se cura. ¿Qué se va a curar? Se muere. [inaudible] Que allá uno hace atender, pero, pero, metiendo candela y eso es muy maluco, pero toca así, porque si no se muere la familia de uno, mi hermanito murió así. Igualito. Vino a la consulta. No, usted que está bien, más o menitos, saque cita. ¿Cuándo? Dizque para tres meses. No, no dijo nada, mi hermanito era poca palabra, entonces se fue para la casa. Siguió la fiebre y allá le dio el derrame y se acabó de joder.

8:29 ¶ 17 – 18, in Salud

2:78 ¶ 132, in Indigena TB.docx

[C-I05] Con la práctica que hemos hecho, caso especial, hace dos semanas, una señora venía con un cólico fuerte. Resulta que la llevamos al hospital al centro de salud. El médico me dijo que yo te voy a tomar signo vital y si aparece signo vital, bien, normal, hay que pedir cita. Entonces yo devolví en esta manera: "Doctor, si el signo vital normal, pero en dolor lo lleva ahí el paciente, ¿Qué hacemos? HÁgame el favor, consulten esa señora". Y me insistió otra vez: hay que sacar cita. Y teniendo el dolor ahí. Y yo no retire del hospital. Yo me quedé ahí. Hagamos una cosa. Yo automáticamente le llame al gerente. Es que él es un médico y a él tiene un padre quien manda. En ese hemos tenido error. Llamé al gerente, al grande allá. Me preguntó quién es esa, tal señor. Y el paciente es tal. Ah, entonces un momentico... Me atendieron. Me atendieron. En buena forma ya, porque yo me tocó.. Al ratico, en una observación se vomitó en un momentico, cuatro vómitos. ¿Qué tal en el camino se me vomita? ¿No se me muere? En ese hemos tenido, porque uno no tiene pacientes que no tienen conocimiento, lo que yo estoy hablando, no lo hablan así mismo. En ese hemos tenido error.

8:32 ¶ 25 – 28, in Salud

2:108 ¶ 102 – 104, in Indigena TB.docx

[C-I04]: El año pasado, acá en hospital que trabajé dos meses en la traductora, el niño era grave. Tenía mucha asfixia, venía de la comunidad de Piedra y ahí se pasó. Y la mamá lo llevó a hospital. Y el niño que estaba asfixiado, tosía, todo. Y el médico le mandó para la casa. No, que el niño que estaba bien, estaba bien. No, yo le dije a el doctor "como así que usted va a mandar para la casa, ¿no ve que el niño estaba grave? ¿No ve que el niño está asfixiada?". Y no, no. Ahí también me regañan porque no se puede hacer nada, no era su trabajo. Y por eso me quedé callada. Desde 3 días, lo mismo lo traía ella al hospital. Y en hospital, dice que "no, que el niño no está asfixiado, llévelo para la casa". Y la mamá también vuelve a traer pa' la casa. Y allá... cuatro veces lo llevó al hospital. Y lo llevó a Pereira. Y de Pereira lo llevó a Armenia. Armenia lo transmitió allá y en la mitad murió el niño. El niño que estaba grave. Y por eso ahí hicieron un paro. También allá en el hospital.

[INVESTIGADORA] ¿eso fue hace..?

[C-I04]: El año pasado, trabajé dos meses y ya se acabó mi contrato.

8:36 ¶ 39 – 42, in Salud

3:41 ¶ 410 – 412, in AFRO_TB.docx

[A01]: ah no, y le digo que las enfermeras también lo hacen...

[A07]: y ellas lo hacen porque como ven al médico hacerlo y como la comunidad no le dice nada al médico, ella se va adoptando esas malas costumbres.

[A01]: cuando yo trabaje en el centro de recuperación nutricional, nosotros tenemos un médico, pero contábamos con la doctora, que ella vive en la cabecera municipal, a veces el niño o algo le decíamos a la doctora, vea ella decía vamos a hacer esto y esto y vamos a mirar la evolución, vamos vigilar el niño o hay niños,

los niños con desnutrición sabemos que es una enfermedad mortal, que tiene atención... una urgencia prioritaria, en cualquier momento se descompensa y llega uno a la seis y media con un niño y que hay... no tuvo todo el día para traerlo, por qué lo trae a esta hora. Entonces uno tiene que saber a qué horas el niño se va a enfermar para llevarlo... eso es lo que le dice la enfermera... ¡ay! Por qué lo trajo... o uno a veces va a las nueve de la noche: "a esta hora no hay médico, por qué no lo trajeron temprano que están los médicos, ahora qué quieren qué haga a esta hora, yo le tomo los signos y le digo al médico a ver él qué dice".

8:39 ¶ 131 – 132, in Salud

4:249 ¶ 1422 – 1425, in INSTITUCIONES ML & LC.docx

[I-I02]: cuando está enfermo dicen que tienen que pedir cita y dan para un mes, para dos meses así y mientras... se muere uno, el niño [inaudible]

8:54 ¶ 69 – 70, in Salud

4:3 ¶ 273, in INSTITUCIONES ML & LC.docx

[I-M01]: entonces se lo llevan, no... enfermera que el niño sigue enfermo, otra vez, devolvámoslo. Hagámosle el acompañamiento para ver si así nos lo atienden porque no sé si es que ellos no saben hablar, no saben explicar o no se les presta la atención que se debe, entonces se les hace el acompañamiento para que ese niño sea atendido y le manden pues la fórmula, explicarles a ellos muy bien cómo vigilar esa fórmula, porque a veces "ah bueno papito, el niño está ah bueno, papito, el niño tiene una infección gastrointestinal y esta es la fórmula", quizá no se le explica cómo se le debe dar el medicamento y se lo llevan para la casa, no saben cómo darle el medicamento. O se lo explican y no se acuerdan, entonces eso es como desde esa parte. Entonces ellos se van como desmotivando.

8:63 ¶ 102 – 103, in Salud

4:118 ¶ 281, in INSTITUCIONES ML & LC.docx

[I-I07]: el niño que falleció, primero llevaron al puesto de salud y después, al otro día, llegó de Sallada [inaudible] tenía... eso es lo que quiero explicar, ellos mandan para la casa ¿por qué? Porque el niño se pone peor y cuando llega a Pereira y allá se muere. Eso es lo que pasa. Porque ese niño tenía malaria, desnutrición, tratamiento también, pero como le digo, mandan para la casa al otro día.

8:68 ¶ 178 – 179, in Salud

4:379 ¶ 1412, in INSTITUCIONES ML & LC.docx

[I-A03]: porque a mi me pasó que día que una recaída y yo llegué que no era capaz ni de dar el paso y me dice un auxiliar ah es que ya fichos no hay y no la pueden atender y yo que pena con usted pero el problema que... a mí, si yo voy así me deben atender porque mi caso porque mi caso no es cualquier cosa y si aquí no hay nadie... o sea, yo sé que aquí, de pronto los primeros auxilios y váyase, ya... pero es que no, que yo no la puedo antender porque ya no hay ficho y el médico está muy

ocupado, entonces yo le dije bueno, listo, me acerqué al médico, me dijeron, le comenté mi caso particular y le dije mire que me pasa esto y esto y dijo suspendan lo que estén haciendo y me la atienden, el mismo y a los 3 días me tuvieron que hospitalizar. Entonces el problema no es que yo no la puedo atender, el caso es que hay que priorizar. Hay que priorizar porque si una persona está en muy mal estado de salud y si, por ejemplo, si usted está más mal y yo estoy más estable, atiendan a usted y depsués me pasan a mi, pero hay casos que no dan espera, no dan espera. Entonces...

9:6 ¶ 21 – 22, in Corresponsabilidad

1:66 ¶ 535, in Instituciones_TB.docx

[I-M08] Yo he pensado siempre que una de las soluciones es no darles todo, como se lo da el gobierno. Yo pienso es como por los resguardos, por comunidades, darle como más agentes comunitarios, en comida darles... no darles el mercado, darles un ingeniero agrónomo... entonces en este resguardo o para tantas comunidades y empezar a hacer truque, que ellos sepan, que hay que cultivar para comer, no que todo es regalado, porque es que ahí donde va. Y a todos nos pasaría, yo creo que si a mí me dicen ven [I-M08], te doy el millón de pesos que te ganas y no haces nada, yo no hago nada, me quedo en la casa. Entonces pasa con ellos pienso yo

9:9 ¶ 27 – 29, in Corresponsabilidad

1:144 ¶ 271 – 272, in Instituciones_TB.docx

[I-M08] Pero yo no entiendo, entonces, por qué vienen después a decir que el hospital es el culpable de todo.

[I-A03] O sea, porque esa es como la percepción que ellos tienen...

9:11 ¶ 37 – 40, in Corresponsabilidad

1:156 ¶ 266 – 268, in Instituciones_TB.docx

[I-M01] Yo creo que de pronto también a veces son despreocupados, una falta de consciencia. Por ejemplo, hoy, vinieron a cobrar lo del incentivo que están, o sea, sí, estuve por ahí cerca y una niña tuvo sangrados nasales, ¿cierto? Y la mamá no la quería traer al hospital, "—no, es que yo tengo que cobrar. — no mamá, primero lleve la niña. — No, que no, que no y que no".

[I-M08] y usted no los puede obligar, qué más hace uno.

[I-M01] Pero se le dieron opciones [inaudible] la pasamos allá adelanta para que la atendieran de los primeras, pa que ella pudiera venirse, pero de no ser así, ella no viene. Entonces como falta también de...

9:16 ¶ 53 – 54, in Corresponsabilidad

1:186 ¶ 484, in Instituciones_TB.docx

[I-I04] ellos decían: si se van para el hospital allá los matan [Risas]. Eso decían, por es ellos... les daba a todos, pero quedaban reservados en la casa.

9:17 ¶ 55 – 56, in Corresponsabilidad

2:1 ¶ 119, in Indigena TB.docx

[C-I02]: A la vez la doctora no es de acá, es de otro departamento. Ella es cauquenia. Entonces no está bien... ...no está muy de mano con el resguardo Chamí Embera Unificado, nos está desconociendo, ella es muy cascarrabia. Cuando ella se está enojando, ella no quiere a los indígenas.

9:18 ¶ 57 – 58, in Corresponsabilidad

2:9 ¶ 143, in Indigena TB.docx

[C-I05]. O sea, otra importancia que hace anitos, hace añitos, médicos que tenían, o sea, confianza a los indígenas, que decían "— Quenverde, ¿dónde duele? — acaí, acaí". Entonces decía "a dónde", entonces el indígena tocas, donde duele ahí, toca el indígena ahí. Entonces, ellos ya el médico ya tenían esa confianza. ¡Poropira! ¡Poropira! ¡Dolor de cabeza! ¡Acaí! acá. El médico ya recetaba... la confianza. La confianza era inmensa, era confianza. Pero ahora no hay confianza.

9:19 ¶ 59 – 60, in Corresponsabilidad

2:19 ¶ 107, in Indigena TB.docx

[C-I02]: Ahí en el centro de salud llegan médicos. Médicos vienen a hacer rurales. Tiene para práctica. No conoce todo lo que es médico abarca de trabajo. Como es nuevo, está practicando, es rural. Pero el médico hay un fallo. Y hay un médico de buena gente. Lo quieren a los indígenas. Hay un médico que no quieren a los indígenas, lo rechazan. Hay dos cosas a jefe. Ahí nosotros vemos la debilidad del Centro de salud. Hay un médico que quieren mucho al Embera. Hay unos, no todos. Y hay unos... Tratan muy poco. No socializan. Más bien dice que esto está bien y esos niños han muerto en la casa. Una vez murió una... No quisiera contarle, pero recordemos. Mi nietica así murió en Santa Cecilia. Venía con asfixia, de la casita venía con asfixia esa nochecita. Vino al médico y no lo atendieron. Dijeron que espere, que espere y en la puertica del Centro de salud murió la nietecita. Y no los demande [inaudible].

9:20 ¶ 61 – 62, in Corresponsabilidad

2:43 ¶ 111, in Indigena TB.docx

[C-I03]: Yo creo que el [C-I05] sabe, [C-I02] sabe... ...lo demás enfermeros saben. Un ejemplo, en buzón de sugerencias, Si uno ya escribe, un ejemplo, si yo escribe y pasa el buzón de sugerencia. Y eso como fue... ...y quién va a mirar o el... el gerente. Porque ya lleva cuatro veces que yo siempre... una cartica partida... haciendo... y hay dos, una negra de anterior... ...y hay una persona que contratado... ...ya es nuevo. Ellos siempre meten grosero. Uno cuando va ahí siempre meten grosero... ...por eso yo escribí para que...

9:21 ¶ 63 – 64, in Corresponsabilidad

2:58 ¶ 133, in Indigena TB.docx

[C-I03]: El médico maltrataba por la palabra. Entonces si mujer avispadita o sabe hablar, enfrentar a uno, uno cuando ya habla, ellos pueden entender normalmente. Pero si uno queda así... Un ejemplo, usted me habla muchas cosas y no entiende, Me queda así. Así como nosotros, usted cuando yo hablo con mi lengua, usted no va a entender, se queda así. Y nosotros también así mismo. Así mismo. Así cuando ya una mujer no entiende palabra en español, uno hablando, mal tratando por la palabra, uno que está hablando, uno piensa eso. Así me paso de en Santa Cecilia. Una mujer que vine y este... la enfermera, una negrita que trabaja allá, ella había hablado mal de mí a la señora y al paciente. Y yo escuchando ahí, al lado de ella, escuchando, al último me tocó hablar, porque hay que respetar a la señora. Ellos no entienden tu palabra, pero tienen que respetar, ella es paciente, usted es un enfermero, pero usted también tiene su jefe que manda. Entonces quedó callado y se fue y consultó a la médica. Y eso pasa.

9:22 ¶ 65 – 66, in Corresponsabilidad

2:60 ¶ 105, in Indigena TB.docx

[C-I01] uno cree que hoy en día de las mujeres, por qué razón no lo quieren traer al hospital. Yo creo que varios años atrás, siempre pasaba así. Este año ha pasado así. Porque hay muchas mujeres, porque [inaudible] no traen cuando un niño está en enfermo, porque no les gusta eso, porque hay veces los médicos solamente lo miran, que el niño está bien y lo mandan a la casa. Y por eso la mujer casi no le gusta, que no, que el médico... y lo mandan, ¿para qué lo van a llevar? Para eso tratan con Jaibaná y lo pasa con mejor trabajo [inaudible] familia. A los último, los enoja uno. Es que el médico no se mide y por qué van a ir más allá. Siempre hay una mujer que lo tratan así. Porque eso... es un mundo porque.... No hay buena atención.

9:25 ¶ 74 – 75, in Corresponsabilidad

2:149 ¶ 110, in Indigena TB.docx

[C-I02]: Es que en el hospital hay unos médicos muy groseros. Sí, muy grosero. Yo estaba la semana pasada, había estado allá, al hospital, que una señora llegó del Chocó... que una niña que tiene mucho brote en la cabeza. Y habló que no, que mantiene muy cochino, que no... Yo no quedo... Que yo le dije al señor ¿Y porque no tratan así a usted? Y como dicea ellas ¿Y usted por qué no [inaudible]? [inaudible] cómo así que van a decir que hay un [inaudible]. No puede tratar así, yo les dije a usted le faltan al respeto. Y la mamá, como ella no sabe hablar... de Choco hay una mujer, allá casi no se habla. Y también que no... que del Chocó son muy brutos que no saben hablar. Y no entienden nada. Ahí me cogió la raya, yo iba a hablar, pero yo les pego mi carpeta y ellos meten el buzón de sugerencias. Todo la parte... uno entiende nada o si no hace caso y a veces no quieren atender. Eso pasa ahí en el hospital.

9:28 ¶ 87 – 88, in Corresponsabilidad

3:64 ¶ 254, in AFRO_TB.docx

[A06]: yo que día llevé a mi mamá, en la noche y lo primero que me dijeron fue "y usted por qué no vino más temprano". ¿Y es que a ti una urgencia te dice voy? La sola urgencia, ah, no es que el médico ya se fue. Y que usted tiene la presión alta y qué por qué la tiene. Oiga niña, la tiene alta por el dolor, el [inaudible] y los signos vitales que se alteren. Es que “tome este” reporte y le dije "yo para qué me lo llevo, si yo no le voy a dar ningún medicamento, si la tiene alta, si la tiene en tanto por tanto, yo no le voy a dar ningún medicamento [inaudible]”. Hay otros días que usted va y ya le digo, usted amaneció como con dios o yo no sé, pero vienen y lo atienden ya sea aquí en Pueblo Rico, Pereira, en cualquier departamento. Esa [inaudible].

9:31 ¶ 102 – 106, in Corresponsabilidad

3:82 ¶ 406 – 409, in AFRO_TB.docx

[INVESTIGADORA] y tiene, porque ayer hablábamos de que un problema en el hospital era que venía gente afuera y había una relación tensa y con los tratos a veces, pero... o sea, qué porcentaje de la gente del hospital es de afuera...

[A01]: más que todos son los médicos...

[A07]: pero los médicos vienen pensando que somos una comunidad ignorante, una comunidad no educada y así nos tratan. No es el respeto con el que te trata un médico de la Clínica los Rosales o el Pinares Médico... no. "Pase a ver... usted no tiene nada, váyase para su casa".

[A02]: tiene que escoger el día y la hora en que se va a enfermar, porque si usted se enferma un domingo, llega con un dolor de cabeza, un malestar, "ah, no... eso es un guayabo"

9:32 ¶ 107 – 119, in Corresponsabilidad

3:133 ¶ 413 – 424, in AFRO_TB.docx

[A07]: Por esa actitud, yo perdí a un hijo. Por esa actitud, el 15 de octubre en el 2021 perdí a mi hijo. Porque desde el 8 consultamos porque ella tenía dolores de parto y ya estaba en los días y llegamos a la... bueno el médico dijo "se va a que es por cesaria" pero llegó el de Pueblo Rico y la devolvió "no, usted no tiene nada, para su casa" y el 12 volvimos y eran las 7 y media y me acuerdo de lo que dijo la enfermera, una persona que trabajó conmigo, una persona que a mí me [inaudible] y dice "siga está bien" ¿y qué hizo? Abrió la puerta malagana a las 7 y media de la noche: "yo no sé a qué vienen aquí si a esta hora no hay médico a trasnocharla a uno".

[A01]: lo primero que le dicen a uno: “¿por qué no vinieron temprano cuando estaba en el médico?”, es el saludo que le dan, por qué no vinieron temprano cuando estaba en el médico.

[A07]: llamó al médico de malagana y le dijo la información como no era. Que ella tenía cólico y que tenía estreñimiento. El médico dijo "devuélvala para su casa". No dijo que eran espasmos abdominales que eso también es síntoma de parto, no, ni que estaba dilatando, no, ella no dijo eso. Yo no estaba aquí, yo estaba en una capacitación y cuando yo llego me cuenta todo eso y yo me enojé.

[A02]: y ella cómo sabía que era estreñimiento si le hizo una palpación...? Solamente la miró y dijo: ¿es esto?

[A01]: es que se supone que si una mujer ya llega en los días de trabajo de parto, hay que hacer, hay que reportarle al médico y hay que hacer el tacto. Hay que hacerle el tacto, reportarlo y remitir.

[A07]: lo primero que dicen es "¿A qué EPS es?" "— Medimás — ah, no, a Medimás no le gustan esos casos" y la devuelven. "no, no, no, no se puede". Sí, así es. Pero cuando llega un docente que dice ahí COSMITEC, inmediatamente el CHIP cambia. [Inaudible] Estos son los que facturan y dan la plata. O otro más perverso, accidente de tránsito, no saben dónde ponerlo... Pero cuando es subsidiado, Medimás que está en la quiebra, que le debe miles de millones... ¿cuál fue el resultado? en la mañana del 15 ya dijo "yo no aguanto más", llegamos con 6 de dilatación, la subieron a la ambulancia, no medicada, sabiendo que ya tenía 6 [inaudible] era de este color el líquido amniótico. Cuarto grado de meconio, mi hijo murió ahogado. Comió meconio y lo respiró. Entonces mire la secuencia: si hay un programa de salud, si hay unas rutas que atender, en lo que sea en lo que nos estemos imaginando, en lo que estamos haciendo hoy y desde la institucionalidad no se le presta la debida atención ni se sigue protocolo diseñado para eso, nunca va a haber un resultado positivo. Nunca. ¿A qué conlleva eso? A que cuando le hicimos ese... para cambiar a la gerente del hospital no había una sola prueba de que ella estuviera haciendo mal su trabajo ¿por qué? porque ni los afros ni los indígenas demandamos. No demandamos.

[A01]: sí, eso dijeron que no había demanda, ni nada...

[A07]: no hubo una prueba, no tuvimos una prueba para sacarla

[A01]: qué pasa a las cosas y nadie demanda.

[X] se hace búlla y ya. Ahí muere.

[A01]: un día de huelga y ya

[A07]: entonces uno como líder... hoy el hospital ya tiene cinco demandas incluyendo la mía y como a mí no me interesa los 1000, 2000 o 50 pesos que lleguen por eso. Es el antecedente. Es el antecedente, con ese antecedente, el nuevo gerente que llegue, porque este ya se va en marzo del próximo año ah, él sí se va a poner la atención y va a decir que no quiero que me sancionen, yo no quiero que mi trabajo aquí se entorpezca porque todos los recursos que llegan son para pagar demandas, ahora sí voy a mirar por eso.

9:33 ¶ 120 – 122, in Corresponsabilidad

3:149 ¶ 268 – 269, in AFRO_TB.docx

[A06]: allá hacen casa y cocina, más si llegan urgencias eso terminan de excusarlos, y pueden haber dos o tres médicos y se demoran para darle una acetaminofén. Por eso es que uno se automedica y da hasta pereza ir allá.

[A02]: a mí sí me da mucha pereza ir la verdad.

9:34 ¶ 123 – 124, in Corresponsabilidad

4:10 ¶ 1452, in INSTITUCIONES ML & LC.docx

[I-I07]: pues la población indígena digamos que no interesa digamos los íntomas, pero como te dije ahorita, recurso, distancia, [inaudible] y a veces no apoya los padres de familia, a veces como los papás,cierto? A veces pasa eso [inaudible] sí pesa mucho que... y también las demás [inaudible] es por eso... como... explicar... no dan ganas de ir al hospital por esos motivos, a veces y también da pereza de caminar a pie sin plata, sin pasajes, eso pasa. Pero cuando a veces, A VECES, uno compra un medicamento, algnos son juiciosos, llevan tratamiento. Pero cuando yo veo que no me importa nada salud o compra medicamento, si no le dan al niño, pero eso cuando compran los medicamentos sí le dan sus medicamentos o hacen tratamiento con medicina así, ¿sí me entiende? Es así de sencillo

9:35 ¶ 125 – 127, in Corresponsabilidad

4:18 ¶ 1493 – 1501, in INSTITUCIONES ML & LC.docx

"tiene que llevar al niño enfermo" y no lleva de pornto es que no les creen. ¿Qué creen ustedes? ¿eso puede pasar o no?

[I-M01]: si, se ha visto muchos casos, claro.

9:36 ¶ 131 – 132, in Corresponsabilidad

4:20 ¶ 233, in INSTITUCIONES ML & LC.docx

[I-I07]: Bueno, ese niño de Dokabú era del programa, y él pertenecía a esa comunidad, pero en esa parte, pues el médico y el demás equipo de trabajo, siempre dan una recomendación a los padres del hijo: que lleve al hospital después de tratar con el jaibaná. Si jaibaná no puede, si con el tratamiento ya no mejora la salud del niño, inmediatamente hay que llevarlo médico, al puesto de salud, para que el médico valore cómo está el niño y qué medicamento le den, pero a veces lo indignan, no hacen caso. Simplemente dicen no hubo plata, no tengo pasaje, eso es lo que pasa. Y ahí el niño va empeorando la salud, eso pasa acá, en Dokabú, siempre damos la recomendación con [inaudible]: lleve al puesto de salud y decía que no, que falta el tratamiento con Jaibaná y después mira el encuentro con la nutricionista y la psicosocial y observa que ese niño [inaudible] era muy tarde y nos toca hacer un compromiso con el papá y como el psicosocial hizo como algo...

9:38 ¶ 135 – 136, in Corresponsabilidad

4:59 ¶ 1450, in INSTITUCIONES ML & LC.docx

[I-I04]: por la distancia también, como ahorita...

9:39 ¶ 137 – 138, in Corresponsabilidad

4:80 ¶ 1458, in INSTITUCIONES ML & LC.docx

[I-M01]: ellos digo yo que no les gusta como mucho desplazarse a muchos lugares, entonces qué dicen ellos: el niño está mal, lo traen al hospital y obviamente lo tienen que remitir, entonces muchas veces las mamás, como es la mamá la que tiene que ir con el niño, ella puede que le preocupen los demás hijos o hasta el mismo esposo, dejarlo solo en la casa, entonces ellos como que priorizan la demás familia y no piensan como en las consecuencias de si yo dejo el niño en la comunidad se me puede hasta morir, ellos no...

9:40 ¶ 139 – 140, in Corresponsabilidad

4:81 ¶ 1410, in INSTITUCIONES ML & LC.docx

[I-I02]: el niño tiene malaria y el médico no quiso atender el niño, entonces yo lo iba a traer y él no quiso atender y yo hablé por urgencias al otro médico que era más formalito y él sí atendió, ese médico no quiso mandar a ese niño con malaria. Entonces él mismo atendió y mando otro medicamento para la casa y dijo que pidiera cita, el otro y a veces uno [inaudible] entonces eso pasa algunos.

9:41 ¶ 141 – 142, in Corresponsabilidad

4:94 ¶ 1461, in INSTITUCIONES ML & LC.docx

[I-I07]: a veces es miedo de ellos y ya ellos... dicen que no hay recurso para comprar pañales, difícilmente, por ese motivo, eso que ellos... esa parte y uno como...

9:42 ¶ 143 – 144, in Corresponsabilidad

4:131 ¶ 237, in INSTITUCIONES ML & LC.docx

Lo otro es que las autoridades deben asumir el rol de autoridad. No es simplemente yo soy cabildo, no, ellos tienen su junta directiva, tienen justicia, tienen gobernadores locales, tiene gobernadores mayores. Pero yo he evidenciado de que ellos ponen de cabildo a cualquiera. Me explico. Un ejemplo, yo soy el que más habla en las asambleas, entonces yo soy cabildo, simplemente porque yo soy el que más hablo, yo no tengo ni idea de pa' qué sirve ser cabildo o cuales son las obligaciones de ser cabildo. Porque ser cabildo no es ser cabildo, eso tiene una responsabilidad legal, porque él es el representante legal de ese territorio. Entonces mientras ellos no asuman ese rol, de que yo, por ejemplo, [I-I04] es cabildo, yo le digo "Cabildo vea, ese niño está enfermo y la mamá no lo quiere sacar" "ah, es que la mamá no quiere". No, yo soy autoridad. "Justicia, haga favor, me retira al niño y me lleva", pero no ejercen esa autoridad. Entonces, si no ejercen esa autoridad, el papá está libre de hacer para lo que a él le convenga y hay muchas excusas. Muchas excusas. No hay recurso, pero, resulta que muchas veces usted le dicen "bájelo, yo le doy los pasajes", "no pero hoy no puedo, mañana". Entonces ya lo

llevan mañana cuando el niño ya está complicado y no pasa nada. Y se murió el niño y no hay una investigación a no ser que sea una entidad que pida un informe, no hay... o sea... nada, a no ser que sea miembro de algún programa que esté vinculado al informe, pero en últimas, no hay una responsabilidad sobre nadie. Aun conociendo de que hay una sentencia, pero no hay responsabilidad, si usted llama las autoridades que vayan a las mesas, no van, están ocupados, no delegan. Entonces hay responsabilidad de las instituciones, hay responsabilidad de los cabildos

9:43 ¶ 145 – 147, in Corresponsabilidad

4:140 ¶ 1462 – 1463, in INSTITUCIONES ML & LC.docx

¿Qué... por qué lado puede irse más la cosa?

[I-M01]: ellos tal vez creen que no les va a pasar.

9:44 ¶ 148 – 149, in Corresponsabilidad

4:141 ¶ 237, in INSTITUCIONES ML & LC.docx

No sé cuál es la percepción que tienen dentro de los resguardos con respeto a la muerte de los niños, con respecto a la muerte de las personas en el territorio, porque ellos conocen las consecuencias, porque no es que ellos no las conozcan, ellos conocen las consecuencias de que si el niño está malito, tienen que sacarlo o si no se les va a morir, y ellos saben que si el niño está enfermo se les va a morir. Pero entonces, es más fácil culpar... Es la percepción personal, ¿cierto? Entonces duró 7 días en jaibaná, lo trajeron, el niño está muy grave, tiene que llevarlo a Pereira, pero la percepción de que se lo lleven apara Pereira es que allá los dejan morir. Pero entonces, yo como familia, yo como padre de familia, ¿por qué dejé llegar al niño a ese estado? No, la percepción es: lo llevo a Pereira y allá se muere o muchas veces no lo llevan, porque ellos no tienen la percepción de la enfermedad del niño, sino de ellos, como padres, y a mi allá quién los pasajes... y a mi allá... Y a mí allá... y a mí allá... y a mí allá... exacto no visualizan la enfermedad o el estado de salud del niño. Entonces eso ha sido una barrera grandísima, por más que uno les dice que en Pereira hay hogar de paso, que usted les dice las cosas. Entonces, muchas veces, estando en el puesto de salud, porque cuando yo era coordinador, les decía los vamos a llevar a Pueblo Rico y ¡Pum! Se volaban... y si los vamos a llevar a Pueblo Rico es porque el niño está maluco, no lo van a llevar a pasear, pero ellos se vuelan. Entonces es como que priorizan primero sus necesidades de padres, a el estado de salud del niño. Y eso pasa con todo. Si usted le dice, el niño tiene malaria,hasta que el niño no está titilando de fiebre y del IRA y que está que... pero ellos ya conocen los signos de malaria. Entonces eso es algo que... algo que tenemos que ir... sacando de la cabeza a ellos de que sepan de que... de que tiene que haber una consecuencia. De que si yo, papá, no lo saco debo tener una responsabilidad. No estoy diciendo que lo metan preso y la justicia ordinaria... aunque estamos violentando los derechos del niño, que son derechos fundamentales y están por encima de autoridades y están por encima de todo. Pero no sé si el estado es... le es mucho más fácil hacerse el... ¿cierto? Exacto y decir

"no, ellos tienen justicia propia, son autoridades, ellos allá manejan..." pero entonces dónde están los derechos fundamentales de los niños que están por encima de todo. Y mientras ello no ocurra y mientras ellos sepan de que el cabildo no es simplemente mandar, y que del cabildo no es simplemente decir vea trabaje usted por puestos... mientras no haya una responsabilidad en ellos, no va a pasar nada y eso no va a cambiar. A parte de que hay un brote de diarrea impresionante y hay mucha malnutrición por la diarrea.

9:46 ¶ 152 – 154, in Corresponsabilidad

4:148 ¶ 271 – 272, in INSTITUCIONES ML & LC.docx

Los niños se enferman, los papás llevan los niños al hospital, llegan al hospital... Ah, en el momento que el niño llega, primero prueba de malaria, porque ahorita es eso, primero prueba de malaria, lo primero así el niño lleve diarrea, bueno, otros síntomas, lo primero es prueba de malaria porque se han visto casos, ¿cierto? El papá, ahí está motivado en llevar ese niño... Porque ellos no son de que se enfermó el niño y corramos al hospital, pues no. Ellos no. Si ellos llevan al niño al hospital es porque nosotros, como operador, le decimos, cuando el niño presente estos síntomas, llévelo al hospital. Entonces, para ellos es desgastante quizás que ellos, bueno, motivados por nosotros, lleven el niño al hospital y que en el hospital, cierto, se le atiende al niño una urgencia siempre y cuando los signos de los niños estén alterados. Si de los signos está bien, para la casa. Pida una cita y para la casa, ¿cierto?. Entonces ellos se desmotivan. Si yo llevo a mi niño al hospital es porque está enfermo, pero sí me lo regresan para la casa, entonces, se van a sentir aprerezados de llevar los hijos al hospital, entonces casos se han visto. Entonces le dicen al papá "papito hay que pedirle una cita", ¿para cuántos días? Las citas están super demoradas.

[I-I02]: un mes, dos.

9:47 ¶ 155 – 162, in Corresponsabilidad

4:228 ¶ 1398 – 1404, in INSTITUCIONES ML & LC.docx

tiene que llevar el niño al médico, el niño está mal, usted está mal y no lo hacían, como si no quisieran llevarlo, ¿por qué creenq ue pasa eso?

[I-A03]: falta de voluntad pienso yo

[INVESTIGADORA] falta de voluntad, ¿qué más?

[I-I02]: falta de recurso

[I-I07]: y a veces los papás dicen que si llevo al hospital, no atienden mi hijo urgente, tengo que esperar una hora, dos horas, eso es lo que pasa casi la mayoría. Dos cosas: recursos y la demora y no atienden correctamente.

[I-I04]: ellos dicen si van a al hospital los medicamentos, primero que van a dar es acetaminofén, ellos dicen.

[I-I07]: y la acetaminofén la compra en la farmacia, es lo que dicen también a veces.

9:54 ¶ 177 – 178, in Corresponsabilidad

4:350 ¶ 1457, in INSTITUCIONES ML & LC.docx

[I-I07]: falta de interés, cuando uno va, se puede ver la falta de control y las vacunas, ¿no? Siempre me dicen no tengo plata con qué, entonces yo les digo, yo los llevo en la moto, yo colaboro y ya... tiene la posibilidad, entonces también por el interés [inaudible] uno colabora, se colabora, ahí llevo en la moto conmigo, pero también [inaudible] llecarlo solo a ellos o sola... y tampoco no caminan, algunos sí caminan, pero alguno sno, todos no, les da pereza, no sé y a veces dicen ah que mi marido está cuidando a mi hijo que yo por qué...[inaudible] que mi esposo no está haciendo nada, que no está apoyando al niño que está enfermo, que le está dando plata y las mamitas quedan ahí, paradas, así es como lo que yo... en el programa, eso comentaban

9:59 ¶ 192 – 194, in Corresponsabilidad

6:32 ¶ 689 – 690, in Anexo 2 Transcripcion grupo focal de malaria y leishmaniasis con comunidad afrodescendiente.docx

ustedes creen que también hayan como problemas tal vez con el conocimiento de la gente como en identificar esos síntomas y saber cuando hay que ir o sí van y siempre es que no los atienden. Bueno, ya hemos dicho que pereza, que a vedes les da pereza.

[C-A01]: la verdad es que le dicen a uno que consulte al otro día. A mí me pasó. Yo tengo una niña de 7 años, desafortunadamente ella me nació enfermita, ella me nació con un asma, todavía la tiene. Una vez, a ella le dan las crisis de la nada, igual es un asma no especificada, los médicos no saben qué... y una vez le dio una crisis así y ella cuando le dan las crisis ella como que se queda así como dormida y bastante agitadita, cuando eso estaba el doctor pediatra y yo la lleve al hospital así tapadita en la tarde, y le dije doc, ya era como las 5, y yo le dije "yo entiendo — por eso le digo que hay mucha falta de persona también, que ya un médico cansado, haber atendido 40, 50, personas en el día también es mucho cansancio, pero si llega alguien suplicándole a uno, quizá uno también debe ponerse la mano en el corazón, yo le dije— Mire, la niña está muy mal" y me dijo "no, tráigala mañana, que ya no estoy haciendo más consulta y todo" y pero mire y no me escuchó, me tocó pedirle el favor a la hermana, a el hermano de la señor que me llevara a Pueblo Rico, la niña de una vez para Pereira y en la UCI 19 días, entonces mire, si b yo no hago la gestión la niña se me mure y fui al hospital...

9:60 ¶ 195 – 206, in Corresponsabilidad

6:108 ¶ 648 – 658, in Anexo 2 Transcripcion grupo focal de malaria y leishmaniasis con comunidad afrodescendiente.docx

[C-A02]: yo sentía que me hacía así, así, así, fue tanto que yo empecé a hiperventilar, cuando me dijeron acuéstela ahí en la camilla, dijeron, acuéstela ahí en la camilla, fueron y le tocaron a la doctora, ah no, yo estoy ocupada, cuando por ahí llegó, atendió a un paciente que llegó por una consulta, para que le leyera unos exámenes, eh, lo atendió y ahí sí salió "qué es lo que tiene", entonces yo no podía hablar, entonces mi hermana, mi mamá y mi prima que estaban ahí, dijeron que no, es que tiene un dolor de cabeza muy fuerte, no es capaz de hablar, vea que está hiperventilando y "ah, ya va" y se fue. Ya por allá llegó una enfermera a aplicarme una inyección, ni siquiera fueron capaz de decir "ah, no, ella tiene esto, voy a aplicar esto para que le baje el dolor", nada, nada, entonces "doctor, qué es lo que le van a aplicar", "ah, usted quiere que se mejore o quiere seguir así?". Entonces mi prima le dijo, yo quiero saber qué le van a aplicar [inaudible] me dijo disque, usted está con dolor de cabeza o se está haciendo.

[INVESTIGADORA] o sea, la actitud del personal de salud

[C-A06]: no y la fama que [inaudible] con un dolor de cabeza horrible... eh.. le dieron la cita y el médico le mando la farmacia y la muchacha le dio el que no era y se lo tomó y la noche le estaba dando...

[C-A01]: se iba a infartar y era un medicamento muy básico, pero a ella le caía mal, la señora se complicó

[C-A06]: y lo que le dijo ella, no, me equivoqué...

[inaudible]

[C-A01]: pero fue la semana pasada, mi hermana fue antier al hospital y dijo que le habían despachado unos medicamentos...

[C-A06]. y le dijo que ay no que se equivocó, que... tan metidas.

[C-A01]: no, cualquiera se equivoca, sí, pero son cosas de mucho cuidado, porque uno puede...

[INVESTIGADORA] ¿es común ese tipo de equivocaciones?

[C-A02]: a mi una vez, le dije que yo era alérgica al ibuprofeno y me formuló ibuprofeno, yo cuando la muchacha fue yo le dije por qué y no es que aquí en la fórmula, cuando yo le dije al médico que era alérgica, cuando regresé donde él le dije, Doc, me está formulando ibuprofeno y yo soy alérgica al ibuprofeno, y qué te da el ibuprofeno, me da taquicardia, ah eso no es una alergia... entonces imagínese pues... eso es negligencia, tanto enfermeras... todo el personal

9:62 ¶ 213 – 215, in Corresponsabilidad

6:212 ¶ 931 – 932, in Anexo 2 Transcripcion grupo focal de malaria y leishmaniasis con comunidad afrodescendiente.docx

[INVESTIGADORA] porque nosotros escuchamos, pues nos han contado que varios niños se han muerto [inaudible] [C-A01]: ¿señora?

[INVESTIGADORA] que últimamente nos han llegado historias de niños indígenas que han muerto [C-A09]: sí, pero cuando los sacan, ellos ya vienen de una manera ya deshidratados, de todo. Entonces a las mamás indígenas hay que educarlas mucho sobre eso, que si los mismos enfermeros ¡Sáquenlos! No dejarlos tanto tiempo.

9:63 ¶ 216 – 222, in Corresponsabilidad

6:213 ¶ 642 – 647, in Anexo 2 Transcripcion grupo focal de malaria y leishmaniasis con comunidad afrodescendiente.docx

[C-A02]: creería que una de los factores que la gente no va y no va al hospital es la negligencia, la negligencia... aquí se ve mucha negligencia con el trato, al servicio en el hospital eso es pero re malo y eso es lo que más está afectando.

[INVESTIGADORA] ¿pero negligencia quieres decir que no los atienden?

[C-A08]: no, en algunos casos sí los atienden, la cosa es que sí los atienden, pero, cómo le dijera yo, a usted lo atienden y usted va por una cosa lo mira el médico, le manda medicamento y nunca le manda pues un examen que es lo que gente anda buscando y lo mandan para la casa, la persona se toma el medicamento y han habido casos que la persona la mandan para la casa, pasan dos tres días y fallecen

[C-A01]: se complican

[C-A08]: y en otros casos les dan la cita, si la persona va por una cita hoy, se la dan para dos, tres, cuatro meses, la persona, ¿qué va a hacer en todo ese tiempo, entonces es más que todo por eso?

[C-A07]: por eso uno busca particular, es más ligero

13:43 ¶ 143 – 146, in Percepción de riesgo

4:105 ¶ 1477 – 1479, in INSTITUCIONES ML & LC.docx

[I-I07]: cuando a veces el niño comienza con síntomas normal, a veces, eso ha pasado, que a veces no se preocupan casi...

[I-I02]: a veces dejan morir

[I-I07]: pero cuando ya... le va a dando [inaudible] ya ahí sí preocupan, ahí sí preocupan, no importa que yo tenga o no tenga plata, ahí tengo que... ahí sí ya... si pasa algo, [inaudible] eso es lo que pasa con el indígena. Entonces usted dice lo de los recursos, no es que solo un rato tiene fiebre y ya, solo una ve, pero uno no sabe.. y el niño va empeorando la salud, se ve el físico como está comiendo y eso es lo que pasa.

13:54 ¶ 201 – 202, in Percepción de riesgo

4:282 ¶ 1473, in INSTITUCIONES ML & LC.docx

[I-M01]: ellos no piensan en eso y tal vez confían de que el niño se va a aliviar o... por ejemplo el caso que yo tenía, porque gracias a dios ya se solucionó y me

preocupaba un montón, ella... veníamos luchando mucho con la niña y con ellos, pero ellos no, porque no tenían con quién dejarlo, un niño más pequeño, porque la niña tenía que estar en el centro de recuperación de santa cecilia y entonces ella decía que no que ella no se podía ir con la niña, que porque ella tenía una niña más pequeña, quién la iba a cuidar, que la niña no podía estar sola, entonces por eso ella se negaba a desplazarse con la niña hasta que ya la niña se complicó tanto que ellos vieron la necesidad de que decidieron buscar ayuda.

14:24 ¶ 72 – 73, in Propuestas

5:95 ¶ 561, in Anexo 1 Transcripcion grupo focal de malaria y leishmaniasis con comunidad indígena.docx

[C-I03]: en caso de la comunidad de nosotros, las mujeres... como le digo... cuando encuentra un taller como hay veces, como los hombres, no más que discriminan nosotras, un ejemplo, si yo estoy recibiendo aquí talleres, si yo voy a la comunidad y por la tarde voy llamando para yo avisarle qué entendí y le estoy enseñando y eso como casi no quiere arrimar, uno desanima y no quiere entender "que ah, que esa mujer qué va a hacer, solamente sale eso". Entonces uno ahí cómo queda, uno quiero llevar una información aquí a la comunidad y ahí queda desanimado, uno queda pensativo, eso no más hace falta.

○ Determinantes sociales_ML

48 Citas:

5:1 ¶ 7 – 8, in Factores económicos

1:2 ¶ 257, in Instituciones_TB.docx

[I-I02] unos dicen, si el niño está enfermo, llévenlo al hospital y dicen no... no hay platica con qué llevarlo al niño al hospital, puedo conseguirlo mañana o pasado mañana [inaudible] las mamás

5:2 ¶ 17 – 18, in Factores económicos

5:125 ¶ 725, in Anexo 1 Transcripcion grupo focal de malaria y leishmaniasis con comunidad indígena.docx

[C-I04]: algunas mamás, algunas mamitas para llevar al hospital, les falta el recurso, porque... un ejemplo, si un enfermara mi hija y no hay recurso, ¿yo con qué voy a llevarla al hospital? No hay recurso. Queda difícil

5:5 ¶ 11 – 12, in Factores económicos

1:108 ¶ 256, in Instituciones_TB.docx

[I-M01] yo pienso que a veces no tienen la facilidad económicamente, o a veces les falta es como la voluntad, porque a veces uno le pone hasta los medios ahí, para que bajen el niño y no, no lo hacen.

7:2 ¶ 9 – 10, in Prácticas culturales

1:30 ¶ 546, in Instituciones_TB.docx

[I-M08] pero si el papá no da la orden, no lo llevan.

7:3 ¶ 14 – 15, in Prácticas culturales

1:46 ¶ 548, in Instituciones_TB.docx

[I-M08] el que manda es el hombre, si él no está ahí, ahí no sé cómo hace, pero la mamá no... que porque no puede disponer, que porque el esposo no está y él es el que manda.

7:5 ¶ 16 – 17, in Prácticas culturales

2:16 ¶ 461, in Indigena TB.docx

[C-I05]: el médico pensará que nosotros no nos alimentamos bien por lo que él tiene alimentación occidental y es diferente a la alimentación de nosotros, por eso debe... pero nosotros comemos alimentación que nos da alimento, por ejemplo las enfermedades que previene, por ejemplo, el que come bastante yuyo, nunca va a tener dificultad de piel la sangre, algo así, no tiene contaminación, en cambio en la occidental, comemos, pero nos contaminamos.

7:6 ¶ 18 – 20, in Prácticas culturales

2:30 ¶ 373 – 374, in Indigena TB.docx

[C-I02] el primitivito

[INVESTIGADORA] ¿el primitivito y qué más? [C-I02] bananito, yuquita... mucho alimento, la yuca y el maíz...

7:8 ¶ 23 – 24, in Prácticas culturales

2:45 ¶ 468, in Indigena TB.docx

[C-I03]: y no comemos tanta carne, para qué voy a decir mentiras si yo no como carne y arroz [inaudible] porque somos culturas diferentes, yo cuando tengo carne, a veces arroz, a veces con primitivo, porque el primitivo nos hace falta. Siempre lo tenemos de la finca, hacemos jugo, fritamos y ya, con eso...

7:9 ¶ 25 – 26, in Prácticas culturales

2:48 ¶ 467, in Indigena TB.docx

[INVESTIGADORA] eso es por la mañana. [C-I04]: por la mañana tomamos esa harina, así como usted toma la aguapanela, siempre tomamos eso, harina con bequé.

7:11 ¶ 30 – 31, in Prácticas culturales

2:93 ¶ 466, in Indigena TB.docx

[INVESTIGADORA] primitivo, yuyo, ¿cómo cocinan el yuyo? [C-I03]: a través del monte es como una... vino, se llama, vino. Otro es el pringamoso. Y eso los cocinamos, ya, eso no echamos nada, eso lo cocinamos y esa es sal, y con harina y ya.

7:12 ¶ 32 – 33, in Prácticas culturales

2:104 ¶ 463, in Indigena TB.docx

[C-I03]: pues yo he sentido cuando fui a hacer un control del niño, me preguntaba el médico si el niño alimentaba en la mañana huevo con arepa, que si toma chocolate, y yo todo escuché, escuché y a lo último yo contesté al médico, médico sabe qué, usted no sabe que indígena alimentamos diferente, culturalmente tenemos diferente comida típica. Un ejemplo, nosotros como embera, en la mañana desyunamos harina con maíz culturalmente que nosotros hicimos, con una hierba que se llama Bequé, a nosotras, ese es como nuestro chocolate con leche, harina con Bequé, así mismo alimentamos. No alimentamos leche por la mañana, no, tenemos diferente, pero alguno médicos piensan que nosostros desnutrimos los niños por no alimento de occidental. No, es que nosostros tenemos otro alimento que es natural.

7:13 ¶ 34 – 36, in Prácticas culturales

2:119 ¶ 464 – 465, in Indigena TB.docx

[INVESTIGADORA] bueno, entonces en el día, ese es el desayuno, la harina de maíz con bequé, ¿qué más?

[C-I03] y yuyo. Sí, ese es nuestro desayuno y a la nevera también primitivo.

7:14 ¶ 37 – 38, in Prácticas culturales

2:122 ¶ 475, in Indigena TB.docx

[C-I05]: lo que pasa la modernización nos ha cambiado, anteriormente la abuelita nos daba pringamosa con salecita y con primitivito y de encima de sobremesa nos daba harina, y quedaba normal, esa era como que la comida y conserbávamos mucho porque algunos muchachos, por decir algo, me confunden con [inaudible], yo tengo 80 años y yo en mi cabeza [inaudible] por encima, eso trae es de comida, en cambio ahora, yo tengo un sobrinito, sobrinito mío, hombre, por dios se llenaron de... es un niñito que crecieron, eso se traía... de resistencia y y lernarnos igualito, de pronto occidental cree que como yo como pollo me quedé bien, pero comiendo comida típica, queda igual lleno la resistencia la sangra, eso ha pasado.

7:18 ¶ 50 – 55, in Prácticas culturales

2:145 ¶ 368 – 372, in Indigena TB.docx

[INVESTIGADORA] ¿qué más? La buena alimentación... ¿el indígena se alimenta bien, regular o mal?

[C-I05]: el indígena se alimenta bien. Ahora ya está alimentando bien.

[C-I03]: estamos alimentando pura natural.

[C-I01] comida natural

[C-I06] y típica.

7:24 ¶ 79 – 80, in Prácticas culturales

4:86 ¶ 127, in INSTITUCIONES ML & LC.docx

[I-M05]: entonces ellos no tienen esas comodidades. Entonces ¿ellos qué hacen? El tarro de límpido grande que les queda, digamos, o un tarro por allá que les regalen, entonces ellos ahí van a almacenar el agua.

7:25 ¶ 81 – 82, in Prácticas culturales

4:113 ¶ 254, in INSTITUCIONES ML & LC.docx

Y que en todo lo que tiene que ver con el hogar, el papá no participa para nada. Para nada. Para nada es para nada; ni en la crianza de los hijos, ni en ir a buscar leña porque eso es trabajo de mujer, ellos lo simplemente llegan, se levanta, trabaja en el campo, llevan los alimentos y ya. Las tardes son para jugar futbol, ¿sí o no? Para reunirse entre compañeros hombre, para reunirse entre hombres y hablar, y hablar y hablar y socializar, esa es la vida del hombre en la humanidad y de ir a proteger el hogar, esa es la razón de ser del hombre, la protección del hogar trabajar la tierra y llevar los alimentos hasta ahí llega el hombre, lo demás, porque ni siquiera la cultura, si ustedes miran las que se pintan quiénes son? La mujer, las que danzan a quiénes son? Las mujeres. ¿las que llevan la crianza de los hijos? Las mujeres. ¿La que llevan la tradición, la tradición oral, toda la tradición? La mujer. Todo, porque todo, todo todo gira al rededor de la mujer. Todo, todo, todo, el hombre no está sino para procrear, proteger el hogar y llevar la alimentación. Pare de contar. No más. Entonces, ella no tiene tiempo de nada más, y antes son muy verracas que tienen tiempo de buscar leña y tienen tiempo de... Y por eso se ve... exacto y por eso es que usted ve que nosotros tratamos de abordar el tema de que, las niñas son niñas y tienen que estar jugando, pero es que ellas no tienen tiempo, y la responsabilidad de la niña que va creciendo, es cuidar al hermanito. Y no hay otra razón, y por eso, muchas veces que eso ha ido cambiando, es que la mujer se vaya vinculando a la educación, pero antes, no ahorita se ve más, y esto es un loco que se ha logrado a través del tiempo, que la mujer vaya a la educación, porque antes no. Y antes había mucho analfabetismo en las comunidades, ahora las mujeres están un poquito más despiertas. Además, de que a los hombres tampoco le gustan mucho que eso suceda porque, o sea, tu cosmovisión va a cambiar, tu pensamiento va a cambiar y es algo que dentro de los territorios no es tan viable, pero sí ha ido cambiando, y nosotros tenemos que buscar metodologías duales. Por ejemplo, lo que decía el compañero, allá llegó el señor de ratón, vamos a ver qué nos dice el señor del ratón... allá llegó [I-A03] con los bichos, vamos a ver cuáles

son los bichos y ahí van... Pero ellos hacen un esfuerzo grandísimo, grandísimo, grandísimo. Entonces los encuentros de la modalidad de bienestar familiar... usted nunca ve un hombre. Y son por la tarde. Y nunca ve un hombre... está ocupado, está arreglando la casa, está haciendo otras cosas que.... entonces nosotros para llegar a ellos hay que ser así: puntual.

7:28 ¶ 90 – 91, in Prácticas culturales

4:226 ¶ 125, in INSTITUCIONES ML & LC.docx

orque los indígenas ellos no tienen como tal una llave donde ellos puedan ir y recoger el agua o donde lavar unos platos.

7:30 ¶ 96 – 97, in Prácticas culturales

4:311 ¶ 238, in INSTITUCIONES ML & LC.docx

[I-A03]: con respecto al brote de diarrea... a ellos se les hace mucho énfasis en las aguas. Hervir agua para el consumo, entonces generalmente cuando hay visita: "¿bueno y si hirvieron el agua? — sí — muestre a ver". Va usted a mirar y no hay agua hervida. Si usted sabe que si tomaba cruda le va a dar diarrea, porque las aguas acá no son aguas tratadas. A parte de eso que hay otro agravante, que es muy fuerte, que es lo que yo siempre recalco, de hecho, una vez... no voy a decir la comunidad, el gobernador estuvo grosero porque yo le dije "venga, cómo es posible que si yo voy — a mí me corresponde visitar acueductos—" fui a hacer la visita a una comunidad y lo primero que me encuentro es que en la bocatoma había materia fecal y justamente ese día me tocaba tomar muestras de agua para laboratorio de salud pública, obvio que esa agua no va a salir apta para consumo humano, yo había tomado la muestra en la retina donde se toma la muestra y después de que tomé la muestra subía la bocatoma cuando,oh, sorpresa. Dios mío, claro. Cuando llegaron la muestra de laboratorio yo dije, de una vez eché mi agua, esas muestras van a salir más malas que caídas. Que por qué. Porque la situación encontrada fueron estas y me dice cómo. Así fue. Así fue. Entonces yo digo, venga ¿por qué motivo tienen que dejar un niño o la persona que sea, e ir a la boca toma a hacer las necesidades fisiológicas? ¿Por qué? Primero, se están exponiendo a un accidente ofídico, porque usted sabe que donde hay materia fecal, van a haber serpientes, porque ellas se alimentan de la materia fecal. Entonces va a ver, cuando menos piensa, lo mordió una culebra, llegó y mordió a cualquiera. Entonces mire y verá que ese es el primer agravante. Segundo, las infecciones que causa en el organismo la materia fecal. De eso son los problemas de diarrea, porque ellos no están hirviendo el agua, no están haciendo un control y usted se puede quemar todo el día detrás "hiervan el agua, hiervan el agua" y "no, es que el agua hervida nos da diarrea". Así le contestan a uno. "El agua hervida nos da diarrea", digo yo, al contrario, el agua hervida lo que hace es prevenir que les de diarrea

7:34 ¶ 114 – 118, in Prácticas culturales

5:2 ¶ 545 – 548, in Anexo 1 Transcripcion grupo focal de malaria y leishmaniasis con comunidad indígena.docx

[INVESTIGADORA] listo, bueno, entonces, al agua le ponemos qué? ¿Se maneja el agua bien, más o menos o...?

[C-I02]: pongámosle doctora el acueducto de la Loma, nosotros no tenemos almacenamiento de boca toma.

[C-I01]: yo, no interrumpo, ya lleva casi 14 años.

[C-I02]: finalmente manejan con almacenamiento, con ese almacenamiento ahí en el bocatoma, nosotros colocamos canecas de 2000 litros, y con eso tan de buenas que dios da suerte, maneja 47 viviendas y 4 escuelas, manejamos 105 niños, se va a cada rato cuando llueve, hay a cada rato que echar agua [inaudible].

7:35 ¶ 119 – 124, in Prácticas culturales

5:17 ¶ 520 – 527, in Anexo 1 Transcripcion grupo focal de malaria y leishmaniasis con comunidad indígena.docx

¿En las comunidades tratan el agua? Quiere decir, cuando tienen el agua guardada para tomar, ¿Cómo lo guardan, en tanques?

[C-I03]: en tanques

[C-I07]: no pero es que los indígenas solamente las mujeres utilizan un galón, un tarrito y le sacan de la quebrada y le dejan guardado, en la casa.

[INVESTIGADORA] ¿esa es la de tomar?

[C-I07]: sí, pero las adultos si lo toman así normal

7:37 ¶ 125 – 127, in Prácticas culturales

[INVESTIGADORA] ¿En Iumadé creen que es parecido?

[C-I03]: no es que la comunidad de nosotros ya tenemos algo de tomar con cloro, en el tanque, tenemos un tanque público que es de toda la comunidad utiliza ese tanque

[C-I01]: tratado, de agua tratado

7:40 ¶ 145 – 148, in Prácticas culturales

[INVESTIGADORA] ¿o sea, que no hay acueductos, pero sí llega el agua a la casa? No, toca traerla, o sea, de la llave, pero una sola llave para todos.

[inaudible]

[lengua embera]

[C-I01]: tenemos 15 mangueras

7:48 ¶ 195 – 200, in Prácticas culturales

6:197 ¶ 869 – 873, in Anexo 2 Transcripcion grupo focal de malaria y leishmaniasis con comunidad afrodescendiente.docx

[INVESTIGADORA] pero volviendo a lo que estábamos hablando ahorita, ¿ustedes creen que hay menos personas dedicadas al campo? O sea, que tal vez eso sea un factor para que haya disminuido la leishmaniasis.

[C-A01]: Ay no, eso sí

[C-A08]: en los últimos años sí.

[C-A09]: la gente, por lo menos mire, nosotros aquí en Santa Cecilia, la gente no compraba revuelto, usted iba a donde el vecino y el vecino le vendía todo. Ahorita no, el revuelto está carísimo, aquí un kilo de plátano vale 3000 pesos. La gente ya casi no...

[C-A01]: pero vea que últimamente y a finales del año pasado, la gente ha tomado consciencia de eso y nuevamente están sembrando, se está apropiando la gente nuevamente del campo porque ya no, la verdad es que la gente no quería cultivar y la generación de nosostros, ahora, nosotros no trabajamos el campo, antes sí. Pero ya último, por la encarecida y todo la gente está cosechando nuevamente.

8:2 ¶ 9 – 10, in Salud

1:153 ¶ 287, in Instituciones_TB.docx

[I-I04] Pues ellos creen los dos, pues. Sí, los dos. El jaibaná y el médico occidental. Ellos acuden primero al médico que trata con jai, y después ellos vienen a los hospitales. Pero también hay unas enfermedades que cura el jai, y el otro que no cura. Ahí, ellos, como confían, pues ellos, se confían de Jaibaná y a veces que no son capaz de curar, ahí sí se queda más... después vienen ya todos malos los niños ya al hospital.

8:12 ¶ 163 – 164, in Salud

4:339 ¶ 1429, in INSTITUCIONES ML & LC.docx

[I-I02]: acetaminofén, siempre que se siente uno mal, uno compra acetaminofén, para andar con ese...

8:18 ¶ 116 – 123, in Salud

4:213 ¶ 1650 – 1657, in INSTITUCIONES ML & LC.docx

[INVESTIGADORA] de hecho en estos días había uno que estaba era como con fiebre y diarrea, porque esa niña estaba en...

[I-M01]: en el centro de recuperación

[I-A03]: de ahí mismo del pueblo, de ahí mismo, era del barrio Cinto y nos quedamos todos aterrados porque, porque...

[I-M01]: ¿y de Piedras también?

[I-A03]: yo supe de dos niños del barrio Cinto que estaban en el CRN, entonces quedé como así... porque yo dije venga... eso es como general aquí que eso pase,

pero era... se me hizo raro que en afro pase algo así, se le hace a uno muy raro, porque generalmente nunca pasa.

[INVESTIGADORA]: ¿con desnutrición?

[I-A03]: eso es muy poquito usted ver un afro desnutrido, eso es un chiripazo, entonces yo me quedé como así...

8:23 ¶ 268 – 271, in Salud

6:65 ¶ 941 – 943, in Anexo 2 Transcripcion grupo focal de malaria y leishmaniasis con comunidad afrodescendiente.docx

[C-A01]: mi papá tiene una balsámica para las lombrices.

[C-A02]: acá la mayoría de Pueblo Rico [inaudible]

[C-A01]: y el ajo y la gasolina son buenísimo, la gasolina con el [inaudible] usted coge un poquito de gasolina, le echa tres goticas de limón y se lo toma y se unta [inaudible] buenísimo

8:25 ¶ 248 – 265, in Salud

6:52 ¶ 949 – 965, in Anexo 2 Transcripcion grupo focal de malaria y leishmaniasis con comunidad afrodescendiente.docx

[C-A08]: en la zona indígena, es un día, ayer estaban a punto de echarnos a todos, un niño de Bajo Gitó, cabeza y...

[C-A01]: yo no sé si el centro fue por dos que habían en Sinaí...

[C-A08]: que yo llevé... salió otro [inaudible]

[C-A01]: en Sinaí vi dos y yo les dije a las den centro

[C-A08]: aquí el cierre y por eso bienestar está que arde

[C-A01]: en La Loma también...

[INVESTIGADORA] ya... eso era lo que nos decían ayer... [C-A08]: ¿el centro? El centro tiene cupo para 10 y está ocupado y está pidiendo cupo...

[C-A01]: pero eso se puede pedir los dos cupos adicionales, eso se puede hacer, eso lo hace la coordinadora.

[C-A08]: ahí hay uno...

[C-A01]: pero solicitar el sobrecupo sí se puede para los 12

[C-A08]: pero mira que los han devuelto, ya que no quieran subir, pero los han devuelto.

[C-A01]: esa gestión sí se hace.

[C-A08]: no sabía, pero ahora sé para pelear.

[C-A01]: hacele, que se pueden los 12, eso se manda un correo.

[C-A04]: ese peladito que yo vi... eso parece una menudencia...

[C-A08]: hay un niño del deportivo que también va para esa misma posición, del deportivo.

[C-A01]: yo estuve saliendo de unas actividades, pues yo les dije, yo cumplí, yo ya no trabajo en el Centro, pero estuve saliendo como 3 meses a campo. Me encontré muchos niños con desnutrición, yo les dije ya si los focalizan o no... ya yo no puedo hacer más de ahí. Y a veces uno les colabora a las mamás, les da el pasaje porque uno ve unos niños que uy no...

8:34 ¶ 31 – 32, in Salud

2:136 ¶ 109, in Indigena TB.docx

[C-I03]: Y creo que... Es que yo creo que por el médico... Yo creo que tiene una remedio... Que puede servir mucho la enfermedad de una persona... de ser humano. Pero lo que pasa... Que el médico siempre cuando va a consultar... Él siempre le da a acetaminofén. Acetaminofén. Y no le da una medicamento que sirva. Eso es para un ratico. Pa un rato sirve el aceterminofen. Pa quitar el dolor. Pero no es para curar. Es que a mí me duele cuando va al médico... Que me de un acetaminofén. Y sabiendo que en esa... Médico siempre cana por nosotros. Uno por fijao. Pagamos la plata por resguardo Indígena, por nosotros. Y ellos no siente ellos. Solamente ellos le dan siempre acetaminofén, metrodinazol, que valen los dos 4 mil pesos y no le da para un remedio que sirva, que se calme la enfermedad de uno. Eso quería... ...que siempre pasa eso.

8:35 ¶ 33 – 34, in Salud

3:2 ¶ 252, in AFRO_TB.docx

[A06]: pues eso dependiendo, pues sí, yo lo diría como que dependiendo como que del día de que uno se levante con el pie izquierdo, el derecho, el santo, porque sí atienden, pero a veces va por una urgencia porque usted se aguantó el dolor. Aquí está y usted puede tener un dolorcito y usted puede decir, no yo me lo aguanto y no le doy a este dolorcito aquí y en algunas ocasiones usted va a determinado sitio y le dicen "ay, el médico ya se fue". Así esté ahí, por no querer atender, o le dicen "no, es que esto no es una urgencia". Y si usted va es porque ya usted, su nivel de tolerancia con el dolor, usted no lo soporta. Y ya usted se automedicó alguna bebida, alguna cosita que lo [inaudible] ya se lo hizo, entonces en esa parte, pues sí atienden, pero hay unos días, sea aquí o en cualquier otro sitio de este pueblo, de cualquier parte de Colombia, hay un día de que algo sucede allá.

8:37 ¶ 125 – 128, in Salud

4:218 ¶ 1675 – 1677, in INSTITUCIONES ML & LC.docx

[I-I07]: la diarrea con no lavar bien los alimentos, eso pasa, como dicen por ahí. Llegan a comer el alimento, rapidito lavan, rapidito y listo con su cuchillo. A veces

eso pasa, se contamina. Y a veces, lo indígenas, a veces, cuando tienen niños de 3 años a veces mandan solos a ensuciar al baño y no acompañan a veces y como el niño, a veces el no limpia eso, entonces ahí está. A veces mete la mano en la boca y ahí sale la... y a veces también se contaminan jugando en el patio y se meten la comida, porque a veces los padres de familia a veces no están pendientes de sus hijos, eso pasa mucho con el indígena a veces. Y la respiración en [inaudible] por la cocina como dijimos ahorita, a veces en la misma pieza hay cocina, digamoslo... se contamina con eso también y a veces también llevan a otra comunidad cuando está serenando.

[INVESTIGADORA]: ¿cuando está qué?

[I-I07]: cuando está serenando, a eso también afecta la respiración y también las mamás a veces fuman cigarro, tienen la mano cerca del niño y ellos se afectan la respiración, eso pasa y de [inaudible] y la IRA y también le da malaria también y leishmaniasis también da, no mucho, pero eso tiene su riesgo en este momento, a veces. Eso es lo que pasa en la comunidad

8:40 ¶ 133 – 134, in Salud

4:259 ¶ 239, in INSTITUCIONES ML & LC.docx

[I-M06] ahí es utilizar las estrategias, porque de todas maneras el agua no es potable, el agua es de fuente hídrica que nosotros decimos contaminada, aparte de que si nosotros nos ponemos en el lugar de ellos, pues es mucho más fácil yo gastarme la leña, que me cuesta tanto traer, en alimentación que en hervir el agua.

8:41 ¶ 135 – 141, in Salud

4:269 ¶ 266 – 271, in INSTITUCIONES ML & LC.docx

[I-M01]: Bueno, pues si voy a hablar un poquito. Pues, de parte de mi trabajo y como amiga de ellos, porque pues yo tengo mucho tiempo distinguiendo de los indígenas y he aprendido a conocerlos como tal. Bueno, como ya sabemos, ellos tienen sus propias creencias, ¿cierto? Pero también hay unos que también han ido cambiando esas creencias. Entonces, cuando se enferma un niño, si está bien primero jaibaná, hay unos que primero jaibaná, como hay otros que ya han cambiado esa mentalidad, y van al hospital, ¿cierto? Entonces, ellos, ¿qué dicen? Bueno, llevamos el niño al jaibaná, en cuanto se enferma. Otras veces se demoran para llevarlo al jaibaná. ¿Por qué? Porque quizás no tienen el dinero para levarlo, porque un jaibaná cobra, eso no es gratis. EL jaibaná cobra.

[I-I02]: cobran 30 mil.

[INVESTIGADORA] ¿ah sí?

[I-M01]: sí señora el jaibaná. Otras veces unos cobran 30 mil y otros pueden cobrar más.

[I-I02]: a veces cobran 50.

[I-M01]: Pues dependiendo de lo que tenga el niño, igual hasta una parteras, las parteras también cobran. Todos ellos cobran, eso no es gratis. Entonces, digamos así, llevan el niño, pues los dos primeros días al jaibaná, o los llevan cuando el niño lleva varios días enfermo, cuando el niño ya está muy complicado. Entonces, mientras el jaibaná hace el ritual que hacen ellos, digamos dos o tres días, y que él ve que el niño definitivamente no mejora, lo manda pues al hospital. Ya un poco complicado porque imagínese, todo ese tiempo el niño enfermoo. Entonces es ahí donde el niño llega muy complicado al hospital. Puede que en el hospital lo recuperen o sencillamente ya demasiado tarde.

8:42 ¶ 153 – 157, in Salud

4:291 ¶ 289 – 292, in INSTITUCIONES ML & LC.docx

[I-M06]: El problema ahí es hacer derechos de peticion para que el medicamento nunca falte.

[I-M01]:pero mire que en este momento ni en la farmacia creo que ya no hay.

[I-M06]: la única solución es que el medicamento no puede faltar, además de que eso es gratis y eso lo da la gobernación. Algo está pasando. Dentro de la ESE algo está pasando. porque no puede... además zona malárica y eso no es un secreto para nadie e incluso hasta uno dice que es Pueblo Rico "— y de qué parte — Santa Cecilia — ay no puedes donar, ay no puedes donar" y uno con ganas de donar y no puede. Ay no puedes, zona malárica, zona malárica. Eso no es un secreto pa' nadie. Algo está pasando al interior de la ESE, que sí es bueno que lo revisen, porque el medicamento no puede faltar. A parte de que eso lo da la gobernación.

[I-M01]: sí en este momento ni la clororquina ni la primaquina lo hay. Y esa es la malaria que está más frecuente por acá.

8:46 ¶ 43 – 44, in Salud

3:89 ¶ 255, in AFRO_TB.docx

[A01]: eso aquí es muy común, la semana pasada yo llevé a mi hija porque ya llevaba muchos días manifestando mucha fiebre, dolor de cabeza [inaudible] le di acetaminofén, no la llevé porque para hacerle la prueba de malaria no puede tomar medicamento, entonces esperé que pasara pues el efecto y luego ahorita ahí sí la llevé. Llegamos como a la una y media y no, que el médico se fue y me tocaba esperar como hasta las cinco que llegara el médico. Y la niña tenía malaria, le hicieron la prueba rápida ese día y le salió negativa y al día siguiente me tocó madrugar, porque eran las 5 de la tarde y médico no venía, que no estaba y llegué al día siguiente y que positivo para malaria, que por qué no la había llevado antes. Entonces ahí uno qué responde... yo la traje, pero no me atendieron, no podía ir a buscar al médico hasta la casa o hasta donde... ya me quedaba muy duro y tampoco le podía dar nada porque la prueba le podía salir negativa entonces queda uno como...

8:48 ¶ 47 – 48, in Salud

3:111 ¶ 444, in AFRO_TB.docx

[A07]: los extramurales no se dan para el pueblo negro. Sólo se da para las comunidades indígenas.

8:50 ¶ 56 – 60, in Salud

3:117 ¶ 425 – 428, in AFRO_TB.docx

[A01]: y yo digo que hace falta más médicos.

[A07]: claro.

[A01]: somos mucha población para dos médicos y eso genera mucha...

[A07]: somos mucha población para no tener un hospital. Si nuestra estructura física... tenemos el puesto de salud más grande de Colombia, al que se le invirtieron cuatro mil ochocientos... el puesto de salud de Santa Cecilia es el más grande de Colombia. ¿Tú sabias eso? El puesto de salud de Santa Cecilia es el más grande.

8:51 ¶ 61 – 64, in Salud

3:119 ¶ 264 – 266, in AFRO_TB.docx

[A02]: sí, yo que día madrugue y cogí una cita. me la dieron para las 9:30 de la mañana. Eran las 3 de la tarde y no me habían atendido.

[A01] y llega usted a las 9:40 y le dicen que ya no la pueden atender porque...

[A02]: me tocó irme para la casa y luego regresar porque las 3 de la tarde y el médico no me había atendido. Esperando ahí desde las 9 de la mañana. [inaudible] no la cita, yo ya tenía la cita agendada, o sea, el ficho, para sacar la cita a uno lo atienden a las 10 de la mañana y a uno lo atienden normal, pero ya era la atención médica como tal.

8:57 ¶ 80 – 82, in Salud

4:43 ¶ 1420 – 1421, in INSTITUCIONES ML & LC.docx

o solo dan es acetaminofén, pero que no atienden también, el tema de las fichas...

[I-M01]: o que si no es una urgencia

8:59 ¶ 87 – 88, in Salud

4:82 ¶ 1678, in INSTITUCIONES ML & LC.docx

[I-M01]: también de los factores que influyen en cuanto a desnutrición, digamoslo así, eh... las madres sin lunas tienden a darle lactancia materna al niño, ¿cierto? Eso sí ellas son muy juiciosas en eso y la lactancia materna exclusiva es hasta los seis meses, ¿cierto? Pero ella el niño cumple los seis meses y le siguen dando leche materna, entonces eso no le aporta los nutrientes que el niño necesita, entonces ya no van a estar como tan preocupadas de ir a hacerle como la alimentación

complementaria que la sopita, que la cremita, sino que si le llegó las 8 y no hay nada [inaudible] entonces ya es la leche, entonces no se preocupan tanto como por una alimentación complementaria que le aporte los nutrientes al niño, sino por esa leche. Entonces esa es una de los factores que lleva a los niños a la desnutrición y la otra es la comida a deshoras, o sea, no son como muy puntuales en el momento de la la alimentación al niño y que tampoco tienen los nutrientes pues... no le aportan los nutrientes suficientes al niño, como... digamos que si tienen primitivo, es primitivo, porque digamos que las condiciones del indígena es muy diferente, ellos no mantienen como todas esas oportunidades que uno tiene, entonces ellos parecen más bien de bajos recursos, digámoslo así, entonces a ellos no les preocupa tanto pues que haya un buen almuerzo o que haya un buen desayuno. Entonces eso lleva como a la desnutrición como tal.

8:62 ¶ 89 – 90, in Salud

4:95 ¶ 237, in INSTITUCIONES ML & LC.docx

y hay responsabilidad de la salud también. Porque la gerente trata de hacer muchas cosas, pero hay muchas cosas que se les salen de las manos, y dentro esas cosas que se le salen de las manos es que de pronto no consigue un médico o apenas hay uno solo y es el que atiende todo y no le va a dar para hacer todo, y ella tiene la voluntad de hacerlo, pero no da. Entonces también, no es un secreto que tal vez cuando llegan al centro de salud no los atendemos de la mejor manera, entonces se junta todo, todo se junta. Entonces... cuando nosotros abordamos los temas, sobre todo esos casos que yo no quiero ni mencionar aquí, pero que estamos alborotados todos... uno trata de ayudar.

8:64 ¶ 112 – 115, in Salud

4:199 ¶ 1407 – 1409, in INSTITUCIONES ML & LC.docx

[I-I07]: el acetaminofén [inaudible]

[I-I02]: y a veces en el hospital, a veces el niño tiene malaria y...

[I-I07]: si tiene infección o algo así: acetaminofen.

8:67 ¶ 110 – 111, in Salud

4:170 ¶ 278, in INSTITUCIONES ML & LC.docx

los de la modalidad tiene que estar pendiente de 350 beneficiarios, ellos tienen que saber quién está enfermo y quién no... entonces llegamos al hospital y encontramos la barrera, porque no nos articulamos. Entonces, es lo que le pasa a uno "llevemos al niño al puesto de salud... pero resulta de que llega al puesto salud y hay un triaje donde se clasifica y dice que el niño no es una urgencia, pero, que lo que la clasificación la clasificación nos da es para una consulta externa, pero, nosotros dentro de la cosmovisión indígena, sabemos de que si se lo llevan, no va a volver. Entonces bueno, entonces, qué vamos a hacer.

8:71 ¶ 213 – 214, in Salud

6:19 ¶ 666 – 671, in Anexo 2 Transcripcion grupo focal de malaria y leishmaniasis con comunidad afrodescendiente.docx

[C-A06]: por ejemplo, Santa Cecilia como tal, es un puesto de salud, no es un hospital, por ejemplo, el problema aquí es el cambio, de que hoy permanece un médico, mañana permanece otro, así sucesivamente, supuestamente hay un médico para estar las 24 horas, cosa que nunca se ha dado

8:72 ¶ 220 – 221, in Salud

6:36 ¶ 664 – 665, in Anexo 2 Transcripcion grupo focal de malaria y leishmaniasis con comunidad afrodescendiente.docx

[C-A01]: yo digo que hace falta más de personal médico, porqe el personal médico no tiene la capacidad para atender tanta población, son dos médicos para tanta gente, eso les genera estrés mental y de todo.

8:81 ¶ 317 – 323, in Salud

6:206 ¶ 683 – 688, in Anexo 2 Transcripcion grupo focal de malaria y leishmaniasis con comunidad afrodescendiente.docx

[INVESTIGADORA] yo les preguntaba ahorita, los médicos que están trabajando aquí, bueno el personal, ¿son de afuera? [C-A08]: de afuera, ni los de aquí quieren trabajar.

[C-A06]: por ejemplo, hay uno que va a trabajar hasta el 8 de este mes, el flaquito, Daniel, que ya se va.

[INVESTIGADORA] ¿y por qué se va? [C-A06]: porque ya terminó el rural y él ya se va. Ahora viene... o sea que acá el médico acá, especializado en sí, no mandan, no vienen, o sea, no vienen que eso acá es un hueco, que porque eso acá es zona roja.

[C-A08]: y por la fama que se ha generado

[C-A06]: y por la fama que se ha generado, entonces les da hasta miedo venir.

[INVESTIGADORA] ¿qué fama se ha generado? [C-A08]: lo que pasa es que acá se ha generado una fama en [inaudible] entonces esa fama la generan y eso se ha creado que Santa Cecilia es la peor cosa que puede exisitir a nivel regional, entonces muchos médicos también evitan venir.

9:4 ¶ 17 – 18, in Corresponsabilidad

1:53 ¶ 471, in Instituciones_TB.docx

[I-M08] Ellos son muy enfermos de otras cosas, entonces [inaudible] la desnutrición es muy alta, entonces no hay sistema de defensa.

9:27 ¶ 80 – 86, in Corresponsabilidad

3:46 ¶ 270 – 275, in AFRO_TB.docx

[A06]: de 7 a 6 de la tarde funciona.

[A02]: de 7 a 7, siete de la mañana a siete de la noche.

[A06]: no, yo ese día que fui con mi mamá lo primero que me dijeron fue "no es que el médico ya se fue hasta el celador"

[A02]: cuando tiene una urgencia vital el médico se va, pero supuestamente es de 7 a 7, si yo fui ese día a la 1 de la tarde y no estaba el médico y era las 5 de la tarde y no había vuelto, se va.

[A06]: y así estén ahí chateando, como uno no sabe quién es quién... ah no, es que el médico ya se fue y puede estar ahí chateando.

[A02]: entonces que ah, llega alguien para una urgencia, que no que es que el médico se fue a almorzar y llega hasta las 3, si quiere espera, si no pues...

○ Malso comportamientos_ML

83 Citas:

4:104 ¶ 331 – 342, in Comportamientos

4:12 ¶ 670 – 680, in INSTITUCIONES ML & LC.docx

[I-M01]: a las embarazadas por la malaria no se les puede dar la quin...

[I-M06]: la primarquina,

[I-M01]: primerquina, solamente la clororquina,

[inaudible]

[I-M01]: pero en leishmaniasis, no.

[I-A03]: no porque...

[I-M06]: eso pasa la vena placentaria, entonces no se puede.

[I-M01]: ¿entonces qué hacen?

[I-M06]. toca desembarazar

[I-M05]: hace mucho una señora en embarazado estaba tomando

[I-M06]: acudía al [inaudible] para tratarse con hierbas.

4:110 ¶ 366 – 367, in Comportamientos

4:26 ¶ 63, in INSTITUCIONES ML & LC.docx

[I-M01]: contando pues el que tenga la colchoneta, pero ustedes saben que la... al indígena como tal le gusta dormir en el piso con todos sus hijos ahí, a veces tienen

cobija, su sábana, así mismo en el piso... no sé, será que les da calor o ya es la forma de ellos dormir.

4:114 ¶ 387 – 388, in Comportamientos

4:48 ¶ 683, in INSTITUCIONES ML & LC.docx

[I-M06]: lo que pasa es que, por lo regular, ellos se rigen a lo que mandan el territorio, pues si lo que manda el territorio, porque igual los síntomas son iguales, entonces lo que mandan en el territorio, pues Vivax, entonces se compran ahí para diagnosticar un paciente, tienen que ir a... entonces la gente tienen la percepción de que lo mandan el Vivax, yo tengo vivax, entonces mandan primaquina y cloroquina.

4:117 ¶ 400 – 401, in Comportamientos

4:63 ¶ 312, in INSTITUCIONES ML & LC.docx

[I-A03]:y otros es que no van a hacer tratamiento, que porque tienen reacción adversa, que porque les da comezón y muchos tienen malaria, salen positivos, pero no reclaman el tratamiento... eso también está pasando.

4:119 ¶ 405 – 408, in Comportamientos

4:76 ¶ 285 – 287, in INSTITUCIONES ML & LC.docx

[I-M01]: pero yo pienso [I-M06] ahí en esa partecita eso... o sea, es ilegal, ¿cierto? Que la farmacia lo venta, pero si nos ponemos a ver, en ese momento, es algo bueno. ¿Por qué? Porque sí, para mí es algo bueno, porque en ese momento el hospital no cuenta con el tratamiento indicado para la malaria, entonces, si yo tengo malaria y el hospital no me da ese tratamiento que... ¿yo qué tengo que hacer? Ir a la farmacia y comprarlo.

[I-M06]: igual, yo respeto todo lo que tú dices, el problema, ¿cuál es? Yo puedo tener vivax, yo puedo tener facilparum o puedo tener dengue. Entonces yo voy a la farmacia y simplemente voy a comprar el vivax y si no es vivax... estoy haciendole daño a mis riñones, estoy haciendole daño a mi...

[I-M01]: ah, no... estoy hablando desde el punto de vista del tratamiento que es para ese tipo de malaria.

4:123 ¶ 433 – 438, in Comportamientos

4:101 ¶ 820 – 824, in INSTITUCIONES ML & LC.docx

[I-A03]: a mi hermano y a mi hermana les dio a cada ratico.

[I-M01]: ¿pero es alérgico?

[I-A03]: Francisco es alérgico al medicamento y francisco comienza con una comezón

[EXPERTA]: ¿y lo termina o...?

[I-A03]: generalmente, pero él ya últimamente no se está tomando el medicamento. Él inmediatamente él se agarra a tomar bebidas amargas, inclusive el año pasadole dio tres veces. En este momento le está dejando descansar.

4:125 ¶ 446 – 456, in Comportamientos

4:111 ¶ 1172 – 1187, in INSTITUCIONES ML & LC.docx

El de la automedicación...

[I-M05]: ese sí funciona, pero

[I-A03]: leishmaniasis no

[I-M05]: leishmaniasis por ejemplo, por allá por la Villa...

[INVESTIGADORA] en malaria?

[I-A03]: sí

[INVESTIGADORA] leishmaniasis

[VARIAS VOCES]: no

[I-M01]: no tienen acceso a ese medicamento, en cambio la malaria sí porque [inaudible]

[I-I02]: es más fácil

4:130 ¶ 487 – 504, in Comportamientos

4:135 ¶ 1227 – 1249, in INSTITUCIONES ML & LC.docx

[INVESTIGADORA] bueno, los líderes, ¿qué tanto funcionan para hacer seguimiento y vigilancia?

[I-M05]: hay algunos que son pilas y están pendientes para ellos

[INVESTIGADORA] ¿de acuerdo todos?

[I-A03]: eso, es que generalmente los líderes como que no están muy pendientes de los...

[I-I04]: nuestro resguardo no...

[I-A03]: por acá no, los líderes no están pendientes de que si el niño está enfermo, no...

[inaudible]

[I-I02]: [inaudible] si un niño está enfermo le toca [inaudible] ellos pueden ver que está enfermo [inaudible].

[I-M01]: eso sí, cuando se muere todo el mundo...

[I-A03]: todo el mundo llega y todo el mudno queja y todo el mundo...

[I-M01]: opina

[I-A03]: y vamos a hacer un paro porque se murió

[INVESTIGADORA] entonces qué le dejamos, ¿amarillo o rojo?

[I-A03]: rojo

[I-M01]: mm... pues... no pero o sea, de ahí no es que no hagan nada tampoco, hacen, pero poquito.

[I-A03]: es que ni poquito, ni poquito

[I-I02]: rojo, derecho

4:131 ¶ 505 – 518, in Comportamientos

4:139 ¶ 1347 – 1361, in INSTITUCIONES ML & LC.docx

[I-A03]: como son peces ahí no se le echan nada... pues de pronto de se le echa...

[I-M01]: ¿en dónde?

[I-A03]: en los estanques

[I-M01]: por eso, pero estamos hablando de...

[inaudible]

[I-A03]: en general porqie usted sabe que tanto indígenas como afro tienen estanques, entonces..

[I-M01]: los estanques son donde hay peces, en las zonas indígenas no...

[I-A03]: algunos sí.

[I-M01]: pero no son tantos.

[INVESTIGADORA] o sea que los peces son más de qué zonas

[I-A03]: afro y mestiza. En algunas zonas indígenas sí tienen sus laguito.

[I-M01]: en la zona en la que yo estoy no hay, entonces no hay como...

[I-A03]: ¿ahí en Dokabú? Dokabú sí tiene

4:133 ¶ 527 – 530, in Comportamientos

4:144 ¶ 54 – 56, in INSTITUCIONES ML & LC.docx

Otra cosa, se ha hecho mucho énfasis también en el toldillo porque resulta que hemos tenido una experiencia muy maluca, porque entregamos los toldillos y cuando salimos, ya están pescando. Me ha tocado evidenciar eso, me ha tocado llamar al gobernador de la comunidad a decirle "venga, ¿qué pasó aquí? Hacia 15 minutos, nosotros terminamos de entregar y cuando el río se creció, cuando eso parecía una jauría, corrían para el río. Entonces, mire, donde queda... todo se

pierde, y luego los toldillos terminan como cerca de los sembrados. No estoy diciendo nada que no sea cierto.

[INVESTIGADORA] ¿Por qué crees que pase eso?

[I-M01]: eso, ahí es dónde voy. Resulta que ese toldillo viene con algo, no sé [un repelente]. A algunos les cae mal ese repelente, no les gusta, no les da alergia, sí, entonces esa parte como que no.

4:135 ¶ 543 – 547, in Comportamientos

4:155 ¶ 1641 – 1644, in INSTITUCIONES ML & LC.docx

[I-I07]: [inaudible] esa bolsa, esa aguasal.

[I-A03]: entonces mire que lo que hacemos en la casa, hacemos en la calle. Entonces yo digo que en la parte afro, sí hay afros, muy limpios, muy ordenados, como también hay otros que son regular tres cuartos, eso es como todo, la verdad es esa.

[INVESTIGADORA] depende de la educación familiar...

[I-A03]: depende de la educación que usted haya recibido.

4:138 ¶ 559 – 562, in Comportamientos

4:163 ¶ 611 – 614, in INSTITUCIONES ML & LC.docx

[I-I02]: dos días.

[INVESTIGADORA] ¿tú esperas dos días? [I-I02]: sí, en dos días uno siente tiene fiebre y otro día no le da, después otro día le da. Y ya uno lleva ahí mismo porque ya yo sé que tengo malaria. Yo ya sé.

[I-I07]: uno como padre de familia uno ya da cuenta uno de los hijos, fiebre y... es diferente con la malaria, es diferente síntoma... hoy da fiebre y más después normal, después como cada hora, usted se da cuenta que es malaria y la temperatura sube normal, más de 38.

4:142 ¶ 531 – 532, in Comportamientos

4:147 ¶ 1366 – 1367, in INSTITUCIONES ML & LC.docx

[I-M05]: yo me refiero, por ejemplo una pecera donde hayan peces, esos peces para que sobrevivan tienen que tener constantemente agua y salir y botar como es...

4:148 ¶ 608 – 609, in Comportamientos

4:195 ¶ 299, in INSTITUCIONES ML & LC.docx

[I-M05]: Estos días a mis dos primas les dio y dar gracias a dios que mi mamá tenía guardado un tratamiento guardado por ahí.

4:149 ¶ 610 – 613, in Comportamientos

4:203 ¶ 74 – 77, in INSTITUCIONES ML & LC.docx

[I-I07]: solo que ellos instalan el toldillo en la pared pero, ellos mismos dicen que [inaudible].

[I-M06]: exacto, entonces la armadera, desarmar eso...

[I-I07]: a veces sueltan [inaudible]

4:151 ¶ 615 – 631, in Comportamientos

4:209 ¶ 573 – 592, in INSTITUCIONES ML & LC.docx

¿Cuál es el primer tratamiento que toma el indígena y el afro? [I-A03]: el afro generalmente se toma su medicamente, pero también consume sus...

[I-M01]: sus plantas amargas.

[I-A03]: como, por ejemplo, la hoja de badea, como la balsamina, como el venadillo...

[I-M01]: que la hoja es como si fuera...

[I-M06]: la penicilina

[INVESTIGADORA] ¿pero eso es para qué enfermedad? [I-M01]: para la malaria

[I-A03]: paludismo

[INVESTIGADORA] ah, ¿sí? ¿Tambien? ¿y los hace al tiempo o..?

[I-A03]: no, no, no. Primero es el uno y ya después deja pasar un tiempo prudente y ya hacer el otro.

[INVESTIGADORA] ¿y qué hace primero? [I-A03]: el medicamento

[INVESTIGADORA] ¿el medicamento y después..? [I-A03]: las plantas. Y teniendo en cuenta que hay cosas que hacen reacción, lo digo por experiencia por mi hermana que le ha dado, 12 o 13 veces también... y ella hace reacción adversa al tratamiento de malaria, él se lo toma y él se quiere morir de la picazón, y es así, entonces no puede... Entonces él lo que se hace son bebidas amargas y después de que ya termina todo el tratamiento, se toma un tónico... ¿arquím es que se llama?

[I-M05]: sí, tónico Arquim.

[I-A03]: que también es amargo...

[I-M05]: sino que ya no viene el...

[I-A03]: como para que le ayude a purificar y pues... o sea, básicamente en las comunidades afro se acostumbran a hacer eso.

[I-M06]: y después el tratamiento se purgan

4:153 ¶ 634 – 638, in Comportamientos

4:211 ¶ 599 – 604, in INSTITUCIONES ML & LC.docx

[I-I02]: yo cuando la niña le da fiebre yo hago primero la hubiera, a bañar, hacer baño y si no sirve yo lo llevo para el hospital a ver qué tiene la niña, a ver tiene malaria. Allá saco el examen y sale positiva y allá mandan medicamento y le voy dando medicamento.

[INVESTIGADORA] listo, así haces tú, ¿y tú crees que en general la gente hace lo mismo que tú o hay diferencias? [I-I02]: no, hay algunos son diferentes. Algunos mantienen en la casa haciendo tratamiento y ahí dejan y no calma

[I-M06]: no llevan directamente...

[I-I02]: y si no calma, ellos también los llevan al hospital a ver si tiene paludismo o no es paludismo.

4:157 ¶ 662 – 664, in Comportamientos

4:221 ¶ 897 – 898, in INSTITUCIONES ML & LC.docx

[I-A03]: recolección de inservibles porque generalmente comunidad donde vamos hacemos recolección de inservibles, de hecho...

[INVESTIGADORA] ¿pero la comunidad sigue manteniendo eso? [I-A03]: mientras está uno allá. Es verdad, no nos digamos mentiras... mientras está uno allá está todo limpio, porque claro, hay comunidades muy organizadas, hay comunidades que... persiguen, continúan con la recolección, como hay otras que es únicamente en el momento que uno está. De hecho, estos días toca programar una, que hay que ir a la alcaldía que nos facilite la volqueta porque hay que ir desde la punta hacia... hacia... eso sí lo hemos hecho también.

4:163 ¶ 684 – 688, in Comportamientos

4:237 ¶ 1362 – 1365, in INSTITUCIONES ML & LC.docx

[I-I07]: es que a veces, como explicar... en los indígenas no... [inaudible] a veces hay lago cerca de la casa, digamos, pero ya como casi no utilizan, como es tan sucio, no limpian ese tanque digamos, se [inaudible] paludismo, ¿cierto? Por eso digo que, de pronto, de ahí sale y a veces lo que no escuchan, lo que no entienden a veces, no recogen basuras, a veces, dejan ahí tres días, cuatro días, o sea que hace la lluvia y ahí se queda, ahí se queda.. [ianudible] en la comunidad indígena. Y algo también hay como, como se dice... charquito de potrero como que ah bueno. También eso.

[I-A03]: porque es que ahí en... en Dokabú desperdician demasiada, demasiada agua, diario hay agua corriendo, entonces usted pasa y usted en la vía ve así el pozo de agua... entonces allá no saben cerrar llaves

[I-M05]: pero entonces el zancudo se da más que todo donde está el agua estancada, por donde no tiene por donde salir.

[I-A03]: por eso, esa agua se estanca, esa agua se estanca.

4:166 ¶ 714 – 720, in Comportamientos

4:292 ¶ 932 – 939, in INSTITUCIONES ML & LC.docx

isto, el repelente

[I-I07]: rojo

[I-A03]: rojo porque el repelente lo usan es para jugar. Es que con la experiencia que ya tuvimos...

[I-I04]: no se usa mucho en...

[INVESTIGADORA] la cultura indígena no usa mucho... ¿y el afro? ¿Usa repelente?

[I-A03]: no todos, hay algunos que no.

4:169 ¶ 730 – 732, in Comportamientos

4:300 ¶ 58 – 59, in INSTITUCIONES ML & LC.docx

[INVESTIGADORA] Bueno, esa es una razón. ¿Qué otra razón puede haber para que la gente no los use? ¿Qué creen ustedes?

[I-M01]: al indígena no le gusta. A ellos no les gusta dormir encerrados.

4:170 ¶ 733 – 742, in Comportamientos

4:309 ¶ 1072 – 1080, in INSTITUCIONES ML & LC.docx

[I-A03]: con seguridad fue en el hospital, porque uno se cura en salud con muchas cosas de pronto hizo una reacción adversa entonces el problema sobre quién va a quedar?

[I-M01]: pues la verdad yo soy enfermera, pero a mí nada de esas cosas me gusta hacerlas, yo solamente las hago con mi familia, pero así que el vecino diga "ah, venga", ah, no que pena, pero no.

[I-A03]: es mejor, por seguridad.

[I-M01]: sí me ha tocado decir que el trabajo que tengo no me permite porque no, lo mío es promoción y prevención, a mí no me tienen autorizado de colocar medicamentos, dirán que uno es malagente, pero uno no es mala gente, uno se tiene que cuidar en salud, por cualquier cosa me llega a... y dónde queda [inaudible] no... ni en las comunidades indígenas, yo eso sí no lo hago.

[I-I02]: por eso me mantiene regañando mi marido.

[I-M01]: eso que ella hace, yo no lo hago

[I-A03]: porque es que vea, el problema es que usted no sabe qué reacción adversa pueda tener el medicamento que usted está aplicando

[I-I02]: [inaudbile] echan culpa a usted que usted hizo este y para echar culpa, para que no ponga así, mejor no...

[I-A03]: es que es verdad, además que es muy delicado del caso de... hace tiempo ya, alguien comenzó a trbaajr en el puesto de salud de santa cecilia y ese alguien no tenía experiencia en inyectología, le aplicó una inyección a un niño que por poquito lo deja invalido. Y eso dio vueltas y vueltas y vueltas y eso estuvo muy delicado, porque el niño estuvo mucho tiempo en una cama, casi lo deja inválido.

4:171 ¶ 743 – 752, in Comportamientos

4:314 ¶ 921 – 931, in INSTITUCIONES ML & LC.docx

[INVESTIGADORA] ¿el toldillo? Antes hablamos harto de eso, ¿el toldillo funciona? ¿Funciona, no funciona, más o menos?

[I-M01]: más o menos

[I-A03]: parcialmente

[I-M01]: para no decir que no.

[I-I04]: en algunos ítems.

[I-A03]: para las moscas

[I-I02]: no funciona.

[INVESTIGADORA] ahí le ponemos...

[I-I02]: amarilla... verde

4:174 ¶ 772 – 775, in Comportamientos

4:324 ¶ 605 – 608, in INSTITUCIONES ML & LC.docx

[I-I07]: eso dependen de cada persona, depende del recurso. El que no quiere ir directo al hospital se compran su medicamento, la [inaudible] y la otra, ¿cómo se llaman? Se compran las dos, eso tienen recurso, si no quieren ir al hospital la compran y si no eso no les dura y a veces los indígenas a veces dan los medicamentos y les sale negativo

[I-I02]: automedican, entonces ellos cuando están automedicados después van a hacer muestra y sale negativa.

[I-I07]: entonces por eso al segundo examen.

4:175 ¶ 776 – 778, in Comportamientos

4:325 ¶ 297 – 298, in INSTITUCIONES ML & LC.docx

[I-M06]: lo bueno es que llegan al hospital y [susurra] "vaya cómprelo a la farmacia" que ya saben que es

[I-M01]: pero mira que ni en la farmacia hay ya.

4:176 ¶ 779 – 780, in Comportamientos

4:328 ¶ 893, in INSTITUCIONES ML & LC.docx

I-I02]: toldillo no.

4:178 ¶ 785 – 786, in Comportamientos

4:338 ¶ 276, in INSTITUCIONES ML & LC.docx

[I-M01]: no es por hablar mal ni nada, pero eso es cierto, si el niño va al hospital y no lo atienden, ¿qué hace el papá? Entonces se van para la farmacia.

4:181 ¶ 795 – 797, in Comportamientos

4:346 ¶ 789 – 790, in INSTITUCIONES ML & LC.docx

¿ustedes por qué creen que eso está pasando?

[I-M06]: yo le hecho la culpa a los viejos porque se murieron y no dejaron la información. Eso es uno. Dos, por el aumento de la población, al aumentar la población va a haber muhco más. Y tres es de que nos hemos vuelto más cochinos, ¿si me hago entender? No aseamos, sabemos que hay un charco y no nos importa, hay llantas... en todas las comunidades indígenas usted encuentra tarros, usted encuentra llantas, en todas las comunidades negras suted encuentra el criadero y no, no nos importa. Entonces es eso, aumenta la población, aumenta y hemos sido un poquito más decuidados, por no decir cochinos, más descuidados a la hora de los criaderos que sabemos que no... nosotros sabemos que ahí está la llanta que se está llenando de agua y no la volteamos, no la sacamos, nada... entonces yo creería que eso, pero sí ha aumentado. Por ejemplo, este año, yo creo que ha sido más que el año pasado.

4:183 ¶ 809 – 811, in Comportamientos

4:348 ¶ 1368 – 1369, in INSTITUCIONES ML & LC.docx

[I-M01]: ah, no, en Bachichí las cosas que hay es el agua ahí estancada, eso no tiene ni por donde salir, entonces yo digo que ahí sí hay criadero, porque no tiene el agua por dónde salir.

[I-A03]: cuando el agua fluye, si el agua fluye, fluye fluye, o sea, busquemos, a veces, hemos [inaudible] porque el agua está corriendo, desde que el agua no corra, usted encuentra y en Dokabú desperdician demasiada agua y esa agua usted ve un camino que... y es empozada, empozada, empozada

4:186 ¶ 816 – 819, in Comportamientos

4:355 ¶ 282 – 284, in INSTITUCIONES ML & LC.docx

[I-M06]: Y algo que yo no percibo es la idea de que en una farmacia vendan los tratamientos de malaria.

[I-I07]: eso está prohibido

[I-I02]: y lo venden.

4:187 ¶ 820 – 823, in Comportamientos

4:356 ¶ 642 – 644, in INSTITUCIONES ML & LC.docx

[I-I07]: a veces ellos toman rápido, "voy a tomar dos al día para que me mejore más rápido", pero eso no funciona así.

[I-M06]: y no solo pasa con los medicamentos con malaria, pasan con todos los medicamentos

[I-I07]: pasa lo mismo, pongamos un ejemplo del jarabe, de la que con 5 milímetros y a veces le dan entre 6 y 7, a veces uno ve que el niño está dando... a ese medicamento.

4:188 ¶ 824 – 825, in Comportamientos

4:358 ¶ 945, in INSTITUCIONES ML & LC.docx

[INVESTIGADORA] entonces digamos que... con los afros funciona un poco mejor que con los indígenas. [I-A03]: porque algunos afros utilizan repelente, bueno no todos, pero unos...

4:190 ¶ 826 – 827, in Comportamientos

4:363 ¶ 274, in INSTITUCIONES ML & LC.docx

[I-A03]: otro caso muy grave es con la automedicación, porque muchos se automedican. A veces no llevan al niño, sino que ellos mismos... y lo que se imaginan, les dicen que es bueno ellos...

4:191 ¶ 828 – 830, in Comportamientos

4:374 ¶ 66 – 67, in INSTITUCIONES ML & LC.docx

[I-M01] yo soy una que toldillo no uso, a mí me da calor el toldillo, no me gusta como el encierro ahí, de estar ahí metida. [INVESTIGADORA] Pero la mayoría de viviendas son abiertas... Entonces, al ser abiertas...

[I-A03]: No les debería de dar.

4:192 ¶ 831 – 832, in Comportamientos

4:376 ¶ 65, in INSTITUCIONES ML & LC.docx

[INVESTIGADORA] eso es cuando, digamos, uno ya lo entendió, se acomodó y pues lo usa y se acostumbra, pero entonces si uno no lo usa y no le gusta, pues... [I-I07]: da calor. [I-A03]: o sea, da calor pero ya.

4:193 ¶ 833 – 836, in Comportamientos

4:377 ¶ 770 – 772, in INSTITUCIONES ML & LC.docx

[I-M01]: no, hasta ahora no. Y desde el comienzo hicimos eso, tomó las bebidas amargas, después tomó complejo b en pastas y esta semana volvió y le dio entonces no me funcionó.

[I-M06]: tiene que purgarlo.

[I-M01]: esta semana compré el purgante, yo lo purgué claro, él se purgo... él se purgó con antivirus que limpia directamente el hígado.

4:197 ¶ 852 – 854, in Comportamientos

5:8 ¶ 458 – 459, in Anexo 1 Transcripcion grupo focal de malaria y leishmaniasis con comunidad indígena.docx

[C-I01]: [inaudible] el hospital del farmacia, porque a veces... una vez me dijo el médico así, quisque en farmacia puede comprar, pero el hospital lo [inaudible] y eso de farmacia... el médico me dijo así, usted puede comprar en farmacia, pero uno sabe que malaria es.

[C-I02]: a veces muchas mamás cuando tiene malaria ah no, no voy a llevar al hospital, voy a comprar en farmacia [inaudible] si es vivax, no sé, eso [inaudible]

4:198 ¶ 855 – 857, in Comportamientos

5:9 ¶ 599 – 600, in Anexo 1 Transcripcion grupo focal de malaria y leishmaniasis con comunidad indígena.docx

¿en las comunidades de ustedes les gusta el toldillo?

[C-I07]: no tienen platica con qué comprar.

4:201 ¶ 866 – 870, in Comportamientos

5:14 ¶ 454 – 457, in Anexo 1 Transcripcion grupo focal de malaria y leishmaniasis con comunidad indígena.docx

[INVESTIGADORA] o sea, empiezan, pero no terminan. ¿Y por qué pasará eso?

[C-I09]: eso puede ser también a veces por condiciones económicas, porque mire que hay mamás, en el caso de malaria, no es caso de condición económica, sino que mamá por el descuido se le hace pasar el tratamiento, no se la toma al día y las pasticas se van quedando y ya cuando de pronto no tenga fiebre hoy, entonces ya dice la mamá, no es que vea se curó.

[INVESTIGADORA] cuando empieza a mejorar... [C-I09]: cuando empieza a mejorar ya dice que se curó, pero mentira que uno todavía la tiene

[C-I03]: uno no sabe el cuerpo del niño todavía tiene la enfermedad, entonces la mamá 3 días cuando ah ya, el niño, cuando a calmadita, el niño ya calmó, pero el medicamento está todavía ahí, cómo va a curar.

4:202 ¶ 871 – 873, in Comportamientos

5:18 ¶ 674 – 675, in Anexo 1 Transcripcion grupo focal de malaria y leishmaniasis con comunidad indígena.docx

[INVESTIGADORA] en el terreno... listo, pero entonces, lo del repelente le ponemos rojo porque no se usa, ¿cierto?

[C-I04]: sí.

4:209 ¶ 898 – 902, in Comportamientos

5:33 ¶ 730 – 733, in Anexo 1 Transcripcion grupo focal de malaria y leishmaniasis con comunidad indígena.docx

[INVESTIGADORA] [C-I01], cuando tú trabajas con las mamás y les das recomendaciones, ¿las mamás hacen caso inmediatamente? [C-I01]: pues ahí normal [inaudible] hay unas que no.

[INVESTIGADORA] ¿y por qué no cumplirán? [C-I01]: pues a veces... por constancia, por lo económico.

[INVESTIGADORA] ¿tú les dices que vayan al médico pero les queda difícil?

[C-I01]: sí

4:212 ¶ 909 – 913, in Comportamientos

5:36 ¶ 624 – 627, in Anexo 1 Transcripcion grupo focal de malaria y leishmaniasis con comunidad indígena.docx

[INVESTIGADORA] ¿y lo usan todos o lo usan más lo niños? Bueno, don [C-I02] dice que de pronto los mayores de la antigua no tanto. ¿Los niños? [C-I07]: los niños más que todo

[C-I08]: más importante los niños

[C-I06]: todos tienen toldillo

[C-I04]: en donde nosotros no hay toldillos, pues así como nosotros, la costumbre, duermen sin toldillo

4:219 ¶ 939 – 940, in Comportamientos

5:65 ¶ 726, in Anexo 1 Transcripcion grupo focal de malaria y leishmaniasis con comunidad indígena.docx

[INVESTIGADORA] ¿y los extramurales, vacunadores, promotores que van son suficientes, van seguido? [C-I02]: no es suficiente porque el resguardo de nosotros es muy grande, es como 35 veredas por todo Pueblo Rico. Yo recuerdo 37, porque hay poquitos trabajadores, en este momento andan 3 vacunadores no más, antes éramos 8, cuando yo trabajaba. Siempre peleábamos ubicarnos en la veredas Chamí, hay unas veredas de 3 horas, hay unas veredas de 6 horas, entonces los trabajadores, son muy poquitos trabajadores, no alcanza. No alcanza con eso, llega

a la cola y no alcanza a llegar hasta la cabecera, entonces [inaudible] siempre se queda...

4:223 ¶ 951 – 956, in Comportamientos

5:76 ¶ 710 – 714, in Anexo 1 Transcripcion grupo focal de malaria y leishmaniasis con comunidad indígena.docx

[INVESTIGADORA] listo. ¿La gente se automedica mucho en malaria? [C-I02]: sí, eso sí compra ellos, ellos se automedican mucho por parte de...

[INVESTIGADORA] ¿se automedican mucho para malaria? [X] sí

[INVESTIGADORA] ¿entonces lo dejamos verde? ¿Van y se toman el tiramiento completo de malaria la gente?

[VARIAS VOCES] sí

[C-I02]: si toman todo completo, quedan curados y hay muchos pacientes compran solamente para 3 dosis y hay muchos indígenas que compran medicamento, mejoran la salud y cuando la salud ya se alivió, guardan el medicamento una partecita entonces no quedan curados, porque sí, eso sí es muy conocido...

4:224 ¶ 957 – 962, in Comportamientos

5:83 ¶ 555 – 559, in Anexo 1 Transcripcion grupo focal de malaria y leishmaniasis con comunidad indígena.docx

[INVESTIGADORA] bueno, listo, ¿ustedes creen que al indígena le gusta los talleres, las charlas que les enseñan?

[VARIAS VOCES] sí

[INVESTIGADORA] ¿y hay suficientes o faltan?

[VARIAS VOCES] faltan

[C-I02]: faltan muchas cosas. Para ser suficiente, nos falta mucho, jefe, para conocer al acento cómo arrancar... como disminuir esta enfermedad, pues nosotros seguimos mucho el taller porque aprendemos, porque sin orientación uno conoce, pero no conoce uno más allá cómo mejorar, me gustaría mucho más.

4:225 ¶ 963 – 964, in Comportamientos

5:84 ¶ 633, in Anexo 1 Transcripcion grupo focal de malaria y leishmaniasis con comunidad indígena.docx

[INVESTIGADORA] listo... el repelente, entonces el repelente, ¿el indígena usa el repelente? [C-I02]: el repelente el embera no utiliza, no sé porque el cuerito del embera no se cuida, yo no sé.

4:229 ¶ 983 – 984, in Comportamientos

5:93 ¶ 662, in Anexo 1 Transcripcion grupo focal de malaria y leishmaniasis con comunidad indígena.docx

[C-I02]: tengo 66 años, no conozco un protector de esos, ando allá flaquito, me entro, voy a sacar leño, me entro al monte, saco maíz, bueno... yo tengo fe de que los mosquitos me tienen miedo.

4:234 ¶ 1014 – 1016, in Comportamientos

5:111 ¶ 452 – 453, in Anexo 1 Transcripcion grupo focal de malaria y leishmaniasis con comunidad indígena.docx

[INVESTIGADORA] bueno... vamos entonces al tratamiento, el tratamiento de malaria, ¿ustedes ven que la gente lo termina? ¿Cómo les va con los medicamentos de malaria? [C-I03]: lo que manda el medicamento... el médico manda cómo puede darle al niño, si es 3 veces al día o si es 3 veces o a que hora pueden dar al niño, ya cumple la mamá no más.

[INVESTIGADORA] el médico dice y la mamá lo hace. [C-I01]: nosotros utilizamos el médico, el medicamento y uno pues, viene por fórmula, por orden indican hoy para darle pasta, pero hay unas mamás que no [inaudible] entonces hay muchas veces cuando no cumplen y bueno [inaudible malaria. Una vez en Bajo Gitó y una niña que tiene como 3 años venía con malaria y la mamá no terminó el tratamiento y volvió a caer de malaria y pues hay una mamá que no hace caso, entonces la niña sigue con malaria.

4:236 ¶ 1019 – 1020, in Comportamientos

5:119 ¶ 635, in Anexo 1 Transcripcion grupo focal de malaria y leishmaniasis con comunidad indígena.docx

[C-I07]: yo sí había usado eso, yo todavía en este momento estoy usando repelente, pero es que... uno, la mamá cuida mucho la salud del niño, pero es que el niño no cuida, a veces cuando uno está caminando y ya se pone a jugar, y ya el niño se pone a bañar y ya se lo lleva el viento del cuerpo lo que había untado, por eso es que da al niño malaria.

4:240 ¶ 1033 – 1045, in Comportamientos

5:136 ¶ 643 – 654, in Anexo 1 Transcripcion grupo focal de malaria y leishmaniasis con comunidad indígena.docx

INVESTIGADORA] ¿y alguna protección para los moscos? ¿para que no los piquen? [C-I08]: alguien le dijo que va a prender el fogón que tiene mucho humo, entonces dice que con ese humo le da miedo y se va

[INVESTIGADORA] ya, entonces ponen humo. [C-I04]: sí.

[C-I08]: eso con el humo se va a otro lado, si va a picar a niños, adultos...

[INVESTIGADORA] ah... ¿si ven que si hacen cosas? ya, entonces prenden el humo y eso sí saca los zancudos. ¿y dónde lo prenden? ¿en la cocina?

[C-I01]: por debajo de la casa

[INVESTIGADORA] ¿por debajo de la casa? Ah... ya, por debajo de la casa hacen eso... [C-I08]: pero si es una casa de esterillo o de pala, en material no es capaz, de humo dentro y cuando ese humo se alborota y ya los niños se pueden afectar.

[INVESTIGADORA] ¿eso es importante no? [C-I01]: cambia mucho, a veces va p'allá [inaudible]

[INVESTIGADORA] ¿y todas las noches hacen eso o cada cuánto? [C-I02]: cuando ventea.

[C-I04]: anteriormente no hacen eso...

[C-I02]: cuando echan ese humo hay que sacar todo los que están en la casa.

[inaudible]

[C-I04]: trae enfermedad.

4:243 ¶ 1055 – 1065, in Comportamientos

5:147 ¶ 400 – 409, in Anexo 1 Transcripcion grupo focal de malaria y leishmaniasis con comunidad indígena.docx

[INVESTIGADORA] ¿y hay plantas para la malaria que decíamos? ¿cierto? Ah bueno, aquí hay dos... esto es cuando uno se medica solo, uno compra el medicamento sin el doctor, automedicarse, pero pues, ¿eso se hace o no se hace? [C-I09]: algunos sí se da.

[INVESTIGADORA] ¿para malaria lo hacen?

[C-I03]: no, la malaria...

[C-I09]: para malaria algunos si lo hacen

[C-I02]: algunos sí hacen

[C-I06]: porque algunos conocen el medicamento

[C-I03]: no, porque nosotros dicen que...

[C-I08]: [inaudible] le da malaria y solo ya lleva al puesto de salud

[INVESTIGADORA] pero hay gente que lo hace...

[C-I03]: los demás

4:258 ¶ 1114 – 1115, in Comportamientos

5:201 ¶ 641, in Anexo 1 Transcripcion grupo focal de malaria y leishmaniasis con comunidad indígena.docx

[C-I02]: nosotros no tenemos repelente.

4:263 ¶ 1126 – 1144, in Comportamientos

6:12 ¶ 297 – 314, in Anexo 2 Transcripcion grupo focal de malaria y leishmaniasis con comunidad afrodescendiente.docx

[INVESTIGADORA] estamos viendo acá... entonces en tratamiento de malaria lo que hemos visto es baños, de los que nos hablaba [C-A01]. El medicamento, ¿no? Y también pues remedios caseros. ¿Y leishmaniasis? [C-A02]: o sea, hay personas que utilizan hierbas, pero es como otras... porque ellos más que todo han tratado...

[C-A01]: pero sus baños para leishmaniasis no...

[C-A06]: o sea, tomas bebidas tampoco, o sea, sí se les pone plantas, no se puede...

[INVESTIGADORA] voy a poner este entonces aquí abajo

[C-A02]: el jabón rey, también se utiliza mucho la tinta de lapicero

[C-A06]: pero mira que la tinta de lapicero yo la uso cuando me dan de a uno...

[C-A02] ¿cómo se llama lo que le echan a las bestias?

[C-A06]: curagán. Esa sirve también para la malaria... ve, para leishmaniasis

[C-A08]: ¿curagán?

[C-A06]; sí

[C-A08]: ¿la veterina?

[INVESTIGADORA] ¿qué dijeron de la leishmaniasis? [C-A01]: jabón rey... vea que también curan con limón pajarito y alkaseltzer

[C-A08]: con borojó también

[C-A01]: uno lo calienta, lo pone a calentar y [inaudible]

[C-A02]: ellas dicen curagán y el curagán es solo para el tratamiento animal

[INVESTIGADORA] ¿pero se usa? O sea, no importa si está bien o no está bien, pero hay gente que lo hace? [C-A06]: pues yo he visto que sí, hay gente que lo hace

[indistinguible]

[C-A04]: no, eso sí se echan porque cuando Robinson él vino muy desesperado [indistinguible]

4:264 ¶ 1145 – 1151, in Comportamientos

6:14 ¶ 104 – 109, in Anexo 2 Transcripcion grupo focal de malaria y leishmaniasis con comunidad afrodescendiente.docx

[C-A06]: detalles que, por ejemplo, el caso de mi hijo a él le suspendían pastillas por ese mismo problema, que a él todo pelado los pies le echaban sangre. Entonces se llevaba, incluso a Paola, tengo una amiga que se llama Paola y también le pasó lo mismo, entonces le suspendieron.

[C-A03]: que son alérgica al medicamento...

[C-A06]: son alérgicos, eso se les pela, les sale una ampolla y entonces se empezaban a rascas, eso era una piquiña y se empezaba a rascar y entonces le echaba sangre y le suspendían el medicamento.

[INVESTIGADORA] entonces les suspendían el medicamento y qué les decían, que era qué... [C-A06]: y mi mamá le hacía medicamento, sus cosas caseras y ya...

[INVESTIGADORA] ¿pero en el hospital qué les decía? ¿nada?

[C-A06]: no... una vez les hicieron exámenes y todo, le hicieron de todo, me acuerdo que estuvo como 10 días hospitalizado, pero mire que allá lo único que hacían era ponerle suero y esas cosas así porque trataron otra vez de darle una sola porque son dos [algarabía] y ya mi mamá dijo que no, que le dieran salida y lo trajeron...

4:265 ¶ 1152 – 1157, in Comportamientos

6:17 ¶ 834 – 838, in Anexo 2 Transcripcion grupo focal de malaria y leishmaniasis con comunidad afrodescendiente.docx

[INVESTIGADORA] ¿y por qué puede estar en aumento enotnces la malaria?

[C-A09]: por lo que yo le dije, el calor y también que muchas personas por lo menos tienen un balde y dejan el balde ahí que recoja agua, ahí está el criadero de los zancudos. Hay partes donde el agua no es corrida, donde hay un pozo y también está el zancudo. Hay un lago, criadero de pescados, hay gente que tiene pescado, como hay gente que no tiene pescado y esos son fijo criaderos.

[INVESTIGADORA] pero... o sea, ¿han hecho más lagos o...? O sea, ¿cuál puede ser el cambio que produzca el número de casos? Porque supongo que charcos y ese tipo de cosas siempre han habido...

[C-A02]: porque ahorita se han hecho más lagos de pecas por las... los proyectos que han llegado de piscicultura y todo eso, cuando llegan esos proyectos... y se comen las semillas, se comen el pescado y dejan el lago ahí.

[INVESTIGADORA] y no lo drenan, dejan el lago ahí... [C-A02]: o aunque lo drenen, usted sabe que la agua lluvia va a almacenar...

4:266 ¶ 1158 – 1161, in Comportamientos

6:18 ¶ 736 – 738, in Anexo 2 Transcripcion grupo focal de malaria y leishmaniasis con comunidad afrodescendiente.docx

[C-A06]: que le echen y después ya nos lo pasan [risas]. Por ejemplo acá en Santa Cecilita hay otra partecita grave con el tema de los comerciantes. Por ejemplo, nosostros el año pasado estuvimos haciendo limpieza en el Río San Juan, pero nos encontramos que la mayoría de los comerciantes en la plaza, tira la basura al San Juan, porque a veces la basura pasa los miércoles y desde los lunes sacan la basura, entonces eso es un cuento que la revuelquen... y eso también trae mucho zancudo.

[C-A08]: zancudo, ratas...

[C-A06]: sí porque revuelven los plásticos, los meten con las papas que se pudren, con todo lo que se... todo eso hacen una mezcla que se... y una vez estuvimos en una reunión y que ah no, que aquí en Santa Cecilia hay una asociación que de eso se trata de eso, pero ellos trabajan cuando los contratan, de resto no.

4:267 ¶ 1162 – 1163, in Comportamientos

6:21 ¶ 400, in Anexo 2 Transcripcion grupo focal de malaria y leishmaniasis con comunidad afrodescendiente.docx

[INVESTIGADORA] entonces fumigar las casas... y allá también, tu nos habías dicho que para la malaria el cloro, no? Se usaba echar cloro en los estanques... [C-A06]: nosotros en Cinto hacemos eso... por ejemplo en estos días se secó una poza allá en el sena, nos tocó que vaciar eso, porque ellos siempre acostumbran a que cuando van a hacer el estudio ahí hay una [inaudible] pa hacer un tanque que nos regalaron, de cloro le echamos.

4:268 ¶ 1164 – 1181, in Comportamientos

6:26 ¶ 716 – 732, in Anexo 2 Transcripcion grupo focal de malaria y leishmaniasis con comunidad afrodescendiente.docx

[INVESTIGADORA] ¿y lo del toldillo que nos decías ahorita que antes lo usaban y ya no lo usan? ¿Creen que haya forma de volver a promover ese uso? [C-A04]: pueda...

[C-A06]: pueda que sí...

[C-A02]: el problema ahorita es como estamos con ese apogeo de la malaria...

[C-A06]: con estos calores quién lo usa...

[C-A02]: resulta que este año la malaria se ha disparado pero horrible, yo creo que si ente momento llegan a darle charla sobre la necesidad de los toldillos, no venga... con un 60% que lo utilice estamos ganando. En la comunidad con un 60% que lo utilice estamos ganando, entonces yo creería que puede funcionar, o sea...

[C-A04]: es que ahora está muy alborotado.

[C-A06]: funciona, pero que no le echen ese químico...

[C-A02]: es que tiene que tener el químico, eso es lo que repele los mosquitos.

[C-A06]: hay unos toldillos demasiado, demasiado, que no pasa, que el zancudo no pasa, pero la gente aquí dejó de utilizar eso, más que todo por ese problema... porque es que había personas...

[C-A01]: da alergia

[C-A02]: da alergia, amanecíamos con la cara hinchada, entonces... no.

[C-A02]: ¿y sí utilizaban el protocolo correspondiente para usar el toldillo? De sacarlo de la bolsa, ponerlo un rato a airear...

[C-A06]: incluso nosotros lo lavábamos

[C-A02]: no, es que ese es el problema, si se lava va activar más ese...

[C-A06]: es que yo lo lavé, ¿sabe por qué lo lavé?

[C-A02]: es que hay químicos que no son compatibles con el agua...

[C-A06]: ¿pero sabe yo por qué lo lavé? Porque los que los intalaron así, fueron los que estuvieron más enfermo y fueron al hospital, entonces dije, vea lo voy a lavar, incluso lo eché en cloro, en agua a ver si sí, incluso lo eché en agua hervida, ¿quién dijo? Me dieron 3, todos 3 los boté, yo sí lo reconozco, los boté y en mi casa... ¿sabe algo? Mi hija, mi hija por ejemplo tuvo un bebé, que día fui a punto rojo y le dije al señor que me vendiera un toldillo, pero le pregunté pues que no, un toldillo que no traiga nada, un toldillo normal o sino no se lo hubiera puesto tampoco.

4:269 ¶ 1182 – 1197, in Comportamientos

6:27 ¶ 371 – 385, in Anexo 2 Transcripcion grupo focal de malaria y leishmaniasis con comunidad afrodescendiente.docx

[C-A06]: el que ha estado fumigando le digo quién es: Darío [C-A03]: ah, pero él es calladito [inaudible]

[C-A06]: pero mire que así sea callado, él ha estado fumigando

[C-A01]: ¿Dario Tapasco?

[inaudible]

[C-A04]: el que pasa por todos los negocios

[INVESTIGADORA] ¿ustedes sienten que cuando fumigaban funcionaba? [C-A02]: sí

[C-A06]: cuando había un carro que bajaba que eso olía como a guayaba

[inaudible]

[C-A03]: pero años no se [inaudible]

[C-A08]: sí, eso picaba, [inaudible] usted tenía que estar todo el día metido en el río, sino tenía que echarse canela.

[indistinguible]

[C-A01]: y un olor tan malo

[C-A04]: guayaba, eso parecía como guayaba

[indistinguible]

[C-A06]: pero muy bueno para leishmaniasis

4:270 ¶ 1198 – 1200, in Comportamientos

6:33 ¶ 350 – 351, in Anexo 2 Transcripcion grupo focal de malaria y leishmaniasis con comunidad afrodescendiente.docx

[C-A06]: uno ve mucha gente que va y compra sus cosas

[C-A04]: ¿quién es el que no se automedica?

4:272 ¶ 1204 – 1217, in Comportamientos

6:37 ¶ 467 – 481, in Anexo 2 Transcripcion grupo focal de malaria y leishmaniasis con comunidad afrodescendiente.docx

pero qué tanto la gente usa el toldillo? Y lo que... [C-A01]: muy poco, la verdad es esa, muy poco, la verdad es esa...

[C-A06]: yo creo que hasta menos

[C-A02]: yo creo que un 40%, de un 100% de la comunidad un 40.

[INVESTIGADORA] entonces vamos a hacer un semáforo, entonces es rojo... rosado, si en serio eso no sirve aquí, nadie lo usa, amarillo si hay una cantidad considerable, un 40% que lo usa y verde si sí funciona y la mayoría de la gente...

[C-A06]: más que todo los reparten en las comunidades, ¿cierto? Porque aquí en Santa se hizo pero la gente después de eso hicieron un recorrido para ver si la gente lo estaba usando y se dieron cuenta que no...

[C-A04]: es que esos toldillos los dejaron de utilizar desde que usted dijo...

[C-A06]: desde eso sí, porque mucha gente...

[C-A01]: eso le picaba a uno la cara, le daba alergia.

[C-A02]: pero eso se utilizaba mucho [inaudible]

[C-A06]: en mi caso, nosotros lo lavamos y pensábamos que lavándolo... porque ellos decían que no podíamos lavar y después de que lo lavamos, al otro día amanecimos con un brote en la cara, hinchada, entonces no...

[INVESTIGADORA] ¿entonces la gente no lo usa?

[C-A02]: en el momento, no

[C-A06]: es muy poco, yo pues no lo utilizo.

4:274 ¶ 1222 – 1224, in Comportamientos

6:44 ¶ 620 – 621, in Anexo 2 Transcripcion grupo focal de malaria y leishmaniasis con comunidad afrodescendiente.docx

[INVESTIGADORA] y esas cosas que influyen, decías que no les daban cita rápido, que les toca esperar todo el día en el hospital, ¿qué otras cosas influyen para que la gente no vaya, no quiera ir al hospital?

[C-A01]: pues por lo general les da pereza por lo que yo les dije, les toca esperar mucho o que no consiguen citas y entonces eso lleva a que la gente se automedique y que vaya a la farmacia, que tengan que ir a la farmacia.

4:276 ¶ 1236 – 1237, in Comportamientos

6:54 ¶ 87, in Anexo 2 Transcripcion grupo focal de malaria y leishmaniasis con comunidad afrodescendiente.docx

Pues en mi caso, en mi casa no se puede usar toldillo, una vez nos repartieron unos toldillos los cual los lavamos, pues nos decían que no se podía lavar, pero más, sin embargo, los lavamos y al otro día amanecimos con la cara hinchada, alborotada, o sea, en mi casa no los utilizamos... en mi casa habemos seis, siete personas ahí pues y hasta ahora únicamente le dio a mi hijo, de resto a nadie de mi casa le ha dado. Porque incluso en el tanque que mantenemos el agua, no me gusta mantenerlo lleno, pero sí conseguimos pastillas de cloro y cuando lo llenamos le echamos esa pastilla, entonces por lo tanto, a veces debajo se mantienen los zancudos, pero entonces nosotros mantenemos pendiente de lavarlo con mucho cloro y después sale esa pastilla.

4:278 ¶ 1242 – 1243, in Comportamientos

6:68 ¶ 619, in Anexo 2 Transcripcion grupo focal de malaria y leishmaniasis con comunidad afrodescendiente.docx

También algunas personas a veces no acuden a tomarse la muestra, porque se sienten débiles y les toca esperar casi todo el día en el hospital, entonces son cosas que influyen a que las cosas se den al 100% y que sí se cumpla todo, entonces por eso le digo que sí y no

4:281 ¶ 1258 – 1272, in Comportamientos

6:73 ¶ 509 – 522, in Anexo 2 Transcripcion grupo focal de malaria y leishmaniasis con comunidad afrodescendiente.docx

[C-A01]: pero ya pusimos acá pero al menos fumigamos las casa, [C-A04] dijo que eso mata todo

[C-A04]: [inaudible]

[INVESTIGADORA] ¿pero qué tanto se usa? ¿muy poquito, mucho?

[C-A01]: no, uno fumigar su casa, muchos, eso sí, verde

[C-A02]: a ese muchacho todo el mundo le compra veneno para fumigar

[C-A04]: digamos que en general, el Pueblo como tal no fumiga como antes, pero la que gente lo maneje en su casa sí

[C-A01]: el muchacho del veneno, la máquina de fumigar [inaudible]

x: [inaudible] hasta ese fumiga malaria

[C-A06]: no y que digamos las personas que mantienen fumigando [inaudible]

[C-A01]: y eso...

[C-A04]: yo en el patio de mi casa sí me gustaría fumigar, pero fumigamos ya con ese mismo, pero porque no mata las plantas, pero el otro sí que quema todo

[C-A01]: pero es que el cilantro vuelve y nace y eso

[C-A04]: no sí, pero están matando cuantas plantas medicinales que todo está saliendo después

[C-A02]: yo tenía un tenía de puras matas medicinales [inaudible] pero entonces [indistinguible] se lo comió, todo todo se lo comió, yo no le eché eso allá y [inaudible] iba a ponerse agua y el cloro y verás que no las mata.

4:285 ¶ 1290 – 1294, in Comportamientos

6:90 ¶ 505 – 508, in Anexo 2 Transcripcion grupo focal de malaria y leishmaniasis con comunidad afrodescendiente.docx

[C-A08]: ahora otra cosa, son muy tendidos a que el estado todo lo regale, que si el estado no lo da nosotros no somos capaces de hacer nuestras propias acciones para prevenir... que está pasando, el estado somos todos nosotros, todos tenemos que poner un grano de arena para que el estado nos pueda conformar. Si el estado no viene a fumigar, nosotros podemos sacar diez mil pesos y fumigar nosotros mismo

[C-A01]: pero hay gente que no... no le da

[inaudible]

[C-A08]: somos muy tendidos a que siempre sea el estado, siempre fuera el estado.

4:288 ¶ 1300 – 1303, in Comportamientos

6:97 ¶ 754 – 756, in Anexo 2 Transcripcion grupo focal de malaria y leishmaniasis con comunidad afrodescendiente.docx

[C-A09]: ahí donde Hernán vive, por ejemplo, ahí también, ahí da mucho, pero porque ahí abajo hay unas pocetas, unas peceras que... yo creo que es por eso a Hernán le da mucho paludismo.

[INVESTIGADORA] ¿por qué hay peceras? ¿O sea, tiene peces?

[C-A09]: sí, pero como ellos se hacen así en el bordito de los estanques y todo eso...

4:292 ¶ 1315 – 1318, in Comportamientos

6:114 ¶ 401 – 403, in Anexo 2 Transcripcion grupo focal de malaria y leishmaniasis con comunidad afrodescendiente.docx

¿qué otras cosas se pueden hacer o qué cosas se hacen en las comunidades? Tanto para malaria como para leishmaniasis...

[C-A06]: en mi caso yo quemo las cubetas del huevo

[C-A01]: también quemar basura, cuando hay muchos zancudos

4:293 ¶ 1319 – 1322, in Comportamientos

6:115 ¶ 428 – 430, in Anexo 2 Transcripcion grupo focal de malaria y leishmaniasis con comunidad afrodescendiente.docx

[INVESTIGADORA] ¿y con la malaria cuando inician los síntomas sí van? [C-A01]: algunos también se automedican, van y compran a la farmacia, van y sienten los síntomas y algunos van y compran a la farmacia.

[C-A06]: porque en la farmacia ya venden el medicamento también, entonces ellos no van hospital, sino que lo compran directamente en la farmacia, como ya saben cuántas le daban, hace uno la cuenta

[C-A06]: pero el problema... se hizo una capacitación sobre eso porque hay unos que compran el medicamento, pero no saben cuál de las dos malarias tienen, porque hay unas que la mandan... cuando es falcíparum mandan un medicamento, cuando es otro, mandan el otro medicamente. O sea, hay uno más fuerte que el otro o le mandan más que otro, pero ellos compran su medicamento.

4:295 ¶ 1325 – 1332, in Comportamientos

6:121 ¶ 460 – 466, in Anexo 2 Transcripcion grupo focal de malaria y leishmaniasis con comunidad afrodescendiente.docx

¿puede que vayan al tratamiento médico o a una persona de la comunidad que sea reconocida por hacer tratamiento o a quién acuden primero en caso de malaria?

[C-A06]: yo creo que la mayoría más que todo asisten al hospital

[C-A01]: porque los tratamiento que se hacen en casa, la mayoría de la gente, conocen algo que se toma.

[INVESTIGADORA] ¿o sea, ya sabe? [C-A01]: exacto, ya saben.

[C-A02]: cuando la persona empieza con síntomas, porque el síntoma que empieza es el dolor de cabeza, lo que uno hace es automedicarse: se toma la pastica para el dolor de cabeza, o sea, que lo primero que hacen es acudir a la farmacia y ya al ver que no pasa, que no calman los dolores, entonces ya ahora sí van al hospital

[C-A08]: y [inaudible] porque tienen el medicamento

[C-A02]: exacto, entonces ahora sí que van al hospital, hacen su debido proceso y ahora sí ya cuando le está con malaria, toman el medicamento y pasan a las cosas medicinales.

4:299 ¶ 1342 – 1358, in Comportamientos

6:141 ¶ 488 – 503, in Anexo 2 Transcripcion grupo focal de malaria y leishmaniasis con comunidad afrodescendiente.docx

[INVESTIGADORA] bueno, ¿y el repelente? ¿qué tanto se usa?

[C-A06]: yo sí lo utilizo, pero cuando salgo a campo, de resto en mi casa no.

[C-A04]: En mi casa sí a veces lo usamos pero para la niña, como está haciendo tanto calor.

[C-A02]: aquí en la población de aquí, no...

[C-A06]: pero mire que fuera del repelente hay otro medicamento que se llama el Caladryl, mi hija sí lo utiliza, es transparente, ella siempre antes de acostarse... ella antes de acostarse se empapa todo porque ella duerme hasta sin ropa, entonces ella se unta eso...

[C-A07]: ¿ese caladryl cómo es?

[C-A06]: es super...

[inaudible]

[INVESTIGADORA] pero entonces el caladryl... bueno, el repelente en general sí es rojo o es más o menos?

[C-A02]: es rojo

[INVESTIGADORA] bueno... ¿las charlas educativas qué tanto pasa? [C-A06]: ¿qué significa este rojo?

[C-A01]: el repelente, que es muy poco

[INVESTIGADORA] rojo es que no se usa... [C-A03]: pero el repelente sí no debería ser rojo

[C-A06]: porque hay muchas mamás que lo utilizan más que todo en sus hijos

[C-A01]: entonces amarillo

[INVESTIGADORA] ¿qué? [C-A01]: para el repelente

4:302 ¶ 1372 – 1373, in Comportamientos

6:149 ¶ 504, in Anexo 2 Transcripcion grupo focal de malaria y leishmaniasis con comunidad afrodescendiente.docx

[INVESTIGADORA] amarillo para el repelente... ¿y charlas educativas? ¿sí se hace? ¿es común? [C-A02]: sí se hace, no a fondo como debería ser, pero sí se hace, así como mencionaba el compañero ahorita, que muchos vienen acá a dictar charla y también a legalizar cosas, pero sí se hace, sino que si... si uno en el momento que vienen a dar charla, nosotros como los propietarios del problema le exigimos a ellos que nos aclaren, que nos digan las cosas bien... pero nosotros, ellos vienen a legalizar y uno por irse con el afán, ah bueno, lo poquito que dé y se va para su casa, entonces es culpa de parte y parte.

4:306 ¶ 1388 – 1391, in Comportamientos

6:161 ¶ 523 – 525, in Anexo 2 Transcripcion grupo focal de malaria y leishmaniasis con comunidad afrodescendiente.docx

[C-A01]: bueno, nos quedamos en las charlas educativas.

[INVESTIGADORA] ¿se hace mucho, poco, cómo lo ven ustedes?

[C-A02]: pongámosle amarillo

4:308 ¶ 1394 – 1397, in Comportamientos

6:163 ¶ 330 – 332, in Anexo 2 Transcripcion grupo focal de malaria y leishmaniasis con comunidad afrodescendiente.docx

[INVESTIGADORA] ¿creen que la gente se automedica? O sea, compre las medicinas... [VARIAS VOCES] sí

[C-A08]: [inaudible] automedicarse

[C-A06]: solo pepas, para ir a comprar Aralem que ya no lo venden.

4:310 ¶ 1407 – 1410, in Comportamientos

6:178 ¶ 485 – 487, in Anexo 2 Transcripcion grupo focal de malaria y leishmaniasis con comunidad afrodescendiente.docx

[INVESTIGADORA] bueno ¿y esto de recoger aguas y basuras? [C-A06]: puede recoger aguas y basuras o puede... ¿le digo la verdad? Es muy duro

[C-A02]: póngale rojo, rojo, rojo, se ve mucha basura en todo lado.

[C-A06]: por la parte donde yo vivo, los miércoles, los miércoles no, los martes, como los miércoles pasa la basura, nosotros cogemos a hacer todos los tarros, pero hay una señora que tenía como una caneca y dentro de la caneca tenía un poco de tarros, estaba lleno de agua... eso estaba así de zancudos.

4:314 ¶ 1424 – 1435, in Comportamientos

6:183 ¶ 526 – 536, in Anexo 2 Transcripcion grupo focal de malaria y leishmaniasis con comunidad afrodescendiente.docx

[INVESTIGADORA] Listo... y entonces cloro en los estanques, ¿qué tan común es? [C-A01]: ya cuando uno los va a lavar como para dejarlos, yo sí...

[C-A06]: ¿para qué?

[C-A01]: para dejarlos

[C-A04]: se lavan, pero nosotros le echamos una pastilla que es...

[C-A01]: para lavarlos sí.

[C-A04]: esa pastilla la vendía

[C-A01]: esa que utilizan para el tanque del baño...

[C-A04]: usted se la echa al baño nomás, eso es como un purificante del agua.

[C-A01]: lo de las plantas, eso sí lo utilizamos mucho

[C-A08]: rosado... rosado pa' qué

[INVESTIGADORA] eso es como rojo en el semáforo.

4:315 ¶ 1436 – 1437, in Comportamientos

6:192 ¶ 337, in Anexo 2 Transcripcion grupo focal de malaria y leishmaniasis con comunidad afrodescendiente.docx

[C-A06]: y sabe cuál está allí, la hija de doña Arenia, de pronto no porque cuando bueno... usted sí la distinguió, la hija de doña Arenia, ella una vez casi se mata con eso... estaba embarazada, ella ya tiene la niña, estaba embarazada y se tomó 7 le dijeron que se tomara, casi se mata esa muchacha. Lo que sí es que eso era super amargo esa...

4:317 ¶ 1443 – 1454, in Comportamientos

6:196 ¶ 388 – 398, in Anexo 2 Transcripcion grupo focal de malaria y leishmaniasis con comunidad afrodescendiente.docx

C-A06]: pues nosotros... por allá hay un niño que él vende... él vende como que un...

[C-A01]: ¿un paisita?

[C-A06]: sí, nosotros les echamos, lo combinamos con agua, le echamos la botellita de cloro y quedamos fumigando con eso, y eso mata... hasta esa [inaudible] se las mata.

[C-A04]: ¿cómo es?

[C-A01]: el peladito que vende los chorizos, que vende ese veneno blanco

[C-A04]: ah... ¿el que va en Chocho?

[C-A06]: no, él anda en moto. Nosotros lo combinamos con agua y le echamos cloro y con eso fumigamos. ¿Y lo bueno sabe qué es? Que fumigamos el patio y no mata las plantas

[indistinguible]

[INVESTIGADORA] pero entonces ustedes lo hacen, compran el veneno de esta persona o cualquiera y lo hacen en sus casa porque ya no hay... ya no vienen [inaudible]

[C-A04]: vale mucha plata

[C-A08]: hoy en día todo es un negocio.

4:322 ¶ 1473 – 1476, in Comportamientos

6:222 ¶ 355 – 357, in Anexo 2 Transcripcion grupo focal de malaria y leishmaniasis con comunidad afrodescendiente.docx

[C-A06]: yo lo que compro así en la farmacia es acetaminofén, porque eso le mandan a uno en el hospital

[C-A08]: y la farmacia de Aleida también falta por un lado.

[C-A01]: porque yo para pagar 7mil y pico en el hospital voy y compro mis cosas que me valen mil pesos. Cuando yo voy me cobran 7 mil seguro, cuando voy al hospital

4:324 ¶ 1480 – 1482, in Comportamientos

6:235 ¶ 739 – 740, in Anexo 2 Transcripcion grupo focal de malaria y leishmaniasis con comunidad afrodescendiente.docx

¿que podríamos hacer para que la gente, en serio, se tome el tratamiento que le manda el médico? [C-A04]: yo creo que es más que todo como es tan amarga y que son buenas, entonces claro, desde el primer momento el malestar le va bajando, entonces ya se siente bien, entonces ya en vez de tomarlo, es una... es varios días, no es igual a la bebida que es un día, amargüísima, sí, pero usted sabe que le va a [inaudible].

[C-A06]: que eso es un medicamento que no se puede tomar con hambre. [inaudible] le da mucha malaria y luego no había desayunado, le dieron el medicamento y lo mandó de una al hospital. Uno que está débil, no ha comido...

9:51 ¶ 171 – 172, in Corresponsabilidad

4:307 ¶ 1445, in INSTITUCIONES ML & LC.docx

[I-A03]: pues a veces por... no nos digamos mentiras, a algunos no les gusta el tratamiento, la verdad es esa e ir al... es que yo no me lo voy a tomar y traer medicamento y amontonar ahí...

9:55 ¶ 179 – 182, in Corresponsabilidad

4:381 ¶ 43 – 45, in INSTITUCIONES ML & LC.docx

¿El indígena está acostumbrado a usar repelente?

[I-M01]: No. Ellos no lo utilizan.

[I-A03]: porque dicen que no, porque... es que sí, es amargo. Porque es que son muy amargos, que es eso me molesta, pero igual antes de entregarlos, por eso se hizo... porque era que los mismos gobernadores pedían "venga, por qué no nos dan repelente", entonces... De pronto algunos utilizan, otros no lo utilizan...

12:8 ¶ 27 – 37, in Experiencias significativas

4:200 ¶ 1564 – 1573, in INSTITUCIONES ML & LC.docx

tratan de sacarlos.. eso les entendí, ya no es en la habitación sino al menos es afuera...

[I-I04]: parte de la cocina.

[INVESTIGADORA] afuera de la cocina?

[I-I07]: es algo a mejorar

[I-I04]: afuera de las casa, pero ahí mismo pegadito

[I-M01]: ahí mismo

[INVESTIGADORA] bueno y cómo creen que lograron eso

[I-M01]. de hecho nosotros hicimos un proyecto de casas sin humo en ese entonces, eso es digamos de 2015, donde se hizo y ya con las familias sacaron... fura de la vivienda, pero ahora se encuentra uno que otra vez tienen el fogón dentro de la vivienda.

[INVESTIGADORA] y qué les enseñaban ustedes o cómo era ese proyecto

[I-M01]: de sacar los fogones fuera de la vivienda con el fin de reducir enfermedades porque ese smog pues afecta la salud de la familia como tal, especialmente los niños que son los más propensos a sufrir la enfermedad, entonces con ese fin se hizo y sí... entonces ellos utilizan su [inaudible] para preparar alimentos, entonces... si el fogón está por fuera de la vivienda, ellos después de que llega la noche tienden a no salir de sus casas, pues por las creencias que ellos tienen. Entonces yo digo que a raíz de eso es que otra vez están volviendo a poner el fogón dentro de la vivienda.

○ Vacios de conocimiento_ML

28 Citas:

1:61 ¶ 190 – 193, in Conocimiento comunidad

4:365 ¶ 996 – 998, in INSTITUCIONES ML & LC.docx

[INVESTIGADORA] listo, en malaria, ¿conocen el vector? [I-A03]: muchos todavía no conocen

[I-M01]: no...

[INVESTIGADORA] ¿entonces le ponemos amarillo o rojo? [I-M01]: no, amarillo

1:69 ¶ 218 – 222, in Conocimiento comunidad

5:37 ¶ 284 – 287, in Anexo 1 Transcripcion grupo focal de malaria y leishmaniasis con comunidad indígena.docx

[INVESTIGADORA] ¿y entonces qué puede ser? [C-I07]: para matar malaria

[inaudible]

[C-I03]: porque si tomamos tantos medicamento se puede morir

[INVESTIGADORA] ¿y eso..? Generalmente eso pasa cuando uno se automedica, cuando uno mismo hace.

1:70 ¶ 223 – 227, in Conocimiento comunidad

5:42 ¶ 149 – 152, in Anexo 1 Transcripcion grupo focal de malaria y leishmaniasis con comunidad indígena.docx

C-I02]: ¿el dengue es lo mismo?

[C-I09]: la picadura del dengue es instantánea.

[C-I02]: el dengue no es hemorrágico?

[C-I09]: la síntoma es hemorragia, puede dañarle a uno el cerebro, puede causar un derrame...

1:73 ¶ 235 – 236, in Conocimiento comunidad

, y les da mucho mareo y para mirar les da ojo escudo

[INVESTIGADORA] ¿ojo escudo? [C-I08]: sí, le da ojo oscuro. Y le da mucha anemia, para parar les da como estrella en el ojo, les da esa... eso no más lo que tengo.

1:77 ¶ 246 – 247, in Conocimiento comunidad

5:57 ¶ 693, in Anexo 1 Transcripcion grupo focal de malaria y leishmaniasis con comunidad indígena.docx

[INVESTIGADORA] saben que es mosquito, pero ¿es el mismo mosco de la leishmaniasis, de la piña, que de la leishmaniasis? [C-I07]: hay varios mosquitos que son de leishmaniasis, que son la malaria, de cuál de los dos cuál es de leishmaniasis o de la malaria.

1:80 ¶ 253 – 254, in Conocimiento comunidad

5:67 ¶ 162, in Anexo 1 Transcripcion grupo focal de malaria y leishmaniasis con comunidad indígena.docx

[C-I07]: yo hice la casa por la ventana está entrando el mosquito para picarlo, que está durmiendo en la cama y tengo un charco producido los huevos de los zancudos y lo que produce, los puso como gusanito y luego, el mosquito viene a picar los animalitos como perro, los marranos... en estos momentos pues como yo tengo varias veces con los niños pues malarias, pues eso malaria es fuerte, algunos duran de primero cuando va a dar malaria dentro de cinco, cuando el mosquito puede picar a una persona y durante cinco días ya se pone a dar dolor de cabeza, les da escalofrío, les da malestar, no come bien, no alimenta bien las comidas y cuando ya eso... cuando uno en el síntoma que viene acá se produce como... unos animalitos

que viene acá y con eso ya se produce, ya se viene como tal fiebre, ya les da como más fuerte y tiembla mucho el cuerpo y ya casi uno no aguanta. Y a uno les da dolor de cabeza alta, le da como... cuando uno se para con los ojos se ve como borroso y le da diarrea, vómito, mareo, malestar, pero cuando uno [inaudible] y ese tratamiento, no dan cloroquina, si no le dan ese tratamiento, le dan una pastica que es como amarilla, eso es... dice que es un falcíparum y eso falcíparum está muy muerte, a los niños con ese falcíparum les está dando convulsión, eso es muy fuerte y a la vez también uno cuando se puede... durante 3 días si es un niño, durante dos días ya se baja de peso, el niño ya se pone como para desnutrido y eso cuando uno la mamita también tiene que estar pendiente una niña si está dando fiebre y vómito, hay que comprar un suero para que no baje de peso el niño, o si no, se puede desnutrir y eso, con esto malaria y el niño ya no queda como sino antes que vivía que era como débil, ya no come bien el desayune, no tolera bien hasta cuando no recupera bien, es como mínimo, cuando el médico le da el tratamiento, durante 3 días, tampoco no se mejora, eso queda como durante 20 días si el niño todavía está con malestar.

1:81 ¶ 255 – 258, in Conocimiento comunidad

5:69 ¶ 420 – 422, in Anexo 1 Transcripcion grupo focal de malaria y leishmaniasis con comunidad indígena.docx

[INVESTIGADORA] entonces, ¿todos estamos de acuerdo con que la gente sabe los síntomas de malaria? [C-I06]: no todos, algunos que no conocen, por ejemplo, otro síntoma, puede ser otro síntoma parecido, entonces uno acude siempre al hospital...

[INVESTIGADORA] ¿la mayoría sabe o no sabe los síntomas de malaria?

[C-I08]: yo no creo que no, porque siempre estará conocimiento de cada persona, que nadie no le sabe qué enfermedad tenemos, entonces es saber la mamá que eso es malaria y llega entremedio de la enfermedad, pero siempre el mal estado siempre está con la borrachera, está en... mejor dicho, eso llega a diferente enfermedad, por eso siempre dicen que es que es malaria, le dicen a la mamá, por eso siempre hay que estar llevando para el hospital

1:101 ¶ 322 – 323, in Conocimiento comunidad

5:144 ¶ 366, in Anexo 1 Transcripcion grupo focal de malaria y leishmaniasis con comunidad indígena.docx

[C-I03]: tengo una pregunta también, es que no entiendo, un ejemplo si... yo creo que hay dos animalitos que se terminen la enfermedad, un ejemplo, creo que los mosquitos tienen dos mosquitos creo pues porque yo pienso que solamente hay una mosca que produce la enfermedad, al leishmaniasis o puede ser el paludismo... entonces eso quiero saber cómo previenen la enfermedad si hay dos animalitos o hay tres...

1:104 ¶ 329 – 339, in Conocimiento comunidad

5:159 ¶ 346 – 355, in Anexo 1 Transcripcion grupo focal de malaria y leishmaniasis con comunidad indígena.docx

[INVESTIGADORA] bueno, ¿esto qué es?

[C-I01]: eso es como una picadura de malaria.

[INVESTIGADORA] ¿de malaria?

[C-I01]: sí porque ya... [inaudible] puede ser leishmaniasis, las dos cosas.

[C-I04]: leishmaniasis.

[INVESTIGADORA] ¿leishmaniasis?

[C-I04]: sí.

[C-I07]: malaria.

[C-I09]: puede ser malaria y leishmaniasis...

[C-I06]: porque son casi similares a esa enfermedad, sino que la malaria da otras síntomas y enfermedades y la piña da otros síntomas como grano y la malaria da es como los síntomas de la fiebre.

1:108 ¶ 348 – 349, in Conocimiento comunidad

5:173 ¶ 413, in Anexo 1 Transcripcion grupo focal de malaria y leishmaniasis con comunidad indígena.docx

[INVESTIGADORA] vamos a quitarlo... eh... bueno, ¿y uno cómo consigue el tratamiento para malaria cuando se lo toman solos? [C-I09]: lo que pasa doctora es que hay gentes que conocen el medicamento, entonces van y lo compran en la farmacia, allá lo venden, pero igual allá les explican que hay que tomar cuantos días, entonces los mandan explicaditos y de acuerdo a esa explicación a la farmacia vienen y hacen el tratamiento en casa y hay mucha gente que se ha curado de esa manera, porque no logra ir al hospital, puede ser una persona de muy lejana que de pronto no logra ir al hospital para el puesto, para poder ir al puesto con ese síntoma, entonces va y compra el medicamento allá, pero lo que es el tratamiento de malaria.

1:109 ¶ 350 – 351, in Conocimiento comunidad

5:174 ¶ 174, in Anexo 1 Transcripcion grupo focal de malaria y leishmaniasis con comunidad indígena.docx

[C-I02]: [lengua embera] tenemos pecera, poquita pecera familiar y también el embera nosotros como indio, no quemamos bota y si no que más bien lo tiramos en el patio, muchas cositas, charquitos alrededor de la casa [lengua embera] molesta la casa y ellos mantienen mucho en charquito, [lengua embera] bregan de entrar a la casa, ahí entonces repelente como decía el repelente, el embera nosotros no compramos, pero sí hace falta doctora, porque eso es prevención, [inaudible] eso afecta la malaria. Gracias.

1:115 ¶ 371 – 375, in Conocimiento comunidad

5:186 ¶ 357 – 363, in Anexo 1 Transcripcion grupo focal de malaria y leishmaniasis con comunidad indígena.docx

[C-I06]: todo el malestar general, pero por ahí yo veo que es como un zancudo como de malaria.

[C-I01]: la palomilla.

[C-I06]: la palomilla tenía que ser de otro estilo más diferente.

[C-I01]: la palomilla, que es como blanquito, más chiquitico.

1:122 ¶ 387 – 388, in Conocimiento comunidad

5:194 ¶ 176, in Anexo 1 Transcripcion grupo focal de malaria y leishmaniasis con comunidad indígena.docx

[C-I06]: yo... bueno a cerca de la malaria que hayan dicho los compañeros, yo no conozco pues como son los síntomas, pero he escuchado como enfermero. La malaria producen mucho lo que son los charquitos, en todos, en casi ocasiones en las basuras, porque en las basuras llenan muchos charquitos de esos de agua, que llueve y mantiene produciendo los huevitos ahí. Para poder alimentar la malaria, para poder criar los huevitos tiene que picar, tiene que tomar una sangre del ser humano o de algún animal, para poder producir los huevos y ese es el produce, el mejor charco es el que produce mucho... mucho mosquito de esa de la malaria, la síntoma cuando empieza a tener la malaria da dolor de cefalea, dolor de articulaciones, escalofrío, fiebre y dolor musculares y malestar general

1:133 ¶ 411, in Conocimiento comunidad

ya si hay un nuevo método de echarle cloro a los estanques a los pozos... o sea, es un método que estamos utilizando para matar los huevos.

1:135 ¶ 418 – 419, in Conocimiento comunidad

6:46 ¶ 25, in Anexo 2 Transcripcion grupo focal de malaria y leishmaniasis con comunidad afrodescendiente.docx

[C-A04]: en tiempo de invierno se reproduce mucho...

1:141 ¶ 429 – 430, in Conocimiento comunidad

6:66 ¶ 696 – 699, in Anexo 2 Transcripcion grupo focal de malaria y leishmaniasis con comunidad afrodescendiente.docx

[INVESTIGADORA] bueno y volviendo a malaria y leishmaniasis... [C-A02]: eh... tu preguntabas que si las personas apenas sentían los síntomas iban al hospital o... el dilema es que como ahorita hay tantas enfermedades, entonces le empieza un síntoma y uno es ¿será eso o no será eso? "yo voy a esperar a ver... puede ser un dolorcito de cabeza por estrés, por calor, alguna condición o algo" ya si pasa el día

y el dolor persiste ahí sí acudir al centro de salud, pero el dilema está es ahí, que uno acude, pero ahí ponen la barrera...

1:148 ¶ 449 – 450, in Conocimiento comunidad

6:89 ¶ 110, in Anexo 2 Transcripcion grupo focal de malaria y leishmaniasis con comunidad afrodescendiente.docx

[C-A02]: principalmente después de que se diagnostique que es malaria, acudir al médico para que le mande droga, las pastas, tomar eso para poder matar el huevo, porque eso deja un huevo dentro del organismo de nosotros, entonces para poder matar ese huevo, tomarse el medicamento

1:149 ¶ 451 – 452, in Conocimiento comunidad

6:92 ¶ 125, in Anexo 2 Transcripcion grupo focal de malaria y leishmaniasis con comunidad afrodescendiente.docx

[C-A05]: con relación al tratamiento, me gustaría entender algo que dijo la compañera y es que este dibujito... bueno, ya estamos hablando de lo de la malaria, ¿cierto? pero hay que tener algo muy importante aquí y es las vacunas, las vacunas que se le aplican a los niños y niñas, ya que estaban tocando el tema de algunos menores, de los 0 a los 5 años, para que estas vacunas contrarresten los agentes patógenos que entren al cuerpo... las vacunas no lo van a inmunizar para toda la vida, pero sí van a ser un ayudante para alivianar.

1:151 ¶ 454, in Conocimiento comunidad

iluminación de la vivienda y hago referencia por ejemplo a los espacios, ya sea que la casa esté pintada, ya sea en una color blanco o ya sea como vivo, eliminar las telarañas y todo ese tipo de cosas, porque en el momento en que el zancudo entra a los hogares, se puede esconder en las telarañas o en las partes oscuras...

1:153 ¶ 457 – 459, in Conocimiento comunidad

6:109 ¶ 117 – 118, in Anexo 2 Transcripcion grupo focal de malaria y leishmaniasis con comunidad afrodescendiente.docx

[C-A03]: yo tengo la duda que dijo [C-A02], que le pone ya el huevo a uno?

[C-A02]: él lo que hace es incrustar el huevito...

1:156 ¶ 470 – 472, in Conocimiento comunidad

6:124 ¶ 333 – 334, in Anexo 2 Transcripcion grupo focal de malaria y leishmaniasis con comunidad afrodescendiente.docx

[C-A04]: pero mire que la Aralem era muy bueno para la malaria...

[C-A06]: pero yo sé, lo descontinuaron

1:161 ¶ 483 – 487, in Conocimiento comunidad

6:139 ¶ 121 – 124, in Anexo 2 Transcripcion grupo focal de malaria y leishmaniasis con comunidad afrodescendiente.docx

[C-A06]: no es que el tratamiento del hospital vaya a matar para toda la vida, no, hay personas con suerte que les dio una sola vez y no les volvió a dar nunca más. Es como la lombriz, cuando los niños sufren de lombriz, usted llega y le dan remedio para que en un momento bote todas las lombrices que tenga, pero resulta y acontece que a medida que lo van alimentando se van produciendo más.

[C-A06]: no salen todas...

[C-A06]: no, el cuerpo es una [inaudible]. lo que hace el zancudo es que hasta que él no penetra el vaso sanguíneo, porque ni siquiera es la piel donde él va a picar, hasta que él no penetra el vaso sanguíneo, él no deposita lo que va a depositar y eso es una forma de dejar los huevitos para que no se me vayan a morir, que no se reproducen dentro no nosotros, porque ya el mosquito allá toma es otra medida, y ahí es donde... empieza a sentir esos síntomas porque el cuerpo siente algo extraño

[C-A04]: necesito que una especialista me aclare eso

1:165 ¶ 512 – 513, in Conocimiento comunidad

6:160 ¶ 174, in Anexo 2 Transcripcion grupo focal de malaria y leishmaniasis con comunidad afrodescendiente.docx

[C-A01]: ya el tratamiento de malaria es por 14 días [inaudible]

1:166 ¶ 514 – 518, in Conocimiento comunidad

6:165 ¶ 175 – 178, in Anexo 2 Transcripcion grupo focal de malaria y leishmaniasis con comunidad afrodescendiente.docx

las pastas lo mismo, ahorita solo son como 3 pastas que se toman, pero ya vienen más concentradas.

[C-A01]:no, al contrario, ahorita el tratamiento es más extenso el de malaria.

[C-A02]: ¿son más?

[C-A01]: son más, por lo general es por 14 días y dependiendo de la malaria que tenga, el peso, todo.

1:169 ¶ 525 – 526, in Conocimiento comunidad

6:179 ¶ 91, in Anexo 2 Transcripcion grupo focal de malaria y leishmaniasis con comunidad afrodescendiente.docx

[C-A03]: tener ya la vivienda bien arregladita, con el toldillito y las camas... buen jardín... los estanques que yo creo que todos dibujamos siempre ahí el estanque, porque por lo general en todas las casas uno tiene su pequeño estanque ahí, para tener sus pescados para el consumo, entonces ahí este estanque tiene que tener abundante agua y que el agua tenga corrida, porque si tiene poquitica el agua no corre, entonces a ahí es donde se van a recoger ahí... entonces tener suficiente agua.

Eh... yo he podido notar, por ejemplo, en mi caso que tengo tanta gallina y de todo que ellas... las gallinas recogen el mosquito. Uno se coloca a analizarlo y de una lo cogen y entonces creo que también puede servir...

1:176 ¶ 545, in Conocimiento comunidad

el mosquito le incomoda mucho lo que es la ventilación, por eso buscan lugares cerrados para poder reproducirse, entonces si la vivienda está ventilada, no van a tener cómo reproducirse. Y la fumigación periódicamente, mantener fumigando periódicamente lo más constante posible para evitar... y más que todo en época de reproducción de ellos [inaudible] en esos días hay que fumigar más.

1:184 ¶ 562 – 581, in Conocimiento comunidad

6:224 ¶ 841 – 859, in Anexo 2 Transcripcion grupo focal de malaria y leishmaniasis con comunidad afrodescendiente.docx

[C-A08]: es que lo pasa es que... hay tanta clase de zancudo... el primer zancudo que lo pique es el que usted mata, pero todo zancudo no da...

[C-A09]: ah, sí, de paludismo es uno solo

[C-A08]: de paludismo no es el de las paticas blancas

[INVESTIGADORA] no, ese es el de leishmaniasis

[C-A02]: no, el de las patas blancas es el paludismo,

[C-A08]: el de las paticas blancas es el paludismo, que usted lo mata y... ahí le queda la patica blanca y queda ahí.

[C-A01]: es que uno hace como que lo pican los zancudos sanos, porque a uno pican y pican zancudos y no le...

[C-A08]: sí, hay tanta clase de zancudos que uno no sabe como diferenciar cuál es el... el de la afectación.

[C-A01]: por ejemplo, hay un zancudo grande que dicen que ese lo pica a uno y lo mata, ese vuela así bobo.

[C-A08]: inclusive en estos días estaba leyendo algo de que ese mosco no se puede matar cuando se entra a la casa

[C-A01]: ¿ese grandote?

[C-A08]: sí

[C-A01]: uno no lo mata porque dicen que ese no pica.

[C-A08]: que uno no lo debe matar que porque él trata de hacer algo pues en bienestar de uno, ¿si me hago enteder? Ese zancudito uno no puede matarlo y uno todo lo mata porque uno piensa que todos los zancudos son...

[C-A01]: ese grande uno no lo mata porque dicen que ese no pica... él es así como... tiene las patas todas largar

[C-A08]: y yo creo que aquí nadie... aquí todo el mundo mata ese zancudo.

[C-A09]: ah sí, porque uno no puede ver un zancudo porque ahí mismo.

[C-A01]: yo no lo mato porque ya a uno le enseñaron que ese no pica, él vuela así todo bobo.

[C-A08]: y uno lo ve y lo primero que se hace es que asusta, esa ese es el papá de los zancudos.

4:220 ¶ 941 – 942, in Comportamientos

5:68 ¶ 634, in Anexo 1 Transcripcion grupo focal de malaria y leishmaniasis con comunidad indígena.docx

[C-I04]: una pregunta, ¿qué es repelente?

TUBERCULOSIS

○ Barreras de aptitud_TB

80 Citas:

8:28 ¶ 19 – 20, in Salud

2:81 ¶ 154, in Indigena TB.docx

[C-I05]: No, pero hay unos también si no, también muy largo la fecha de la cita. La dan muy largo. Yo la pedí el mes de abril y me llegó el diez de junio. ¿Y a eso uno enfermo qué? Hasta la fecha esperando qué [inaudible]. Yo he ido siempre a donde el médico y realmente el paciente lleva enfermo, con esa cita no se cura. ¿Qué se va a curar? Se muere. [inaudible] Que allá uno hace atender, pero, pero, metiendo candela y eso es muy maluco, pero toca así, porque si no se muere la familia de uno, mi hermanito murió así. Igualito. Vino a la consulta. No, usted que está bien, más o menitos, saque cita. ¿Cuándo? Dizque para tres meses. No, no dijo nada, mi hermanito era poca palabra, entonces se fue para la casa. Siguió la fiebre y allá le dio el derrame y se acabó de joder.

8:29 ¶ 17 – 18, in Salud

2:78 ¶ 132, in Indigena TB.docx

[C-I05] Con la práctica que hemos hecho, caso especial, hace dos semanas, una señora venía con un cólico fuerte. Resulta que la llevamos al hospital al centro de salud. El médico me dijo que yo te voy a tomar signo vital y si aparece signo vital, bien, normal, hay que pedir cita. Entonces yo devolví en esta manera: "Doctor, si el

signo vital normal, pero en dolor lo lleva ahí el paciente, ¿Qué hacemos? HÁgame el favor, consulten esa señora". Y me insistió otra vez: hay que sacar cita. Y teniendo el dolor ahí. Y yo no retire del hospital. Yo me quedé ahí. Hagamos una cosa. Yo automáticamente le llame al gerente. Es que él es un médico y a él tiene un padre quien manda. En ese hemos tenido error. Llamé al gerente, al grande allá. Me preguntó quién es esa, tal señor. Y el paciente es tal. Ah, entonces un momentico... Me atendieron. Me atendieron. En buena forma ya, porque yo me tocó.. Al ratico, en una observación se vomitó en un momentico, cuatro vómitos. ¿Qué tal en el camino se me vomita? ¿No se me muere? En ese hemos tenido, porque uno no tiene pacientes que no tienen conocimiento, lo que yo estoy hablando, no lo hablan así mismo. En ese hemos tenido error.

8:32 ¶ 25 – 28, in Salud

2:108 ¶ 102 – 104, in Indigena TB.docx

[C-I04]: El año pasado, acá en hospital que trabajé dos meses en la traductora, el niño era grave. Tenía mucha asfixia, venía de la comunidad de Piedra y ahí se pasó. Y la mamá lo llevó a hospital. Y el niño que estaba asfixiado, tosía, todo. Y el médico le mandó para la casa. No, que el niño que estaba bien, estaba bien. No, yo le dije a el doctor "como así que usted va a mandar para la casa, ¿no ve que el niño estaba grave? ¿No ve que el niño está asfixiada?". Y no, no. Ahí también me regañan porque no se puede hacer nada, no era su trabajo. Y por eso me quedé callada. Desde 3 días, lo mismo lo traía ella al hospital. Y en hospital, dice que "no, que el niño no está asfixiado, llévelo para la casa". Y la mamá también vuelve a traer pa' la casa. Y allá... cuatro veces lo llevó al hospital. Y lo llevó a Pereira. Y de Pereira lo llevó a Armenia. Armenia lo transmitió allá y en la mitad murió el niño. El niño que estaba grave. Y por eso ahí hicieron un paro. También allá en el hospital.

[INVESTIGADORA] ¿eso fue hace..?

[C-I04]: El año pasado, trabajé dos meses y ya se acabó mi contrato.

8:36 ¶ 39 – 42, in Salud

3:41 ¶ 410 – 412, in AFRO_TB.docx

[A01]: ah no, y le digo que las enfermeras también lo hacen...

[A07]: y ellas lo hacen porque como ven al médico hacerlo y como la comunidad no le dice nada al médico, ella se va adoptando esas malas costumbres.

[A01]: cuando yo trabaje en el centro de recuperación nutricional, nosotros tenemos un médico, pero contábamos con la doctora, que ella vive en la cabecera municipal, a veces el niño o algo le decíamos a la doctora, vea ella decía vamos a hacer esto y esto y vamos a mirar la evolución, vamos vigilar el niño o hay niños, los niños con desnutrición sabemos que es una enfermedad mortal, que tiene atención... una urgencia prioritaria, en cualquier momento se descompensa y llega uno a la seis y media con un niño y que hay... no tuvo todo el día para traerlo, por qué lo trae a esta hora. Entonces uno tiene que saber a qué horas el niño se va a

enfermar para llevarlo... eso es lo que le dice la enfermera... ¡ay! Por qué lo trajo... o uno a veces va a las nueve de la noche: "a esta hora no hay médico, por qué no lo trajeron temprano que están los médicos, ahora qué quieren qué haga a esta hora, yo le tomo los signos y le digo al médico a ver él qué dice".

8:39 ¶ 131 – 132, in Salud

4:249 ¶ 1422 – 1425, in INSTITUCIONES ML & LC.docx

[I-I02]: cuando está enfermo dicen que tienen que pedir cita y dan para un mes, para dos meses así y mientras... se muere uno, el niño [inaudible]

8:54 ¶ 69 – 70, in Salud

4:3 ¶ 273, in INSTITUCIONES ML & LC.docx

[I-M01]: entonces se lo llevan, no... enfermera que el niño sigue enfermo, otra vez, devolvámoslo. Hagámosle el acompañamiento para ver si así nos lo atienden porque no sé si es que ellos no saben hablar, no saben explicar o no se les presta la atención que se debe, entonces se les hace el acompañamiento para que ese niño sea atendido y le manden pues la fórmula, explicarles a ellos muy bien cómo vigilar esa fórmula, porque a veces "ah bueno papito, el niño está ah bueno, papito, el niño tiene una infección gastrointestinal y esta es la fórmula", quizá no se le explica cómo se le debe dar el medicamento y se lo llevan para la casa, no saben cómo darle el medicamento. O se lo explican y no se acuerdan, entonces eso es como desde esa parte. Entonces ellos se van como desmotivando.

8:63 ¶ 102 – 103, in Salud

4:118 ¶ 281, in INSTITUCIONES ML & LC.docx

[I-I07]: el niño que falleció, primero llevaron al puesto de salud y después, al otro día, llegó de Sallada [inaudible] tenía... eso es lo que quiero explicar, ellos mandan para la casa ¿por qué? Porque el niño se pone peor y cuando llega a Pereira y allá se muere. Eso es lo que pasa. Porque ese niño tenía malaria, desnutrición, tratamiento también, pero como le digo, mandan para la casa al otro día.

8:68 ¶ 178 – 179, in Salud

4:379 ¶ 1412, in INSTITUCIONES ML & LC.docx

[I-A03]: porque a mi me pasó que día que una recaída y yo llegué que no era capaz ni de dar el paso y me dice un auxiliar ah es que ya fichos no hay y no la pueden atender y yo que pena con usted pero el problema que... a mí, si yo voy así me deben atender porque mi caso porque mi caso no es cualquier cosa y si aquí no hay nadie... o sea, yo sé que aquí, de pronto los primeros auxilios y váyase, ya... pero es que no, que yo no la puedo antender porque ya no hay ficho y el médico está muy ocupado, entonces yo le dije bueno, listo, me acerqué al médico, me dijeron, le comenté mi caso particular y le dije mire que me pasa esto y esto y dijo suspendan lo que estén haciendo y me la atienden, el mismo y a los 3 días me tuvieron que hospitalizar. Entonces el problema no es que yo no la puedo atender, el caso es que

hay que priorizar. Hay que priorizar porque si una persona está en muy mal estado de salud y si, por ejemplo, si usted está más mal y yo estoy más estable, atiendan a usted y depsués me pasan a mi, pero hay casos que no dan espera, no dan espera. Entonces...

9:6 ¶ 21 – 22, in Corresponsabilidad

1:66 ¶ 535, in Instituciones_TB.docx

[I-M08] Yo he pensado siempre que una de las soluciones es no darles todo, como se lo da el gobierno. Yo pienso es como por los resguardos, por comunidades, darle como más agentes comunitarios, en comida darles... no darles el mercado, darles un ingeniero agrónomo... entonces en este resguardo o para tantas comunidades y empezar a hacer truque, que ellos sepan, que hay que cultivar para comer, no que todo es regalado, porque es que ahí donde va. Y a todos nos pasaría, yo creo que si a mí me dicen ven [I-M08], te doy el millón de pesos que te ganas y no haces nada, yo no hago nada, me quedo en la casa. Entonces pasa con ellos pienso yo

9:9 ¶ 27 – 29, in Corresponsabilidad

1:144 ¶ 271 – 272, in Instituciones_TB.docx

[I-M08] Pero yo no entiendo, entonces, por qué vienen después a decir que el hospital es el culpable de todo.

[I-A03] O sea, porque esa es como la percepción que ellos tienen...

9:11 ¶ 37 – 40, in Corresponsabilidad

1:156 ¶ 266 – 268, in Instituciones_TB.docx

[I-M01] Yo creo que de pronto también a veces son despreocupados, una falta de consciencia. Por ejemplo, hoy, vinieron a cobrar lo del incentivo que están, o sea, sí, estuve por ahí cerca y una niña tuvo sangrados nasales, ¿cierto? Y la mamá no la quería traer al hospital, "—no, es que yo tengo que cobrar. — no mamá, primero lleve la niña. — No, que no, que no y que no".

[I-M08] y usted no los puede obligar, qué más hace uno.

[I-M01] Pero se le dieron opciones [inaudible] la pasamos allá adelanta para que la atendieran de los primeras, pa que ella pudiera venirse, pero de no ser así, ella no viene. Entonces como falta también de...

9:16 ¶ 53 – 54, in Corresponsabilidad

1:186 ¶ 484, in Instituciones_TB.docx

[I-I04] ellos decían: si se van para el hospital allá los matan [Risas]. Eso decían, por es ellos... les daba a todos, pero quedaban reservados en la casa.

9:17 ¶ 55 – 56, in Corresponsabilidad

2:1 ¶ 119, in Indigena TB.docx

[C-I02]: A la vez la doctora no es de acá, es de otro departamento. Ella es cauquenia. Entonces no está bien... ...no está muy de mano con el resguardo Chamí Embera Unificado, nos está desconociendo, ella es muy cascarrabia. Cuando ella se está enojando, ella no quiere a los indígenas.

9:18 ¶ 57 – 58, in Corresponsabilidad

2:9 ¶ 143, in Indigena TB.docx

[C-I05]. O sea, otra importancia que hace anitos, hace añitos, médicos que tenían, o sea, confianza a los indígenas, que decían "— Quenverde, ¿dónde duele? — acaí, acaí". Entonces decía "a dónde", entonces el indígena tocas, donde duele ahí, toca el indígena ahí. Entonces, ellos ya el médico ya tenían esa confianza. ¡Poropira! ¡Poropira! ¡Dolor de cabeza! ¡Acaí! acá. El médico ya recetaba... la confianza. La confianza era inmensa, era confianza. Pero ahora no hay confianza.

9:19 ¶ 59 – 60, in Corresponsabilidad

2:19 ¶ 107, in Indigena TB.docx

[C-I02]: Ahí en el centro de salud llegan médicos. Médicos vienen a hacer rurales. Tiene para práctica. No conoce todo lo que es médico abarca de trabajo. Como es nuevo, está practicando, es rural. Pero el médico hay un fallo. Y hay un médico de buena gente. Lo quieren a los indígenas. Hay un médico que no quieren a los indígenas, lo rechazan. Hay dos cosas a jefe. Ahí nosotros vemos la debilidad del Centro de salud. Hay un médico que quieren mucho al Embera. Hay unos, no todos. Y hay unos... Tratan muy poco. No socializan. Más bien dice que esto está bien y esos niños han muerto en la casa. Una vez murió una... No quisiera contarle, pero recordemos. Mi nietica así murió en Santa Cecilia. Venía con asfixia, de la casita venía con asfixia esa nochecita. Vino al médico y no lo atendieron. Dijeron que espere, que espere y en la puertica del Centro de salud murió la nietecita. Y no los demande [inaudible].

9:20 ¶ 61 – 62, in Corresponsabilidad

2:43 ¶ 111, in Indigena TB.docx

[C-I03]: Yo creo que el [C-I05] sabe, [C-I02] sabe... ...lo demás enfermeros saben. Un ejemplo, en buzón de sugerencias, Si uno ya escribe, un ejemplo, si yo escribe y pasa el buzón de sugerencia. Y eso como fue... ...y quién va a mirar o el... el gerente. Porque ya lleva cuatro veces que yo siempre... una cartica partida... haciendo... y hay dos, una negra de anterior... ...y hay una persona que contratado... ...ya es nuevo. Ellos siempre meten grosero. Uno cuando va ahí siempre meten grosero... ...por eso yo escribí para que...

9:21 ¶ 63 – 64, in Corresponsabilidad

2:58 ¶ 133, in Indigena TB.docx

[C-I03]: El médico maltrataba por la palabra. Entonces si mujer avispadita o sabe hablar, enfrentar a uno, uno cuando ya habla, ellos pueden entender normalmente.

Pero si uno queda así... Un ejemplo, usted me habla muchas cosas y no entiende, Me queda así. Así como nosotros, usted cuando yo hablo con mi lengua, usted no va a entender, se queda así. Y nosotros también así mismo. Así mismo. Así cuando ya una mujer no entiende palabra en español, uno hablando, mal tratando por la palabra, uno que está hablando, uno piensa eso. Así me paso de en Santa Cecilia. Una mujer que vine y este... la enfermera, una negrita que trabaja allá, ella había hablado mal de mí a la señora y al paciente. Y yo escuchando ahí, al lado de ella, escuchando, al último me tocó hablar, porque hay que respetar a la señora. Ellos no entienden tu palabra, pero tienen que respetar, ella es paciente, usted es un enfermero, pero usted también tiene su jefe que manda. Entonces quedó callado y se fue y consultó a la médica. Y eso pasa.

9:22 ¶ 65 – 66, in Corresponsabilidad

2:60 ¶ 105, in Indigena TB.docx

[C-I01] uno cree que hoy en día de las mujeres, por qué razón no lo quieren traer al hospital. Yo creo que varios años atrás, siempre pasaba así. Este año ha pasado así. Porque hay muchas mujeres, porque [inaudible] no traen cuando un niño está en enfermo, porque no les gusta eso, porque hay veces los médicos solamente lo miran, que el niño está bien y lo mandan a la casa. Y por eso la mujer casi no le gusta, que no, que el médico... y lo mandan, ¿para qué lo van a llevar? Para eso tratan con Jaibaná y lo pasa con mejor trabajo [inaudible] familia. A los último, los enoja uno. Es que el médico no se mide y por qué van a ir más allá. Siempre hay una mujer que lo tratan así. Porque eso... es un mundo porque.... No hay buena atención.

9:25 ¶ 74 – 75, in Corresponsabilidad

2:149 ¶ 110, in Indigena TB.docx

[C-I02]: Es que en el hospital hay unos médicos muy groseros. Sí, muy grosero. Yo estaba la semana pasada, había estado allá, al hospital, que una señora llegó del Chocó... que una niña que tiene mucho brote en la cabeza. Y habló que no, que mantiene muy cochino, que no... Yo no quedo... Que yo le dije al señor ¿Y porque no tratan así a usted? Y como dicea ellas ¿Y usted por qué no [inaudible]? [inaudible] cómo así que van a decir que hay un [inaudible]. No puede tratar así, yo les dije a usted le faltan al respeto. Y la mamá, como ella no sabe hablar... de Choco hay una mujer, allá casi no se habla. Y también que no... que del Chocó son muy brutos que no saben hablar. Y no entienden nada. Ahí me cogió la raya, yo iba a hablar, pero yo les pego mi carpeta y ellos meten el buzón de sugerencias. Todo la parte... uno entiende nada o si no hace caso y a veces no quieren atender. Eso pasa ahí en el hospital.

9:28 ¶ 87 – 88, in Corresponsabilidad

3:64 ¶ 254, in AFRO_TB.docx

[A06]: yo que día llevé a mi mamá, en la noche y lo primero que me dijeron fue "y usted por qué no vino más temprano". ¿Y es que a ti una urgencia te dice voy? La

sola urgencia, ah, no es que el médico ya se fue. Y que usted tiene la presión alta y qué por qué la tiene. Oiga niña, la tiene alta por el dolor, el [inaudible] y los signos vitales que se alteren. Es que “tome este” reporte y le dije "yo para qué me lo llevo, si yo no le voy a dar ningún medicamento, si la tiene alta, si la tiene en tanto por tanto, yo no le voy a dar ningún medicamento [inaudible]”. Hay otros días que usted va y ya le digo, usted amaneció como con dios o yo no sé, pero vienen y lo atienden ya sea aquí en Pueblo Rico, Pereira, en cualquier departamento. Esa [inaudible].

9:31 ¶ 102 – 106, in Corresponsabilidad

3:82 ¶ 406 – 409, in AFRO_TB.docx

[INVESTIGADORA] y tiene, porque ayer hablábamos de que un problema en el hospital era que venía gente afuera y había una relación tensa y con los tratos a veces, pero... o sea, qué porcentaje de la gente del hospital es de afuera...

[A01]: más que todos son los médicos...

[A07]: pero los médicos vienen pensando que somos una comunidad ignorante, una comunidad no educada y así nos tratan. No es el respeto con el que te trata un médico de la Clínica los Rosales o el Pinares Médico... no. "Pase a ver... usted no tiene nada, váyase para su casa".

[A02]: tiene que escoger el día y la hora en que se va a enfermar, porque si usted se enferma un domingo, llega con un dolor de cabeza, un malestar, "ah, no... eso es un guayabo"

9:32 ¶ 107 – 119, in Corresponsabilidad

3:133 ¶ 413 – 424, in AFRO_TB.docx

[A07]: Por esa actitud, yo perdí a un hijo. Por esa actitud, el 15 de octubre en el 2021 perdí a mi hijo. Porque desde el 8 consultamos porque ella tenía dolores de parto y ya estaba en los días y llegamos a la... bueno el médico dijo "se va a que es por cesaria" pero llegó el de Pueblo Rico y la devolvió "no, usted no tiene nada, para su casa" y el 12 volvimos y eran las 7 y media y me acuerdo de lo que dijo la enfermera, una persona que trabajó conmigo, una persona que a mí me [inaudible] y dice "siga está bien" ¿y qué hizo? Abrió la puerta malagana a las 7 y media de la noche: "yo no sé a qué vienen aquí si a esta hora no hay médico a trasnocharla a uno".

[A01]: lo primero que le dicen a uno: “¿por qué no vinieron temprano cuando estaba en el médico?”, es el saludo que le dan, por qué no vinieron temprano cuando estaba en el médico.

[A07]: llamó al médico de malagana y le dijo la información como no era. Que ella tenía cólico y que tenía estreñimiento. El médico dijo "devuélvala para su casa". No dijo que eran espasmos abdominales que eso también es síntoma de parto, no, ni que estaba dilatando, no, ella no dijo eso. Yo no estaba aquí, yo estaba en una capacitación y cuando yo llego me cuenta todo eso y yo me enojé.

[A02]: y ella cómo sabía que era estreñimiento si le hizo una palpación...? Solamente la miró y dijo: ¿es esto?

[A01]: es que se supone que si una mujer ya llega en los días de trabajo de parto, hay que hacer, hay que reportarle al médico y hay que hacer el tacto. Hay que hacerle el tacto, reportarlo y remitir.

[A07]: lo primero que dicen es "¿A qué EPS es?" "— Medimás — ah, no, a Medimás no le gustan esos casos" y la devuelven. "no, no, no, no se puede". Sí, así es. Pero cuando llega un docente que dice ahí COSMITEC, inmediatamente el CHIP cambia. [Inaudible] Estos son los que facturan y dan la plata. O otro más perverso, accidente de tránsito, no saben dónde ponerlo... Pero cuando es subsidiado, Medimás que está en la quiebra, que le debe miles de millones... ¿cuál fue el resultado? en la mañana del 15 ya dijo "yo no aguanto más", llegamos con 6 de dilatación, la subieron a la ambulancia, no medicada, sabiendo que ya tenía 6 [inaudible] era de este color el líquido amniótico. Cuarto grado de meconio, mi hijo murió ahogado. Comió meconio y lo respiró. Entonces mire la secuencia: si hay un programa de salud, si hay unas rutas que atender, en lo que sea en lo que nos estemos imaginando, en lo que estamos haciendo hoy y desde la institucionalidad no se le presta la debida atención ni se sigue protocolo diseñado para eso, nunca va a haber un resultado positivo. Nunca. ¿A qué conlleva eso? A que cuando le hicimos ese... para cambiar a la gerente del hospital no había una sola prueba de que ella estuviera haciendo mal su trabajo ¿por qué? porque ni los afros ni los indígenas demandamos. No demandamos.

[A01]: sí, eso dijeron que no había demanda, ni nada...

[A07]: no hubo una prueba, no tuvimos una prueba para sacarla

[A01]: qué pasa a las cosas y nadie demanda.

[X] se hace búlla y ya. Ahí muere.

[A01]: un día de huelga y ya

[A07]: entonces uno como líder... hoy el hospital ya tiene cinco demandas incluyendo la mía y como a mí no me interesa los 1000, 2000 o 50 pesos que lleguen por eso. Es el antecedente. Es el antecedente, con ese antecedente, el nuevo gerente que llegue, porque este ya se va en marzo del próximo año ah, él sí se va a poner la atención y va a decir que no quiero que me sancionen, yo no quiero que mi trabajo aquí se entorpezca porque todos los recursos que llegan son para pagar demandas, ahora sí voy a mirar por eso.

9:33 ¶ 120 – 122, in Corresponsabilidad

3:149 ¶ 268 – 269, in AFRO_TB.docx

[A06]: allá hacen casa y cocina, más si llegan urgencias eso terminan de excusarlos, y pueden haber dos o tres médicos y se demoran para darle una acetaminofén. Por eso es que uno se automedica y da hasta pereza ir allá.

[A02]: a mí sí me da mucha pereza ir la verdad.

9:34 ¶ 123 – 124, in Corresponsabilidad

4:10 ¶ 1452, in INSTITUCIONES ML & LC.docx

[I-I07]: pues la población indígena digamos que no interesa digamos los íntomas, pero como te dije ahorita, recurso, distancia, [inaudible] y a veces no apoya los padres de familia, a veces como los papás,cierto? A veces pasa eso [inaudible] sí pesa mucho que... y también las demás [inaudible] es por eso... como... explicar... no dan ganas de ir al hospital por esos motivos, a veces y también da pereza de caminar a pie sin plata, sin pasajes, eso pasa. Pero cuando a veces, A VECES, uno compra un medicamento, algnos son juiciosos, llevan tratamiento. Pero cuando yo veo que no me importa nada salud o compra medicamento, si no le dan al niño, pero eso cuando compran los medicamentos sí le dan sus medicamentos o hacen tratamiento con medicina así, ¿sí me entiende? Es así de sencillo

9:35 ¶ 125 – 127, in Corresponsabilidad

4:18 ¶ 1493 – 1501, in INSTITUCIONES ML & LC.docx

"tiene que llevar al niño enfermo" y no lleva de pornto es que no les creen. ¿Qué creen ustedes? ¿eso puede pasar o no?

[I-M01]: si, se ha visto muchos casos, claro.

9:36 ¶ 131 – 132, in Corresponsabilidad

4:20 ¶ 233, in INSTITUCIONES ML & LC.docx

[I-I07]: Bueno, ese niño de Dokabú era del programa, y él pertenecía a esa comunidad, pero en esa parte, pues el médico y el demás equipo de trabajo, siempre dan una recomendación a los padres del hijo: que lleve al hospital después de tratar con el jaibaná. Si jaibaná no puede, si con el tratamiento ya no mejora la salud del niño, inmediatamente hay que llevarlo médico, al puesto de salud, para que el médico valore cómo está el niño y qué medicamento le den, pero a veces lo indignan, no hacen caso. Simplemente dicen no hubo plata, no tengo pasaje, eso es lo que pasa. Y ahí el niño va empeorando la salud, eso pasa acá, en Dokabú, siempre damos la recomendación con [inaudible]: lleve al puesto de salud y decía que no, que falta el tratamiento con Jaibaná y después mira el encuentro con la nutricionista y la psicosocial y observa que ese niño [inaudible] era muy tarde y nos toca hacer un compromiso con el papá y como el psicosocial hizo como algo...

9:38 ¶ 135 – 136, in Corresponsabilidad

4:59 ¶ 1450, in INSTITUCIONES ML & LC.docx

[I-I04]: por la distancia también, como ahorita...

9:39 ¶ 137 – 138, in Corresponsabilidad

4:80 ¶ 1458, in INSTITUCIONES ML & LC.docx

[I-M01]: ellos digo yo que no les gusta como mucho desplazarse a muchos lugares, entonces qué dicen ellos: el niño está mal, lo traen al hospital y obviamente lo tienen que remitir, entonces muchas veces las mamás, como es la mamá la que tiene que ir con el niño, ella puede que le preocupen los demás hijos o hasta el mismo esposo, dejarlo solo en la casa, entonces ellos como que priorizan la demás familia y no piensan como en las consecuencias de si yo dejo el niño en la comunidad se me puede hasta morir, ellos no...

9:40 ¶ 139 – 140, in Corresponsabilidad

4:81 ¶ 1410, in INSTITUCIONES ML & LC.docx

[I-I02]: el niño tiene malaria y el médico no quiso atender el niño, entonces yo lo iba a traer y él no quiso atender y yo hablé por urgencias al otro médico que era más formalito y él sí atendió, ese médico no quiso mandar a ese niño con malaria. Entonces él mismo atendió y mando otro medicamento para la casa y dijo que pidiera cita, el otro y a veces uno [inaudible] entonces eso pasa algunos.

9:41 ¶ 141 – 142, in Corresponsabilidad

4:94 ¶ 1461, in INSTITUCIONES ML & LC.docx

[I-I07]: a veces es miedo de ellos y ya ellos... dicen que no hay recurso para comprar pañales, difícilmente, por ese motivo, eso que ellos... esa parte y uno como...

9:42 ¶ 143 – 144, in Corresponsabilidad

4:131 ¶ 237, in INSTITUCIONES ML & LC.docx

Lo otro es que las autoridades deben asumir el rol de autoridad. No es simplemente yo soy cabildo, no, ellos tienen su junta directiva, tienen justicia, tienen gobernadores locales, tiene gobernadores mayores. Pero yo he evidenciado de que ellos ponen de cabildo a cualquiera. Me explico. Un ejemplo, yo soy el que más habla en las asambleas, entonces yo soy cabildo, simplemente porque yo soy el que más hablo, yo no tengo ni idea de pa' qué sirve ser cabildo o cuales son las obligaciones de ser cabildo. Porque ser cabildo no es ser cabildo, eso tiene una responsabilidad legal, porque él es el representante legal de ese territorio. Entonces mientras ellos no asuman ese rol, de que yo, por ejemplo, [I-I04] es cabildo, yo le digo "Cabildo vea, ese niño está enfermo y la mamá no lo quiere sacar" "ah, es que la mamá no quiere". No, yo soy autoridad. "Justicia, haga favor, me retira al niño y me lleva", pero no ejercen esa autoridad. Entonces, si no ejercen esa autoridad, el papá está libre de hacer para lo que a él le convenga y hay muchas excusas. Muchas excusas. No hay recurso, pero, resulta que muchas veces usted le dicen "bájelo, yo le doy los pasajes", "no pero hoy no puedo, mañana". Entonces ya lo llevan mañana cuando el niño ya está complicado y no pasa nada. Y se murió el niño y no hay una investigación a no ser que sea una entidad que pida un informe, no hay... o sea... nada, a no ser que sea miembro de algún programa que esté vinculado al informe, pero en últimas, no hay una responsabilidad sobre nadie. Aun conociendo de que hay una sentencia, pero no hay responsabilidad, si usted llama

las autoridades que vayan a las mesas, no van, están ocupados, no delegan. Entonces hay responsabilidad de las instituciones, hay responsabilidad de los cabildos

9:43 ¶ 145 – 147, in Corresponsabilidad

4:140 ¶ 1462 – 1463, in INSTITUCIONES ML & LC.docx

¿Qué... por qué lado puede irse más la cosa?

[I-M01]: ellos tal vez creen que no les va a pasar.

9:44 ¶ 148 – 149, in Corresponsabilidad

4:141 ¶ 237, in INSTITUCIONES ML & LC.docx

No sé cuál es la percepción que tienen dentro de los resguardos con respeto a la muerte de los niños, con respecto a la muerte de las personas en el territorio, porque ellos conocen las consecuencias, porque no es que ellos no las conozcan, ellos conocen las consecuencias de que si el niño está malito, tienen que sacarlo o si no se les va a morir, y ellos saben que si el niño está enfermo se les va a morir. Pero entonces, es más fácil culpar... Es la percepción personal, ¿cierto? Entonces duró 7 días en jaibaná, lo trajeron, el niño está muy grave, tiene que llevarlo a Pereira, pero la percepción de que se lo lleven apara Pereira es que allá los dejan morir. Pero entonces, yo como familia, yo como padre de familia, ¿por qué dejé llegar al niño a ese estado? No, la percepción es: lo llevo a Pereira y allá se muere o muchas veces no lo llevan, porque ellos no tienen la percepción de la enfermedad del niño, sino de ellos, como padres, y a mi allá quién los pasajes... y a mi allá... Y a mí allá... y a mí allá... y a mí allá... exacto no visualizan la enfermedad o el estado de salud del niño. Entonces eso ha sido una barrera grandísima, por más que uno les dice que en Pereira hay hogar de paso, que usted les dice las cosas. Entonces, muchas veces, estando en el puesto de salud, porque cuando yo era coordinador, les decía los vamos a llevar a Pueblo Rico y ¡Pum! Se volaban... y si los vamos a llevar a Pueblo Rico es porque el niño está maluco, no lo van a llevar a pasear, pero ellos se vuelan. Entonces es como que priorizan primero sus necesidades de padres, a el estado de salud del niño. Y eso pasa con todo. Si usted le dice, el niño tiene malaria,hasta que el niño no está titilando de fiebre y del IRA y que está que... pero ellos ya conocen los signos de malaria. Entonces eso es algo que... algo que tenemos que ir... sacando de la cabeza a ellos de que sepan de que... de que tiene que haber una consecuencia. De que si yo, papá, no lo saco debo tener una responsabilidad. No estoy diciendo que lo metan preso y la justicia ordinaria... aunque estamos violentando los derechos del niño, que son derechos fundamentales y están por encima de autoridades y están por encima de todo. Pero no sé si el estado es... le es mucho más fácil hacerse el... ¿cierto? Exacto y decir "no, ellos tienen justicia propia, son autoridades, ellos allá manejan..." pero entonces dónde están los derechos fundamentales de los niños que están por encima de todo. Y mientras ello no ocurra y mientras ellos sepan de que el cabildo no es simplemente mandar, y que del cabildo no es simplemente decir vea trabaje usted por puestos... mientras no haya una responsabilidad en ellos, no va a pasar

nada y eso no va a cambiar. A parte de que hay un brote de diarrea impresionante y hay mucha malnutrición por la diarrea.

9:46 ¶ 152 – 154, in Corresponsabilidad

4:148 ¶ 271 – 272, in INSTITUCIONES ML & LC.docx

Los niños se enferman, los papás llevan los niños al hospital, llegan al hospital... Ah, en el momento que el niño llega, primero prueba de malaria, porque ahorita es eso, primero prueba de malaria, lo primero así el niño lleve diarrea, bueno, otros síntomas, lo primero es prueba de malaria porque se han visto casos, ¿cierto? El papá, ahí está motivado en llevar ese niño... Porque ellos no son de que se enfermó el niño y corramos al hospital, pues no. Ellos no. Si ellos llevan al niño al hospital es porque nosotros, como operador, le decimos, cuando el niño presente estos síntomas, llévelo al hospital. Entonces, para ellos es desgastante quizás que ellos, bueno, motivados por nosotros, lleven el niño al hospital y que en el hospital, cierto, se le atiende al niño una urgencia siempre y cuando los signos de los niños estén alterados. Si de los signos está bien, para la casa. Pida una cita y para la casa, ¿cierto?. Entonces ellos se desmotivan. Si yo llevo a mi niño al hospital es porque está enfermo, pero sí me lo regresan para la casa, entonces, se van a sentir aprerezados de llevar los hijos al hospital, entonces casos se han visto. Entonces le dicen al papá "papito hay que pedirle una cita", ¿para cuántos días? Las citas están super demoradas.

[I-I02]: un mes, dos.

9:47 ¶ 155 – 162, in Corresponsabilidad

4:228 ¶ 1398 – 1404, in INSTITUCIONES ML & LC.docx

tiene que llevar el niño al médico, el niño está mal, usted está mal y no lo hacían, como si no quisieran llevarlo, ¿por qué creenq ue pasa eso?

[I-A03]: falta de voluntad pienso yo

[INVESTIGADORA] falta de voluntad, ¿qué más?

[I-I02]: falta de recurso

[I-I07]: y a veces los papás dicen que si llevo al hospital, no atienden mi hijo urgente, tengo que esperar una hora, dos horas, eso es lo que pasa casi la mayoría. Dos cosas: recursos y la demora y no atienden correctamente.

[I-I04]: ellos dicen si van a al hospital los medicamentos, primero que van a dar es acetaminofén, ellos dicen.

[I-I07]: y la acetaminofén la compra en la farmacia, es lo que dicen también a veces.

9:54 ¶ 177 – 178, in Corresponsabilidad

4:350 ¶ 1457, in INSTITUCIONES ML & LC.docx

[I-I07]: falta de interés, cuando uno va, se puede ver la falta de control y las vacunas, ¿no? Siempre me dicen no tengo plata con qué, entonces yo les digo, yo los llevo en la moto, yo colaboro y ya... tiene la posibilidad, entonces también por el interés [inaudible] uno colabora, se colabora, ahí llevo en la moto conmigo, pero también [inaudible] llecarlo solo a ellos o sola... y tampoco no caminan, algunos sí caminan, pero alguno sno, todos no, les da pereza, no sé y a veces dicen ah que mi marido está cuidando a mi hijo que yo por qué...[inaudible] que mi esposo no está haciendo nada, que no está apoyando al niño que está enfermo, que le está dando plata y las mamitas quedan ahí, paradas, así es como lo que yo... en el programa, eso comentaban

9:59 ¶ 192 – 194, in Corresponsabilidad

6:32 ¶ 689 – 690, in Anexo 2 Transcripcion grupo focal de malaria y leishmaniasis con comunidad afrodescendiente.docx

ustedes creen que también hayan como problemas tal vez con el conocimiento de la gente como en identificar esos síntomas y saber cuando hay que ir o sí van y siempre es que no los atenden. Bueno, ya hemos dicho que pereza, que a vedes les da pereza.

[C-A01]: la verdad es que le dicen a uno que consulte al otro día. A mí me pasó. Yo tengo una niña de 7 años, desafortunadamente ella me nació enfermita, ella me nació con un asma, todavía la tiene. Una vez, a ella le dan las crisis de la nada, igual es un asma no especificada, los médicos no saben qué... y una vez le dio una crisis así y ella cuando le dan las crisis ella como que se queda así como dormida y bastante agitadita, cuando eso estaba el doctor pediatra y yo la lleve al hospital así tapadita en la tarde, y le dije doc, ya era como las 5, y yo le dije "yo entiendo — por eso le digo que hay mucha falta de persona también, que ya un médico cansado, haber atendido 40, 50, personas en el día también es mucho cansancio, pero si llega alguien suplicándole a uno, quizá uno también debe ponerse la mano en el corazón, yo le dije— Mire, la niña está muy mal" y me dijo "no, tráigala mañana, que ya no estoy haciendo más consulta y todo" y pero mire y no me escuchó, me tocó pedirle el favor a la hermana, a el hermano de la señor que me llevara a Pueblo Rico, la niña de una vez para Pereira y en la UCI 19 días, entonces mire, si b yo no hago la gestión la niña se me mure y fui al hospital...

9:60 ¶ 195 – 206, in Corresponsabilidad

6:108 ¶ 648 – 658, in Anexo 2 Transcripcion grupo focal de malaria y leishmaniasis con comunidad afrodescendiente.docx

[C-A02]: yo sentía que me hacía así, así, así, fue tanto que yo empecé a hiperventilar, cuando me dijeron acuéstela ahí en la camilla, dijeron, acuéstela ahí en la camilla, fueron y le tocaron a la doctora, ah no, yo estoy ocupada, cuando por ahí llegó, atendió a un paciente que llegó por una consulta, para que le leyera unos exámenes, eh, lo atendió y ahí sí salió "qué es lo que tiene", entonces yo no podía hablar, entonces mi hermana, mi mamá y mi prima que estaban ahí, dijeron que no, es que tiene un dolor de cabeza muy fuerte, no es capaz de hablar, vea que está

hiperventilando y "ah, ya va" y se fue. Ya por allá llegó una enfermera a aplicarme una inyección, ni siquiera fueron capaz de decir "ah, no, ella tiene esto, voy a aplicar esto para que le baje el dolor", nada, nada, entonces "doctor, qué es lo que le van a aplicar", "ah, usted quiere que se mejore o quiere seguir así?". Entonces mi prima le dijo, yo quiero saber qué le van a aplicar [inaudible] me dijo disque, usted está con dolor de cabeza o se está haciendo.

[INVESTIGADORA] o sea, la actitud del personal de salud

[C-A06]: no y la fama que [inaudible] con un dolor de cabeza horrible... eh.. le dieron la cita y el médico le mando la farmacia y la muchacha le dio el que no era y se lo tomó y la noche le estaba dando...

[C-A01]: se iba a infartar y era un medicamento muy básico, pero a ella le caía mal, la señora se complicó

[C-A06]: y lo que le dijo ella, no, me equivoqué...

[inaudible]

[C-A01]: pero fue la semana pasada, mi hermana fue antier al hospital y dijo que le habían despachado unos medicamentos...

[C-A06]. y le dijo que ay no que se equivocó, que... tan metidas.

[C-A01]: no, cualquiera se equivoca, sí, pero son cosas de mucho cuidado, porque uno puede...

[INVESTIGADORA] ¿es común ese tipo de equivocaciones?

[C-A02]: a mi una vez, le dije que yo era alérgica al ibuprofeno y me formuló ibuprofeno, yo cuando la muchacha fue yo le dije por qué y no es que aquí en la fórmula, cuando yo le dije al médico que era alérgica, cuando regresé donde él le dije, Doc, me está formulando ibuprofeno y yo soy alérgica al ibuprofeno, y qué te da el ibuprofeno, me da taquicardia, ah eso no es una alergia... entonces imagínese pues... eso es negligencia, tanto enfermeras... todo el personal

9:62 ¶ 213 – 215, in Corresponsabilidad

6:212 ¶ 931 – 932, in Anexo 2 Transcripcion grupo focal de malaria y leishmaniasis con comunidad afrodescendiente.docx

[INVESTIGADORA] porque nosotros escuchamos, pues nos han contado que varios niños se han muerto [inaudible] [C-A01]: ¿señora?

[INVESTIGADORA] que últimamente nos han llegado historias de niños indígenas que han muerto [C-A09]: sí, pero cuando los sacan, ellos ya vienen de una manera ya deshidratados, de todo. Entonces a las mamás indígenas hay que educarlas mucho sobre eso, que si los mismos enfermeros ¡Sáquenlos! No dejarlos tanto tiempo.

9:63 ¶ 216 – 222, in Corresponsabilidad

6:213 ¶ 642 – 647, in Anexo 2 Transcripcion grupo focal de malaria y leishmaniasis con comunidad afrodescendiente.docx

[C-A02]: creería que una de los factores que la gente no va y no va al hospital es la negligencia, la negligencia... aquí se ve mucha negligencia con el trato, al servicio en el hospital eso es pero re malo y eso es lo que más está afectando.

[INVESTIGADORA] ¿pero negligencia quieres decir que no los atienden?

[C-A08]: no, en algunos casos sí los atienden, la cosa es que sí los atienden, pero, cómo le dijera yo, a usted lo atienden y usted va por una cosa lo mira el médico, le manda medicamento y nunca le manda pues un examen que es lo que gente anda buscando y lo mandan para la casa, la persona se toma el medicamento y han habido casos que la persona la mandan para la casa, pasan dos tres días y fallecen

[C-A01]: se complican

[C-A08]: y en otros casos les dan la cita, si la persona va por una cita hoy, se la dan para dos, tres, cuatro meses, la persona, ¿qué va a hacer en todo ese tiempo, entonces es más que todo por eso?

[C-A07]: por eso uno busca particular, es más ligero

10:1 ¶ 7 – 8, in Estigma

3:1 ¶ 296, in AFRO_TB.docx

[A06]: [inaudible] sobre el tabú y el tapujo que llamamos nosotros antes nosotros seguimos agrandándola, porque si... si yo no le digo a él y tenemos relación [inaudible] pero si yo digo, por más [inaudible] pues la gente va a hablar de eso, pero todos a empezar a tomar las medidas de prevención, pero la gente por temor no... [inaudible] como con la citología "que yo no quiero que me vea que..." mientras tanto el problemita va a avanzar. Igual que en los hombres, por temor de decir [inaudible] por temor, porque me ha tocado esos casos [inaudible] muy amiguita con ella y ella me dice, espérate yo lo busco. No menciona mi nombre y ella me trae la solución "hacete esto o esto", pero la gente... es que hay mucho tabú.

10:2 ¶ 9 – 10, in Estigma

3:13 ¶ 382, in AFRO_TB.docx

[A02]: Y a veces no es que la haya pasado a uno, si no que como la gente comenta. Todo el mundo hace comentar y comentar y comentar y entonces uno como que ya se cohíbe de hacer las cosas.

10:3 ¶ 11 – 12, in Estigma

3:15 ¶ 333, in AFRO_TB.docx

[A01]: yo digo que una de las cosas que a veces la gente no divulga porque la habladuría ataca mucho la parte psicológica de la persona y eso hace que la persona se le baje la autoestima y entonces uno que todo el mundo comentando y eso

afecta, entonces yo pienso que si una persona ya, bueno se sabe, ayudarle como en la parte psicológica para que pueda mantener el autoestima y pueda compartir y así como sobrellevar más fácil la enfermedad porque usted sabe que una enfermedad lo acaba a uno mentalmente y si uno se acaba mentalmente la enfermedad también se lo va consumiendo. Entonces esa parte es muy importante.

10:4 ¶ 13 – 14, in Estigma

3:16 ¶ 377, in AFRO_TB.docx

[A02]: Mira lo que decía Marta ahorita de que gente aquí o más que todo las mujeres no iban a examinarse al hospital. A veces vienen brigadas desde pueblo rico, para lo de la citología. Para lo de la prevención a... no asisten las mujeres a hacerse la citología porque ya el miedo está del blabla por ahí, esto y lo otro. Bueno, ¿si me entiendes? Entonces el miedo igual va a estar. Que hay unas que bueno, a la final van... Yo soy unas que cuando vienen de Pueblo Rico no me la hago, no me hago una citología con los funcionarios de aquí, pero cuando vienen de Pueblo Rico.

10:6 ¶ 19 – 22, in Estigma

3:38 ¶ 402 – 404, in AFRO_TB.docx

[A02]: Pero ¿saben cuándo se acaba eso? Esas cosas tienen, esas cosas tienen penalización. Si yo como paciente llego "ay, tengo esto, esto y esto" y se lo conté a usted como médico. ¿Por qué tiene que enterarse digamos, el vigilante del hospital? Si yo le comenté fue a usted.

[A01]: La consulta fue allá a puerta cerrada.

[A02]: Yo me puedo quejar como paciente, es que yo me acerqué donde el joven y le dije tengo esta, esta y esta patología. ¿Por qué se enteró? Entonces cuando nos comencemos a quejar esas cosas empiezan a cambiar. [inaudible]

10:7 ¶ 25 – 28, in Estigma

3:66 ¶ 301 – 303, in AFRO_TB.docx

[A05]: la verdad eso fue secreto, yo me enteré porque yo era compañero de ella y yo le sentí unos síntomas que pues son los de la tuberculosis, ella fue al puesto de salud, de allí la enviaron a Pueblo Rico, digo, para Pereira y pues... le dieron medicamento y todo aislada en su casa un buen tiempo, sí como por tres meses más o menos no podía salir, nada.

[INVESTIGADORA] O sea, ¿fue secreto, pero la gente al ver que estaba aislada no se dio cuenta?

[A05]: no, le hicieron la prueba en Pueblo Rico, de ahí salieron para Pereira y así se la pasó.

10:8 ¶ 29 – 30, in Estigma

3:69 ¶ 491, in AFRO_TB.docx

[A07]: [inaudible] por un contacto sexual, por una [inaudible] ya van a decir [inaudible] la puta del pueblo. Ya van a decir que se metió a un puteadero [inaudible] los casos de tuberculosis que han confirmado han sido de personas que habitan la calle [inaudible], porque saben que ese es el estigma, lo relacionamos con la vagancia [inaudible]

10:9 ¶ 31 – 32, in Estigma

3:75 ¶ 151, in AFRO_TB.docx

[INVESTIGADORA] ¿y han escuchado de algún caso de alguien que diga que sí tiene tuberculosis y cuál ha sido la reacción de la comunidad? [A02]: yo pienso que precisamente es muy parecido como cuando el COVID y por acá se ve mucho que hubieron muchas personas que les dio COVID y por el mismo hecho de qué va a decir la gente o que la gente pueda estar con [inaudible] nadie dice que puede tener tuberculosis, pueden haber, se puede haber presentado casos, pero por lo mismo de qué va a decir la gente o cómo lo van a tomar la gente, nadie dice nada o a veces ni van médico.

10:11 ¶ 35 – 42, in Estigma

3:79 ¶ 370 – 376, in AFRO_TB.docx

[A01]: es lo que yo le decía ahorita, porque eso... cuando una persona...

[A02]: yo le voy a decir a ella y ella le va a ella [inaudible] yo se lo puedo decir a ella de una forma de preocupación, pero ella se lo puede decir a ella como por lo mismo o por chisme o mirá que miedo. [inaudible] ese es el temor de la comunidad.

[A06]: [inaudible] ¿cuál es el deber ser de la gente? Hacer caso y preguntarme venga verdad que... [inaudible] ahí era que estaba el problema, [inaudible] aislando a la persona cómo es que le vamos a ayudarle a que se alivie.

[A01]: pero quitarle ese temor a la gente es muy difícil

[A07]. hubo un caso en el que le tiraron piedras a la casa ¿Pero aquí pasó?

[INVESTIGADORA] ¿Con el COVID?

[A07]: Sí, tiraron piedras a la... Y entonces, la gente no podía salir... De esta casa no podía salir ni a la puer

10:13 ¶ 48 – 49, in Estigma

3:101 ¶ 185, in AFRO_TB.docx

[A03]: yo me acuerdo que como antes, hace unos años, lo viví cuando yo tenía mucho maíz, que hacían la harina de maíz y uno lo veía e iba a las comunidades y lo primero que le pedían era esa harina y cuando ya empezó a verse mucha tuberculosis, yo me acuerdo que mi mamá me decía… regañaba a papá cuando

pasaba un indígena que era amigo y le decía "ay, regáleme un poquito de harina ve". Y mi mamá le decía "Evaristo, deje eso que ellos tienen mucha tuberculosis". Uno con ese miedo de que le fueran a... y la gente mermó de comprarle de pedirle, algunos les pedían y les regalaban tremendos caballos de harina, sabiendo de la manera como manipulaban esa harina, la echaban y todo con su [inaudible] pero cuando empezó a ver mucho caso, uno sí ya tuvo mucha prevención y ya consumirle estas cosas a ellos por miedo a...

10:15 ¶ 54 – 55, in Estigma

3:122 ¶ 297, in AFRO_TB.docx

[A01]: y también porque acá también a veces salen a la luz pública ese tipo de cosas que son tan personales.

10:16 ¶ 56 – 59, in Estigma

3:127 ¶ 379 – 381, in AFRO_TB.docx

[A02]: pero es exactamente por eso, de que llegó el resultado de Marta. Eso pasó por las manos de todos. Y después le llegó a Marta. Cuando yo la entregó este resultado a Marta esto tiene que estar sellado. Y ya está abierto.

[A01]: eso lo que le ponen un ganchito y ese ganchito se lo pueden retirar y vuelven y se lo ponen porque a veces no ve, a veces uno reclama un laboratorio y ya se ve que quitaron el ganchito que pusieron otro.

[A06]: pero aquel que no se mueve del mismo punto, yo le diría que tome consciencia

11:3 ¶ 11 – 14, in Experiencias con la enfermedad

2:41 ¶ 78 – 80, in Indigena TB.docx

[C-I04]: La mamá lo trajo al hospital y el médico [ianudible] que el niño que estaba bien, no estaba enfermo. Y también la mamá lo llevó a la casa otra vez y allá dicen que el niño [inaudible] en la comunidad cuando estaba de la noche y le dio como tos, fiebre...

[X] Asfixia.

[C-I04]: y también se salió por la nariz sangre.

11:13 ¶ 48 – 49, in Experiencias con la enfermedad

2:154 ¶ 72, in Indigena TB.docx

[C-I03]: la abuela del niño que murió, ella es mi prima. Ah, ya. Entonces, como mi prima contó, cuando el niño tenía tos, llevaron al hospital. Del hospital dijeron que el que el niño estaba bien. Que él estaba bien, no tenía enfermedad. Entonces la mamá trajo otra vez a la casa y en la casa quedó más enfermo. Y el otro que también [inaudible]. La mamá llevó a Pueblo Rico [inaudible]

11:16 ¶ 56 – 57, in Experiencias con la enfermedad

3:11 ¶ 161, in AFRO_TB.docx

[A03]: yo creo que acá no lo sentimos tanto porque [inaudible] si hubiera sucedido eso, el temor hubiera sido más, uno de saber... la raza de nosotros, un muerto es acompañado por toda la comunidad y que nos hubiese pasado eso...

11:19 ¶ 62 – 66, in Experiencias con la enfermedad

3:24 ¶ 147 – 150, in AFRO_TB.docx

[A06]:[inaudible] pues cuando yo era muy pequeña sí escuché un tío, pero ya después cuando uno va creciendo y va escuchando experiencias de algunos vecinos o escucha que a alguien le dio, como eso la gente la utiliza como con un mito, un tapujo, entonces uno queda en la mismas, porque a veces la gente... como un misterio ahí para decir "no, es que yo tengo tal enfermedad o fulano tal en mi casa tiene... entonces no,... y entonces todo el mundo [susurro]... entonces como un mito, que uno sabe que los cuidados son los utensilios, utilizarlos [inaudible], un poquito... pero la gente en sí le da como temor decir "ah, en mi casa mi mamá tiene cáncer" no, "tú mamá que tiene?" "ahí..." no... en mi punto de vista, para que los demás sepan qué tiene, qué le sirvió, qué no le sirvió, qué le produjo

[A01]: o en qué le pueden ayudar.

[A06]: pero nosotros no miramos que este remedio, medicamento, hierba, eso sirve y no lo decimos. Yo sí cuando me doy cuenta, "que fulano de tal" que ensaye porque todos los organismos no son los mismos.

[A02]: o a veces tienen las enfermedades y se quedan en la casa y así como dice ella, le dicen al vecino o al amigo "no, es que mi familiar tiene esto" y entonces "no, yo lo manejo en la casa" y entonces como uno tiene el conocimiento o más o menos la idea, "no, eso hay que manejarlo con el médico; consulte porque puede ser peor". A veces cuando consultan el médico ya es muy tarde.

11:20 ¶ 67 – 69, in Experiencias con la enfermedad

3:25 ¶ 4 – 5, in AFRO_TB.docx

¿les ha dado o conocen a alguien en sus familias, en su comunidad que haya tenido tuberculosis?

[A04]: de acá no.

11:21 ¶ 70 – 74, in Experiencias con la enfermedad

3:28 ¶ 318 – 321, in AFRO_TB.docx

[INVESTIGADORA] [A07], hoy de lo que hemos estado hablando es de la tuberculosis, nos hemos expandido un poco en algunos puntos, pero esencialmente de la tuberculosis. entonces, la primera pregunta es si has tenido o conoces a alguien en tu comunidad, en tu familia, que haya tenido tuberculosis.

[A07]: No.

[INVESTIGADORA] ¿A nadie?

[A07]: no

11:29 ¶ 102 – 103, in Experiencias con la enfermedad

3:88 ¶ 21, in AFRO_TB.docx

[A05]: pues acá sí han habido casos, eh... pues la mayoría han sido en población indígena, pero en la población afro también han habido casos de tuberculosis. Pero es más que todo con población adulta.

11:33 ¶ 112 – 113, in Experiencias con la enfermedad

3:98 ¶ 295, in AFRO_TB.docx

[A01] de la comunidad indígena yo sí conozco un caso de VIH y tuberculosis, pero acá en la comunidad Afro, no.

13:2 ¶ 11 – 14, in Percepción de riesgo

1:55 ¶ 61 – 63, in Instituciones_TB.docx

[I-I04] Pues, cuando estuve trabajando, no... no encontraba mucho de síntoma de tuberculosis... eso estaba más bien como poco, poco ahí en lo usuarios.

[INVESTIGADORA]. ¿Y afectaba tú crees más a... había más mestizos, indígenas o afro?

[I-I04] No, a mí me tocaba solamente allá en la zona indígena, pero no. No encontraba mucho de tuberculosis, no.

13:3 ¶ 15 – 17, in Percepción de riesgo

1:68 ¶ 38 – 39, in Instituciones_TB.docx

[INVESTIGADORA] Y en esas veredas, ¿tú crees que hay tuberculosis? ¿has visto? ¿Has escuchado?

[I-M05] No. De pronto en Umacaruté. Porque de resto no...

13:4 ¶ 18 – 19, in Percepción de riesgo

1:81 ¶ 77, in Instituciones_TB.docx

[I-M08] Sí, viejos. Santa Teresa creo que tiene, tiene... Mentuará, Bichuara, no recuerdo que otra vereda, hay unas poquitas veredas que tienen casos.

13:6 ¶ 24 – 25, in Percepción de riesgo

1:102 ¶ 25, in Instituciones_TB.docx

[I-M01] en esas comunidades no está muy presente. Tuvimos un caso en una de las comunidades que ya venía, está siendo tratado, ya está para terminando su tratamiento. Le ha ido muy bien gracias a Dios.

13:8 ¶ 28 – 29, in Percepción de riesgo

1:120 ¶ 75, in Instituciones_TB.docx

[I-M08] no, mira que la tuberculosis no es tanta, tanta. Nosotros tenemos como cuatro, cinco, casos activos.

13:13 ¶ 37 – 38, in Percepción de riesgo

1:178 ¶ 87, in Instituciones_TB.docx

[I-M07] Pues la verdad llegan más que todo mujeres. Sí, la verdad llegan más que todos mujeres. Y nos han llegado dos que están en periodo de gestación. Ya esas han sido, es como las más recurrentes, pero... nada más.

13:14 ¶ 39 – 40, in Percepción de riesgo

1:180 ¶ 47, in Instituciones_TB.docx

[I-A03] mira que en estos momentos no hay, a pesar de la semana pasada estuve en campo, estuve en varias veredas, no vi ninguno.

13:18 ¶ 53 – 55, in Percepción de riesgo

3:4 ¶ 322 – 323, in AFRO_TB.docx

[INVESTIGADORA] y cómo calificas como la situación con la comunidad con la tuberculosis. O sea, de 1 a 5, ¿qué tan frecuente es, 1 siendo no siendo casi nunca, 5 siendo, pasa mucho..?

[A07]: yo fui conductor de ambulancia y aquí en el corregimiento 10 meses y esos 10 meses conocí sólo 3 casos.

13:22 ¶ 63 – 68, in Percepción de riesgo

3:31 ¶ 355 – 359, in AFRO_TB.docx

[FACILITADOR]: bueno, ¿ustedes creen que la gente asistiría más a una reunión de malaria que a una de tuberculosis?

[X] uff, sí.

[X] claro.

[A07]: no habría tal... si hablas de una cosa vendrían si hablas de la otra también...

[A01]: lo que más ataca es lo que la gente más le pone atención...

13:23 ¶ 69 – 70, in Percepción de riesgo

3:33 ¶ 6, in AFRO_TB.docx

[A01] en la comunidad afro, aquí como tal, no. A no ser que haya sido años atrás, pero del tiempo que yo tengo conocimiento, no. eso más que todo aquí se da en las comunidades indígenas. Sí, eso no, nosotros no. No sé si los compañeros de pronto... pero de lo que yo sepa aquí no. Las comunidades indígenas sí, pero las afros no.

13:24 ¶ 71 – 72, in Percepción de riesgo

3:61 ¶ 489, in AFRO_TB.docx

[A05]: para nosotros aquí la tuberculosis es un mito [inaudible] sabemos que existe, pero al 99.9% de la población le dice usted sabe que es tuberculosis... no, qué es, una mujer, es un ánima, qué es.

13:29 ¶ 88 – 92, in Percepción de riesgo

3:125 ¶ 24 – 27, in AFRO_TB.docx

[A01]: Sí, por lo general es allá.

[A05]: sí, es allá.

[A01]: Uno cuando va pa esas comunidades, esas...

[A04] Por eso le dicen a uno que se cuide mucho, porque.... allá sí es verdad.

13:31 ¶ 97 – 102, in Percepción de riesgo

:131 ¶ 277 – 281, in AFRO_TB.docx

[INVESTIGADORA] listo... en general, si tuvieran que darle una calificación de uno a 5, ¿cómo ven la situación de tuberculosis en sus comunidades?

[A01]: 1, de nosotros a casi nadie le da.

[A06] 1 por no darle 0.

[A02]: como para no dejarla descartada.

[INVESTIGADORA] ¿pero sí 1, más cercano al 0?

13:33 ¶ 105 – 108, in Percepción de riesgo

3:141 ¶ 59 – 61, in AFRO_TB.docx

[A01] se ve en los indígenas, pero es que no se dan tantos, no.

[INVESTIGADORA] O sea , incluso en los indígenas, no son así tantos... [A01]: como de la malaria y la leishmaniasis, no. Son más pocos los casos. A acá en Risaralda no, pero en las comunidades del Chocó, sí han fallecido bastante indígena por Tuberculosis. Como no pertenecen allá, eso queda registrado acá.

[A03] no y como allá no... la salud es más mala y ellos tan lejos que viven algunos a vece

13:38 ¶ 130 – 135, in Percepción de riesgo

4:29 ¶ 854 – 858, in INSTITUCIONES ML & LC.docx

[INVESTIGADORA]: también en Bajo San Juan hay harta leishmanisis

[I-A03]: no...

[I-M06]: sí hay, pero no...

[I-A03]: no es tan relevante

[I-M06]: lo que pasa es que como ahorita a está la vía, pues... exacto, pero no...

13:43 ¶ 143 – 146, in Percepción de riesgo

4:105 ¶ 1477 – 1479, in INSTITUCIONES ML & LC.docx

[I-I07]: cuando a veces el niño comienza con síntomas normal, a veces, eso ha pasado, que a veces no se preocupan casi...

[I-I02]: a veces dejan morir

[I-I07]: pero cuando ya... le va a dando [inaudible] ya ahí sí preocupan, ahí sí preocupan, no importa que yo tenga o no tenga plata, ahí tengo que... ahí sí ya... si pasa algo, [inaudible] eso es lo que pasa con el indígena. Entonces usted dice lo de los recursos, no es que solo un rato tiene fiebre y ya, solo una ve, pero uno no sabe.. y el niño va empeorando la salud, se ve el físico como está comiendo y eso es lo que pasa.

13:54 ¶ 201 – 202, in Percepción de riesgo

4:282 ¶ 1473, in INSTITUCIONES ML & LC.docx

[I-M01]: ellos no piensan en eso y tal vez confían de que el niño se va a aliviar o... por ejemplo el caso que yo tenía, porque gracias a dios ya se solucionó y me preocupaba un montón, ella... veníamos luchando mucho con la niña y con ellos, pero ellos no, porque no tenían con quién dejarlo, un niño más pequeño, porque la niña tenía que estar en el centro de recuperación de santa cecilia y entonces ella decía que no que ella no se podía ir con la niña, que porque ella tenía una niña más pequeña, quién la iba a cuidar, que la niña no podía estar sola, entonces por eso ella se negaba a desplazarse con la niña hasta que ya la niña se complicó tanto que ellos vieron la necesidad de que decidieron buscar ayuda.

13:74 ¶ 307 – 313, in Percepción de riesgo

6:25 ¶ 799 – 804, in Anexo 2 Transcripcion grupo focal de malaria y leishmaniasis con comunidad afrodescendiente.docx

[INVESTIGADORA] ¿y leishmaniasis? ¿Hay más casos ahora, hay menos casos?
[C-A09]: no, la leishmaniasis casi no

[C-A01]: casi no se está escuchando ahora, antes casi no se veía varias personas...

[C-A09]: antes sí, por ahora no... y por lo menos en las comunidades indígenas se ven más casos de leishmaniasis que en las comunidades afro, pero los indígenas hay algunos que les dan leishmaniasis, pero ellos no van a los puestos de salud, porque ellos mismos se hacen sus tratamientos. A veces cuando no les funciona, a veces salen cuando ya la enfermedad a veces es´ta muy avanzada, pero casi no.

[INVESTIGADORA] ¿y para la comunidad afro de 1 a 5, ¿qué tanto le da a la gente? [C-A09]: ¿leishmaniasis?

[INVESTIGADORA] 1, es casi nada, 5 es mucho [C-A01]: un 2 por ciento

[C-A02]: eso sí un 2 por ciento

14:9 ¶ 32 – 40, in Propuestas

3:126 ¶ 338 – 345, in AFRO_TB.docx

[FACILITADOR]: ¿y cómo creen que se podría educar en tuberculosis, qué se podría hacer para que se conozca la [inaudible]?

[A01]: capacitaciones es lo ideal. Por medio de actividades que vengan a hacer [inaudible]

[inaudible]

[INVESTIGADORA] pero hablábamos ayer u poco sobre que había poca motivación para asistir a este tipo de eventos, ¿ustedes creen que podría ser más difícil si el tema es tuberculosis para que asistan?

[A01]: es que hay gente que a veces no le para bolas a las cosas.

[INVESTIGADORA] pero también, como hay pocos casos, es probable que la gente diga...

[A01]: como eso casi no pasa acá...

[A02]: no le dan importancia.

14:24 ¶ 72 – 73, in Propuestas

5:95 ¶ 561, in Anexo 1 Transcripcion grupo focal de malaria y leishmaniasis con comunidad indígena.docx

[C-I03]: en caso de la comunidad de nosotros, las mujeres... como le digo... cuando encuentra un taller como hay veces, como los hombres, no más que discriminan nosotras, un ejemplo, si yo estoy recibiendo aquí talleres, si yo voy a la comunidad y por la tarde voy llamando para yo avisarle qué entendí y le estoy enseñando y eso como casi no quiere arrimar, uno desanima y no quiere entender "que ah, que esa mujer qué va a hacer, solamente sale eso". Entonces uno ahí cómo queda, uno quiero llevar una información aquí a la comunidad y ahí queda desanimado, uno queda pensativo, eso no más hace falta.

○ Determinantes sociales_TB

38 Citas:

5:1 ¶ 7 – 8, in Factores económicos

1:2 ¶ 257, in Instituciones_TB.docx

[I-I02] unos dicen, si el niño está enfermo, llévenlo al hospital y dicen no... no hay platica con qué llevarlo al niño al hospital, puedo conseguirlo mañana o pasado mañana [inaudible] las mamás

5:2 ¶ 17 – 18, in Factores económicos

5:125 ¶ 725, in Anexo 1 Transcripcion grupo focal de malaria y leishmaniasis con comunidad indígena.docx

[C-I04]: algunas mamás, algunas mamitas para llevar al hospital, les falta el recurso, porque... un ejemplo, si un enfermara mi hija y no hay recurso, ¿yo con qué voy a llevarla al hospital? No hay recurso. Queda difícil

5:5 ¶ 11 – 12, in Factores económicos

1:108 ¶ 256, in Instituciones_TB.docx

[I-M01] yo pienso que a veces no tienen la facilidad económicamente, o a veces les falta es como la voluntad, porque a veces uno le pone hasta los medios ahí, para que bajen el niño y no, no lo hacen.

7:2 ¶ 9 – 10, in Prácticas culturales

1:30 ¶ 546, in Instituciones_TB.docx

[I-M08] pero si el papá no da la orden, no lo llevan.

7:3 ¶ 14 – 15, in Prácticas culturales

1:46 ¶ 548, in Instituciones_TB.docx

[I-M08] el que manda es el hombre, si él no está ahí, ahí no sé cómo hace, pero la mamá no... que porque no puede disponer, que porque el esposo no está y él es el que manda.

7:5 ¶ 16 – 17, in Prácticas culturales

2:16 ¶ 461, in Indigena TB.docx

[C-I05]: el médico pensará que nosotros no nos alimentamos bien por lo que él tiene alimentación occidental y es diferente a la alimentación de nosotros, por eso debe... pero nosotros comemos alimentación que nos da alimento, por ejemplo las enfermedades que previene, por ejemplo, el que come bastante yuyo, nunca va a

tener dificultad de piel la sangre, algo así, no tiene contaminación, en cambio en la occidental, comemos, pero nos contaminamos.

7:6 ¶ 18 – 20, in Prácticas culturales

2:30 ¶ 373 – 374, in Indigena TB.docx

[C-I02] el primitivito

[INVESTIGADORA] ¿el primitivito y qué más? [C-I02] bananito, yuquita... mucho alimento, la yuca y el maíz...

7:8 ¶ 23 – 24, in Prácticas culturales

2:45 ¶ 468, in Indigena TB.docx

[C-I03]: y no comemos tanta carne, para qué voy a decir mentiras si yo no como carne y arroz [inaudible] porque somos culturas diferentes, yo cuando tengo carne, a veces arroz, a veces con primitivo, porque el primitivo nos hace falta. Siempre lo tenemos de la finca, hacemos jugo, fritamos y ya, con eso...

7:9 ¶ 25 – 26, in Prácticas culturales

2:48 ¶ 467, in Indigena TB.docx

[INVESTIGADORA] eso es por la mañana. [C-I04]: por la mañana tomamos esa harina, así como usted toma la aguapanela, siempre tomamos eso, harina con bequé.

7:11 ¶ 30 – 31, in Prácticas culturales

2:93 ¶ 466, in Indigena TB.docx

[INVESTIGADORA] primitivo, yuyo, ¿cómo cocinan el yuyo? [C-I03]: a través del monte es como una... vino, se llama, vino. Otro es el pringamoso. Y eso los cocinamos, ya, eso no echamos nada, eso lo cocinamos y esa es sal, y con harina y ya.

7:12 ¶ 32 – 33, in Prácticas culturales

2:104 ¶ 463, in Indigena TB.docx

[C-I03]: pues yo he sentido cuando fui a hacer un control del niño, me preguntaba el médico si el niño alimentaba en la mañana huevo con arepa, que si toma chocolate, y yo todo escuché, escuché y a lo último yo contesté al médico, médico sabe qué, usted no sabe que indígena alimentamos diferente, culturalmente tenemos diferente comida típica. Un ejemplo, nosotros como embera, en la mañana desyunamos harina con maíz culturalmente que nosotros hicimos, con una hierba que se llama Bequé, a nosotras, ese es como nuestro chocolate con leche, harina con Bequé, así mismo alimentamos. No alimentamos leche por la mañana, no, tenemos diferente, pero alguno médicos piensan que nosostros desnutrimos los niños por no alimento de occidental. No, es que nosostros tenemos otro alimento que es natural.

7:13 ¶ 34 – 36, in Prácticas culturales

2:119 ¶ 464 – 465, in Indigena TB.docx

[INVESTIGADORA] bueno, entonces en el día, ese es el desayuno, la harina de maíz con bequé, ¿qué más?

[C-I03] y yuyo. Sí, ese es nuestro desayuno y a la nevera también primitivo.

7:14 ¶ 37 – 38, in Prácticas culturales

2:122 ¶ 475, in Indigena TB.docx

[C-I05]: lo que pasa la modernización nos ha cambiado, anteriormente la abuelita nos daba pringamosa con salecita y con primitivito y de encima de sobremesa nos daba harina, y quedaba normal, esa era como que la comida y conserbávamos mucho porque algunos muchachos, por decir algo, me confunden con [inaudible], yo tengo 80 años y yo en mi cabeza [inaudible] por encima, eso trae es de comida, en cambio ahora, yo tengo un sobrinito, sobrinito mío, hombre, por dios se llenaron de... es un niñito que crecieron, eso se traía... de resistencia y y lernarnos igualito, de pronto occidental cree que como yo como pollo me quedé bien, pero comiendo comida típica, queda igual lleno la resistencia la sangra, eso ha pasado.

7:18 ¶ 50 – 55, in Prácticas culturales

2:145 ¶ 368 – 372, in Indigena TB.docx

[INVESTIGADORA] ¿qué más? La buena alimentación... ¿el indígena se alimenta bien, regular o mal?

[C-I05]: el indígena se alimenta bien. Ahora ya está alimentando bien.

[C-I03]: estamos alimentando pura natural.

[C-I01] comida natural

[C-I06] y típica.

7:25 ¶ 81 – 82, in Prácticas culturales

4:113 ¶ 254, in INSTITUCIONES ML & LC.docx

Y que en todo lo que tiene que ver con el hogar, el papá no participa para nada. Para nada. Para nada es para nada; ni en la crianza de los hijos, ni en ir a buscar leña porque eso es trabajo de mujer, ellos lo simplemente llegan, se levanta, trabaja en el campo, llevan los alimentos y ya. Las tardes son para jugar futbol, ¿sí o no? Para reunirse entre compañeros hombre, para reunirse entre hombres y hablar, y hablar y hablar y socializar, esa es la vida del hombre en la humanidad y de ir a proteger el hogar, esa es la razón de ser del hombre, la protección del hogar trabajar la tierra y llevar los alimentos hasta ahí llega el hombre, lo demás, porque ni siquiera la cultura, si ustedes miran las que se pintan quiénes son? La mujer, las que danzan a quiénes son? Las mujeres. ¿las que llevan la crianza de los hijos? Las mujeres. ¿La que llevan la tradición, la tradición oral, toda la tradición? La mujer.

Todo, porque todo, todo todo gira al rededor de la mujer. Todo, todo, todo, el hombre no está sino para procrear, proteger el hogar y llevar la alimentación. Pare de contar. No más. Entonces, ella no tiene tiempo de nada más, y antes son muy verracas que tienen tiempo de buscar leña y tienen tiempo de... Y por eso se ve... exacto y por eso es que usted ve que nosotros tratamos de abordar el tema de que, las niñas son niñas y tienen que estar jugando, pero es que ellas no tienen tiempo, y la responsabilidad de la niña que va creciendo, es cuidar al hermanito. Y no hay otra razón, y por eso, muchas veces que eso ha ido cambiando, es que la mujer se vaya vinculando a la educación, pero antes, no ahorita se ve más, y esto es un loco que se ha logrado a través del tiempo, que la mujer vaya a la educación, porque antes no. Y antes había mucho analfabetismo en las comunidades, ahora las mujeres están un poquito más despiertas. Además, de que a los hombres tampoco le gustan mucho que eso suceda porque, o sea, tu cosmovisión va a cambiar, tu pensamiento va a cambiar y es algo que dentro de los territorios no es tan viable, pero sí ha ido cambiando, y nosotros tenemos que buscar metodologías duales. Por ejemplo, lo que decía el compañero, allá llegó el señor de ratón, vamos a ver qué nos dice el señor del ratón... allá llegó [I-A03] con los bichos, vamos a ver cuáles son los bichos y ahí van... Pero ellos hacen un esfuerzo grandísimo, grandísimo, grandísimo. Entonces los encuentros de la modalidad de bienestar familiar... usted nunca ve un hombre. Y son por la tarde. Y nunca ve un hombre... está ocupado, está arreglando la casa, está haciendo otras cosas que.... entonces nosotros para llegar a ellos hay que ser así: puntual.

7:48 ¶ 195 – 200, in Prácticas culturales

6:197 ¶ 869 – 873, in Anexo 2 Transcripcion grupo focal de malaria y leishmaniasis con comunidad afrodescendiente.docx

[INVESTIGADORA] pero volviendo a lo que estábamos hablando ahorita, ¿ustedes creen que hay menos personas dedicadas al campo? O sea, que tal vez eso sea un factor para que haya disminuido la leishmaniasis.

[C-A01]: Ay no, eso sí

[C-A08]: en los últimos años sí.

[C-A09]: la gente, por lo menos mire, nosotros aquí en Santa Cecilia, la gente no compraba revuelto, usted iba a donde el vecino y el vecino le vendía todo. Ahorita no, el revuelto está carísimo, aquí un kilo de plátano vale 3000 pesos. La gente ya casi no...

[C-A01]: pero vea que últimamente y a finales del año pasado, la gente ha tomado consciencia de eso y nuevamente están sembrando, se está apropiando la gente nuevamente del campo porque ya no, la verdad es que la gente no quería cultivar y la generación de nosostros, ahora, nosotros no trabajamos el campo, antes sí. Pero ya último, por la encarecida y todo la gente está cosechando nuevamente.

8:2 ¶ 9 – 10, in Salud

1:153 ¶ 287, in Instituciones_TB.docx

[I-I04] Pues ellos creen los dos, pues. Sí, los dos. El jaibaná y el médico occidental. Ellos acuden primero al médico que trata con jai, y después ellos vienen a los hospitales. Pero también hay unas enfermedades que cura el jai, y el otro que no cura. Ahí, ellos, como confían, pues ellos, se confían de Jaibaná y a veces que no son capaz de curar, ahí sí se queda más... después vienen ya todos malos los niños ya al hospital.

8:12 ¶ 163 – 164, in Salud

4:339 ¶ 1429, in INSTITUCIONES ML & LC.docx

[I-I02]: acetaminofén, siempre que se siente uno mal, uno compra acetaminofén, para andar con ese...

8:18 ¶ 116 – 123, in Salud

4:213 ¶ 1650 – 1657, in INSTITUCIONES ML & LC.docx

[INVESTIGADORA] de hecho en estos días había uno que estaba era como con fiebre y diarrea, porque esa niña estaba en...

[I-M01]: en el centro de recuperación

[I-A03]: de ahí mismo del pueblo, de ahí mismo, era del barrio Cinto y nos quedamos todos aterrados porque, porque...

[I-M01]: ¿y de Piedras también?

[I-A03]: yo supe de dos niños del barrio Cinto que estaban en el CRN, entonces quedé como así... porque yo dije venga... eso es como general aquí que eso pase, pero era... se me hizo raro que en afro pase algo así, se le hace a uno muy raro, porque generalmente nunca pasa.

[INVESTIGADORA]: ¿con desnutrición?

[I-A03]: eso es muy poquito usted ver un afro desnutrido, eso es un chiripazo, entonces yo me quedé como así...

8:23 ¶ 268 – 271, in Salud

6:65 ¶ 941 – 943, in Anexo 2 Transcripcion grupo focal de malaria y leishmaniasis con comunidad afrodescendiente.docx

[C-A01]: mi papá tiene una balsámica para las lombrices.

[C-A02]: acá la mayoría de Pueblo Rico [inaudible]

[C-A01]: y el ajo y la gasolina son buenísimo, la gasolina con el [inaudible] usted coge un poquito de gasolina, le echa tres goticas de limón y se lo toma y se unta [inaudible] buenísimo

8:25 ¶ 248 – 265, in Salud

6:52 ¶ 949 – 965, in Anexo 2 Transcripcion grupo focal de malaria y leishmaniasis con comunidad afrodescendiente.docx

[C-A08]: en la zona indígena, es un día, ayer estaban a punto de echarnos a todos, un niño de Bajo Gitó, cabeza y...

[C-A01]: yo no sé si el centro fue por dos que habían en Sinaí...

[C-A08]: que yo llevé... salió otro [inaudible]

[C-A01]: en Sinaí vi dos y yo les dije a las den centro

[C-A08]: aquí el cierre y por eso bienestar está que arde

[C-A01]: en La Loma también...

[INVESTIGADORA] ya... eso era lo que nos decían ayer... [C-A08]: ¿el centro? El centro tiene cupo para 10 y está ocupado y está pidiendo cupo...

[C-A01]: pero eso se puede pedir los dos cupos adicionales, eso se puede hacer, eso lo hace la coordinadora.

[C-A08]: ahí hay uno...

[C-A01]: pero solicitar el sobrecupo sí se puede para los 12

[C-A08]: pero mira que los han devuelto, ya que no quieran subir, pero los han devuelto.

[C-A01]: esa gestión sí se hace.

[C-A08]: no sabía, pero ahora sé para pelear.

[C-A01]: hacele, que se pueden los 12, eso se manda un correo.

[C-A04]: ese peladito que yo vi... eso parece una menudencia...

[C-A08]: hay un niño del deportivo que también va para esa misma posición, del deportivo.

[C-A01]: yo estuve saliendo de unas actividades, pues yo les dije, yo cumplí, yo ya no trabajo en el Centro, pero estuve saliendo como 3 meses a campo. Me encontré muchos niños con desnutrición, yo les dije ya si los focalizan o no... ya yo no puedo hacer más de ahí. Y a veces uno les colabora a las mamás, les da el pasaje porque uno ve unos niños que uy no...

8:34 ¶ 31 – 32, in Salud

2:136 ¶ 109, in Indigena TB.docx

[C-I03]: Y creo que... Es que yo creo que por el médico... Yo creo que tiene una remedio... Que puede servir mucho la enfermedad de una persona... de ser humano. Pero lo que pasa... Que el médico siempre cuando va a consultar... Él siempre le da a acetaminofén. Acetaminofén. Y no le da una medicamento que sirva. Eso es para un ratico. Pa un rato sirve el aceterminofen. Pa quitar el dolor. Pero no es para

curar. Es que a mí me duele cuando va al médico... Que me de un acetaminofén. Y sabiendo que en esa... Médico siempre cana por nosotros. Uno por fijao. Pagamos la plata por resguardo Indígena, por nosotros. Y ellos no siente ellos. Solamente ellos le dan siempre acetaminofén, metrodinazol, que valen los dos 4 mil pesos y no le da para un remedio que sirva, que se calme la enfermedad de uno. Eso quería... ...que siempre pasa eso.

8:35 ¶ 33 – 34, in Salud

3:2 ¶ 252, in AFRO_TB.docx

[A06]: pues eso dependiendo, pues sí, yo lo diría como que dependiendo como que del día de que uno se levante con el pie izquierdo, el derecho, el santo, porque sí atienden, pero a veces va por una urgencia porque usted se aguantó el dolor. Aquí está y usted puede tener un dolorcito y usted puede decir, no yo me lo aguanto y no le doy a este dolorcito aquí y en algunas ocasiones usted va a determinado sitio y le dicen "ay, el médico ya se fue". Así esté ahí, por no querer atender, o le dicen "no, es que esto no es una urgencia". Y si usted va es porque ya usted, su nivel de tolerancia con el dolor, usted no lo soporta. Y ya usted se automedicó alguna bebida, alguna cosita que lo [inaudible] ya se lo hizo, entonces en esa parte, pues sí atienden, pero hay unos días, sea aquí o en cualquier otro sitio de este pueblo, de cualquier parte de Colombia, hay un día de que algo sucede allá.

8:37 ¶ 125 – 128, in Salud

4:218 ¶ 1675 – 1677, in INSTITUCIONES ML & LC.docx

[I-I07]: la diarrea con no lavar bien los alimentos, eso pasa, como dicen por ahí. Llegan a comer el alimento, rapidito lavan, rapidito y listo con su cuchillo. A veces eso pasa, se contamina. Y a veces, lo indígenas, a veces, cuando tienen niños de 3 años a veces mandan solos a ensuciar al baño y no acompañan a veces y como el niño, a veces el no limpia eso, entonces ahí está. A veces mete la mano en la boca y ahí sale la... y a veces también se contaminan jugando en el patio y se meten la comida, porque a veces los padres de familia a veces no están pendientes de sus hijos, eso pasa mucho con el indígena a veces. Y la respiración en [inaudible] por la cocina como dijimos ahorita, a veces en la misma pieza hay cocina, digamoslo... se contamina con eso también y a veces también llevan a otra comunidad cuando está serenando.

[INVESTIGADORA]: ¿cuando está qué?

[I-I07]: cuando está serenando, a eso también afecta la respiración y también las mamás a veces fuman cigarro, tienen la mano cerca del niño y ellos se afectan la respiración, eso pasa y de [inaudible] y la IRA y también le da malaria también y leishmaniasis también da, no mucho, pero eso tiene su riesgo en este momento, a veces. Eso es lo que pasa en la comunidad

8:41 ¶ 135 – 141, in Salud

4:269 ¶ 266 – 271, in INSTITUCIONES ML & LC.docx

[I-M01]: Bueno, pues si voy a hablar un poquito. Pues, de parte de mi trabajo y como amiga de ellos, porque pues yo tengo mucho tiempo distinguiendo de los indígenas y he aprendido a conocerlos como tal. Bueno, como ya sabemos, ellos tienen sus propias creencias, ¿cierto? Pero también hay unos que también han ido cambiando esas creencias. Entonces, cuando se enferma un niño, si está bien primero jaibaná, hay unos que primero jaibaná, como hay otros que ya han cambiado esa mentalidad, y van al hospital, ¿cierto? Entonces, ellos, ¿qué dicen? Bueno, llevamos el niño al jaibaná, en cuanto se enferma. Otras veces se demoran para llevarlo al jaibaná. ¿Por qué? Porque quizás no tienen el dinero para levarlo, porque un jaibaná cobra, eso no es gratis. EL jaibaná cobra.

[I-I02]: cobran 30 mil.

[INVESTIGADORA] ¿ah sí?

[I-M01]: sí señora el jaibaná. Otras veces unos cobran 30 mil y otros pueden cobrar más.

[I-I02]: a veces cobran 50.

[I-M01]: Pues dependiendo de lo que tenga el niño, igual hasta una parteras, las parteras también cobran. Todos ellos cobran, eso no es gratis. Entonces, digamos así, llevan el niño, pues los dos primeros días al jaibaná, o los llevan cuando el niño lleva varios días enfermo, cuando el niño ya está muy complicado. Entonces, mientras el jaibaná hace el ritual que hacen ellos, digamos dos o tres días, y que él ve que el niño definitivamente no mejora, lo manda pues al hospital. Ya un poco complicado porque imagínese, todo ese tiempo el niño enfermoo. Entonces es ahí donde el niño llega muy complicado al hospital. Puede que en el hospital lo recuperen o sencillamente ya demasiado tarde.

8:48 ¶ 47 – 48, in Salud

3:111 ¶ 444, in AFRO_TB.docx

[A07]: los extramurales no se dan para el pueblo negro. Sólo se da para las comunidades indígenas.

8:50 ¶ 56 – 60, in Salud

3:117 ¶ 425 – 428, in AFRO_TB.docx

[A01]: y yo digo que hace falta más médicos.

[A07]: claro.

[A01]: somos mucha población para dos médicos y eso genera mucha...

[A07]: somos mucha población para no tener un hospital. Si nuestra estructura física... tenemos el puesto de salud más grande de Colombia, al que se le invirtieron cuatro mil ochocientos... el puesto de salud de Santa Cecilia es el más grande de Colombia. ¿Tú sabias eso? El puesto de salud de Santa Cecilia es el más grande.

8:51 ¶ 61 – 64, in Salud

3:119 ¶ 264 – 266, in AFRO_TB.docx

[A02]: sí, yo que día madrugue y cogí una cita. me la dieron para las 9:30 de la mañana. Eran las 3 de la tarde y no me habían atendido.

[A01] y llega usted a las 9:40 y le dicen que ya no la pueden atender porque...

[A02]: me tocó irme para la casa y luego regresar porque las 3 de la tarde y el médico no me había atendido. Esperando ahí desde las 9 de la mañana. [inaudible] no la cita, yo ya tenía la cita agendada, o sea, el ficho, para sacar la cita a uno lo atienden a las 10 de la mañana y a uno lo atienden normal, pero ya era la atención médica como tal.

8:57 ¶ 80 – 82, in Salud

4:43 ¶ 1420 – 1421, in INSTITUCIONES ML & LC.docx

o solo dan es acetaminofén, pero que no atienden también, el tema de las fichas...

[I-M01]: o que si no es una urgencia

8:59 ¶ 87 – 88, in Salud

4:82 ¶ 1678, in INSTITUCIONES ML & LC.docx

[I-M01]: también de los factores que influyen en cuanto a desnutrición, digamoslo así, eh... las madres sin lunas tienden a darle lactancia materna al niño, ¿cierto? Eso sí ellas son muy juiciosas en eso y la lactancia materna exclusiva es hasta los seis meses, ¿cierto? Pero ella el niño cumple los seis meses y le siguen dando leche materna, entonces eso no le aporta los nutrientes que el niño necesita, entonces ya no van a estar como tan preocupadas de ir a hacerle como la alimentación complementaria que la sopita, que la cremita, sino que si le llegó las 8 y no hay nada [inaudible] entonces ya es la leche, entonces no se preocupan tanto como por una alimentación complementaria que le aporte los nutrientes al niño, sino por esa leche. Entonces esa es una de los factores que lleva a los niños a la desnutrición y la otra es la comida a deshoras, o sea, no son como muy puntuales en el momento de la la alimentación al niño y que tampoco tienen los nutrientes pues... no le aportan los nutrientes suficientes al niño, como... digamos que si tienen primitivo, es primitivo, porque digamos que las condiciones del indígena es muy diferente, ellos no mantienen como todas esas oportunidades que uno tiene, entonces ellos parecen más bien de bajos recursos, digámoslo así, entonces a ellos no les preocupa tanto pues que haya un buen almuerzo o que haya un buen desayuno. Entonces eso lleva como a la desnutrición como tal.

8:62 ¶ 89 – 90, in Salud

4:95 ¶ 237, in INSTITUCIONES ML & LC.docx

y hay responsabilidad de la salud también. Porque la gerente trata de hacer muchas cosas, pero hay muchas cosas que se les salen de las manos, y dentro esas cosas

que se le salen de las manos es que de pronto no consigue un médico o apenas hay uno solo y es el que atiende todo y no le va a dar para hacer todo, y ella tiene la voluntad de hacerlo, pero no da. Entonces también, no es un secreto que tal vez cuando llegan al centro de salud no los atendemos de la mejor manera, entonces se junta todo, todo se junta. Entonces... cuando nosotros abordamos los temas, sobre todo esos casos que yo no quiero ni mencionar aquí, pero que estamos alborotados todos... uno trata de ayudar.

8:64 ¶ 112 – 115, in Salud

4:199 ¶ 1407 – 1409, in INSTITUCIONES ML & LC.docx

[I-I07]: el acetaminofén [inaudible]

[I-I02]: y a veces en el hospital, a veces el niño tiene malaria y...

[I-I07]: si tiene infección o algo así: acetaminofen.

8:67 ¶ 110 – 111, in Salud

4:170 ¶ 278, in INSTITUCIONES ML & LC.docx

los de la modalidad tiene que estar pendiente de 350 beneficiarios, ellos tienen que saber quién está enfermo y quién no... entonces llegamos al hospital y encontramos la barrera, porque no nos articulamos. Entonces, es lo que le pasa a uno "llevemos al niño al puesto de salud... pero resulta de que llega al puesto salud y hay un triaje donde se clasifica y dice que el niño no es una urgencia, pero, que lo que la clasificación la clasificación nos da es para una consulta externa, pero, nosotros dentro de la cosmovisión indígena, sabemos de que si se lo llevan, no va a volver. Entonces bueno, entonces, qué vamos a hacer.

8:71 ¶ 213 – 214, in Salud

6:19 ¶ 666 – 671, in Anexo 2 Transcripcion grupo focal de malaria y leishmaniasis con comunidad afrodescendiente.docx

[C-A06]: por ejemplo, Santa Cecilia como tal, es un puesto de salud, no es un hospital, por ejemplo, el problema aquí es el cambio, de que hoy permanece un médico, mañana permanece otro, así sucesivamente, supuestamente hay un médico para estar las 24 horas, cosa que nunca se ha dado

8:72 ¶ 220 – 221, in Salud

6:36 ¶ 664 – 665, in Anexo 2 Transcripcion grupo focal de malaria y leishmaniasis con comunidad afrodescendiente.docx

[C-A01]: yo digo que hace falta más de personal médico, porqe el personal médico no tiene la capacidad para atender tanta población, son dos médicos para tanta gente, eso les genera estrés mental y de todo.

8:81 ¶ 317 – 323, in Salud

6:206 ¶ 683 – 688, in Anexo 2 Transcripcion grupo focal de malaria y leishmaniasis con comunidad afrodescendiente.docx

[INVESTIGADORA] yo les preguntaba ahorita, los médicos que están trabajando aquí, bueno el personal, ¿son de afuera? [C-A08]: de afuera, ni los de aquí quieren trabajar.

[C-A06]: por ejemplo, hay uno que va a trabajar hasta el 8 de este mes, el flaquito, Daniel, que ya se va.

[INVESTIGADORA] ¿y por qué se va? [C-A06]: porque ya terminó el rural y él ya se va. Ahora viene... o sea que acá el médico acá, especializado en sí, no mandan, no vienen, o sea, no vienen que eso acá es un hueco, que porque eso acá es zona roja.

[C-A08]: y por la fama que se ha generado

[C-A06]: y por la fama que se ha generado, entonces les da hasta miedo venir.

[INVESTIGADORA] ¿qué fama se ha generado? [C-A08]: lo que pasa es que acá se ha generado una fama en [inaudible] entonces esa fama la generan y eso se ha creado que Santa Cecilia es la peor cosa que puede exisitir a nivel regional, entonces muchos médicos también evitan venir.

9:4 ¶ 17 – 18, in Corresponsabilidad

1:53 ¶ 471, in Instituciones_TB.docx

[I-M08] Ellos son muy enfermos de otras cosas, entonces [inaudible] la desnutrición es muy alta, entonces no hay sistema de defensa.

9:27 ¶ 80 – 86, in Corresponsabilidad

3:46 ¶ 270 – 275, in AFRO_TB.docx

[A06]: de 7 a 6 de la tarde funciona.

[A02]: de 7 a 7, siete de la mañana a siete de la noche.

[A06]: no, yo ese día que fui con mi mamá lo primero que me dijeron fue "no es que el médico ya se fue hasta el celador"

[A02]: cuando tiene una urgencia vital el médico se va, pero supuestamente es de 7 a 7, si yo fui ese día a la 1 de la tarde y no estaba el médico y era las 5 de la tarde y no había vuelto, se va.

[A06]: y así estén ahí chateando, como uno no sabe quién es quién... ah no, es que el médico ya se fue y puede estar ahí chateando.

[A02]: entonces que ah, llega alguien para una urgencia, que no que es que el médico se fue a almorzar y llega hasta las 3, si quiere espera, si no pues...

○ Malos comportamientos_TB

34 Citas:

4:3 ¶ 17 – 18, in Comportamientos

1:5 ¶ 443, in Instituciones_TB.docx

[I-A03] porque cuando ellos, vea por ejemplo, cuando ellos van a estornudar y jamás se van a tapar. Sino que de una, ya. Entonces nunca se van a proteger con nada y los tapabocas si estornudan con los tapabocas, entonces se los presta al otro, y entonces ahí está. [Risas]. Es cierto porque es que hemos evidenciado esos casos.

4:4 ¶ 19 – 20, in Comportamientos

1:14 ¶ 499, in Instituciones_TB.docx

[I-M08] ¿pero por qué? ¿por qué? y es que utilizan el humo como repelente para los zancudos. Entonces cuelgan los niños para que no les pique entonces por eso, cuelga a los niños aquí el humo e imagínate

4:7 ¶ 27 – 35, in Comportamientos

1:24 ¶ 444 – 451, in Instituciones_TB.docx

[INVESTIGADORA] ¿y ustedes creen que hay alguna... por ejemplo, con el COVID, que fue tan grave, aprendieron a taparse, a usar tapabocas?

[X] no.

[INVESTIGADORA] ¿y en el confinamiento más..?

[I-M08] no y nos les dio.

[I-M01] ellos lo utilizan cuando salen al hospital porque...

[I-A03] porque les toca y a veces son "póngase el tapabocas", "póngase el tapabocas" y así.

[I-I04] y apenas salen, de una...

[I-A03] y a cuando se sale tiene el tapabocas ahí tirado en el piso, entonces ahí está... No sirve para nada

4:12 ¶ 46 – 49, in Comportamientos

1:54 ¶ 437 – 439, in Instituciones_TB.docx

[I-A03] [inaudible] si les da la capacitación de lavado de manos, en el momento, pero ya después "— ¿y usted se lavó las manos? — ah, se me olvidó" y con eso... usted les dice en la capacitación "se lava las manos" y ya después se van a comer lo

que sea "— ¿usted se lavó las manos? — Ah. se me olvidó". Entonces ya se comieron lo que iba a ser y ya...

[INVESTIGADORA] ¿pero a veces lo hacen?

[I-A03] a veces lo hacen, a veces...

4:13 ¶ 50 – 54, in Comportamientos

1:57 ¶ 433 – 436, in Instituciones_TB.docx

El lavado de manos: ¿La gente se lava en las manos?

[I-M08] Sí, eso sí.

[INVESTIGADORA] ¿Al comer..?

[I-M08] Ah, pues todo el tiempo no. pues...

4:14 ¶ 55 – 56, in Comportamientos

1:58 ¶ 457, in Instituciones_TB.docx

[I-M08] ellos sí se automedican mucho.

4:17 ¶ 61 – 62, in Comportamientos

1:98 ¶ 497, in Instituciones_TB.docx

[I-M08] hay un problema muy grave con ellos con las enfermedades respiratorias: los fogones. Y ellos tienen una técnica que apenas hace poquito nos dimos cuenta y es que ellos ponen el fogón dentro de la pieza, normal y es cocinado y el bebé es colgado en una hamaca al lado del fogón.

4:20 ¶ 67 – 70, in Comportamientos

1:115 ¶ 368 – 370, in Instituciones_TB.docx

[I-M08] Y nosotros, una cosa que hemos aprendido mucho es que no entregamos el tratamiento completo, porque, anteriormente, cuando no teníamos, pues, como mucho conocimiento, nosotros "ah no, tenga sus 40 pastas, sus 50 pastas" y ya, mentiras que se van y se pierden y vaya Encuéntrelos.

[I-M07] van y las vende en Santa. [Risas]

[I-M08] Entonces, nosotros le damos tratamiento para diez días. A los diez días, tiene que volver. Y tráigame el sello, ya vacío para nosotros saber que se lo tomaron. Bueno, ya nosotros así, para poder hacerle un seguimiento.

4:22 ¶ 73 – 75, in Comportamientos

1:145 ¶ 415 – 416, in Instituciones_TB.docx

Por ejemplo, el tapabocas. Ustedes enseñan a que la gente use tapabocas en las comunidades, ¿eso funciona nada, poco o mucho?

[I-A03] No funciona y aparte de eso, a veces se presta los tapabocas. Es cierto. [Risas]

4:23 ¶ 76 – 78, in Comportamientos

1:146 ¶ 401 – 402, in Instituciones_TB.docx

[INVESTIGADORA] ¿Y será que la gente se mejora un poco con el primer tratamiento y se va?

[I-M08] Sí, ellos mejoran con el primer tratamiento.

4:25 ¶ 81 – 85, in Comportamientos

1:157 ¶ 452 – 455, in Instituciones_TB.docx

¿lo usan?

X. No, no, tampoco.

[I-M08] al indígena no le gusta el tapabocas.

[I-M07] y cuando vamos a hablar de estos de tuberculosis, que es el especial del N95, peor... claro, porque ese aprieta más y cubre mucho más.

4:26 ¶ 86 – 91, in Comportamientos

1:159 ¶ 461 – 465, in Instituciones_TB.docx

Bueno... El mantener el distanciamiento cuando hay una persona enferma?

[I-M08] tampoco

[I-M05] no, eso es un respirando...

[inaudible]

[I-I02] [inaudible] metro de distancia y ahí mismo sientan, no ven ni letrero "no debe sentar" [inaudible]

4:27 ¶ 92 – 93, in Comportamientos

1:164 ¶ 496, in Instituciones_TB.docx

[I-M01] de hecho una de las causas de enfermedades respiratorias son esas. Que las casas son muy destapadas, entra mucho frío.

4:28 ¶ 94 – 98, in Comportamientos

1:177 ¶ 348 – 351, in Instituciones_TB.docx

[X] como automedicarse.

[I-I02] tomar muchas pastillas.

[INVESTIGADORA] ¿es algo que hacen ellos? ¿cómo que tomen pastas...? ¿y es común que se automediquen? [I-M08] uno les dice que si ellos les sirvió un medicamento que no se lo vayan a dar al otro porque puede salir alérgico.

[I-A03] pero generalmente eso lo hacen, por ejemplo, si este niño, un ejemplo, le dieron este antibiótico y ese antibiótico le sirvió para una alergia llamémoslo así, entonces a este le van a dar a este, así a este le hizo reacción adversa, igual se lo dieron, ya. Como a este le sirvió entonces le tiene que servir al otro.

4:30 ¶ 101 – 103, in Comportamientos

2:3 ¶ 295 – 296, in Indigena TB.docx

[INVESTIGADORA] bueno, ¿la gente se automedica? [C-I05]: con ese medicamento la gente no se automedica, ese no vale más bien... eso no vale, le quita fiebre, tos tampoco

[C-I05]: hasta se intoxica yo creo, se puede intoxicar.

4:33 ¶ 109 – 110, in Comportamientos

2:17 ¶ 413, in Indigena TB.docx

[C-I07] pero, algunos mamitas es así, pero algunos no cumplen de lavar las manos a los niños. Si el niño está jugando en el patio, a él se le llama y ya se le sirve los platos y los niños para que coman. Pues de mi parte, no es así, cuando yo primero, voy a cocinar los alimentos, yo primero lavo los alimentos bien, espero cocinar y cuando ya voy sirviendo a los niños, les digo, primero lave las manos con agua y jabón, porque a veces se pueden jugar y vienen y si están jugando en una arena, ya con esa arena ponen muchas bacterias en las manos y ya con eso cuando se meten la boca ya se pueden contaminar, entonces primero hay que lavar las manos bien y la mamita también cuando va a servir los plátanos niño, al almuerzo, ya también primero que lavar las manos para servirles a los niños.

4:34 ¶ 111 – 114, in Comportamientos

2:21 ¶ 356 – 358, in Indigena TB.docx

[C-I05]: no, ahora sí. Ya hemos reconectado.

[C-I04]: que si en las comunidades se ponen tapabocas.

[C-I02] no les gusta.

4:37 ¶ 119 – 120, in Comportamientos

2:27 ¶ 361, in Indigena TB.docx

[C-I07] algunos que lo hacen, algunos pues... mi parte, cuando a mí me da gripa siempre cuando voy toser, tapo acá y toso porque delante del niño no puedo toser porque se puede contaminar el niño también porque a veces uno es así, cuando el niño está cuidando en salud y se va, porque los niños siempre se camina, se pone a jugar con los otros más compañeritos cuando está teniendo tos o gripa y ya se

ponen a jugar con él, ya se ponen a jugar y el niño all otro día amanece con tos, con gripa, como ya se contaminó con el otro niño.

4:38 ¶ 121 – 122, in Comportamientos

2:29 ¶ 420, in Indigena TB.docx

[INVESTIGADORA] creo que ayer hablamos de la ventilación de las viviendas, ¿están bien ventiladas? ¿Ustedes qué creen? [C-I06] ¿sí están bien ventiladas? No, Para mí es amarillo, no sé para los compañeros.

4:40 ¶ 127 – 128, in Comportamientos

2:39 ¶ 93, in Indigena TB.docx

[C-I03]: un día como a nosotros dijeron, mi papá, mi mamá llevaba al hospital clínica Pinares, ahí salió tuberculosis, pero el otro día cuando hicieron otro test ahí no salió. entonces después de eso mi esposo me dijo "no, usted la mamá, yo siempre voy a pasear donde la mamá", entonces se han afectado mis hijos o a hasta a mí. Nosotros fuimos al hospital de Pueblo Rico y pedimos cita para que saquen todas las muestras, todas las familias mía de la casa de tuberculosis, a ver si puede afectar también. Y de los hospital dijeron que vinieron el día martes y nosotros vinimos. Y de ahí dijeron, no es el hospital también, es muy... como le digo, no entienden bien a uno. Y dije que tenía cita el martes para sacar todos la muestra. Y qué paso? dijo que... la doctor dijo que no, que la muestra tiene que ser en Pereira. Y con qué plata va a ir toda la familia allá. Entonces yo le dije a mi esposo, no mejor nos vamos, porque yo a Pereira con todos los niños, con qué va a ir el pasaje. Y de ahí, veo otra vez regresarme a la comunidad y otro día pillo cita también. Y pues se entregaba que a usted le dio una cita pa' Pereira [inaudible] y de ahí no podía hacer nada, me quedé así en la casa. Gracias a dios, nosotros de por asfixia no sufrimos mucho.

4:63 ¶ 191 – 192, in Comportamientos

2:113 ¶ 344, in Indigena TB.docx

[C-I05]: quemar los elementos que uno utilizó.

4:67 ¶ 197 – 199, in Comportamientos

2:124 ¶ 352 – 353, in Indigena TB.docx

Por ejemplo, ¿usar el tapabocas le gusta al indígena?

[Varias voces]: no.

4:69 ¶ 202 – 203, in Comportamientos

2:128 ¶ 363, in Indigena TB.docx

[INVESTIGADORA] ¿y el niño sí se deja poner tapabocas? [C-I03]: no, el niño no pone tapabocas

4:78 ¶ 231 – 236, in Comportamientos

[A06]: ¿y con cuánto tiempo habían estado ustedes relacionados?

[INVESTIGADORA] o sea, cuando tú te diste cuenta que tenía los síntomas y todo eso, ¿cuál fue su reacción?

[A05]: no, yo le pregunte que qué pasaba y ella me dijo que tenía pues síntomas.

[INVESTIGADORA] pero ella estaba usando tapabocas, ¿nada? No se te pegó de milagro.

[X] no es que qué le iba a dar

[A05]: a mí no me iba a dar, estábamos los dos en la misma comunidad [inaudible]

4:81 ¶ 239 – 242, in Comportamientos

3:42 ¶ 234 – 236, in AFRO_TB.docx

[A06]: o sea, yo asociaría todo esto como una automedicación, porque ella entiende de los remedios tradicionales entonces yo la busco y ella me va a decir "hágase esto" y si yo veo que esta me dijo "ah, vaya a la droguería" yo voy allá.

[A04]: sí porque el farmacéutico es muy bueno, me recomendó unas pastas que...

[A06]: eso, así me a caiga mal a mí, pero a usted le cayó bien

4:83 ¶ 255 – 259, in Comportamientos

3:60 ¶ 201 – 204, in AFRO_TB.docx

[INVESTIGADORA] ¿este de taparse cuando tocen? [A05]: amarillo, algunas personas...

[A01]: amarillo, porque unos sí, otros no lo hacen, siempre no lo hacen todas las personas

[A05]: uno que ya sabe cómo es la cuestión, ¿cierto?

[A01]: y hay veces que uno ni lo hace, hay veces que [inaudible]

4:84 ¶ 260 – 262, in Comportamientos

3:68 ¶ 181 – 182, in AFRO_TB.docx

[INVESTIGADORA] listo y el tratamiento, ¿creen que la gente va al médico a que le manden su tratamiento normalmente o a veces se automedican porque creen que es una gripa?

[A06]: habitualmente uno sí se automedica porque uno no sabe que es lo que lo empieza atacar. No la que verdaderamente es. Como decimos el cáncer que "a mí me duele aquí y uno gasta plata y tiempo y ya avanzado es que viene el médico a decirle "es que es esto" entonces uno automedicarse eso sí es normal y común, así le digan no. Así sea con las plantas. Eso sí lo usa uno mucho, automedicarse, pero

ya después de que ya a usted le recetaron los medicamentos de la medicina oriental es que uno llega y dice, ah no, es que cuando fulano, yo le daba esto, a parte del cuidado

4:85 ¶ 263 – 267, in Comportamientos

3:71 ¶ 191 – 195, in AFRO_TB.docx

Entonces aquí hicimos las de protección: el tapabocas.

[A01]: yo digo que lo usábamos cuando se sabe que el otro está enfermo, porque de resto... esa es la verdad, entonces pongámosle roja porque...

[X] o amarilla...

[A01]: amarillo.

4:86 ¶ 269 – 271, in Comportamientos

3:77 ¶ 140 – 141, in AFRO_TB.docx

[INVESTIGADORA] ¿y han escuchado que la gente se automedique para la tuberculosis? ¿Qué compran?

[A01]: con lo mismo que le decía ahorita porque los síntomas son gripales, entonces ¿qué hace la gente? Va a la farmacia y compra cosas para la gripa, se inyecta pa la gripa. Acá la gente siente malestar y va y se inyecta porque uno no sabe qué es y lo pone a uno mejor y todo eso, entonces a la farmacia.

4:87 ¶ 272 – 274, in Comportamientos

3:83 ¶ 228 – 229, in AFRO_TB.docx

[INVESTIGADORA] ¿pero no es muy..? ¿Y qué tan común es que la gente se haga un examen para saber si tiene tuberculosis?

[A06]: La verdad que no, porque como le digo, como yo no estoy sufriendo de eso y es no es el problema higiénico sanitario que está funcionando acá [inaudible] la gente no [inaudible] si le da a usted una fiebre hoy, mañana, pasado, mañana, va a hacerse para malaria o el dengue, que sí se sabe es eso [inaudible] pero no es tan común como le da relevancia. Yo me imagino que el día, porque cada cosa tiene un día y mes, pues ahí de pronto coja determinado sitio y vayan den charlas, yo me imagino más que todo en comunidades indígenas que incluso saben que pueden haber un poquito más casos, no muchos, tendría que haber algunos y otros quede charlas más allá, pero nosotros...

4:92 ¶ 288 – 291, in Comportamientos

3:110 ¶ 174 – 176, in AFRO_TB.docx

[INVESTIGADORA] ah listo. Sí, eso tiene sentido. ¿Creen que hay gente que pensaría más fácil que es tuberculosis? Tu ahora nos decías que esta persona

trabajaba en las comunidades, ¿crees que podría haber gente que es más fácil como que [inaudible]?

[A05]: la verdad eso es más que todo por lo del diagnóstico, porque una persona puede que haya tenido la enfermedad y se la pase tosiendo y "ah, eso es una tos normal" y lleve dos tres meses, entonces no tiene como esa sospecha que lleva esa tos es algo que va mucho más allá.

[A01]: como también está la tuberculosis [inaudible] que es la que afecta toda esa parte [inaudible] que quizás por ese lado sí lo haría pensar a uno, pero igual no es tan común, eso tampoco...

4:93 ¶ 292 – 294, in Comportamientos

3:114 ¶ 224 – 227, in AFRO_TB.docx

¿Y las charlas educativas de tuberculosis qué tal? ¿sí hace charlas educativas de tuberculosis? ¿la gente sí va y las escucha?

[A01]: o sea, otras entidades que venga a darlas como tal aquí... que yo sepa no...

4:94 ¶ 297 – 298, in Comportamientos

3:118 ¶ 45, in AFRO_TB.docx

[INVESTIGADORA] ¿y alguna vez han recibido talleres, información, capacitaciones sobre tuberculosis? [A05]: no, de tuberculosis no, acá solo de leishmaniasis y malaria.

4:95 ¶ 308 – 314, in Comportamientos

3:145 ¶ 205 – 210, in AFRO_TB.docx

[INVESTIGADORA] ¿el distanciamiento? [A01]: mm... qué le digo yo, pues póngale amarillo porque aun cuando estábamos en pandemia uno a vece estaba haciendo fila uno encima del otro.

[INVESTIGADORA] entonces amarillo, más o menos.

[A04]: o tomando trago con la misma copa [risas].

[A04]: y con el tapabocas puesto, yo sí vi muchos así que iban a la discoteca disque con el tapabocas y tomaban de la misma copa.

[INVESTIGADORA] bueno, este amarillo en general, pero si alguien tiene tuberculosis ya... [X] claro, uno... hay que tenerlo lejos...

[X] lejos, a metros.

○ Vacios de conocimiento_TB

26 Citas:

1:1 ¶ 9 – 13, in Conocimiento comunidad

2:15 ¶ 244 – 247, in Indigena TB.docx

[C-I01] bueno, mi pregunta es, [INVESTIGADORA], si una persona tiene tuberculosis, por decir un ejemplo, si yo tengo tuberculosis, ¿puedo dormir con mi señora así junto..?

[C-I05] ¿o relaciones sexuales?

[C-I01] ¿se duerme o así normal?

[INVESTIGADORA] ahí tiene que seguir las indicaciones médicas. [C-I03]: pero tiene... hay que hacer un año

1:3 ¶ 18 – 19, in Conocimiento comunidad

2:28 ¶ 193, in Indigena TB.docx

[C-I02]: está recogiendo baciloscopia.

1:9 ¶ 35 – 36, in Conocimiento comunidad

2:55 ¶ 35, in Indigena TB.docx

[C-I03]: Yo. Pues doctora yo la conozco como [inaudible] la tuberculosis, pues había escuchado que la tuberculosis se afecta por medio del humo, o una persona que si fuma cigarrillo también se afecta en ese medio y entonces para no tener tuberculosis tiene que alejar la cocina al niño, tiene que retirar y eso afecta en el corazón o en el apetito que eso tuberculosis siempre afecta en esa enfermedad, eso tiene el lavado de manos [inaudible]

1:12 ¶ 41 – 42, in Conocimiento comunidad

2:77 ¶ 265, in Indigena TB.docx

[C-I02]: más de los 3 si el paciente tiene tuberculosis, tiene que salir en esos tres vasitos, pero si tiene seguro tiene que salir en un solo vaso, ese también sale salido, si la persona está llena de bichos, [inaudible]

1:13 ¶ 44, in Conocimiento comunidad

Hay que tomar muestra baciloscopia seriada, a una persona tres vasitos seriada. ¿Y cómo se consigue? Hay que madrugar madrugadito para lograr ese tos tuberculosis.

1:15 ¶ 45 – 46, in Conocimiento comunidad

2:86 ¶ 211, in Indigena TB.docx

[C-I05]: prevención porque ahí está enseñando como andar con tapabocas, la ruanita, el sombrero, botas largas, pantalon largo. [inaudible]. Prevención.

1:18 ¶ 50, in Conocimiento comunidad

[C-I01]: pues no lo hice en [inaudible], este dibujito [inaudible]. Tuberculosis yo cree que es eso es muy contagioso, de pronto con tos de una persona que tiene tuberculosis, hay que mantener con tapabocas y hay que tener distancia, distancia porque [inaudible] puede afectar a los demás compañeros y también he escuchado que cuando una persona tiene tuberculosis que si utiliza la cuchara que no puede entregar a otro porque va a contagiar. Yo he escuchado eso, ese tuberculosis está muy contagiosos.

1:20 ¶ 53 – 54, in Conocimiento comunidad

2:92 ¶ 263, in Indigena TB.docx

[C-I02]: que con tos en 15 días, toman baciloscopia, tomar como 3 vasitos

1:21 ¶ 55 – 57, in Conocimiento comunidad

2:103 ¶ 61 – 62, in Indigena TB.docx

[C-I04]: si ya está infectado, esa persona se muere.

[C-I06] se puede morir de asfixia por la respiración porque la tuberculosis puede afectar el signo de respiración de uno, por eso puede asfixiarse y se puede morir.

1:23 ¶ 61 – 62, in Conocimiento comunidad

2:109 ¶ 426, in Indigena TB.docx

[INVESTIGADORA] ¿qué diría [C-I05]? ¿La gente conoce los síntomas de tuberculosis o no? [C-I05]: la gente, la mayoría, no la conocen. [inaudible] como si fuera malaria. Como entre eso no más.

1:25 ¶ 68 – 69, in Conocimiento comunidad

2:118 ¶ 342, in Indigena TB.docx

[C-I02]: sí cura antes, demora... uno solo no afecta pero sí te da tiempo [habla en lengua Embera]. Lo que pasa es tos... cuando uno no sabe nada, lo bota en cualquier parte y hay gente que pisa encima, hay posibilidad de un contagio. Pero si usted sabe, hay que cuajarle, o sea, prevención, una botellita, lo botan lejos o se quema. Es mejor quemar

1:26 ¶ 70 – 73, in Conocimiento comunidad

2:125 ¶ 227 – 229, in Indigena TB.docx

[C-I06] los útiles personales pueden ser una prevención

[INVESTIGADORA] ¿qué?

[C-I06] los útiles personales, si tiene pues en caso del diagnóstico de tuberculosis, como los platos...

1:27 ¶ 74 – 76, in Conocimiento comunidad

2:132 ¶ 422 – 423, in Indigena TB.docx

[INVESTIGADORA] ¿la gente conoce los síntomas de tuberculosis?

[Varias voces] no

1:28 ¶ 77 – 78, in Conocimiento comunidad

2:135 ¶ 230, in Indigena TB.docx

[C-I05]: como en el tratamiento hay que tenerle cuidado a la alimentación, de no contagiarse a otras personas... como el plato, la cuchara.

1:31 ¶ 83 – 85, in Conocimiento comunidad

2:147 ¶ 427 – 428, in Indigena TB.docx

¿La gente sabe cómo es el diagnóstico?

[C-I05]: de eso la gente no sabe nada. Sabemos yo como auxiliar.

1:33 ¶ 91, in Conocimiento comunidad

dolor de oído una cosa así.

1:35 ¶ 95 – 97, in Conocimiento comunidad

solamente que hay que tenerle sus utensilios y cosas todo a parte, sí, porque contagia a la otra persona

[A01] Son los fluidos.

[A02] Su plato, su cuchara, su vaso...

1:37 ¶ 101 – 102, in Conocimiento comunidad

[INVESTIGADORA] y como no hay tantos casos, la gente no piensa tanto en tuberculosis, ¿o será que sí? [A01]: no son frecuentes.

[A06]: y los síntomas, algunos síntomas del COVID, a la amigdalitis, cuando estaban muy asociados a la malaria, al del dengue porque le duele a usted aquí, le da fiebre, que dolor en todo el cuerpo, que dolor de cabeza, casi los mismos síntomas.

1:38 ¶ 106 – 107, in Conocimiento comunidad

3:35 ¶ 291, in AFRO_TB.docx

[A02]: no y también es que cuando una persona tiene cáncer, tiene alguna otra enfermedad inmune como el VIH y les da tuberculosis, eso es algo que... mejor dicho, se lo lleva una cosa o se lo lleva la otra, cuando se junta las dos patologías, la persona se complica y hace muy difícil la recuperación.

1:41 ¶ 111 – 114, in Conocimiento comunidad

3:47 ¶ 42 – 44, in AFRO_TB.docx

[INVESTIGADORA] ¿Y saben qué les hacen? O sea, ¿son pastas, son inyecciones, son..?

[A05]: eh... ahí más que todo el médico.

[A01]: inicialmente el tratamiento se inicia intrahospitalario según el avance de la enfermedad, después de los resultados y eso... después de cierto tiempo ya hay un tratamiento que se puede terminar en casa con pastillas, pero eso ya lo determina el médico según lo avanzado que esté la enfermedad.

1:42 ¶ 115 – 116, in Conocimiento comunidad

3:52 ¶ 94, in AFRO_TB.docx

[A05]: sí, falta 1 que... esa... estar alejado de alguien que esté contagiado.

1:45 ¶ 125 – 126, in Conocimiento comunidad

3:84 ¶ 367, in AFRO_TB.docx

[A02]: a cerca de la enfermedad mucha gente no conoce, no tienen ese tipo de conocimiento, lo poco es lo que uno escucha, "ay fulano tiene tuberculosis" o que en el noticiero te digan que hay casos, pero como esa información que uno debe tener para tener esa precaución, no realmente, no... Entonces yo pienso que muchos irían... asistirían al...

1:47 ¶ 130 – 133, in Conocimiento comunidad

3:93 ¶ 335 – 337, in AFRO_TB.docx

[FACILITADOR]: ¿ustedes creen que en sus comunidades la gente tiene conocimiento de qué es la tuberculosis, cómo se trasmite o más bien..?

[A01]: muy poco los que tienen el conocimiento.

[A04]: como no ha sido tan común acá, entonces no...

1:49 ¶ 124, in Conocimiento comunidad

[A01]: o sea, la tuberculosis así la gargoral que es la inflamación de todos estos ganglios, asociado a la fiebre, dolor de cabeza, entonces pues quizás a uno con esa le prestaría más atención, porque la otra es más asociada a la gripe. También hay una tuberculosis ganglial.

1:53 ¶ 155 – 156, in Conocimiento comunidad

3:116 ¶ 331, in AFRO_TB.docx

[A02]: por lo regular el tratamiento es en el hospital, ¿cierto?

1:56 ¶ 167 – 168, in Conocimiento comunidad

3:144 ¶ 21, in AFRO_TB.docx

Pues cómo se transmite, se transmite por vía aérea, cuando una persona toce o estornuda, o también cuando esta persona ya contagiada manipula elementos u objetos pues estas partículas quedan en estos objetos o elementos y una persona sana llega y los utiliza y pues también se contagia
